# ﻿An annotated checklist of fish species described from the Philippines

**DOI:** 10.3897/zookeys.1246.145752

**Published:** 2025-07-21

**Authors:** Rodulf Anthony T. Balisco, Te-Yu Liao

**Affiliations:** 1 Department of Oceanography, National Sun Yat-sen University, Lian-hai Road, Gushan District, Kaohsiung, Taiwan National Sun Yat-sen University Kaohsiung Taiwan; 2 College of Fisheries and Natural Sciences, Western Philippines University, Rafols Road, Santa Monica, Puerto Princesa City, Palawan, Philippines Western Philippines University Puerto Princesa City Philippines

**Keywords:** Biodiversity, endemic species, fish diversity, museum collection, taxonomy, type specimen

## Abstract

A comprehensive list of fish species collected from Philippine waters and taxonomically described from 1770 to 2024 is herein compiled and presented. A total of 1212 species from 156 families were described, of which 828 from 135 families are currently considered valid, 349 invalid, and 34 with unknown or uncertain status. There are also 228 species currently recorded only in Philippine waters, with 85 species found only in estuarine and freshwater areas, and 143 species in the marine habitats. The type information of these species is also listed together with their current valid names and authorships. The checklist also includes approximately 3842 lots containing 12549 specimens scattered in different museums, including 966 holotypes/lectotypes, 2654 lots of paratypes/paralectotypes, 216 lots of syntypes, and 15 lots of neotypes. Forty-five lots were un-cataloged, while approximately 324 lots were believed to have been lost or destroyed during World War II.

## ﻿﻿Introduction

The collection and identification of fish specimen in the Philippines has a long history. It involved several research expeditions led by different renowned ichthyologists, curators, and institutions, with specimens housed in different museums worldwide (e.g., British Museum of Natural History, United States Natural Museum, Academy of Natural Science Philadelphia, etc.). The earliest known record of fish described from Philippine waters dates back to the 18^th^ century by Pallas (1770) who described *Callionymusocellatus*, a dragonet species. Subsequently, Marion de Proce’s work in 1819–1820 described 13 new predominantly marine fish species collected during his expedition to the Philippines (Table 1) (Marion de Procé 1822). Many species could have been described, but a revolt in Manila before his return to Europe largely destroyed many of his specimens. Thus, most of his species’ descriptions were made based on detailed drawings he made earlier (Marion de Procé 1822). After Marion de Proce’s work, a few species were described by other taxonomists after examining museum specimens of different groups (e.g., Cuvier and Valenciennes 1840; Müller 1842; Heckel 1843; Steindachner 1861). Peters (1864, 1865a, 1865b, 1866, 1868) described 17 new species collected in 1859–1860 from the Philippine archipelago, complemented by efforts from Bleeker (1864) and Kner (1864). Further taxonomic advancements occurred during the 1872–1895 period, with 33 new species described based on specimens from museum collections, including the 15 species described by Cartier (1874) based on the collections of Professor Semper.

**Table 1.** Fish species described by Mario de Proce in the early 19^th^ century without known type specimens.

Family	Species	Remarks
Balistidae	*Balistesrotundatus*	Unknown status
Carangidae	*Caranxscutatus*	Unknown status
Clupeidae	*Clupeamanulensis*	Invalid
Gobiidae	*Gobiusrufus*	Unknown status
Holocentridae	*Holocentruszebra*	Nomen oblitum
Labridae	*Labrusbaccatus*	Invalid
Mullidae	*Mullusmanilensis*	Nomen oblitum
Scorpaenidae	*Taenianotesminutus*	Unknown status
Siganidae	*Amphacanthusovatus*	Unknown status
Sparidae	*Dentexelongatus*	Unknown status
Synodontidae	*Saurusdepressus*	Unknown status
Tetraodontidae	*Tetraodoncompressus*	Unknown status
Tetraodontidae	*Tetraodonmanilensis*	Unknown status

Different research expeditions sent to the Philippines have also contributed to the advancement of ichthyological research in the country. For instance, Günther (1872, 1880, 1887) described fishes obtained during the voyage of H.M.S. Challenger in 1873–1876 which collected coastal, open ocean, and deep-water species. Most of the collected specimens, including the type specimens, were deposited in the Natural History Museum, London (BMNH) . On the other hand, the Albatross Expedition in 1907–1910 by the US Government to the Philippines facilitated the mapping of the marine waters and collection of natural history specimens. The majority of the specimens collected by this expedition are housed in the United States Natural Museum (USNM) (Smith and Williams 1999).

At the turn of the 20^th^ century, Dr. F.W. Richardson’s exploration of Lake Buhi in Luzon yielded significant discoveries, including the world’s smallest commercial fish, *Mistichthysluzonensis*, described by Smith (1902a). Thereafter, other American ichthyologists greatly improved the taxonomic works in the Philippines from 1901 to 1940, describing more than half of the total species to date (54.3%). Seale and colleagues made substantial contributions, describing 119 species from various collection periods (Jordan and Seale 1905, 1907; Smith and Seale 1906; Evermann and Seale 1907; Seale and Bean 1907; Seale 1910), while Jordan and colleagues also contributed 51 species (Jordan and Richardson 1908; Jordan and Snyder 1908). The specimens collected from the Albatross Expedition were also the basis of taxonomic works by Smith and Radcliffe which yielded a comprehensive understanding of fish diversity in the Philippines. This resulted in the descriptions of 205 new species from 1911 to 1917 (Radcliffe 1911, 1912a, 1912b, 1913; Smith 1917), and significantly advanced the taxonomic knowledge during this period. Similarly, Fowler and company also used samples from the Albatross Expedition and made greatest contributions, adding 223 species to the list of newly described fish species during that time (Fowler 1918, 1931, 1934, 1938, 1939, 1941, 1943; Fowler and Bean 1922, 1928, 1929, 1930). Meanwhile, Herre and collaborators also contributed significantly from his Philippine and Oriental Expeditions (Herre 1923a, 1923b, 1924a, 1924b, 1924c, 1924d, 1925a, 1925b, 1926a, 1926b, 1926c, 1926d, 1927a, 1927b, 1927c, 1927d, 1927e, 1928, 1932, 1933, 1934, 1936a, 1936b, 1938, 1939, 1940, 1942a, 1942b, 1942c, 1942d, 1943, 1944a, 1944b, 1945a, 1945b, 1945c, 1945d; Herre and Ablan 1934; Herre and Kauffman 1952), with some of the gobioid specimens were examined by Koumans (1940). However, most of the type specimens were deposited in the Bureau of Science Museum (BSM) and vanished during the Japanese occupation, when the BSM building was destroyed during World War II. This destruction resulted in the loss of significant collections, laboratories, and a vast library (Herre 1953a; Pagunsan 2020). After the war, Herre continued his taxonomic efforts to describe new species from the Philippines (Herre 1950, 1952, 1953a, 1953b). In total, Herre described 196 fish species, the majority of which specimens were deposited in the BSM.

From the 1950s to 2010s, it was Randall and colleagues who greatly contributed to this taxonomic era in the Philippines, describing 37 species (Randall 1955, 1956, 1963, 1980, 1981, 1994, 1996a, 1996b, 2001a, 2001b, 2003, 2011; Klausewitz et al. 1978; Randall and Carpenter 1980; Böhlke and Randall 1981; Randall and Ferraris 1981; Randall and Lubbock 1981; Hoese and Randall 1982; Randall et al. 1985, 1987; Choat and Randall 1986; Randall 1988; Randall and Taylor 1988; Randall and Heemstra 1991; Randall and McCosker 1992; Randall and Greenfield 1996, 2001; Randall and Baldwin 1997; McCosker and Randall 2001; Fraser and Randall 2002, 2011; Fraser et al. 2002; Randall and Eschmeyer 2002; Gon and Randall 2003; Hensley and Randall 2003; Randall and Desoutter-Meniger 2007); Randall and Suzuki 2008; Randall 2009; Randall and Allen 2010; Parenti and Randall 2020). Between the 2000s and 2020s, Allen and others continued working on fish taxonomy in the Philippines (Springer and Allen 2001; Allen and Randall 2002, 2006, 2011; Allen and Erdmann 2012, 2016, 2017; Fraser and Allen 2006; Gill et al. 2012; McCosker and Allen 2012). During this period, advancements in fish species identification and description techniques emerged, including the development of molecular methods that significantly improved both the accuracy and the rate of species discovery (Fischer 2013). This was exhibited in the works of Arango et al. (2019), Hata and Motomura (2019), Hata et al. (2021), Huang et al. (2023), Keith et al. (2017), Liao et al. (2022), Maeda and Palla (2015), Maeda et al. (2021a, 2021b), Tea et al. (2016, 2020), and Williams and Carpenter (2015) to name a few. As of May 2024, approximately 3749 fish species have been reported in the Philippines, with 3463 are considered valid species, while the remaining records are classified either questionable or misidentified (Froese and Pauly 2024). Notably, the majority of the specimens (60.5%) have been described or co-described by 12 ichthyologists, with the majority of these specimens deposited in the USNM, Stanford University, Academy of Natural Science Philadelphia, and BMNH, among others (Table 2).

**Table 2.** Top authors with more than 20 fish species described/co-described from the Philippines.

Author	Number of species described/co-described	Inclusive year/s	Museum/s deposited
Henry W. Fowler	233	1912–1952	ANSP, BPBM, CAS-SU, FMNH, Philippine Government^1^, USNM,
Albert W.C.T Herre	196	1912–1953	AMS IB, ANSP, NHMUK (ex BMNH), BSMP^2^, CAS-SU, FMNH, MNHN, Philippines Fish and Game Administration Collection^1^, UMMZ, USNM, ZMA, ZRC
Alvin Seale	119	1905–1910	AMS, ANSP, BSMP^2^, Bureau of Fisheries^1^, CAS, CAS-SU, FMNH, KIZ, Museum in Manila^1^, MCZ, USBF, USNM
Hugh M. Smith	90	1902–1917	NHMUK (ex BMNH), BSMP^2^, CAS-SU, FAKU, USNM
Lewis Radcliffe	82	1911–1913	NHMUK (ex BMNH), CAS-SU, FAKU, USNM
David S. Jordan	51	1905–1910	CAS-SU, MCZ, USNM
John E. Randall	50	1955–2011	AMNH, AMS, ANSP, BLIH, NHMUK (ex BMNH), BPBM, CAS, CAS-SU, FMNH, MNHN, NSMT, ROM, SAIAB, SMF, UPLB, USNM, WAM, YCM, ZUMT
Barton A. Bean	42	1907–1930	ANSP, BPBM, BSMP^2^, FMNH, Philippine Government^1^, USNM
Barton W. Evermann	25	1906–1907	ANSP, BSMP^2^, Bureau of Fisheries^1^, CAS, CAS-SU, FMNH, IU, MCZ, USNM
Gerald R. Allen	23	1972–2017	AMS, NHMUK (ex BMNH), BPBM, CAS, MHNG, UPLB, UPMSI, USNM, WAM, ZRC
Charles H. Gilbert	23	1920	CAS-SU, USNM
Carl H. Hubbs	23	1920	CAS-SU, USNM

^1^ Actual or exact location unknown; ^2^ Samples deposited presumed destroyed (Herre 1953a). Note: For complete list of abbreviations used, see Sabaj (2020).

Herein, we provide an annotated checklist that represents information on all fish species collected and taxonomically described from the Philippine waters from 1770 to 2024. Through a thorough examination of articles containing original species descriptions and cross-referencing data with existing databases, we aim to significantly contribute to the scientific understanding of fish diversity and their distribution in the Philippines. This checklist serves as a comprehensive reference for fish taxonomists and systematists studying Philippine fish fauna, offering valuable insights into species distributions, taxonomy, and ecology. Furthermore, this resource will prove instrumental in conservation efforts, policy development, and education initiatives related to Philippine marine biodiversity. It also opens up avenues for further research opportunities and fosters international collaboration in fish biodiversity studies.

## ﻿﻿Materials and methods

A list of all fish species collected from Philippine waters and taxonomically described as of 31 March 2024 was compiled from several literature. The information of each species, including valid name, authority, type catalogs, localities, and current status, was meticulously examined using Eschmeyer’s Catalog of Fishes (Fricke et al. 2024), the Global Biodiversity Information Facility (GBIF, https://www.gbif.org/), and the Smithsonian Museum Archive (USNM) (https://collections.nmnh.si.edu/; Smithsonian Museum Archive 2024) websites. Only the species whose types (i.e., holotype/ lectotype, paratype/ paralectotype, syntype, and neotype) mentioned Philippine waters as locality were listed after validating the geographical location/s stated in the original descriptions. The general information (e.g., locality, number of specimens, date collected, etc.) of type specimens from the original description were verified on the museum websites where they were deposited. This includes the Smithsonian Institution, National Museum of Natural History (USNM) (https://collections.nmnh.si.edu/), The Academy of Natural Sciences of Philadelphia (ANSP) (http://clade.ansp.org/ichthyology/), Museum of Comparative Zoology, Harvard University (MCZ) (https://mczbase.mcz.harvard.edu/Specimens), Natural History Museum, London (BMNH) (https://data.nhm.ac.uk/search), California Academy of Sciences (CAS-SU) (https://researcharchive.calacademy.org/research/Ichthyology/collection/Index.asp), and French Museum of Natural History (MNHN) (https://science.mnhn.fr/institution/mnhn/collection/ic/item/search). The FishNet 2 website (http://www.fishnet2.net/search.aspx) was also used in verifying some specimens not found in the online catalog of the above museums.

Fish are classified as freshwater species if they inhabit entirely in freshwater environments throughout their lives or rely on freshwater for at least part of their life cycle (e.g., diadromous, amphidromous) (Jamandre 2023). Estuarine fishes are euryhaline species capable of living in marine, freshwater, and estuarine environments, while marine fishes are restricted to marine habitats (Whitfield 2020). The distribution and endemism status of each species were also determined using Alava et al. (2009), GBIF, and Fish Base. In GBIF, we only consider the presence of a concerned species outside the Philippines if there is a preserved specimen deposited in a known museum and a reputable taxonomist confirms the identification. The updated name of orders followed Betancur-R et al. (2017) or Nelson et al. (2016).

In presenting the checklist, the order and family names (after Betancur-R et al. 2017; Nelson et al. 2016) were listed chronologically, except for newly designated orders and families wherein no order and family numbers were designated yet. The nominal taxa within each family were alphabetically listed. The current valid name (i.e., genus and specific name) and updated taxonomic classification followed Fricke et al. (2024). For each nominal taxon, the original name, authority, and year of publication, along with the page of descriptions, and citation of figure/s and plate/s, were listed first. Below the original name, the current valid name and authorship (if different from the original name) were indicated by an equal (=) sign. Additional information provided includes type category, institution code, catalog number, number, sex, and body size of specimens, locality, depth, and date of capture (if available). Body size data, when available, were converted and presented in standard length (SL, mm) unless otherwise specified. To ensure consistency, we converted the depth from its original description (i.e., fathoms to meters). We also verified information from the original description using type catalogs (if available). Institutional codes follow the standards outlined by Sabaj (2020). However, for consistency with previous literature, the former BMNH institution code is retained despite its change to NHMUK. Additionally, any relevant information on the type specimen, including locality and condition based on type catalogs, radiographs, and photos in online catalogs, are also provided under “Remarks”, when available.

**Figure 1.**
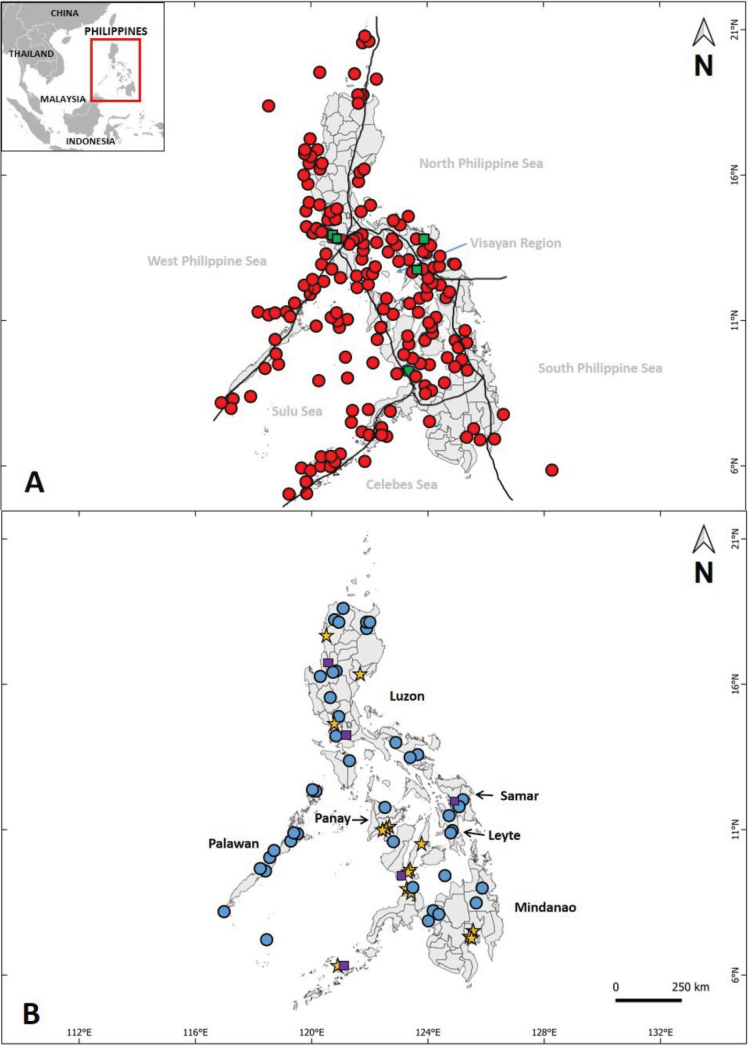
Type localities of valid species described from the Philippines: **A** marine fishes described from major marine biogeographic regions (after Aliño and Gomez 1994); red circles indicate holotypes/ lectotypes, green squares for neotypes, **B** fishes collected from the freshwater and fish markets/ aquarium shops; blue circles indicated holotypes/lectotypes, purple squares for neotypes.

Finally, a distribution map of the type localities (holotype/ lectotype and neotype) was made using the marine biogeographic regions proposed by Alino and Gomez (1994) "for marine species and major islands for freshwater species" as boundaries. (Fig. 1)

## ﻿﻿Results

A total of 1212 fish species belonging to 156 families were collected and taxonomically described from the Philippines between 1770 and 2024. Among these species, 828 from 135 families remained recognized as valid, while 349 are invalid (synonyms), and 34 have unknown or uncertain status. Among the valid species, 228 are considered endemic to the Philippines, with 85 species exclusive to estuarine and freshwater areas (VF), and 143 species inhabiting marine habitats (VM) (Suppl. material 1: tables S1, S2).

Among the valid marine fish species described from major marine biogeographic regions, 307 species were documented from the Visayan Region, 268 species from the West Philippine Sea, 204 species from the Sulu Sea, 63 species from the Northern Philippine Sea, 23 species from the Celebes Sea, and six species from the Southern Philippine Sea. Additionally, 15 species were collected from fish markets, while 30 species had unspecific collection locations within the Philippines. In terms of freshwater fishes, 111 species were described from various major islands, including 38 from Mindanao, 34 from Luzon, 16 from Palawan, five each from Panay and Leyte, and four from Samar, while the rest originated from other islands (Fig. 1).

**Figure 2.**
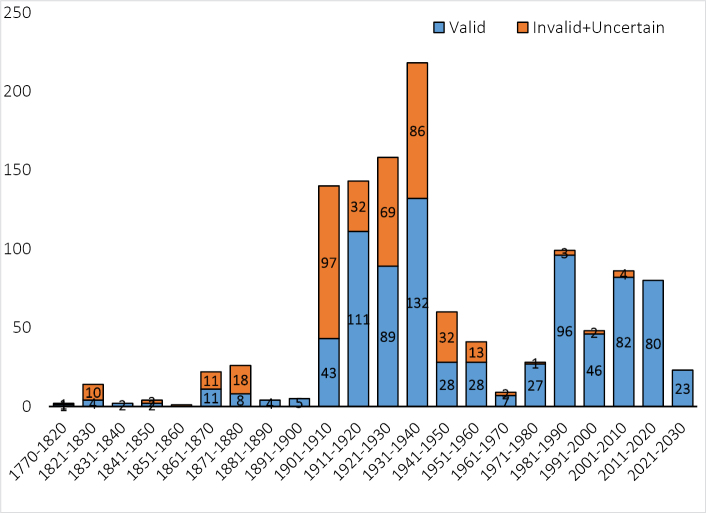
Number of valid and invalid (including uncertain/unknown) fish species described from the Philippines in each decade (as of 31 March 2024).

The number of species described varied per decade, reaching its peak between 1931 and 1940 with 216 described species. However, during this period, a significant proportion of species were classified as invalid or had uncertain/unknown status, accounting for 40.7% of the total described species. In contrast, species described from 1971 to 2024 demonstrate a notable trend, with the majority of described species being validated. Only a small fraction (2.8%) was classified as invalid or with uncertain/unknown status (Fig. 2).

A total of 3842 lots containing 12549 specimens were collected from the Philippines, with the majority (87.1% of lots and 85.7% of specimens) deposited in various museums worldwide. Notably, only 96 lots containing 128 specimens are deposited in Philippine museums, predominantly in the Philippine National Museum (PNM) and Western Philippines University – Puerto Princesa Campus (WPU-PPC-P). Of these specimens, 966 are “holotypes/ lectotypes”, while 2654 lots containing 10426 specimens are paratypes/ paralectotypes. Additionally, 216 lots with 937 specimens are classified as “syntypes”, and 15 lots/specimens serve as “neotypes”. However, 45 lots with 270 specimens remain un-cataloged, and 15 species lack known type specimens. Furthermore, ~ 324 lots containing 1496 specimens were lost or destroyed during World War II (Table 3).

**Table 3.** Number of type series collected from the Philippines and held in world museum collections (as of 31 March 2024). Note: Institution codes followed Sabaj (2020).

Museum	Holotype/ Lectotype	Paratype/ Paralectotype		Syntype		Neotype
	*lot/ spec.*	*lot*	*spec.*	*lot*	*spec.*	*lot/spec.*
***Foreignmuseums***						
AMNH	2	4	5	–	–	–
AMS	6	64	143	–	–	2
ANSP	31	93	158	–	–	3
ASIZP	5	13	22	–	–	–
ASU	–	3	3	–	–	–
BMNH	19	83	134	29	38	–
BPBM	22	71	232	–	–	–
BSKU	2	10	46	–	–	–
CAS	18	118	350	–	–	–
CAS-SU^1^	102	148	1772	8	124	3
CMK	–	8	167	–	–	–
CSIRO	–	9	9	–	–	–
DOS	–	4	4	–	–	–
FAKU	–	1	3	–	–	–
FMNH	3	42	144	–	–	–
FRLM	–	34	39	–	–	–
FSKU	–	1	1	–	–	–
GCRL	–	7	8	–	–	–
IOAN	–	1	1	–	–	–
IU	–	1	1	–	–	–
KAUM-I	10	45	45	–	–	–
KIZ	–	–	–	–	–	1
LACM	1	2	2	–	–	–
LICPP	–	3	12	–	–	–
MCZ	–	6	6	–	–	–
MNHG	–	1	1	–	–	–
MNHN	32	48	61	13	25	–
MSNVR	1	2	2	–	–	–
MUFS	2	6	6	–	–	–
NMMB-P	1	7	12	–	–	–
NMNZ	–	2	2	–	–	–
NMV	–	1	1	–	–	–
NMW	3	–	–	4	6	–
NSMT-P	9	20	53	–	–	–
NTM	–	2	5	–	–	–
QMI	–	2	2	–	–	–
RMNH	1	5	5	1	2	–
ROM	10	69	516	–	–	–
SAIAB	–	5	6	–	–	–
SIO	–	4	11	–	–	–
SMF	2	3	4	–	–	–
SMNHTAU	–	1	6	–	–	–
SMNS	–	3	6	–	–	–
UMMZ	1	6	40	–	–	–
URM-P	–	44	68	–	–	–
USBF	–	2	2	–	–	–
USNM	467	1389	5181	4	38	5
UW	–	1	5	–	–	–
WAM	8	23	100	–	–	–
YCM-HLP		2	2			
YCM-P	–	2	6	–	–	–
YPM	1	1	1	–	–	–
ZIN	1	2	2	–	–	–
ZMA	2	–	–	2	6	–
ZMB	6	1	1	43	130	–
ZMH	2	2	5	1	1	–
ZMMU	3	1	1	–	–	–
ZMUC-P	7	22	23	–	–	–
ZRC	4	16	26	–	–	1
ZSM	–	1	1	–	–	–
ZMUT	1	4	4	–	–	–
**Subtotal**	**784**	**2467**	**9469**	**105**	**380**	**15**
***Philippine museums***						
Philippine Gov’t	–	1	3	–	–	–
PNM	24	11	14	–	–	–
SUML	–	11	11	–	–	–
UPC	–	2	2	–	–	–
UPLB	1	3	11	–	–	–
UPMSI	1	1	10	–	–	–
UPVMI	–	7	7	–	–	–
WPU-PPC	1	32	42	–	–	–
Mus. in Manila	–	1	1	–	–	–
**Subtotal**	**27**	**69**	**101**	**0**	**0**	**0**
***Uncataloged***	10	24	224	11	36	–
***Destroyed/No types known***						
BSM/BSMP	128	93	506^2^	100	521	–
College Agric. Univ.	1	1	126	–	–	–
Philippines Fish & Game Admin. Coll.	1	–	–	–	–	–
***No types known***	15	–	–	–	–	–
**Subtotal**	**145**	**94**	**632**	**100**	**521**	**0**
**Grand total^3^**	**966**	**2654**	**10426**	**216**	**937**	**15**

^1^ Transferred from Stanford University (SU) to California Academy of Science (CAS). ^2^ Minimum number of destroyed specimens. ^3^ Excluding destroyed/lost types and no species without known types.

### ﻿﻿Annotated checklist of fish species collected and described from the Philippines

#### ﻿﻿ORDER MYXINIFORMES (1)

**Family Myxinidae (1)**

**(1) *Eptatretusfernholmi* McMillan & Wisner, 2004: 55, fig. 3**

= *Eptatretusluzonicus* Fernholm, Norén, Kullander, Quattrini, Zintzen, Roberts, Mok & Kuo, 2013.

**Holotype.**USNM 207761 (373.0 TL), Albatross station 5444 (12°43.85'N, 124°58.83'E), Atalaya Point, off Batag Island, east coast of Luzon, 563 m, 3 Jun. 1909.

**Remarks.***E.luzonicus* is a replacement name for *E.fernholmi* which is secondarily pre-occupied by *Paramyxinefernholmi* Kuo, Huang & Mok, 1994 in *Eptatretus*. The specimen was initially lost but found in 2012. Based on USNM’s record, the specimen was dissected and compromised but still intact Smithsonian Museum Archive 2024).

**(2) *Eptatretusstrahani* McMillan & Wisner, 1984: 262, figs. 3(4), 4, 5(11)**

**Holotype.**MNHN 1978-0462 (female, 520.0 TL), Musorstom 1 station 22 (14°0.0'N, 120°18.2'E), near Lubang Island, west of Luzon, 189 m, 21 Mar. 1976.

**Paratypes.**MNHN 1981-722 (1 female, 420.0 TL), SIO 81-116 (1 female, 265.0 TL + 1 male, 450.0 TL), and USNM 227442 (1 male, 465.0 TL), same data as holotype.

**Remarks.** A photograph of the holotype is available in the MNHN record.

#### ﻿﻿ORDER CHIMAERIFORMES (3)

##### Family Chimaeridae (7)

**(3) *Chimaeradeani* Smith & Radcliffe in Smith, 1912: 232, pl. 29**

= *Hydrolagusdeani* (Smith & Radcliffe, 1912).

**Holotype.**USNM 72284 (female, 430.0), Albatross station 5111 (13°45.02'N, 120°46.50'E), off Sombrero Island, west of Luzon, 432 m, 15 Jan. 1908.

#### ﻿﻿ORDER CARCHARHINIFORMES (7)

##### Family Proscylliidae (24)

**(4) *Eridacnisradcliffei* Smith, 1913: 599, pl. 47 (figs 1–3)**

**Holotype.**USNM 74604 (female, 230.0), Albatross station 5135 (06°11.83'N, 121°08.33'E), off Jolo Lighthouse, Sulu Archipelago, 294 m, 7 Feb. 1908.

**Paratypes.**USNM 317570 (2 embryos from holotype; 1 is male, 113.0), same data as holotype. Remarks: The holotype contained two embryos which were removed and re-assigned as paratypes (Smithsonian Museum Archive 2024).

##### Family Pseudotriakidae (25)

**(5) *Gollumsuluensis* Last & Gaudiano, 2011: 19, figs 1–7**

**Holotype.**PNM 15175 [ex SUML JPAG 235] (male, 585.0 TL), Liberty landing site, Bagong Sikat, Puerto Princesa Bay, Palawan Island, ~ 730 m, 16 Mar. 2000. Paratypes: CSIRO H 7193-01 [ex SUML JPAG 229] (male, 575.0 TL), PNM 10294 [ex SUML JPAG 237] (male, 584.0 TL), SUML JPAG 230, 233, and PNM 10295 [ex SUML JPAG 234] (3 females, 534–652.0 TL), same data as holotype.

##### Family Triakidae (27)

**(6) *Hemitriakisleucoperiptera* Herre, 1923a: 71, pl. 1**

**Holotype.** BSMP (955.0 TL), Dumaguete, Negros Island.

**Remarks.** The original description did not indicate the collection date. The type specimen was presumed destroyed.

##### Family Pentanchidae

**(7) *Galeusfriedrichi* Ebert & Jang, 2022: 47, figs 1–5**

**Holotype.**CAS-ICH 247314 (mature male, 534.0 TL), off Sikayab-Bukana (landed location), Dapitan City, Zamboanga, Mindanao, 550 m, 17 Apr. 1999.

**Paratypes.**CAS-ICH 247315 (juvenile male, 455.0 TL), 25 Mar. 2000; CAS-ICH 247316 (female, 413.0 TL), 14 Apr. 1999; both have the same landing location with holotype.

**(8) *Galeusschultzi* Springer, 1979: 67**

**Holotype.**USNM 122312 (female, 297.0), Albatross station 5363, Cape Santiago Lighthouse, Balayan Bay, Luzon, 329 m, 20 Feb. 1909.

**Paratypes.**USNM 122311 (female, 268.0), same locality as holotype, 391 m, 22 Feb. 1909; USNM 122307 (male, 254.0), Albatross station 5111, Sombrero Island, off southern Luzon, 431 m, 16 Jan. 1908.

**Remarks.** The original description indicated the holotype was collected in Albatross 4693 as the station, but different in the USNM record. USNM 122311 was originally composed of two specimens (male and female), but the male specimen was re-assigned to USNM 316614, leaving the female specimen as the only paratype (Smithsonian Museum Archive 2024).

**(9) *Pentanchusherklotsi* Fowler, 1934: 238, fig. 3**

= *Apristurusherklotsi* (Fowler, 1934).

**Holotype.**USNM 93134 (312.0 TL, head damaged), Albatross station 5424 (09°37.08'N, 121°12.62'E), Cagayancillo Island, Palawan, 622 m, 31 Mar. 1909.

**Remarks.** Radiographs of the holotype are available in the USNM record.

**(10) *Pentanchusprofundicolus* Smith & Radcliffe in Smith, 1912: 490, pl. 42**

**Holotype.**USNM 70260 (male, 508.0), Albatross station 5486 (10°02.0'N, 125°19.33'E), Botobolo Point, between Mindanao and Leyte islands, 1070 m, 31 Jul. 1909.

**Remarks.** The holotype is in very poor condition. Photographs and radiographs of the holotype are available in the USNM record.

#### ﻿﻿ORDER SQUALIFORMES (9)

##### Family Centrophoridae (33)

**(11) *Nasisqualusprofundorum* Smith & Radcliffe in Smith, 1912: 681, pl. 53 (fig. 3)**

= *Deaniaprofundorum* (Smith & Radcliffe, 1912).

**Holotype.**USNM 70258 (male, 440.0 TL), Albatross station 5491 (09°24.00'N, 125°12.00'E), Diuata Point, between Leyte and Mindanao islands, 1346 m, 2 Aug. 1909.

**Paratypes.**USNM 99491 (1 female, 590.0), Albatross station 5495, Diuata Point, between Leyte and Mindanao islands, 1785 m, 1 Aug. 1909; USNM 99492 (1 female, 225.0), Albatross station 5527, Balicasag Island, between Siquijor and Bohol islands, 717 m, 11 Aug. 1909; USNM 99493 (1 female, 440.0), Albatross station 5219, Mompog Island, between Marinduque and Luzon islands, 969 m, 23 Apr. 1908; USNM 99495 (1 female, 335.0) and USNM 99497 (1 female, 210.0), Albatross station 5511, off Camp Overton Lighthouse, northern Mindanao, 750 m, 7 Aug. 1909.

**Remarks.** Most paratypes are in poor condition (Howe and Springer 1993).

##### Family Etmopteridae (34)

**(12) *Etmopterusbrachyurus* Smith & Radcliffe in Smith, 1912: 679, pl. 52 (fig. 2)**

**Holotype.**USNM 70257 (male, 227.0 TL), Albatross station 5550 (06°02.00'N, 120°44.67'E), off Jolo Lighthouse, Sulu Archipelago, 472 m, 17 Sep. 1909.

**Remarks.** The caudal fin is missing (Howe and Springer 1993). The original description indicated 481 m as the collection depth.

**(13) *Etmopterusmarshae* Ebert & Van Hees, 2018: 198, figs 1–4**

**Holotype.**PNM 15353 (male, 205.0), 13°46.21'N to 13°48.89'N, 120°50.91'E to 120°50.67'E, between Luzon and Mindoro islands, 322–337 m, 30 May 2011.

**Paratypes.**CAS 234011 (2 males, 150.0–234.0 TL + 8 females, 97.0–192.0 TL), same data as holotype.

##### Family Dalatiidae (37)

**(14) *Squalioluslaticaudus* Smith & Radcliffe in Smith, 1912: 684, pls 50, 54; fig. 4**

**Holotype.**USNM 70259 (male, 150.0 TL), Albatross station 5268 (13°42.00'N, 120°57.25'E), Matocot Point, between Batangas Bay and Verde Island Passage, Luzon, 311 m, 8 Jun. 1908.

**Paratype.**USNM 76679 (1, 115.0 TL), same locality as holotype, 24 Jul. 1909.

**Remarks.** Radiographs and very small dentition material in a small vial in a jar are available for holotype in the USNM record.

##### Family Squalidae (38)

**(15) *Squalusmontalbani* Whitley, 1931: 310**

**Holotype.**USNM 70256 (male, 325.0), Albatross station 5111 (13°45.25'N, 120°46.50'E), off Sombrero Island, west of Luzon, 432 m, 16 Jan. 1908.

**Remarks.** A replacement name for *Squalusphilippinus* Smith & Radcliffe, 1912, which is preoccupied by *Squalusphilippinus* Shaw, 1804.

**(16) *Squalusphilippinus* Smith & Radcliffe in Smith, 1912: 677, pl. 51; fig. 1**

= *Squalusmontalbani* Whitley, 1931.

**Holotype.**USNM 70256 (male, 325.0), Albatross station 5111 (13°45.25'N, 120°46.50'E), off Sombrero Island, west of Luzon, 432 m, 16 Jan. 1908.

**Remarks.** Preoccupied by *Squalusphilippinus* Shaw, 1804; replaced by *Squalusmontalbani* Whitley, 1931.

#### ﻿﻿ORDER SQUATIFORMES (11)

##### Family Squatinidae (40)

**(17) *Squatinacaillieti* Walsh, Ebert & Compagno, 2011: 50, figs 1, 2, 4A, 5A**

**Holotype.**CAS 226473 (female, 328.0 TL), 13°08.98'N–13°09.84'N, 124°04.72'E–124°00.01'E, Albay Gulf, east of Luzon, 363–385 m, 23 Sep. 1995.

#### ﻿﻿ORDER PRISTIOPHORIFORMES (12)

##### Family Pristiophoridae (41)

**(18) *Pristiophoruslanae* Ebert & Wilms, 2013: 87, figs 1–3, 4a**

**Holotype.**CAS 34942 (female, 775.0 TL), ca. 13°23.00'N, 121°07.90'E, northwest of Baltazar Island, Marinduque, 298–307 m, 10 Dec. 1966.

**Paratypes.**CAS 236420 (1 male, 669.0 TL), same data as holotype; CAS 34930 (1 female, 725.0 TL), south of Barrio Salong, Balayan Bay, Luzon, 229–247 m, 18 Jul. 1966.

#### ﻿﻿ORDER RAJIFORMES (14)

##### Family Rajidae (44)

**(19) *Dipturusamphispinus* Last & Alava, 2013: 215, figs 1–10**

**Holotype.**PNM 15178 [SUML JPAG 078] (male, 666.0 TL), Dipolog Public Market, Zamboanga, Mindanao Island, 7 Apr. 1999.

**Paratypes.** SUML BRU 147 (1 female, 878.0 TL), Punta Miray, Baliangao, Misamis Occidental, Mindanao Island, 29 Mar. 2000; CSIRO H 7416-01 [SUML BRU 096] (1 male, 637.0 TL), Palapala, Cadiz, Negros Island, 18 Apr. 1999; SUML MMLM 014 (1 female, 855.0 TL), Dumaguete, Negros Island, 11 Jun. 1999; SUML MMLM 018 (1 female, 896.0 TL) and SUML MMLM 021 (1 female, 805.0 TL), Silliman Beach, Bantayan, Dumaguete, Negros Island, 17–19 Aug. 1999.

**(20) *Okamejeijensenae* Last & Lim, 2010: 102, figs 1–6**

= *Orbirajajensenae* (Last & Lim, 2010).

**Holotype.**PNM 15096 [ex. SUML F 1135] (female, 488.0 TL), ca. 08°28.00'N, 123°20.00'E, Dipolog Public Market, Zamboanga del Norte, Mindanao Island, 8 Apr. 1999.

**Paratypes.** SUML F 1136 (1 female, 518.0 TL), taken with holotype; CSIRO H 7111-01 [ex BRU 169] (1 female, 464.0 TL), Palapala, Cadiz, Negros Island, 6 Apr. 2000.

**(21) *Okamejeipanayensis* Misawa, Babaran & Motomura, 2022: 3, figs 1–9**

**Holotype.** KAUM-I 147882 (male, 304.0 TL), Iloilo, Sulu Sea, Panay Island, 13 Feb. 2013.

**Remarks.** The type specimen was purchased at Iloilo Central Market, Panay Island.

##### Family Arhynchobatidae

**(22) *Notorajasubtilispinosa* Stehmann, 1989: 249, figs 1–4, 6–7**

= *Insentirajasubtilispinosa* (Stehmann, 1989).

**Holotype.**MNHN 1985-0134 (415.0 TL), Musorstrom 2 station 56 (13°54.00'N, 119°55.98'E, off Lubang, west of Luzon, 970 m, 28 Nov. 1980.

#### ﻿﻿ORDER PRISTIFORMES (15)

##### Family Rhinobatidae (45)

**(23) *Rhinobatoswhitei* Last, Corrigan & Naylor, 2014: 33, figs 1–8**

**Holotype.**PNM 15189 [ex SUML F1086 and JPAG 079] (male, 597.0 TL), Dipolog Public Market, Zamboanga, Mindanao Island, 10 Apr. 1999.

**Paratypes.**PNM 10318 [ex SUML JPAG 277] (1 female, 620.0 TL), 6 Apr. 2000; CSIRO H 7542-01 [ex SUML JPAG 300] (1 male, 641.0 TL), 10 Apr. 2000; CSIRO H 7542-02 [ex SUML JPAG 301] (1 female, 494.0 TL), 10 Apr. 2000; CSIRO H 7542-03 [ex SUML JPAG 306] (1 male, 556.0 TL), 10 Apr. 2000; CSIRO H 7542-04 [ex SUML JPAG 308] (1 male, 636.0 TL), 10 Apr. 2000; SUML JPAG 309 (1 male, 471.0 TL), 10 Apr. 2000; CSIRO H 7542-05 [ex SUML JPAG 310] (1 male, 617.0 TL), 1 Apr. 2000; SUML BRU 144 (1, 317.0 TL), 28 Mar. 2000; SUML MMLM 001 (1 female, 764.0 TL), 1 Mar. 1999; SUML F1 [ex MMLM 012] (1 female, 686.0 TL), 1 Apr. 1999. Localities: Suba, Pasil and Pasil Fish Port I, Cebu City; Dipolog City Public Market, Zamboanga, Mindanao; Palapala Fish Port, Bacolod, Negros Island; Palapala Fish Port, Cadiz, Negros Island; Punta Miray, Baliangao, Misamis Occ., Mindanao; Silliman Beach, Dumaguete, Negros Island.

**Remarks.** The original description mentioned many localities but did not mention the specific locality of each paratype.

#### ﻿﻿ORDER NOTACANTHIFORMES (25)

##### Family Halosauridae (71)

**(24) *Halosaurusmediorostris* Günther, 1887: 239, pl. 59 (fig. C)**

= *Aldrovandiamediorostris* (Günther, 1887).

**Holotype.**BMNH 1887.12.7.242 (444.5), Challenger station 207, west of Tablas Island, Romblon, 1280 m, 16 Jan. 1875.

**Other catalog number.** NHMUK:ecatalogue:3112627.

**(25) *Halosauropsisridgwayi* Fowler, 1934: 265, fig. 26**

= *Halosaurusridgwayi* (Fowler, 1934).

**Holotype.**USNM 92334 (354.0 TL), Albatross station 5527 (09°22.50'N, 123°42.67'E), Balicasag Island, between Siquijor and Bohol islands, 717 m, 11 Aug. 1909.

**Paratypes.**ANSP 128419 [ex USNM 93367] (1), Albatross station 5501, and USNM 93369 (1, 69.0 gnathoproctal length), Albatross station 5504, Macabalan Point Lighthouse, northern Mindanao, 366–391 m, 4 Aug. 1909; ANSP 128420 [ex USNM 93368] (1), Albatross station 5508, USNM 93364 (2, 125.0–163.0 gnathoproctal length), Albatross station 5501 and USNM 93366 (1, 157.0 gnathoproctal), Albatross station 5513, Camp Overton Lighthouse, northern Mindanao Island, 494–924 m, 5–7 Aug. 1909; USNM 93365 (1, 150.0 gnathoproctal length), Albatross station 5348, Tabonan Point, Palawan Passage, 686 m, 27 Dec. 1908.

##### Family Notacanthidae (72)

**(26) *Notacanthusabbotti* Fowler, 1934: 267, fig. 28**

**Holotype.**USNM 92349 (260.0 TL), Albatross station 5510 (08°16.00'N, 124°03.83'E), Camp Overton Lighthouse, northern Mindanao, 774 m, 7 Aug. 1909.

**Paratypes.**USNM 93351 (1, 45.0), Albatross station 5293, Escarceo Lighthouse, southern Luzon, 329 m, 23 Jul. 1908; USNM 93352 (1, 75.0), Albatross station 5504, Macabalan Point, northern Mindanao, 366 m, 5 Aug. 1909; USNM 93353 (1, ~64.0), Albatross station 5508, Camp Overton Lighthouse, northern Mindanao, 494 m, 5 Aug. 1909.

**Remarks.**USNM 93351 was drawn and USNM 93353’s head was crushed (Smith 1994).

#### ﻿﻿ORDER ANGUILLIFORMES (26)

##### Family Synaphobranchidae (74)

[**27] *Dysommadolichosomatum* Karrer, 1983: 93, fig. 28A, B**

**Holotype.**MNHN 1979-0004. Madagascar.

**Paratypes.**MNHN 1978-0717 (1, 272.0 TL), Musorstom 1 station 21, off Lubang Island, west of Luzon, 223 m, 21 Mar. 1976.

**(28) *Ilyophisrobinsae* Sulak & Shcherbachev, 1997: 1171, figs 3C, 4B**

**Holotype.** ZMMU P-14759 (348.0 TL), 07°39.90'N, 121°32.00'E, Vitiaz Cruise 57, station 7237, Sulu Sea, west of Mindanao, 4800 m, 28 Feb. 1975.

##### Family Muraenidae (77)

**(29) *Anarchiasreticulatus* Herre, 1923b: 230, pl. 9 (fig. 3)**

= *Uropterygiusmicropterus* (Bleeker, 1852).

**Syntypes.** BSMP (3, 122.0–182.0), Sitanki, Sulu Archipelago.

**Remarks.** The original description did not indicate the collection date of the holotype. All type specimens were presumed lost during World War II (Böhlke and Smith 2002).

**(30) *Diaphenchelyslaimospila* Huang, Smith & Liao in Huang, Smith, Loh and Liao, 2021: 2, figs 1–3**

**Holotype.** NMMBP 26218. Donggang, Pingtung County, Taiwan.

**Paratype.**USNM 407544 (1, 519.0 TL), Sitio Pasiquit, San Vicente, Cagayan, Luzon, 4 Jun. 2012.

**Remarks.** Purchased from a market at Sitio Pasiquit. The original description mentioned 525 mm TL as the size of the paratype.

**(31) *Gymnothoraxannulatus* Smith & Böhlke, 1997: 179, fig. 3**

**Holotype.**ANSP 141418 (female, 442.0), 11°38.33'N, 123°58.63'E, southwest of Caduruan Point, between Negros and Masbate islands, 0–80.5 m, 5 Jun. 1978.

**Paratypes.**ANSP 141417 (3, 343.0–465.0) and USNM 343861 [ex ANSP 141417] (1 male, 400.0), southeast of Tanguingui Island, between Negros and Masbate islands, 0–69.5 m, 5 Jun. 1978; ANSP 141419 (1, 382.0), off Tanguingui Island, between Negros and Masbate islands, 0–75 m, 6 Jun. 1978; BPBM 23497 (1, 501.0), Cebu City fish market, Cebu Island, 31 July 1978.

**Remarks.** Additional description of the holotype was provided by Böhlke and Smith (2002).

**(32) *Gymnothoraxbrunneus* Herre, 1923b: 212**

= *Gymnothoraxherrei* Beebe & Tee-Van, 1933.

**Neotype.**ANSP 164931 (150.0), 13°04.00'N, 120°55.00'E, Analao (Anilao), Batangas, Luzon, 0–3 m, 25 Apr. 1980.

**Remarks.** The original holotype of *G.brunneus* from Puerto Galera, Mindoro Island was destroyed during World War II, and a neotype was selected by Böhlke (2000). *G.brunneus* Herre, 1923 is a junior homonym of *G.brunneus* Nichols, 1920 and was replaced by *G.herrei* (Böhlke and Smith 2002).

**(33) *Gymnothoraxherrei* Beebe & Tee-Van, 1933: 138**

**Neotype.**ANSP 164931, 13°04.00'N, 120°55.00'E Analao (Anilao), Batangas, Luzon, 0–3 m, 25 Apr. 1980.

**Remarks.** Replacement name for *Gymnothoraxbrunneus* Herre, 1923. Neotype selected by Böhlke (2000).

**(34) *Gymnothoraxindong* Seale, 1910: 491**

= *Gymnothoraxzonipectis* Seale, 1906.

**Holotype.** BSMP 4445 (385.0), Zamboanga, Mindanao, 16 Jun. 1908.

**Remarks.** The type specimen was destroyed during World War II (Böhlke and Smith 2002).

**(35) *Gymnothoraxmicrostictus* Böhlke, 2000: 413, figs 2H, 3, 9**

**Holotype.**USNM 357424. Hermit Island, Papua New Guinea.

**Paratypes.**ANSP 141362 (2, 165.0–188.0), NE Bararin Island, Cuyo islands, Palawan, 0–17.4 m, 24 May 1978; ANSP 141368 (3, 96.0–159.0) and ANSP 177773 (1, 184.), Mactan Island, Cebu, 0–30 m, 3 Jun. 1978; ANSP 141379 (1, 111.0), ANSP 141413 (2, 126.0–155.0) and USNM 357428 [ex ANSP 141414] (2, 95.0–134.0), Balicasag Island, Off Bohol Island, 0–24 m, 10–11 Jun. 1978; ANSP 141420 (1, 76.0), Cocoro Island, Cuyo islands, Palawan, 0–21 m, 26 May 1978; ANSP 141421 (1, 97.0), off Bonbonon Point, Negros Island, 0–12 m, 13 May 1978; ANSP 144452 (1, 176.0), Pagnagtaran Point, Puerto Princesa Bay, Palawan Island, 6–12 m, 2 Jul. 1979; ANSP 144454 (1, 90.0), White Beach, Puerto Princesa Bay, Palawan Island, 12–16 m, 14 Nov. 1979; ANSP 144455 (2, 158.0–168.0), same locality and depth as ANSP 144454, 13 Jul. 1979; ANSP 164934 (1, 146.0), ca. 2 km west of Siquijor, Siquijor Island, 24.4–30.5 m, 14 May 1979; ANSP 164935 (9, 89.0–188.0), Caceres Reef near Huisan Point, Cebu, 18 May 1979; ROM 53466 (3, 128.0–178.0), mouth of Bais Bay, Tanon Strait, Negros Island, 15 May 1987; USNM 357427 [ex ANSP 141375] (2, 180.0–213.0), Pamilacan Island, Bohol, 0–24 m, 12 Jun. 1978; USNM 357429 [ex ANSP 141435] (4, 84.0–179.0), reef off White Beach, Puerto Princesa Bay, Palawan Island, 12 m, 14 Nov. 1979.

**(36) *Gymnothoraxphilippinus* Jordan & Seale, 1907: 7, fig. 2**

**Holotype.**CAS-SU 9215 (584.2), Cavite, Luzon, 1900.

**Remarks.** An additional description of the holotype was provided by Böhlke and Smith (2002). A photograph and radiographs are available in the CAS record.

**(37) *Gymnothoraxpseudoherrei* Böhlke, 2000: 408, figs 2F, 3D, 7**

**Holotype.**USNM 357430 [ex ANSP 164928] (female, 147.0 TL), 08°51.40'N, 123°24.60'E, west of Solino Island, Zamboanga, Mindanao, 0–4.6 m, 3 May 1979.

**Paratypes.**ANSP 177775 [ex 164928] (2, 151.0–156.0), taken with holotype; ANSP 164646 (1, 152.0), ANSP 177712 [ex 164644] (7, 115.0–152.0) and ANSP 177769 [ex 177712] (1, 174.0), same locality and depth with the holotype, 3–4 May 1979; CAS 52575 (1, 130.0), Siluag Island, Sulu Archipelago, 23 Jun. 1948; SU 26801 (1, 118.0), Dumaguete, Negros Island, Jun. 1931; ANSP 144451 (7, 102.0–148.0), Puerto Princesa Bay, Palawan Island, 7–13 m, 3 Jul. 1979; USNM 357432 [ex ANSP 144456] (2, 115.0–124.0), Pagnagtaran Point, Puerto Princesa Bay, Palawan Island, 7–13 m, 2 Jul 1979.

**Remarks.** Additional description of the holotype was provided by Böhlke and Smith (2002).

**(38) *Gymnothoraxpseudokidako* Huang, Loh & Liao in Huang, Smith, Loh and Liao, 2021: 7, figs 5–8, 9A, C, E. G**

**Holotype.** ASIZP 0080920. Fugang, Taitung County, Taiwan.

**Paratype.**USNM 438035 (1, 637.0 TL), Dumaguete, Negros Island, 11 May 2015.

**Remarks.** The paratype was bought from Dumaguete City Fish Market. The original description indicated the paratype size as 618.0 TL, different from the museum’s record (Smithsonian Museum Archive 2024).

**(39) *Gymnothoraxsamalensis* Seale, 1910: 492**

= *Gymnothoraxchilospilus* Bleeker, 1864.

**Holotype.** BSMP 3781 (220.0), Samal Island, Davao Gulf, Mindanao, 1 May 1908.

**Remarks.** The type specimen was destroyed during World War II (Böhlke and Smith 2002).

**(40) *Uropterygiusmactanensis* Huang, Balisco, Evacitas & Liao, 2023: 593–602, figs 2, 4–6, 8**

**Holotype.**PNM 15711 (male, 283.0 TL), 10°14.81'N, 123°56.28'E, intertidal zone of Cordova, Mactan Island, Cebu, 27 Dec. 2018.

**Paratypes.** UPC A0521-1 (1 male, 255.0), UPC A0521-2 (1 male, 242.0), AMS I.50833-001 (1 male, 276.0), AMS I.50833-002 (1 female, 242.0), AMS I.50833-003 (1 male, 341.0), NMMB-P37431 (1 male, 341.0), NMMB-P37432 (1 male, 293.0), NMMB-P37433 (1 male, 260.0), NMMB-P37434 (1 female, 231.0), PNM 15712 (1 female, 264.0), PNM 15713 (1 male, 342.0), USNM 445727 (1 male, 232.0), USNM 445728 (1 male, 261.0), USNM 445729 (1 male, 331.0), ZRC 63647 (1 male, 234.0), ZRC 63648 (1 male, 280.0), ZRC 63649 (1 male, 271.0), all taken with the holotype; DOS 08972 (1 female, 245.0), DOS 08973 (1 male, 240.0), and DOS 08974 (1 male, 238.0), same locality as the holotype, 25 Aug. 2015.

##### Family Ophichthidae (80)

**(41) *Benthenchelyscartieri* Fowler, 1934: 267, fig. 29**

**Holotype.**USNM 92356 (115.0 TL), Albatross station 5185 (10°05.76'N, 122°18.50'E), Lusaran Lighthouse, between Panay and Negros islands, 1006 m, 30 Mar. 1908.

**Paratypes.**USNM 93349 (4), same data as holotype.

**Remarks.** The original description mentioned 1167 m as the collection depth but different from the USNM record. Radiograph and vertebral count of the holotype are available in the USNM record.

**(42) *Brachysomophisumbonis* McCosker & Randall, 2001: 28, figs 5G, 7**

**Holotype.**USNM 364282, 12°56.00'N, 121°43.80'E, Batuanan Point, Maeste de Campo Island, Concepcion, Romblon, 14 m, 29 May 2000.

**Remarks.** Although the type locality is near Mindoro Island, it is under Romblon’s jurisdiction. Both the original description and USNM did not provide the size of the type. Photographs are available in the USNM record. The specimen is currently in USNM but will be deposited in the Philippine National Museum (Smithsonian Museum Archive 2024).

**(43) *Caeculamindora* Jordan & Richardson, 1908: 239, fig. 4**

= *Lamnostomamindora* (Jordan & Richardson, 1908).

**Holotype.**CAS-SU 20209 (381.0), Mindoro Island, 1908.

**Remarks.** A photograph and radiograph are available in the CAS record.

**(44) *Caeculaphilippinensis* Herre, 1936b: 358, pl. 1 (fig. 1)**

= *Yirrkalaphilippinensis* (Herre, 1936).

**Holotype.**CAS-SU 30977 (365.0), Dumaguete, Negros Island, 2 Dec. 1933.

**Remarks.** A photograph and radiograph are available in the CAS record.

**(45) *Caeculataylori* Herre, 1923b: 183, pl. 6 (fig. 2)**

= *Lamnostomataylori* (Herre, 1923).

**Holotype.** BSMP (164.0), Cabatoan River, near Iba, Zambales, Luzon, Jan. 1922.

**Remarks.** The type specimen was lost (Kottelat 2013).

**(46) *Callechelysmyersi* Herre, 1932: 139**

= *Bascanichthysmyersi* (Herre, 1932).

**Holotype.**CAS-SU 26828 (788.0), Dumaguete, Negros Island, 15 Jun. 1931.

**(47) *Chlevasteselaps* Fowler, 1912: 13, fig. 3**

= *Myrichthyscolubrinus* (Boddaert, 1781).

**Holotype.**ANSP 1001 (704.9), Philippines.

**Remarks.** The original description or ANSP record did not indicate the specific locality and collection date of the holotype.

**(48) *Jenkinsiellanectura* Jordan in Jordan & Seale, 1907: 6, fig. 1**

= *Cirrhimuraenatapeinoptera* Bleeker, 1863.

**Holotype.**CAS-SU 9984 (190.5), Cavite, Luzon, 1907.

**Remarks.** A photograph and radiograph are available in the CAS record.

**(49) *Jenkinsiellaoliveri* Seale, 1910: 493**

= *Cirrhimuraenaoliveri* (Seale, 1910).

**Holotype.** BSMP 4299 (360.0), Zamboanga, Mindanao, 2 Jun. 1908.

**(50) *Leiuranuslithinus* Jordan & Richardson, 1908: 238, fig. 3**

= *Ophichthuslithinus* (Jordan & Richardson, 1908).

**Holotype.**CAS-SU 20211 (304.8), Cuyo Island, Palawan, 1908.

**Remarks.** A photograph and radiograph are available in the CAS’s record.

**(51) *Muraenichthyselerae* Fowler, 1934: 278, fig. 38**

**Holotype.**USNM 92348 (156.0), Albatross station 5131, island off Panabuton Point, Sulu Sea, off west of Mindanao, 49 m, 6 Feb. 1909.

**Remarks.** Probable synonym of *Scolecenchelysgodeffroyi* (Regan, 1909) (Castle and McCosker 1999). A radiograph and vertebral count are available in the USNM record.

**(52) *Muraenichthysmalabonensis* Herre, 1923b: 157, pl. 2 (fig. 1)**

= *Muraenichthysthompsoni* Jordan & Richardson, 1908.

**Syntypes.** BSMP 839 (175.0), BSMP 840 (193.0), BSMP 841 (172.0) and BSMP 842 (117.0), milkfish pond at Malabon, Manila Bay, Luzon.

**Remarks.** The original description did not indicate the collection date of the syntypes. All type specimens were lost (Kottelat 2013).

**(53) *Muraenichthysphilippinensis* Schultz & Woods, 1949: 173, fig. 2**

**Holotype.**USNM 134951 (92.0 TL), Albatross station 5206 (11°31.67'N, 124°42.67'E), Badian Island, off west of Samar Island, 59.5 m, 14 Apr. 1908.

**Paratype.**USNM 134952 (1, 119.0), Taal anchorage, Balayan Bay, Luzon, 20 Feb. 1909.

**Remarks.** Radiograph and vertebral count are available for the holotype in the USNM record.

**(54) *Muraenichthysretropinnis* Fowler, 1934: 277, fig. 37**

= *Schultzidiaretropinnis* (Fowler, 1934).

**Holotype.**USNM 92355 (114.0 TL), Albatross station 5208 (11°45.88'N, 124°42.83'E), Taratara Island, off west of Samar Island, 47.5 m, 14 Apr. 1908.

**Paratypes.**USNM 134953 (1, 57.0), Albatross station 5507, Camp Overton, northern Mindanao, 777 m, 5 Aug. 1909; USNM 134954 (1, 66.0), Port Binanga, off southern Luzon, 777 m, 8 Jan. 1908.

**Remarks.** The original description mentioned four paratypes but provided no information. USNM 134953 and USNM 134954 could be paratypes (status questionable) as these have the same accession as the holotype, and no paratypes were deposited at CAS-SU or ANSP (Böhlke 1953). These have been cataloged after the description was published but could have been available to Fowler when the manuscript was written (Smith 1994). A radiograph and vertebral count are available for the holotype in the USNM record.

**(55) *Muraenichthysthompsoni* Jordan & Richardson, 1908: 237, fig. 1**

**Holotype.**CAS-SU 20201 (95.3), Manila Bay, Luzon.

**Remarks.** The original description or CAS did not indicate the collection date. A photograph and radiographs are available in the CAS record.

**(56) *Muraenichthysvelinasalis* Hibino & Kimura, 2015a: 65, figs 2b, 3f, 4, 5a–c**

**Holotype.**USNM 313976. Bay between Ken-Ting and Ta-Yuan Shan, Taiwan.

**Paratypes.**USNM 396145 (1, 123.8 TL), BUS 03-18, Coron Island, Palawan, 15–31 m, 7 Mar. 2003.

**(57) *Myrichthyspaleracio* McCosker & Allen, 2012: 36, figs 1–3a + front cover**

**Holotype.**CAS 233313 (female, 311.0), 13°41.28'N, 120°50.46'E, Layag-Layag, Batangas, Luzon, 15 May 2011.

**Paratype.** WAM 33154.001 (female, 315.0), Caban Island, Verde Island Passage, Batangas, Luzon, 33 m, 12 Jun. 2009.

**(58) *Ophichthusmanilensis* Herre, 1923b: 176, pl. 5**

**Holotype.** BSMP 9477 (672.0), Cavite, Luzon.

**Paratype.** BSMP 9488 (1, 515.0), Tondo Market, Manila, Luzon.

**Remarks.** The original description did not indicate the collection date. All type specimens are presumed destroyed.

**(59) *Ophichthustomioi* McCosker, 2010: 32, figs 32–35**

**Holotype.**CAS 214208 (male, 390 mm), 12°54.48'N, 124°23.60'E, San Bernardino Strait, Luzon, 376–382 m, 23 Sep. 1995.

**(60) *Pisoodonophiscopelandi* Herre, 1953b: 10**

= *Pisodonophiscopelandi* Herre, 1953.

**Holotype.**USNM 202516 [ex UW 6485] (308.0), off Dewey Blvd., Manila Bay, Luzon, 21 Nov. 1947.

**Paratype.**USNM 202571 [ex UW 19694] (1, 303.0), same data as holotype.

**Remarks.**USNM record noted two specimens for the paratype, including radiograph, photographs, and vertebral count.

**(61) *Pisoodonophismacgregori* Jordan & Richardson, 1908: 238, fig. 2**

= *Pisodonophiscancrivorus* (Richardson, 1848).

**Holotype.**CAS-SU 20210 (254.0), Manila, Luzon.

**Remarks.** The original description or CAS record did not indicate the collection date. A photograph and radiographs are available in the CAS record.

**(62) *Pylorobranchushearstorum* McCosker, 2014: 334, figs 1–4**

**Holotype.**CAS 235464 (female, 1218 TL), 10°34.98'N–10°34.50'N, 120°22.92'E–124° 23.60'E, Verde Island Passage, 892–966 m, 31 May 2011.

**(63) *Scolecenchelysbrevicaudata* Hibino & Kimura, 2015b: 10, figs 1e, 2e, 4b**

**Holotype.**USNM 134960 (314.0 TL), 12°12.35'N, 124°02.48'E, between Samar and Masbate islands, 246 m, 13 Mar. 1909.

**Remarks.** The original description mentioned USNM 134960 as the holotype but was not found in USNM record. It may still be under the author’s care and have not been deposited in USNM yet.

**(64) *Skythrenchelyszabra* Castle & McCosker, 1999: 116, figs 1A–C, 2A**

**Holotype.** NMNZ 35152. Thevara, Ernakulam, southern India.

**Paratypes.**MNHN 1998-0681 (1, 296.0 TL), Musorstom 2 station 2, Manila Bay, Luzon, 182–187 m, 19. Mar. 1976; USNM 148574 (1, 133.0 TL), Iloilo, Panay Island, 20 Mar. 1929.

##### Family Nettastomatidae (82)

**(65) *Nettastomasolitarium* Castle & Smith in Smith, Böhlke and Castle, 1981: 548, figs 2–7**

**Holotype.**MNHN 1979-0187 (female, 457.0), Musorstom 1 station 50 (13°49.20'N, 120°01.98'E), west of Cabral Island, Mindoro, 415–510 m, 25 Mar. 1976.

**Paratypes.**MNHN 1979-0188 (1, 465.0), Musorstom 1 station 43, 484–448 m; MNHN 1978-0189 (1, 405.0); Musorstom 1 station 44, 592–610 m, 24 Mar. 1976; all from Verde Island Passage, Batangas, Luzon.

**(66) *Saurenchelystaiwanensis* Karmovskaya, 2004: S27, fig. 14**

**Holotype.**MNHN 1998-0668 (342.0 TL), Musorstom 2 station 69 (14°05.10'N, 120°01.98'E, off Lubang Island, west of Luzon, 1800–1950 m, 20 Nov. 1980.

**Remarks.** The geographical coordinates indicated in the original description is different from MNHN record.

##### Family Congridae (83)

**(67) *Ariosomabrachyrhynchus* Fowler, 1934: 269, fig. 30**

= *Parabathymyrusbrachyrhynchus* (Fowler, 1934).

**Holotype.**USNM 92357 (330.0), Albatross station 5256 (07°21.75'N, 124°07.25'E), Utara Point, Bongo Island, Illana Bay, Mindanao, 289 m, 22 May 1908.

**Paratype.**USNM 135121 (1, 268.0), Albatross station 5247, Dumalag Island, Davao Gulf, Mindanao, 247 m, 18 May 1908.

**Remarks.** Radiographs and vertebral counts of the holotype are available in the USNM record.

**(68) *Ariosomaobud* Herre, 1923b: 144, pl. 1 (fig. 2)**

**Holotype.** BSMP (184.0), Marinduque Island.

**Remarks.** The original description did not indicate the collection date. The type specimen was presumed destroyed.

**(69) *Ariosomasazonovi* Karmovskaya, 2004: S7, fig. 4**

**Holotype.**MNHN 1998-0647 (male, 227.0 TL), Musorstrom 2 station 40 (13°08.00'N, 122°39.00'E), south of Luzon, 280–440 m, 25 Nov. 1980.

**Paratypes.**MNHN 1984-0627 (1, 310.0) and MNHN 1998–0646 (1, 395.0 TL), same data as holotype; MNHN 1998-0642 (2, 155.0–230.0), Musorstom 2 station 35, off Calapan Port, between Luzon and Mindoro islands, 160–198 m, 24 Nov. 1980.

**(70) *Bathycongrusbleekeri* Fowler, 1934: 272**

**Holotype.**USNM 92353 (80.0 TL), Albatross station 5257 (07°22.20'N, 124°12.25'E), Utara Point, Bongo Island, Illana Bay, Mindanao, 51 m, 18 Sep. 1909.

**Remarks.** The station published was “d.5557 May 22-08”, but Albatross station 5557 was surveyed on 18 Sep. 1909, while station 5257 was established on 22 May 1908 and regarded as correct (although with some doubt) (Smith 1994). Radiograph and vertebral count are available in the USNM record.

**(71) *Bathycongrusmegalops* Fowler, 1934: 270, fig. 31**

= *Bathycongrusretrotinctus* (Jordan & Snyder, 1901).

**Holotype.**USNM 92345 (330.0 TL), Albatross station 5527 (09°22.50'N, 123°42.67'E), Diuata Point, Balicasag Island, between Siquijor and Bohol islands, 717 m, 11 Aug. 1909.

**Paratype.**USNM 93295 (1, 96.0), Albatross station 5537, Apo Island, off Negros Island, 465 m, 19 Aug. 1909.

**Remarks.** The original description indicated the holotype’s size as 330 mm TL, but different in the USNM record. Vertebral count of the holotype is available in the USNM records.

**(72) Bathycongrus (Microcephalocongrus) stimpsoni Fowler, 1934: 270, fig. 32**

= *Bathycongrusguttulatus* (Günther, 1887).

**Holotype.**USNM 92344 (670.0), Albatross station 5494 (09°06.50'N, 125°18.67'E), between Leyte and Mindanao islands, 1240 m, 2 Aug. 1909.

**Remarks.** A radiograph and vertebral count are available in the USNM record.

**(73) *Bathycongrusvillosus* Smith, Karmovskaya & da Silva, 2020: 92, figs 1–3, 4A, 5A, 6A, 7A, 8A, 9A, 10A, 11A, 12A**

**Holotype.**MNHN 1997-3795.

**Paratypes.**USNM 344104 (2 females, 239.0–296.0) and USNM 451061 (1 male, 252.0), Albay Gulf, Luzon, 174–190 m, 22 Sep. 1995.

**Remarks.** Based on the USNM record, paratype 344104 originally had 3 specimens but 1 was donated to the University of Sao Paulo (MZUSP). USNM 451061 is stained and partly dissected based on the USNM record.

**(74) *Bathyurocongerfowleri* Smith, Ho & Tashiro, 2018: 158, figs 7, 8A**

**Holotype.**USNM 93376 (298.0), Albatross station 5326, Hermanos Island, off northern Luzon, 421 m, 12 Nov. 1908.

**Paratypes.**USNM 441776 [ex USNM 93376] (2, 145.0–155.0), same data as holotype; USNM 93379 (3, 135.0–186.0), Albatross station 5325, Hermanos Island, off northern Luzon, 410 m, 12 Nov. 1908; USNM 93377 (1, 275.0+), Albatross station 5329, Font Island, off northern Luzon, 388 m, 19 Nov. 1908.

**Remarks.** The type specimens were previously mixed, but the two smallest specimens were moved to USNM 441776.

**(75) *Chilocongerphilippinensis* Smith & Karmovskaya, 2003: 9, figs 7–9**

**Holotype.**MNHN 1998-0664 (male, 166.0 TL), Musorstom 2 station 59 (14°00.00'N, 120°16.00'E), off Lubang Island, west of Luzon, 186–190 m, 28 Nov. 1980.

**Paratypes.**MNHN 2002-3730 (4, 161.0–183.0), same data as holotype; MNHN 1998-0666 (1 female, 190.0), Musorstom 2 station 63, same locality as holotype, 215–230 m, 29 Nov. 1980.

**(76) *Congermacrocephalus* Kanazawa, 1958: 254, pl. 1 (fig. M)**

**Holotype.**USNM 164334 (803.0 TL), Albatross station 5367 (13°34.62'N, 121°07.50'E), Malabrigo Lighthouse, Verde Island Passage, Luzon, 329 m, 22 Feb. 1909.

**Remarks.** Dry osteological and radiographs are available in the USNM record. Antiorbitals removed, stained, and dried.

**(77) *Congerphilippinus* Kanazawa, 1958: 255, pl. 1 (fig. K)**

**Holotype.**USNM 134969 (234.0 TL), Cebu Market, Cebu Island, 22 Mar. 1909.

**Paratypes.**CAS-SU 26848 (2, 212.0–278.0), Dumaguete, Negros Island, 18 Jun. 1931; CAS-SU 27116 (1, 223.0), Cebu Island, 27 Aug. 1931.

**Remarks.** Dry osteological and radiographs are available in the USNM record. Antiorbitals were removed, stained, and dried.

**(78) *Congrhynchustalabonoides* Fowler, 1934: 273, fig. 33**

**Holotype.**USNM 92350 (292.0 TL), Albatross station 5502 (08°37.62'N, 124°35.0'E), Macabalan Point, northern Mindanao, 391 m, 4 Aug. 1909.

**Paratypes.**USNM 93347 (1, 300.0), Albatross station 5216, Anima Sola Island, between Burias and Luzon islands, 391 m, 22 Apr. 1908; USNM 93348 (1, 118.0), Albatross station 5247, Dumalag Island, Davao Gulf, Mindanao, 247 m, 18 May 1908.

**Remarks.** Photographs, radiographs, and vertebral count of the holotype are available in the USNM record.

**(79) *Gnathophisasanoi* Karmovskaya, 2004: S18, fig. 10**

**Holotype.**MNHN 1998-0645 (male, 266.0 TL), Musorstom 2 station 40 (13°08.00'N, 122°39.00'E), Sibuyan Sea, south of Luzon, 280–440 m, 25 Nov. 1980.

**Paratype.**MNHN 1998-0650 (1, 350.0 TL), Musorstom 2 station 41, same locality as holotype, 166–172 m, 25 Nov. 1980.

**(80) *Gorgasiapreclara* Böhlke & Randall, 1981: 379, figs 1E, 4C, 5 (right), 8**

**Holotype.** BPBM 21012 (299.0 TL), Sumilon Island, Cebu, 24–29 m, 26 Aug. 1977.

**Paratypes.**ANSP 142713 [ex BPBM 22135] (1, 280.0), ANSP 142710 [ex BPBM 22135] (1, 263.0), ANSP 142732 [ex BPBM 21015] (1, 323.0), BPBM 21013 (1, 287.0), BPBM 22135 (4, 275.0–331.5), CAS 45890 (1, 219.5), MNHN 1980-1191 (1, 323.0) and USNM 221381 (1, 328.5), taken with holotype; ANSP 142712 [ex BPBM 21015] (1, 236.5) and BPBM 21015 (2, 229–265.5), Caban Island (near Maricaban Island), Luzon, 30 m, 2 Sep. 1977; ANSP 142714 (1, 244.0), southwest of Caban Island, Batangas, Luzon, 30 m, 28 Jul. 1978.

**(81) *Heterocongerperissodon* Böhlke & Randall, 1981: 377, figs 1D, 4B, 5 (lower left), 7**

**Holotype.** BPBM 21017 (537.0 TL), off Dumaguete pier, Negros Island 10 m, 30 Aug. 1977.

**Paratype.**ANSP 136746 [ex BPBM 21017] (1), taken with holotype.

**Remarks.** Anterior part of the paratype only based on ANSP’s record.

**(82) Leptocephalus (Diaphanichthys) brevicaudus Peters, 1864: 399**

**Syntypes.** ZMB 5405 (7), between Masbate and Luzon islands.

**Remarks.**Peters (1865b) also mentioned it as a new species. Unknown status in Fricke et al. (2024). Further study is needed to confirm its status. The original description did not indicate the collection date.

**(83) *Leptocephalusindicus* Weber, 1913: 74, fig. 22**

**Holotype.** ZMA 112.602 (115.0), Siboga station 101 (06°15.00'N, 120°21.00'E), Sulu Sea, 1270 m.

**Remarks.** Unknown status in Fricke et al. (2024). The original description did not indicate the collection date. Nijssen et al. (1993) indicated that the sample collected in Indonesia differed from the original description. They also mentioned many errors in type information due to incomplete curatorial administration from 1883 to 1949.

**(84) *Parabathymyrusphilippinensis* Ho, Smith & Shao, 2015: 132, figs 1**

**Holotype.** ASIZP 68112 (398.0), 14°32.40'N, 121°42.00'E, off Aurora, Luzon, 233–249 m, 29 May 2007.

**Paratype.** ASIZP 68117 (1, 341.0), off Aurora, Luzon, 335–356 m, 28 May 2007.

**(85) *Rostrocongermacrouriceps* Smith, 2018: 79, figs 1, 2**

**Holotype.** ASIZP 68072 (241 TL), 15°11.07'N, 121°34.72'E, Aurora, Luzon, 244–296 m, 28 May 2007.

**(86) *Silvesterinaparvibranchialis* Fowler, 1934: 275, fig. 35**

= *Bathyurocongerparvibranchialis* (Fowler, 1934).

**Holotype.**USNM 92346. Buton Sea, Indonesia.

**Paratypes.**USNM 93371 (1, 585.0), Albatross station 5202, Limasaua Island, Sogod Bay, Southern Leyte, 918 m, 10 Apr. 1908; USNM 93373 (1, 610.0), Albatross station 5527, Balicasag Island, between Siquijor and Bohol islands, 717 m, 11 Aug. 1909; USNM 93374 (1, 598.0) Albatross station 5513, and USNM 93375 (1, 512.0), Albatross station 5511, Camp Overton Lighthouse, northern Mindanao, 750–924 m, 7 Aug. 1909; USNM 93378 (1, 290.0), Albatross station 5111, Sombrero Island, off southern Luzon, 432 m, 16 Jan. 1908; USNM 93376 (3, 145.0–298.0) and USNM 93379 (3, 135.0–186.0), Albatross station 5326, Hermanos Island, off northern Luzon, 410–420 m, 12 Nov. 1908; USNM 93377 (1, 275.0), Albatross station 5329, Font Island, off northern Luzon, 388 m, 19 Nov. 1908.

**Remarks.** The original description mentioned Buton Strait, Philippines as the holotype locality but Buton Strait is part of Indonesia. It also mentioned ten paratypes but could meant ten lots of paratypes instead of ten specimens (Smith 1994).

**(87) *Taeniocongerchapmani* Herre, 1923b: 152, pl. 3**

= *Heterocongerchapmani* (Herre, 1923).

**Holotype.** BSMP (690.0), Dumaguete, Negros Island, ca. 1914.

**Remarks.** No catalog number was mentioned in the original description. The type specimen was presumed destroyed.

**(88) *Taeniocongernaeocepaeus* Böhlke, 1951: 32, fig. 1**

= *Gorgasianaeocepaea* (Böhlke, 1951).

**Holotype.**CAS-SU 16052 (304.0 TL), Patalon, Zamboanga, Mindanao, Aug. 1940.

**Paratype.**CAS-SU 16051 (1, 285.0 TL), same data as holotype.

**Remarks.** A photograph and radiographs are available in the CAS record.

##### Family Moringuidae (84)

**(89) *Moringuacagayana* Seale, 1910: 493**

= *Moringuaguthriana* (McClelland 1844).

**Holotype.** BSMP 1621 (female, 616.0), Cagayan, Mindanao, 13 Sep. 1907.

**Remarks.** The type specimen was lost (Kottelat 2013).

**(90) *Moringuapenni* Schultz in Schultz et al., 1953: 96, fig. 20b**

**Holotype.**USNM 130660. Milne Bay, Papua New Guinea.

**Paratype.**USNM 52040 (1, 284.0), southern Negros Island, 1901.

**(91) *Moringuarobusta* Herre, 1923b: 185, pl. 7**

= *Moringuaraitaborua* (Hamilton, 1822).

**Holotype.** BSMP 9664 (655.0), Dumaguete, Negros Island.

**Remarks.** The original description did not indicate the collection date. The type specimen was lost (Kottelat 2013).

##### Family Anguillidae (91)

**(92) *Anguillahuangi* Teng, Lin & Tzeng, 2009: 812, figs 1, 2**

= *Anguillaluzonensis* Watanabe, Aoyama & Tsukamoto, 2009.

**Holotype.** ASIZP 0069360 (1000.0 TL), Cagayan River, Luzon.

**Paratypes.** ASIZP 0069361-9 (9, 219.0–507.7 TL), same as holotype.

**Remarks.** Publication of *Anguillaluzonensis* (Mar. 2009) predates *Anguillahuangi* (Nov. 2009). The original description did not indicate the collection date.

**(93) *Anguillaluzonensis* Watanabe, Aoyama & Tsukamoto, 2009: 389, fig. 2**

**Holotype.** NSMT-P 90000 (528.0 TL), upper reaches of the Pinacanauan River near Saua, northern Luzon, 21 Jan. 2009.

**Paratypes.** NSMT-P 90001 (1, 682.0 TL), Lagum area, upper reaches of the two main branches of the Pinacanauan River, northern Luzon, 29 Jan. 2008; NSMT-P 90002 (1, 370.0 TL), same locality as holotype, 16 Dec. 2008; NSMT-P 90004–90028, (25, 244.0–674.0 TL), same locality as holotype, 21–22 Jan. 2009; NSMT-P 90003 (583.0 TL), upper reaches of the Pinacanauan River, near Lamiga, northern Luzon, 28 Dec. 2008.

**(94) *Muraenamanillensis* Bleeker, 1864: 31**

= *Anguillamarmorata* Quoy & Gaimard, 1824.

**Holotype.**MNHN 0000-0803 (660.0), Manila, Luzon, 1861.

#### ﻿﻿ORDER CLUPEIFORMES (29)

##### Family Engraulidae (100)

**(95) *Stolephorusbabarani* Hata, Lavoué & Motomura, 2020: 510, figs 1–3**

**Holotype.** KAUM-I 62918 (75.6), off Iloilo, Panay Island, 26 Jul. 2014.

**Paratypes.** KAUM-I. 62920 (1, 57.4); KAUM-I. 62921 (1, 55.3 SL), taken with holotype; KAUM-I. 63064 (1, 58.1), 1 Aug. 2014; KAUM-I. 91758 (1, 78.5), KAUM-I. 91759 (1, 76.3); KAUM-I. 91760 (1. 76.6); KAUM-I. 91761 (1, 80.8); KAUM-I. 91762 (1, 77.1); KAUM-I. 91877 (1, 76.6); KAUM-I. 91884 (1, 80.5); KAUM-I. 91886 (1, 75.9); KAUM-I. 91888 (1, 77.2); KAUM-I. 91892 (1, 77.9); KAUM-I. 91894 (1, 79.3); KAUM-I. 91896 (1, 74.8); KAUM-I. 91901 (1, 81.1); KAUM-I. 91909 (1, 78.7); KAUM-I. 91910 (1, 74.7); KAUM-I. 91913 (1, 78.5); KAUM-I. 91917 (1, 79.8); KAUM-I. 91919 (1, 77.3); KAUM-I. 91922 (1, 78.6); KAUM-I. 91925 (1, 75.1); UPVMI 2676 (1, 78.1); UPVMI 2677 (1, 76.5), 10 Sep. 2016; all specimens were taken off Iloilo, Panay Island.

**Remarks.** All type specimens were purchased at Iloilo Central Market, Panay Island.

**(96) *Stolephorusinsignus* Hata & Motomura, 2018: 284, fig. 3**

**Holotype.** KAUM-I 80742 (73.4), off Oton, Panay Island, 11 Nov. 2015.

**Paratypes.**USNM 138518 (5, 60.4–65.4), Cavite, Manila Bay, Luzon; USNM 444945 [ex USNM 138527] (1, 51.8), Manila Bay, Luzon; USNM 444946 [ex USNM 138523] (1, 67.5), off Iloilo, Panay Island.

**Remarks.** All type specimens were bought from fish markets.

**(97) *Stolephorusmercurius* Hata, Lavoue & Motomura, 2021: 32, fig. 21**

**Holotype.** KAUM-I 80755 (84.7), off Oton, Panay Island (purchased at Oton Fish Market).

**Paratypes.** KAUM-I 80755 (1, 84.7), KAUM-I 80770 (1, 76.1) SL, taken with the holotype; KAUM-I 50950 (1, 86.9), KAUM-I 80654 (1, 67.7), UPVMI 177 (1, 79.9), off Iloilo, Panay (purchased at Iloilo Central Market); KAUM-I 80619 (1, 79.6), off Oton, Panay (purchased at Oton Fish Market); UPVMI 59 (1, 85.3), UPVMI 61 (1, 77.8), off Miagao, Panay (purchased at Miagao Fish Market); USNM 138534 (1, 73.2), Pucot River, Manila Bay.

**Remarks.** The original description did not indicate the collection date.

**(98) *Stolephorusoligobranchus* Wongratana, 1983: 397, fig. 15**

= *Encrasicholinaoligobranchus* (Wongratana, 1983).

**Holotype.**BMNH 1979.12.5.3 (57.0), Rosario, Cavite, Manila Bay, Luzon, 21 Jun. 1958.

**Paratypes.**BMNH 1979.12.5.4–5 (2, 53.1–62.0), same data as holotype.

**Other catalog numbers.** holotype (NHMUK:ecatalogue:2545315), paratypes (NHMUK:ecatalogue:2545316).

**Remarks.** The collection date was not indicated in the original description.

**(99) *Stolephorusronquilloi* Wongratana, 1983: 399, fig. 17**

**Holotype.**BMNH 1969.5.30.88 (48.7), Manila Bay, Luzon.

**Paratypes.**BMNH 1960.4.7.103–115 (13, 43.0–54.0) and BMNH 1969.5.30.79–87 (9, 45.0–48.5), Manila Bay, Luzon; BMNH 1966.1.17.126–134 (9, 33.0–47.5), Cavite, Luzon; BMNH 1969.4.22.1620–1627 (8, 47.0–50.0), Mindanao.

**Other catalog numbers.** holotype (NHMUK:ecatalogue:2650722), paratypes BMNH 1960.4.7.103–115 (NHMUK:ecatalogue:2525132), BMNH 1969.5.30.79–87 (NHMUK:ecatalogue:2533301), BMNH 1966.1.17.126–134 (NHMUK:ecatalogue:2530199) and BMNH 1969.4.22.1620–1627 (NHMUK:ecatalogue:2533042).

**Remarks.** The original description or NMH record did not indicate the collection dates. There is a discrepancy on the number of specimens mentioned in the original description compared to NHM’s record: 14 specimens were mentioned for BMNH 1960.4.7.103–115, but only 13 in NHM’s record; 21 specimens for BMNH 1969.5.30.79–87, but only 9 in NHM’s record; 8 specimens for BMNH 1966.1.17.126–134, but 9 in NHM’s record; 9 specimens for BMNH 1969.4.22.1620, but 9 in NHM’s record.

##### Family Clupeidae (102)

**(100) *Clupeamanulensis* Marion de Procé, 1822: 132**

Manila Bay, Luzon.

**Remarks.** No type known. The name has been overlooked and its status needs further study.

##### Family Dorosomatidae

**(101) *Harengulatawilis* Herre, 1927b: 273, pl. 1**

= *Sardinellatawilis* (Herre, 1927).

**Holotype.** BSMP 13198, Taal Lake, Luzon.

**Paratypes.** BSMP (77, 78.0–128.0), same data as holotype.

**Remarks.** The collection date was not indicated in the original description. All type specimens were presumed destroyed.

**(102) *Sardinellagoni* Stern, Rinkevich & Goren, 2016: 16, figs 2D, 4D**

**Holotype.**PNM 15191 (133.2), Boracay Island, Aklan, 16 Aug. 2014.

**Paratypes.** SMNHTAU P.15447 (6, 129.8–144.1), same data as holotype.

**(103) *Sardinellapacifica* Hata & Motomura, 2019: 76, figs 1, 2**

**Holotype.**BMNH 1985.4.12.1 (105.1), Manila Harbor, Manila Bay, Luzon.

**Paratypes.**BMNH 1985.4.12.2 (1, 98.7), same data as holotype; BMNH 1960.4.7.52 (1, 90.2), Palawan Island; CAS 38365 (1, 105.9), CAS 51909 (1, 96.5), CAS 52501 (1, 98.4), CAS-SU 28569 (1, 101.3), CAS-SU 29920 (2, 97.6–103.3), CAS-SU 32915 (2, 95.7–97.8) and KAUM–I. 125000 (1, 95.9), all from Manila Bay, Luzon; CAS 59712 (1, 100.3), USNM 56232 (1, 94.5) and USNM 56233 (1, 92.2), Bacon, Sorsogon, Luzon; CAS-SU 28568 (1, 96.5), Alabat Island; Bacon, Sorsogon, Luzon; USNM 72197 (1, 92.9), Manila, Luzon; USNM 177667 (2, 93.4–96.7); USNM 403460 (1, 95.9), Navotas, Manila, Luzon; USNM 427789 (1, 94.9), Catbalogan, Samar Island.

**Other catalog numbers.** holotype (NHMUK:ecatalogue:2555320) and paratype BMNH 1985.4.12.2 (NHMUK:ecatalogue:8844391).

**Remarks.** Most of the type specimens were previously identified as *Sardinellafimbriata*. A photograph is available in the USNM record. The collection date was not indicated in the original description. BMNH 1960.4.7.52 was not found in NHM’s record.

##### Family Dussumieriidae

**(104) *Etrumeusalbulina* Fowler, 1934: 244, fig. 7**

= *Dussumieriaalbulina* (Fowler, 1934).

**Holotype.**USNM 93136 (116.9), Iloilo Market, Panay Island, 1 Jun. 1908.

**Paratypes.**USNM 120854 (6), same data as holotype.

**Remarks.** The paratypes were mixed with 15 additional specimens, with the ledger record stated 21 specimens originally in the jar. The original description did not clearly specify the paratypes but mentioned tag numbers as paratypes but no sizes were provided.

#### ﻿﻿ORDER ALEPOCEPHALIFORMES (30)

##### Family Alepocephalidae (105)

**(105) *Alepocephalusandersoni* Fowler, 1934: 246, fig. 8**

**Holotype.**USNM 92329 (253.0), Albatross station 5527, Balicasag Island, between Siquijor and Bohol islands, 717 m, 11 Aug. 1909.

**Paratype.**USNM 93624 (1), Albatross station 5123, Malabrigo Lighthouse, east of Mindoro Island, 518 m, 2 Feb. 1908.

**(106) *Bathytroctesharperi* Fowler, 1934: 250, fig. 12**

= *Rouleinaattrita* (Vaillant 1888).

**Holotype.**USNM 92333. Gulf of Boni, Sulawesi, Indonesia.

**Paratype.**USNM 93382 (1), Albatross station 5463, Sialat Point Lighthouse, San Bernardino Strait, east of Luzon, 549 m, 16 Jun. 1909.

**(107) *Bathytrocteshataii* Fowler, 1934: 249, fig. 11**

**Holotype.**USNM 92331 (172.0), Albatross station 5463 (13°40.95'N, 123°57.75'E), Sialat Point Lighthouse, San Bernardino Strait, east of Luzon, 549 m, 16 Jun. 1909.

**Paratypes.**USNM 93389–90 (2,1), all taken with the holotype.

**Remarks.** Unknown status in Fricke et al. (2024). A further study is needed to verify its status.

**(108) *Leptodermaretropinna* Fowler, 1943: 55, fig. 5**

**Holotype.**USNM 99512 (147.0), Albatross station 5495 (09°06.50'N, 125°00.33'E), off Diuata Point, between Leyte and Mindanao islands, 1785 m, 2 Aug. 1909.

**Remarks.** A radiograph is available in the USNM record.

**(109) *Narcetesgarmani* Fowler, 1934: 255, fig. 17**

**Holotype.**USNM 92337 (158.0), Albatross station 5282, South China Sea, southern Luzon, 454 m, 18 Jul. 1908.

**Remarks.** Unknown status in Fricke et al. (2024). A further study is needed to verify its status. A radiograph is available in the USNM record.

**(110) *Narceteslloydi* Fowler, 1934: 253, fig. 16**

**Holotype.**USNM 92335 (>470.0 TL), Albatross station 5460 (13°32.50'N, 123°58.10'E), Sialat Point Lighthouse, Lagonoy Gulf, east of Luzon, 1033 m, 10 Jun. 1909.

**(111) *Rouleinadanae* Parr, 1951: 14**

**Holotype.** ZMUC P1778 (108.0, without caudal), Dana station 3686 (08°34.00'N, 119°55.00'E), Sulu Sea, near Tubbataha Reef Natural Park, Palawan, 2000 m, 6 Apr. 1929.

**(112) *Rouleinaeuryops* Sazonov, 1999: 438 [480], fig. 1**

**Holotype.**USNM 352405 [ex USNM 137725] (171.0), Albatross station 5511 (08°15.00'N, 123°57.00'E), Camp Overton Lighthouse, northern Mindanao, 750 m, 7 Aug. 1909.

**Paratypes.**USNM 137725 (14, 106.0–173.5), USNM 137737 (1, 179.0), USNM 137738 (3, 104.5–177.0) and USNM 137740 (1, 141.0), same locality as the holotype, 750–924 m, 7 Aug. 1909; USNM 137730 (2, 179.0–182.0), Albatross station 5528, and USNM 137731 (1, 127.0), Albatross station 5533, Balicasag Island, between Siquijor and Bohol islands, 803 m, 11 and 19 Aug. 1909; USNM 137734 (1, 224.5), Albatross station 55487, and USNM 137735 (4, 186.0–194.0), Albatross station 5448, San Ricardo Point, Panaon Island, between Leyte and Mindanao islands, 1339–1412 m, 31 Jul. 1909; USNM 137736 (10, 71.0–234.0), Albatross station 5201, Limasaua Island, Sogod Bay, Southern Leyte, 1013 m, 10 Apr. 1908.

**Remarks.**USNM 137736 originally contained 24 specimens, but 10 were re-cataloged as USNM 359855, and 4 were removed to USNM 326308.

**(113) *Xenodermichthysfunebris* Fowler, 1943: 54, fig. 4**

= *Rouleinasquamilatera* (Alcock, 1898).

**Holotype.**USNM 99534 (188.0 to end of broken caudal), Albatross station 5348 (10°57.75'N, 118°38.25'E), off Tabonan Point, Palawan Passage, 686 m, 27 Dec. 1908.

#### ﻿﻿ORDER CYPRINIFORMES (32)

##### Family Cyprinidae (109)

**(114) *Barbodesamara* Herre, 1924a: 295**

= *Barbodesamarus* Herre, 1924.

**Holotype.** BSMP 9167, Dansalan, Lake Lanao, Mindanao, 11 Sep. 1922.

**Paratypes.** BSMP (14, 66.0–92.0).

**Remarks.** All type specimens were lost (Kottelat 2013).

**(115) *Barbodesbaoulan* Herre, 1926c: 499, pl. 1**

**Syntypes.** BSMP (8) and CAS-SU 24469 (1) (88.0–108.0), Lake Lanao, Mindanao, 9 Apr 1925.

**Remarks.** All BSMP specimens were lost (Kottelat 2013). A photograph and radiographs are available in the CAS record.

**(116) *Barbodesclemensi* Herre, 1924a: 293**

**Holotype.** BSMP 10159 (188.0), Dansalan, Lake Lanao, Mindanao, May 1921.

**Paratypes.** BSMP (3, 116.0–137.0); BSMP (5, 114.0–190.0), Lake Lanao, Jun. 1907.

**Remarks.** All type specimens were lost (Kottelat 2013).

**(117) *Barbodesdisa* Herre, 1932: 140**

**Holotype.**CAS-SU 27713 (82.0), Dansalan Market, Lanao, Mindanao, 9 Jul. 1931.

**Paratypes.** BSMP (81.0), same data as holotype; USNM 148143 [ex CAS-SU 27713] (1), Lake Lanao, Mindanao, 9 Jul. 1931.

**Remarks.** BSMP paratype was presumed destroyed. A photograph and radiographs of the holotype are available in CAS’s record.

**(118) *Barbodesflavifuscus* Herre, 1924a: 296**

**Holotype.** BSMP 9164 (105.0), Lumbatan, Lake Lanao, Mindanao.

**Paratypes.** BSMP (2, 89.0 each), same data as holotype.

**Remarks.**Kottelat (2013) did not provide information on the type status, but it is presumed that all type specimens were destroyed. The collection date was not indicated in the original description.

**(119) *Barbodeshemictenus* Jordan & Richardson, 1908: 241, fig. 5**

**Holotype.**CAS-SU 20213, Mindoro Island.

**Paratypes.**CAS-SU 20443 (3) and USNM 61685 (1), 1906, same locality with the holotype.

**Remarks.** The original description mentioned specimen sizes (76.2–101.6) but did not specify which type specimen they belong to, including the collection date. A photograph and radiographs of the holotype are available in CAS’s record.

**(120) *Barbodeskatolo* Herre, 1924a: 301**

**Holotype.** BSMP 9161 (110.0), Dansalan, Lake Lanao, Mindanao.

**Paratypes.** BSMP (2, 95.0–101.0), same data as holotype, 5 May 1921.

**Remarks.** All type specimens were lost (Kottelat 2013).

**(121) *Barbodeslanaoensis* Herre, 1924a: 300**

**Syntypes.** BSMP (8, 78.0–95.0), Dansalan, Lake Lanao, Mindanao, 5 May 1921.

**Remarks.** All type specimens were lost (Kottelat 2013).

**(122) *Barbodeslindog* Herre, 1924a: 304**

**Syntypes.** BSMP; CAS-SU 27714 (2) and CAS-SU 24466 (3) (100.0–107.0), Dansalan, Lake Lanao, Mindanao, 4 May 1921.

**Remarks.** BSMP specimen was lost (Kottelat 2013). A photograph and a radiograph of CAS-SU 27714 are available in the CAS record.

**(123) *Barbodesmanalak* Herre, 1924a:302**

**Holotype.** BSMP 9998 (240.0), Dansalan, Lake Lanao, Mindanao, May 1921.

**Paratypes.** BSMP (3, 182.0–236.0), same data as holotype.

**Remarks.** All type specimens were lost (Kottelat 2013).

**(124) *Barbodespalata* Herre, 1924a: 305**

**Syntypes.** BSMP (5 males + 5 females, 97.0–112.0), Dansalan, Lake Lanao, Mindanao, 5 May 1921.

**Remarks.** All type specimens were lost (Kottelat 2013).

**(125) *Barbodespyrpholeos* Tan &Husana, 2021: 310, figs 1–8**

**Holotype.**PNM 15649 (104.1), Ugnop Cave system, Agusan drainage, Mindanao, 14 May 2014.

**Paratypes.**PNM 15650 [2 ex ZRC 61230] (3, 69.6–93.1) and ZRC 61232 (2, 59.7–76.1) same data as holotype; PNM 15651 [ex ZRC 61231] (3, 68.2–103.4), same locality as holotype, 1 May 2012.

**(126) *Barbodessirang* Herre, 1932: 140**

**Holotype.**CAS-SU 69047 (59.0), Lumbatan, south of Lake Lanao, Mindanao, 2 Jul. 1931.

**Paratypes.**CAS-SU 27715 (39, 40.0–61.0) and USNM 148144 (1), same data as holotype.

**Remarks.**CAS-SU paratypes are a mix of types and non-types and cannot easily be isolated. Two specimens are separated in a vial within the bottle, and one was assumed to be the holotype (Böhlke 1953). A photograph and a radiograph of the holotype are available in CAS’s record.

**(127) *Barbodestras* Herre, 1926c: 501, pl. 2**

**Holotype.** BSMP (126.0), Datu Samba-an, Camp Keithley, Lanao, Mindanao, 9 Apr. 1925.

**Remarks.** The type specimen was lost (Kottelat 2013).

**(128) *Barbodestumba* Herre, 1924a: 285**

**Syntypes.** BSMP (39), Siwagat River and Lake Nunuñgan, outlet of Lake Dapao, Lanao, Mindanao; CAS-SU 24471 (2), Lake Uyaan, highlands of Lanao, Mindanao, 13 Sep. 1922.

**Remarks.** BSMP specimens were lost (Kottelat 2013).

**(129) *Barbusbantolanensis* Day, 1914: 188, pl. 1 (figs 1, 2)**

= *Barbodesmanguaoensis* (Day, 1914).

**Holotype.**CAS-SU 29823 (118.0 BL), Lake Manguao, Taytay, Palawan Island, May 1913.

**Paratypes.** FMNH 47122 (12) and CAS-SU 69674 (5), same data as holotype.

**Remarks.** The original description mentioned measurements of six specimens only (89.0–115.0 BL). The first reviser (Cervancia and Kottelat 2007) gave precedence to *B.manguaoensis*). A photograph and a radiograph of the holotype are available in CAS’s record.

**(130) *Barbuscataractae* Fowler, 1934: 280, fig. 40**

= *Barbodescataractae* (Fowler, 1934).

**Holotype.**USNM 93137 (135.0 TL), Cascade River, Murcielagos Bay, Mindanao, 20 Aug. 1909.

**Paratypes.**USNM 93415 (26), same data as holotype.

**(131) *Barbusherrei* Fowler, 1934: 280, fig. 41**

= *Barbodesherrei* (Fowler, 1934).

**Holotype.**USNM 93138 (110.0 TL), Vicar market, Lake Lanao, Mindanao, 23 May 1908.

**Paratypes.**CAS-SU 14971 [ex USNM 93408] (1) and USNM 93408 (3), same data as holotype.

**Remarks.** Radiographs are available in the USNM record.

**(132) *Barbusivis* Seale, 1910: 494, pl. 1**

= *Barbodesivis* (Seale, 1910).

**Holotype.** BSMP 5233 (130.0), a small stream near Balabac Island, Palawan, 11 Aug. 1908.

**Paratypes.** BSMP (89).

**Remarks.** All type specimens were lost (Kottelat 2013).

**(133) *Barbusmanguaoensis* Day, 1914: 189, pl. 1 (fig. 3)**

= *Barbodesmanguaoensis* (Day, 1914).

**Holotype.** [Specimen No. 15] (89.0 BL), Lake Manguao, Taytay, Palawan Island.

**Paratypes.** Uncat. (6, 52.0–132.0 BL), same data as the holotype.

**Remarks.** No catalog numbers were provided for the paratypes during the joint expedition of the Bureau of Science and the University of the Philippines. I suspect that the type specimens might have been deposited in the BSM but destroyed during World War II. The collection date was also not indicated in the original description.

**(134) *Barbuspalavanensis* Boulenger, 1895: 186**

= *Barbodespalavanensis* (Boulenger, 1895).

**Syntypes.**BMNH 1894.6.30.188–190 (3), Palawan Island.

**Other catalog number.** NHMUK:ecatalogue:3119305.

**Remarks.** The original description mentioned that one specimen measured 160.0 TL but did not indicate the collection date of the syntypes.

**(135) *Barbusquinquemaculatus* Seale & Bean, 1907: 229, fig. 1**

= *Barbodesquinquemaculatus* (Seale & Bean, 1907).

**Holotype.**USNM 57840 (88.9), Zamboanga, Mindanao.

**Remarks.** The original description mentioned that there are numerous paratypes, but no further information was given. Three lots (USNM 61155–57) with 78 specimens were found in the USNM record with the same collector and locality with the holotype. It is unclear whether these are the paratypes mentioned in the original description. The original description did not indicate the collection date.

**(136) *Cephalakompsuspachycheilus* Herre, 1924a: 276, pl. 2 (fig. 2)**

= *Barbodespachycheilus* (Herre, 1924).

**Holotype.** BSMP (112.0), Dansalan, Lake Lanao, Mindanao, 5 May 1921.

**Remarks.** The type specimen was lost (Kottelat 2013).

**(137) *Cyclocheilichthysschoppeae* Cervancia & Kottelat, 2007: 141, fig. 1**

**Holotype.**PNM (100.3) Iraan River, a tributary of Barbacan River, between Iraan and Dumarao villages, Roxas, Palawan Island, 18 Nov. 2004.

**Paratypes.** WPU-PPC (2, 79.1–82.5), Abongan River, SE of Abongan village, Taytay, 16 Nov. 2004; WPU-PPC (2, 61.1–64.2), Barbacan River, west of Dumarao village, Roxas, 18 Nov. 2004; WPU-PPC (9, 39.2–94.8) and CMK 18533 (3, 96.1–103.7), same locality as precedence, 27–29 Jan. 2005; all taken from Palawan Island.

**Remarks.** The original description only mentioned the institution code, but no catalog numbers were provided (except for CMK specimens). The WPU-PPC Collection Room is undergoing renovation, and no further information was provided by the staff in charge if the type specimens were indeed deposit there.

**(138) *Cyrenecyanopareja* Heckel, 1843: 1025 [35**]

= *Osteochilusvittatus* (Valenciennes, 1842).

**Holotype.** NMW 10814 (127.0), Philippines.

**Remarks.** The original description did not provide a specific locality and the published type locality is doubtful according to Kottelat (2013). The original description did not indicate the collection date.

**(139) *Cyrenephilippinia* Heckel, 1843: 1025 [35**]

**Holotype.** NMW 52774 (152.4), Philippines.

**Remarks.** Questionably a synonym of *Labiobarbusleptocheilus* (Valenciennes, 1842) (Roberts 1994). The original description did not provide a specific locality but was probably in error (Roberts 1994; Kottelat 2013). Roberts (1994) also mentioned that the holotype in Vienna, Austria was not seen. The original description did not indicate the collection date.

**(140) *Hampalalopezi* Herre, 1924a:275**

**Holotype.** BSMP 9186, Langbuan, Busuanga Island, Palawan.

**Paratypes.** BSMP (3, 55.0–85.0), same data as holotype.

**Remarks.** The original description did not indicate the collection date. All type specimens were lost (Kottelat 2013).

**(141) *Mandibularcaresinus* Herre, 1924a: 273**

= *Barbodesresinus* (Herre, 1924).

**Syntypes.** BSMP (3), Agus River, Lake Lanao, Mindanao.

**Remarks.** Misspelled as *resimus* in Kottelat (2013). The original description did not indicate the collection date. All type specimens were lost (Kottelat 2013).

**(142) *Ospatuluspalaemophagus* Herre, 1924a: 279**

= *Barbodespalaemophagus* (Herre, 1924).

**Holotype.** BSMP 9200 (male, 105.0), Lumbatan, Lake Lanao, Mindanao.

**Remarks.** The original description did not indicate the collection date. The type specimen was lost (Kottelat 2013).

**(143) *Ospatulustruncatulus* Herre, 1924a: 278, pl. 2 (fig. 1)**

= *Barbodestruncatulus* (Herre, 1924).

**Holotype.** BSMP 9190, Dansalan, Lake Lanao, Mindanao, May 1921.

**Remarks.** The type specimen was lost (Kottelat 2013).

**(144) *Puntiusjoaquinae* Wood, 1968: 415, fig. 3**

= *Barbodesjoaquinae* (Wood, 1968).

**Holotype.** FMNH 73395, small stream from tiny Basak Lake to the Agus River, Saguiaran, Lanao, Mindanao, 17 Jan. 1964.

**Paratypes.** FMNH 73396 (orig. 5, now 1), 17 Jan 1964; FMNH 98318 (1); FMNH 96451 (1), 5 May 1963; FMNH 96452 (2), 1 Mar. 1964; all specimens from Agus River, Lanao, Mindanao.

**(145) *Puntiusmontanoi* Sauvage, 1881: 103**

= *Barbodesmontanoi* (Sauvage, 1881).

**Holotype.**MNHN A-3398 (1, 90.0 TL), Simulao River, a tributary of Agusan, Mindanao, 1881.

**Paratype.**MNHN A-3399 (12), same data as holotype.

**Remarks.** A re-description was provided by Tan and Husana (2021) who mentioned that the types as “syntypes”. MNHN records indicate the above specimens as holotype and paratypes. Photographs are available in the MNHN record.

**(146) *Puntiusumalii* Wood, 1968: 412, fig. 1**

= *Barbodesumalii* (Wood, 1968).

**Holotype.** FMNH 73393, Agus River, Barrio Matampay, Lanao, Mindanao, 16 Aug. 1963.

**Paratypes.** FMNH 73394 (2), 16 Aug. 1963 and FMNH 96456 (3), 23 Dec. 1962, Agus River, Lanao, Mindanao; MNH 96453 (2), 28 Feb. 1964; FMNH 96454 (2), 14 Feb. 1964; FMNH 96455 (3), 5 Nov. 1962; all specimens from Linamon river, Lanao, Mindanao.

##### Family Danionidae

**(147) *Mearnsellaalestes* Seale & Bean, 1907: 231, fig. 2**

= *Nematabramisalestes* (Seale & Bean, 1907).

**Holotype.**USNM 57841 (62.2), Zamboanga, Mindanao.

**Paratype.**USNM 61151 (1, 50.8), same data as holotype.

**Remarks.** The original description did not indicate the collection date.

**(148) *Nematabramisverecundus* Herre, 1924a: 259**

= *Nematabramisalestes* (Seale & Bean, 1907).

**Holotype.** BSMP, Titunod River, Kolambugan, Lanao, Mindanao.

**Remarks.** The original description did not indicate the collection date. The type specimen was lost (Kottelat 2013).

**(149) *Rasboraeveretti* Boulenger, 1895: 187**

**Syntypes.**BMNH 1894.6.30.191–92, Palawan Island.

**Other catalog number.** NHMUK:ecatalogue:3119306.

**Remarks.** The original description did not indicate the collection date but mentioned that one specimen measured 100.0 TL.

**(150) *Rasboraphilippina* Günther, 1880: 54**

**Syntype.**BMNH 1879.5.14.646 (1), River at Pasananca, Zamboanga, Mindanao.

**Other catalog number.** NHMUK:ecatalogue:3108952.

**Remarks.** The original description mentioned that specimens measured 63.5–88.9 but only one was found in the NMH record. It also did not indicate the collection date.

**(151) *Rasborapunctulatus* Seale & Bean, 1907: 232, fig. 3**

= *Rasboraphilippina* Günther, 1880.

**Holotype.**USNM 57842 (76.2), Zamboanga, Mindanao.

**Paratypes.**USNM 61150 (10, 38.1–76.2), same data as holotype.

**Remarks.** The original description or the USNM record did not indicate the collection date of the holotype. Photographs and a radiograph of the holotype are available in the USNM record.

**(152) *Rasborataytayensis* Herre, 1924a: 264**

**Holotype.** Pool in a dry stream between Taytay and Malampaya Sound, Palawan Island.

**Remarks.** Possibly a synonym of *Rasborasemilineata* Weber and de Beafort 1916 (Brittan 1954) but Kottelat (2013) considers it a valid species. The original description did not provide a catalog number and collection date. The species was described based on six spawning females (38.0–50.0) taken from the type locality with another 164 specimens (18.0–45.0) also taken from a creek near Taytay, Palawan Island. However, the current location of these specimens is unknown. Additional specimens from the type locality are needed to determine its taxonomic validity.

#### ﻿﻿ORDER SILURIFORMES (34)

##### Family Siluridae (149)

**(153) *Hitotaytayensis* Herre, 1924d: 703**

= *Pterocryptistaytayensis* (Herre, 1924).

**Holotype.** BSMP 9357 (120.0 CL), small freshwater creek near Taytay, Palawan Island, May 1918.

**Paratypes.** BSMP (17).

**Remarks.**Herre (1924b) published two new species (together with *Penesiluruspalavanensis*) with identical information. The first reviser (Haig 1952) could not determine which of the species has priority and selected *Hito* over *Hitoichthys*. All type specimens were lost (Kottelat 2013).

**(154) *Penesiluruspalavanensis* Herre, 1924d: 704**

= *Pterocryptistaytayensis* (Herre, 1924).

**Holotype.** BSMP (108.0) Lake Manguao, Taytay, Palawan Island, May 1913.

**Remarks.** The holotype was in poor condition (Haig 1952).

##### Family Clariidae (169)

**(155) *Clariasgilli* Smith & Seale, 1906: 74, figs a, b**

= *Clariasnieuhofii* Valenciennes, 1840.

**Holotype.**USNM 55620 (323.9), Rio Grande de Mindanao, Oct. 1903.

**Paratype.**USNM 126628 [ex USBF 1668] (1, 342.9), same data as holotype.

**Remarks.** Radiographs of the holotype are available in the USNM record.

##### Family Ariidae (172)

**(156) *Ariusdispar* Herre, 1926d: 405, pl. 1 (fig. 6)**

**Holotype.** BSMP (235.0), Paco Market, Manila, Luzon.

**Paratypes.** BSMP (4, 200.0–234.0), Laguna de Bay; Pasig River; Quiapo Market; Los Banos, Laguna, Luzon.

**Remarks.** The original description did not indicate the collection date. All type specimens were lost (Kottelat 2013).

**(157) *Ariusmagatensis* Herre, 1926d: 396, pl. 1 (fig. 1)**

= *Plicofollismagatensis* (Herre, 1926).

**Syntypes.** BSMP (6, 190.0–268.0), Magat River, Bagabag, Nueva Vizcaya, Luzon.

**Remarks.** The original description did not indicate the collection date. All type specimens were lost (Kottelat 2013).

**(158) *Ariusmanillensis* Valenciennes in Cuvier and Valenciennes, 1840: 93**

**Holotype.**MNHN (355.6), Manila, Luzon.

**Remarks.** The original description did not indicate the catalog numbers and collection date.

**(159) *Ariusvenosus* Valenciennes in Cuvier and Valenciennes, 1840: 69**

**Syntypes.**MNHN 0000-1205 (1), Manila, Luzon.

**Remarks.** A drawing of a sample from Manila (M. de Mertens) was mentioned in the original description, but no other information (including collection date) was provided.

**(160) *Netumapatriciae* Takahashi, Kimura & Motomura, 2019: 2, [266], figs 1a–b, 2a–b, 3, 5a–b, 6a–b**

**Holotype.** KAUM-I 98403 (female, 200.1), Iloilo, Panay Island, 20 Mar. 2017.

**Paratypes.**CAS-ICH 63624 (2, 127.3–139.3), CAS-SU 29565 (2, 159.6–239.4) and CAS-SU 69118 (2, 134.8–139.3), Manila Bay, Luzon, 8–22 Dec. 1953; CAS-SU 14416 (1 male, 303.4), FRLM 32750 (1 male, 192.1) and CAS-SU 38203 (1 female, 180.2), Iloilo, Panay Island, 4 Aug. 1940.

**(161) *Pimelodusmanillensis* Valenciennes in Cuvier and Valenciennes, 1840: 93**

= *Cephalocassismanillensis* (Valenciennes, 1840).

**Holotype.**MNHN 0000-1209 (139.7), Manila, Luzon.

**Remarks.** The original description or MNHN did not indicate the collection date.

**(162) *Pseudariusphilippinus* Sauvage, 1880: 226**

= *Ariusmanillensis* Valenciennes, 1840.

**Holotype.**MNHN A-2615, 04°49.98'N, 122°00.00'E, Polilio Island, east ofLuzon, 1876.

**Remarks.** The original description mentioned Lake Laglaize as the type locality, but the MNHN record indicates that Laglaize is the collector’s name.

#### ﻿﻿ORDER ARGENTINIFORMES (39)

##### Family Opisthoproctidae (191)

**(163) *Dolichopteryxandriashevi* Parin, Belyanina & Evseenko, 2009: 843, fig. 5**

**Holotype.** ZMMU P-22214 (56.5), Albatross station 4490 (05°58.00'N, 127°44.00'E), southeast of Mindanao.

**Paratypes.** Uncat. (1, 41.4), same locality as holotype; Uncat. (1, 52.4), Albatross station 3721 (07°00.0'N, 126°43.0'E), east of Mindanao; Uncat. (1, 68.5), Albatross station 5040 (14°01.0'N, 130°32.0'E), Philippine Sea.

**Remarks.** The holotype was not mentioned in the materials examined but determined through a footnote and in the caption in figure 5 (p. 844). The original description did not provide catalog numbers and collection dates for three paratypes.

##### Family Microstomatidae (192)

**(164) *Microstomaschmitti* Fowler, 1934: 256, fig. 18**

= *Nanseniaschmitti* (Fowler, 1934).

**Holotype.**USNM 92327 (218.0 TL), Albatross station 5445, Atalaya Point, Batag Island, east of Luzon, 700 m, 3 Jun. 1909.

**Paratype.**USNM 93355 (1), same locality and date as holotype, 563 m.

#### ﻿﻿ORDER AULOPIFORMES

##### Family Synodontidae (205)

**(165) *Saurusdepressus* Marion de Procé, 1822: 131**

Manila, Luzon.

**Remarks.** No type known. Unknown status in Fricke et al. (2024). Collection of specimens and further study is needed to confirm its status. The collection date was not indicated in the original description.

**(166) *Synodusfasciapelvicus* Randall, 2009: 404, figs 2–3**

**Holotype.** BPBM 29790. Tanjung Luar fish market, Lombok, Indonesia.

**Paratypes.**USNM 391165 (1, 65.0) and BPBM 22134 (1, 56.5), Sumilon Island, Cebu, 24–29 m, 26 Aug. 1977.

**(167) *Synoduspacificus* Ho, Chen & Shao, 2016: 135, figs 1A–C, 2A, 3A, C, E, G, I**

**Holotype.** NMMB-P 22438. Kaohsiung, Taiwan.

**Paratype.**MNHN 2005-1492 (1, 108.7), 12°03.0'N, 121°28.98'E, east of Semirara Island, central Philippines, 120–123 m, 4 Jun. 1985.

**(168) *Synodustectus* Cressey, 1981: 39**

**Holotype.**USNM 218935 (175.0), Caduruan Point, Visayan Sea, between Negros and Masbate islands, 0–75 m, 5 Jun. 1978.

**Paratypes.**USNM 218936 (1, 124.0), East of Sicogon Island; USNM 218952 (6, 111.0–126.0), Tanguingui Island, Visayan Sea, between Negros and Masbate islands, 0–70 m, 4–5 Jun. 1978; USNM 136223 (1, 83.0), Albatross station 5098, off Corregidor Lighthouse, west of Luzon, 70 m, 2 Jan. 1908; USNM 217766 (1, 119.0) and USNM 217767 (1, 93.0), Manila fish market, 13 May 1969.

**Remarks.** Radiographs of the holotype are available in the USNM record.

##### Family Paraulopidae (208)

**(169) *Chlorophtalmusbrevirostris* Fourmanoir, 1981: 87, fig. 2**

= *Paraulopusbrevirostris* (Fourmanoir, 1981).

**Holotype.**MNHN 1979-0433 (130.0), Musorstom 1 station 64 (14°00.06'N, 120°16.14'E), Philippines, 194–195 m, 27 Mar. 1976.

**Remarks.** The original description did not indicate a specific locality.

##### Family Paralepididae (218)

**(170) *Paralepisphilippinus* Fowler, 1934: 281, fig. 42**

= *Lestrolepisjaponica* (Tanaka, 1908).

**Holotype.**USNM 92323 (122.0 TL), Varadero Bay, Mindoro Island, 0 m, 22 Jul. 1908.

**Paratypes.** FMNH 42783 (2), Leyte Island, 17 Mar. 1909; CAS-SU 14970 [ex USNM 135257] (1) and USNM 135257 (2), Dupon Bay, Leyte Island, 17 Mar. 1909; USNM 93414 (2), Albatross station 5547, Noble Point, Tulayan Is, Jolo, Sulu Archipelago, 283 m, 15 Sep. 1909.

**Remarks.** Five specimens were previously listed with USNM 135257.

#### ﻿﻿ORDER MYCTOPHIFORMES (45)

##### Family Neoscopelidae (221)

**(171) *Solivomerarenidens* Miller, 1947: 84, fig. 2**

**Holotype.**USNM 138929 [ex USNM 135928] (220.0), Albatross station 5488 (10°00.00'N, 125°06.75'E), off San Ricardo Point, Panaon Island, between Leyte and Mindanao islands, 1412 m, 31 Jul. 1909.

**Paratypes.**USNM 135928 (9), same data as holotype; USNM 135927 (3), Albatross station 5487, 11.2 miles off San Ricardo Point, Panaon Island, between Leyte and Mindanao Islands, 1339 m, 31 Jul. 1909; USNM 135929 (1), Albatross station 5491, 19.3 miles off Diuata Point, Mindanao, 1346 m, 1 Aug. 1909; USNM 135930 (3), Albatross station 5492, 15.2 miles off Diuata Point, Mindanao, 1344 m, 1 Aug. 1909; USNM 135931 (4), Albatross station 5494, 4.2 miles off Diuata Point, Mindanao, 1240 m, 2 Aug. 1909; USNM 135932 (1), Albatross station 5495, 94 miles off Diuata Point, Mindanao, 1785 m, 2 Aug. 1909; USNM 135933 (4), Albatross station 5526, 18.4 miles off Balicasag Island, between Siquijor and Bohol islands, 1472 m, 1 Aug. 1909; USNM 135934 (1), Albatross station 5515, 24.6 miles off Camp Overton Lighthouse, northern Mindanao, 8 Aug. 1909; USNM 135935 (1), Albatross station 5203, 5 miles off Limasaua Island, Sogod Bay, southern Leyte Island, 1417 m, 10 Apr. 1908; USNM 135419 (3), Albatross station 5428, 19.5 miles of 30^th^ of June Island, eastern Palawan, 2021 m, 3 Apr. 1909.

**Remarks.** Radiographs of the holotype are available in the USNM record. Some of the specimens of USNM 135928 were stained and exchanged with other museums.

##### Family Myctophidae (222)

**(172) *Diaphusaliciae* Fowler, 1934: 295, fig. 53**

**Holotype.**USNM 92316 (53.0), Albatross station 5233, Limasaua Island, between Bohol and Leyte islands, 183 m, 7 May 1908.

**Paratypes.**USNM 122349 (2), same data as holotype.

**Remarks.** The two paratypes (probably) were re-cataloged by Paxton (1979).

**(173) *Diaphusashmeadi* Fowler, 1934: 311, fig. 66**

= *Diaphusgarmani* Gilbert, 1906.

**Holotype.**USNM 93161 (70.0 TL), Albatross station 5291 (13°29.67'N, 121°00.75'E), Escarceo Lighthouse, southern Luzon, 316 m, 23 Jul. 1908.

**(174) *Diaphusatkinsoni* Fowler, 1934: 321, fig. 75**

**Holotype.**USNM 93159 (127.0 TL), Albatross station 5268 (13°42.00'N, 120°57'15"E), Verde Island Passage and Batangas Bay, Luzon, 311 m, 8 Jun. 1908.

**Remarks.** Questionably a synonym of *Diaphuswhitleyi* Fowler, 1934 (Paxton 1979). Further study is needed to confirm its status.

**(175) *Diaphusbryani* Fowler, 1934: 319, fig. 73**

= *Diaphusburtoni* Fowler, 1934.

**Holotype.**USNM 93150 (140.0 TL), Albatross station 5289 (13°41.83'N, 120°58.50'E), Matocot Point, southern Luzon, 315 m, 22 Jul. 1908.

**Remarks.** Published at the same time with *Diaphusburtoni* but considered a synonym by Paxton (1979). The precedence was given to *D.burtoni* as it appeared earlier in the publication.

**(176) *Diaphusburtoni* Fowler, 1934: 315, fig. 70**

**Holotype.**USNM 93146 (104.0 TL), Albatross station 5291 (13°29.67'N, 121°00.75'E), Escarceo Lighthouse, southern Luzon, 316 m, 22 Jul. 1908.

**(177) *Diaphuscarlsoni* Fowler, 1934: 312, fig. 67**

= *Diaphusjenseni* Tåning, 1932.

**Holotype.**USNM 93151 (34.0 TL), Albatross station 5263 (12°38.33'N, 121°37.33'E), Origon Point, off eastern Mindoro Island, 119 m, 4 Jun. 1908.

**(178) *Diaphusdehaveni* Fowler, 1934: 320, fig. 74**

**Holotype.**USNM 93160 (95.0 TL), Albatross station 5392, between Samar and Masbate islands, 247 m, 13 Mar. 1909.

**(179) *Diaphusehrhorni* Fowler, 1934: 304, fig. 60**

**Holotype.**USNM 92319 (78.0 TL), Albatross station 5387, Bagatao Island Lighthouse, between Burias and Luzon islands, 382 m, 11 Mar. 1909.

**Paratypes.** Uncat. (49).

**Remarks.** The original description mentioned 49 paratypes, but no further information was provided. The specific type locality was inferred in the USNM record.

**(180) *Diaphusfaustinoi* Fowler, 1934: 300, fig. 57**

**Holotype.**USNM 92321 (34.0 TL), Albatross station 5190 (10°08.25'N, 123°16.75'E), Pescador Island, east of Negros, 539 m, 1 Apr. 1908.

**(181) *Diaphusgudgeri* Fowler, 1934: 302, fig. 59**

= *Diaphusjenseni* Tåning, 1932.

**Holotype.**USNM 92322 (58.0 TL), Albatross station 5544, Coronado Point, northern Mindanao, 1388 m, 6 Sep. 1909.

**Paratypes.** Uncat. (4).

**Remarks.** The original description mentioned four paratypes but provided no further information. These were also not found in the USNM record.

**(182) *Diaphushandi* Fowler, 1934: 290, fig. 49**

**Holotype.**USNM 93163 (113.0 TL), Albatross station 5033 (09°27.25'N, 123°31.80'E), between Cebu and Siquijor islands, 790 m, 19 Aug. 1909.

**(183) *Diaphusharveyi* Fowler, 1934: 294, fig. 52**

= *Diaphusrichardsoni* Tåning, 1932.

**Holotype.**USNM 92317 (23.0 TL), Albatross station 5177, Escarceo Lighthouse, Verde Island Passage, Batangas, Luzon, 475 m, 24 Mar. 1908.

**Paratypes.** Uncat. (6).

**Remarks.** The original description mentioned six paratypes but provided no further information. Furthermore, it mentioned Manila Bay as the type locality with depth of 260 fathoms (475 m), but the USNM record showed it was collected in the Verde Island Passage at 46 m depth. No record was found in USNM for the paratypes.

**(184) *Diaphuskendalli* Fowler, 1934: 297, fig. 55**

= *Diaphusparri* Tåning, 1932.

**Holotype.**USNM 93157 (69.0 TL), Albatross station 5487 (10°02.75'N, 125°05.55'E), San Ricardo Point, between Leyte and Mindanao islands, 1339 m, 31 Jul. 1909.

**Remarks.** The original description indicated 1070 as the collection depth, but different from the USNM record.

**(185) *Diaphuskylei* Tåning, 1932: 133, fig. 5**

= *Diaphusjenseni* Tåning, 1932.

**Holotype.** ZMUC P2329206, 07°22.0'N, 121°16.0'E, west of Mindanao.

**Remarks.** The original description did not indicate the collection date.

**(186) *Diaphuslayi* Fowler, 1934: 292, fig. 51**

= *Diaphusaliciae* Fowler, 1934.

**Holotype.**USNM 93145 (38.0 TL), Albatross station 5500, Macabalan Point, northern Mindanao, 488 m, 4 Aug. 1909.

**(187) *Diaphuslongleyi* Fowler, 1934: 296, fig. 54**

= *Diaphusparri* Tåning, 1932.

**Holotype.**USNM 92320 (38.0 TL), Albatross station 5229, between Cebu and Leyte islands, 290 m, 7 May 1908.

**Remarks.** The original description indicated 530 m as the collection depth but different from the USNM record.

**(188) *Diaphuslucifrons* Fowler, 1934: 307, fig. 63**

**Holotype.**USNM 93147 (104.0 TL), Albatross station 5444 (12°43.85'N, 124°58.83'E), Atalaya Point, Batag Island, east of Luzon, 563 m, 3 Jun. 1909.

**(189) *Diaphusmeyeri* Fowler, 1934: 314, fig. 68**

= *Diaphusmalayanus* Weber, 1913.

**Holotype.**USNM 93152 (35.0 TL), Albatross station 5437 (15°45.90'N, 119°42.75'E), Hermana Mayor Lighthouse, west of Luzon, 183–1097 m, 8 May 1908.

**(190) *Diaphusmonodi* Fowler, 1934: 306, fig. 62**

= *Diaphuslucidus* (Goode & Bean, 1896).

**Holotype.**USNM 92315 (78.0 TL), Albatross station 5190 (10°08.25'N, 123°16.75'E), Pescador Island, Tañon Strait, east of Negros Island, 457 m, 1 Apr. 1908.

**Remarks.** The original description indicated 539 m as the collection depth, but different from the USNM record.

**(191) *Diaphusphillipsi* Fowler, 1934: 287, fig. 47**

**Holotype.**USNM 93149 (77.0 TL), Albatross station 5184, Lusaran Point, between Panay and Negros islands, 1033 m, 30 Mar. 1908.

**(192) *Diaphusreidi* Fowler, 1934: 309, fig. 64**

= *Diaphuslucidus* (Goode & Bean, 1896).

**Holotype.**USNM 93154 (96.0 TL), Albatross station 5368 (13°35.33'N, 121°48.0'E), Tayabas Lighthouse, Marinduque Island, 331 m, 23 Feb. 1909.

**(193) *Diaphusrivatoni* Bourret, 1985: 69, fig. 5, pl. 1A**

**Holotype.**MNHN 1984-0392 (female, 110.0), Musorstom 2 station 6 (13°55.98'N, 120°21.07'E), off Lubang Island, west of Luzon, 136–152 m.

**Remarks.** The original description indicated 128–143 m as the collection depth, but different from the USNM record.

**(194) *Diaphusstreetsi* Fowler, 1934: 291, fig. 50**

= *Diaphustermophilus* Tåning 1928.

**Holotype.**USNM 93162 (88.0 TL), Albatross station 5111 (13°45.25'N, 120°46.33'E), Sombrero Island, off southern Luzon, 432 m, 16 Jan. 1908.

**(195) *Diaphusthiollierei* Fowler, 1934: 289, fig. 48**

**Holotype.**USNM 93158 (98.0 TL), Albatross station 5199 (09°31.83'N, 124°40.00'E), Pamilacan Island, western Bohol, 0 m, 9 Apr. 1908.

**(196) *Diaphusumbroculus* Fowler, 1934: 317, fig. 72**

**Holotype.**USNM 93148 (140.0 TL), Albatross station 5268 (13°42.00'N, 120°57.25'E), Matocot Point, Verde Island Passage and Batangas Bay, Luzon, 311 m, 8 Jun. 1908.

**(197) *Diaphuswhitleyi* Fowler, 1934: 305, fig. 61**

**Holotype.**USNM 92318 (128.0 TL), Albatross station 5268 (13°42.00'N, 120°57.25'E), Matocot Point, Verde Island Passage and Batangas Bay, Luzon, 311 m, 8 Jun. 1908.

**Paratypes.** Uncat. (2).

**Remarks.** The original description mentioned two paratypes but provided no further information including in the USNM record.

**(198) *Myctophumgilberti* Evermann & Seale, 1907: 55, fig. 1**

= *Benthosemapterotum* (Alcock, 1890).

**Holotype.**USNM 55900 (63.5), Bulan, Sorsogon, Luzon.

**Paratypes.** BSMP (1), CAS-SU 22232 (1) and USNM 126397 [ex USBF 1487/4535] (1) (53.3–63.5), same data as holotype.

**Remarks.** The original description mentioned 20000 as the catalog number for CAS-SU 22232 (Böhlke 1953). BSMP specimen was presumed destroyed. The original description or USNM record did not indicate the collection date of the holotype.

**(199) *Serpablacki* Fowler, 1934: 284, fig. 44**

= *Bolinichthyspyrsobolus* (Alcock, 1890).

**Holotype.**USNM 92312 (80.0 TL), Albatross station 5507, Camp Overton Lighthouse, northern Mindanao, 777 m, 5 Aug. 1909.

**Paratypes.**USNM 122359 (2), same data as holotype.

**Remarks.** The original description mentioned two paratypes but provided no catalog number. USNM 122359 were presumed paratypes as stated in the note of specimens in the USNM record.

**(200) *Serpaturneri* Fowler, 1934: 285, fig. 45**

= *Lampanyctusturneri* (Fowler, 1934).

**Holotype.**USNM 92313 (61.0 TL), Albatross station 5497, Bantigue Island, between Leyte and Mindanao islands, 1756 m, 3 Aug. 1909.

**Paratype.** Uncat. (1), same data as holotype.

**Remarks.** The original description mentioned a paratype but provided no further information including in the USNM record.

#### ﻿﻿ORDER ZEIFORMES (49)

##### Family Parazenidae (235)

**(201) *Zencypho* Fowler, 1934: 350, fig. 103**

= *Cyttopsiscypho* (Fowler, 1934).

**Holotype.**USNM 93140 (112.0 TL), Albatross station 5517, Tagolo Point Lighthouse, northern Mindanao, 309 m, 9 Aug. 1909.

**Paratypes.**USNM 111725 (1), Albatross station 5519, Tagolo Point Lighthouse, northern Mindanao, 333 m, 9 Aug. 1909; USNM 111726 (1), Albatross station 5547, Dumalag Island, Davao Gulf, Mindanao, 247 m, 18 May 1908; USNM 111727 (1), Albatross station 5353, Cape Melville Lighthouse, northern Balabac Strait, Palawan, 271 m, 1 Jan. 1909; USNM 111728 (1), Albatross station 5418, Lauis Point, between Cebu and Bohol islands, 291 m, 25 Mar. 1909.

**Remarks.** A radiograph of the holotype is available in the USNM record.

##### Family Grammicolepididae (237)

**(202) *Macrurocyttusacanthopodus* Fowler, 1934: 351, fig. 104**

**Holotype.**USNM 93144 (43.0 TL), Albatross station 5467 (13°35.45'N, 123°37.30'E), Lagonoy Gulf, eastern Luzon, 878 m, 18 Jun. 1909.

**Remarks.** The holotype is mixed with several disarticulated remains of several specimens and stained with glycerine with bone based on the USNM record. A non-type specimen (USNM 331213) was removed as it came from different station than the type and was not considered as a type.

#### ﻿﻿ORDER GADIFORMES (51)

##### Family Bathygadidae (242)

**(203) *Bathygadusmultifilis* Günther, 1887: 155, pl. 42 (fig. B)**

= *Gadomusmultifilis* (Günther, 1887).

**Holotype.**BMNH 1887.12.7.146 (127.0), south of the Philippine islands, Challenger station 214, 914 m.

**Other catalog number.** NHMUK:ecatalogue:3112545.

**(204) *Bathygadusspongiceps* Gilbert & Hubbs, 1920: 381, fig. 1**

**Holotype.**USNM 78210. Darvel Bay, Borneo.

**Paratypes.**USNM 78235 (1), Albatross station 5467, Atulayan Island, east coast of Luzon, 878 m, 18 Jun. 1909; USNM 220978 [ex USNM 78235] (2), Albatross station 5274, Malavatuan Island, northwest of Lubang Island, Luzon, 960 m, 16 Jul. 1908; USNM 220980 [ex USNM 78235] (1), Albatross station 5460, Sialat Point Lighthouse, east coast of Luzon, 1033 m, 10 Jun. 1909.

**Remarks.** The original description or USNM record did not indicate the sizes of the specimens. USNM 78235 originally contained 6 specimens: 2 specimens removed to 220980; 1 specimen removed to 220979.

**(205) *Gadomusdenticulatus* Gilbert & Hubbs, 1920: 393, fig. 3**

**Holotype.**USNM 78207 (307.0), Albatross station 5505, Macabalan Point Lighthouse, northern Mindanao, 40 m, 5 Aug. 1909.

**Paratypes.**USNM 148983 (1), taken with holotype; USNM 148977 (1), Albatross station 5445, Batag Island, San Bernardino Strait, east of Luzon, 700 m, 3 Jun. 1909; USNM 148979 (1), Albatross station 5123, Malabrigo Lighthouse, east of Mindoro Island, 518 m, 2 Feb. 1908; USNM 148980 (1), Albatross station 5410, Bagacay Point Lighthouse, between Cebu and Leyte islands, 704 m, 18 Mar. 1909; USNM 148981 (1), Albatross station 5406, Ponson Island, Dupon Bay, Leyte Island, 17 Mar. 1909; USNM 148985 (2), Albatross station 5198, Balicasag Island, west of Bohol, 402 m, 9 Apr. 1908.

**(206) *Gadomusmagnifilis* Gilbert & Hubbs, 1920: 398, fig. 4**

**Holotype.**USNM 78208 (337.0+), Albatross station 5515, off northern Mindanao, 1280 m, 8 Aug. 1909.

**Paratypes.**USNM 78234 (1), Albatross station 5423, Cagayancillo Island, Palawan, 929 m, 31 Mar. 1909; USNM 221089 (1), Albatross station 5201, Limasaua Island, Sogod Bay, southern Leyte Island, 1013 m, 10 Apr. 1908.

**Remarks.** The type specimen was measured to the end of a very small pseudocaudal. USNM 221089 was removed from USNM 78234 since the two were collected from different localities.

**(207) *Reganiasulcata* Smith & Radcliffe in Radcliffe, 1912: 108, pl. 22 (fig. 3)**

= *Bathygadussulcatus* (Smith & Radcliffe, 1912).

**Holotype.**USNM 72925 (440.0), Albatross station 5423 (09°38.33'N, 121°11.00'E), near Cagayancillo Island, Palawan, 929 m, 31 Mar. 1909.

**Paratypes.**USNM 192597 (1) and USNM 149277 (2), same collection data as holotype, 622 m; USNM 149276 (1), Albatross station 5219, Mompog Island, between Marinduque and Luzon islands, 969 m, 23 Apr. 1908; USNM 149278-80 (2, 1, 1), Albatross stations 5527-5529, Balicasag Island, between Siquijor and Bohol islands, 717–807 m, 11 Aug. 1909; USNM 149281 (2), Albatross station 5536, Apo Island, between Negros and Siquijor islands, 510 m, 19 Aug. 1909; USNM 149282 (1), Philippines; USNM 149839 (4), Albatross station 5513, Camp Overton Lighthouse, northern Mindanao, 924, 7 Aug. 1909.

**Remarks.** The original description did not mention if paratypes were included, but only seen in the USNM record. USNM 192597 was considered a paratype by O. Okamura (May 1979). USNM 149280 originally had two specimens but was exchanged to BMNH. Both linen tags are still in the lot, although only one specimen is available. No associated data in the linen tag of USNM 149282, including the collection date.

##### Family Macrouridae (243)

**(208) *Coelorhynchusacutirostris* Smith & Radcliffe in Radcliffe, 1912: 134, fig. 10, pl. 30 (fig. 2)**

= *Coelorinchusacutirostris* Smith & Radcliffe, 1912.

**Holotype.**USNM 72947 (205.0), Albatross station 5418 (10°08.83'N, 123°52.33'E), Lauis Point Lighthouse, between Cebu and Bohol islands, 291 m, 25 Mar. 1909.

**(209) *Coelorhynchusargentatus* Smith & Radcliffe in Radcliffe, 1912: 137, pl. 31 (fig. 1)**

= *Coelorinchusargentatus* Smith & Radcliffe, 1912.

**Holotype.**USNM 72949 (female, 365.0), Albatross station 5172 (06°03.25'N, 120°35.33'E), Jolo Lighthouse, Sulu Archipelago, 35 m, 14 Feb. 1908.

**Paratypes.**USNM 149283 (1), Albatross station 5517, Tagolo Point Lighthouse, northern Mindanao, 309 m, 9 Aug. 1909; USNM 149284 (1), Albatross station 5417, Lauis Point Lighthouse, between Cebu and Bohol islands, 302 m, 25 Mar. 1909; USNM 149285 (1), Albatross station 5279, Malavatuan Island, southern Luzon, 214 m, 17 Jul. 1908.

**Remarks.** The original description did not mention any paratype but were found in USNM records. It also mentioned 582 m as the depth of capture of the holotype, but different in the USNM record (35 m). USNM 149284 was re-identified as *Coelorinchusacutirostris* by O. Okamura (Smithsonian Museum Archive 2024).

**(210) *Coelorhynchuscarinifer* Gilbert & Hubbs, 1920: 490, fig. 19**

= *Coelorinchuscarinifer* Gilbert & Hubbs, 1920.

**Holotype.**USNM 78223 (332.0), Albatross station 5111, Sombrero Island, off southern Luzon, 432 m, 16 Jan. 1908.

**(211) *Coelorhynchuscommutabilis* Smith & Radcliffe in Radcliffe, 1912: 128, pl. 29 (fig. 2)**

= *Coelorinchuscommutabilis* Smith & Radcliffe, 1912.

**Holotype.**USNM 72945 (320.0), Albatross station 5348 (10°57.75'N, 118°38.25'E), Tabonan Point, Palawan Passage, 686 m, 27 Dec. 1908.

**Paratypes.**USNM 148891 (1), Albatross station 5172, Jolo Lighthouse, Sulu Archipelago, 582 m, 5 Mar. 1908; USNM 149462-63 (1, 1), Albatross stations 5444-45, Atalaya Point, Batag Island, east of Luzon, 563–700 m, 3 Jun. 1909; USNM 149506 (1), Albatross station 5502, Macabalan Point Lighthouse, northern Mindanao, 391 m, 4 Aug. 1909.

**Remarks.** Paratypes were undoubted to be this species according to N. Nakayama. USNM 149506 was re-identified as *C.radcliffei* while USM 149462–63 were not *C.commutabilis* (did not provided other identification) (Smithsonian Museum Archive 2024).

**(212) *Coelorhynchusdorsalis* Gilbert & Hubbs, 1920: 469, fig. 13**

= *Coelorinchusdorsalis* Gilbert & Hubbs, 1920.

**Holotype.**USNM 78219 (male, 200.0), Albatross station 5329, Font Island, off northern Luzon, 388 m, 19 Nov. 1908.

**Paratype.**USNM 78232 (1, 155.0), Albatross station 5326, Hermanos Island, off northern Luzon, 421 m, 12 Nov. 1908.

**(213) *Coelorhynchusmacrolepis* Gilbert & Hubbs, 1920: 477, fig. 14.**

= *Coelorinchusmacrolepis* Gilbert & Hubbs, 1920.

**Holotype.**USNM 78220 (143.0), Albatross station 5111, Sombrero Island, off southern Luzon, 432 m, 16 Jan. 1908.

**Paratypes.**CAS-SU 24043 (19), same data as holotype; CAS-SU 23977 (15), southern Luzon, 16 Jan. 1908; CAS-SU 26774 (12), USNM 148884 (2), Albatross station 5124, and USNM 149301 (4), Albatross station 5365, Cape Santiago Lighthouse, Balayan Bay, Luzon, 391 m, 22 Feb. 1909; USNM 49586 (1), Albatross station 5281, Malavatuan Island, southern Luzon, 368 m, 18 Jul. 1908; USNM 149840 (1), Philippines.

**Remarks.** Some specimens (USNM 148884 and 149301) were mutilated and badly disintegrated, while USNM 149586 has no head based on the USNM record. No specific locality was indicated for USNM 149840.

**(214) *Coelorhynchusmacrorhynchus* Smith & Radcliffe in Radcliffe, 1912: 127, pl. 29 (fig. 1)**

= *Coelorinchusmacrorhynchus* Smith & Radcliffe, 1912.

**Holotype.**USNM 72944 (560.0), Albatross station 5367 (13°34.62'N, 121°07.50'E), Malabrigo Lighthouse, Verde Island Passage, Luzon, 329 m, 22 Feb. 1909.

**Paratypes.**USNM 148888 (4), Albatross station 5111, Sombrero Island, off southern Luzon, 432 m, 16 Jan. 1908.

**Remarks.** The original description mentioned nine paratypes (152.0–560.0). taken at depths of 330–750 m. Four specimens from the Philippines collected between 1908 and 1909 were found in the USNM record but did not state if these were the other unaccounted paratypes. These specimens were found in a multi-lot jar and separated into one single-lot jar.

**(215) *Coelorhynchusmaculatus* Gilbert & Hubbs, 1920: 446, fig. 9**

= *Coelorinchusmaculatus* Gilbert & Hubbs, 1920.

**Holotype.**USNM 78215. Between Gillolo and Makyan Islands, Indonesia.

**Paratype.**USNM uncat. (1), Albatross station 5366, Batangas Bay, Luzon, 439 m.

**Remarks.** The original description mentioned 12 paratypes; one collected specimen from South China Sea off southern Luzon was in poor condition but was not designated as a paratype. No record of paratype being deposited in USNM record.

**(216) *Coelorhynchusnotatus* Smith & Radcliffe in Radcliffe, 1912: 136, pl. 30 (fig. 3)**

= *Coelorinchusnotatus* Smith & Radcliffe, 1912.

**Holotype.**USNM 72948 (270.0), Albatross station 5162 (05°10.00'N, 119°4733'E), Tinakta Island, Sulu Archipelago, 421 m, 22 Feb. 1908.

**Paratypes.**USNM 148987 (3), Albatross station 5135, Jolo Lighthouse, Sulu Archipelago, 294 m, 7 Feb. 1908.

**Remarks.** The original description did not mention any paratype but was found in the USNM record.

**(217) *Coelorhynchusquincunciatus* Gilbert & Hubbs, 1920: 438, fig. 7**

= *Coelorinchusquincunciatus* Gilbert & Hubbs, 1920.

**Holotype.**USNM 78213 (female, 237.0), Albatross station 5392, Escarceo Lighthouse, between Samar and Masbate islands, 329 m, 23 Jul. 1908.

**Paratypes.**USNM 137824 (7), Albatross station 5392, Tubig Point, between Samar and Masbate islands, 247 m, 13 Mar. 1909; USNM 137825 (1, 188.0), Albatross station 5412, Lauis Point Lighthouse, between Cebu and Bohol islands, 296 m, 23 Mar. 1909; USNM 137823 (1, 182.0) and USNM 137826 (2), Albatross stations 5396-97, Panalangan Point, Talajit Island, between Samar and Masbate islands, 245–251 m, 15 Mar. 1909; USNM 137827 (2, 194.0–225.0), Albatross station 5121, Malabrigo Lighthouse, east of Mindoro Island, 198 m, 2 Feb. 1908.

**(218) *Coelorhynchusradcliffei* Gilbert & Hubbs, 1920: 498, figs 21–24**

= *Coelorinchusradcliffei* Gilbert & Hubbs, 1920.

**Holotype.**USNM 78224 (268.0), Albatross station 5503, Macabalan Point Lighthouse, off northern Mindanao, 413 m, 4 Aug. 1909.

**Paratypes.**USNM 149303 (1) and USNM 149304 (1), same locality as holotype, 391–402 m, 4–5 Aug. 1909; USNM 149287 (1), Albatross station 5122, Malabrigo Lighthouse, east of Mindoro Island, 402 m, 2 Feb. 1908; USNM 149288 (1), Albatross station 5221, San Andreas Island, between Marinduque and Luzon islands, 353 m, 24 Apr. 1908; USNM 149289-90 (1, 1), 149495 (1) and 149305, Albatross stations 5535-37, Apo Island, between Negros and Siquijor islands, 465–567 m, 19 Aug. 1909; USNM 149496 (1), Albatross station 5374, Tayabas Lighthouse, Marinduque Island, 347 m, 2 Mar. 1909.

**(219) *Coelorhynchussexradiatus* Gilbert & Hubbs, 1920: 458, fig. 11**

= *Coelorinchussexradiatus* Gilbert & Hubbs, 1920.

**Holotype.**USNM 78217 (male, 205.0 TL), Albatross station 5172 (06°03.25'N, 120°35.33'E), Jolo Lighthouse, Sulu Archipelago, 582 m, 5 Mar. 1908.

**Paratypes.**USNM 99476 (1), same data as holotype; CAS-SU 25434 (3) and USNM 99477 (3), Dammi Island, between Jolo and Tawi Tawi islands, Sulu Archipelago, 444 m, 21 Sep. 1909.

**(220) *Coelorhynchussmithi* Gilbert & Hubbs, 1920: 493, fig. 20**

= *Coelorinchussmithi* Gilbert & Hubbs, 1920.

**Holotype.**USNM 78212. Between Gillolo and Makyan Islands, Indonesia.

**Paratypes.**CAS-SU 23993 (2, 151.0–162.0 AL), Cagayancillo Island, Palawan, 622 m, 31 Mar. 1909; CAS-SU 69079 (1, 290.0+), Origin Point, east of Mindoro Island, 514 m, 2 Feb. 1908; CAS-SU 69081 (1) and CAS-SU 69082 (1, 84.0+), Apo Island, between Negros and Siquijor islands, 567 m, 19 Aug. 1909.

**(221) *Coelorhynchusthompsoni* Gilbert & Hubbs, 1920: 442, fig. 8**

= *Coelorinchusthompsoni* Gilbert & Hubbs, 1920.

**Holotype.**USNM 78214 (female, 206.0+ TL), Albatross station 5363, Cape Santiago Lighthouse, Balayan Bay, southwest of Luzon, 329 m, 20 Feb. 1909.

**Paratypes.**CAS-SU 25496 (1, 150.0), Legaspi Lighthouse, east of Luzon, 280 m, 7 Jun. 1909; USNM 148886 (2), Tagolo Point Lighthouse, northern Mindanao, 366 m, 9 Aug. 1909; USNM 149838 (1, 176.0), Sombrero Island, Balayan Bay and Verde Island Passage, Luzon, 291 m, 21 Jan. 1908.

**(222) *Coelorhynchustriocellatus* Gilbert & Hubbs, 1920: 466, fig. 12**

= *Coelorinchustriocellatus* Gilbert & Hubbs, 1920.

**Holotype.**USNM 78218 (male, 190.0+), Albatross station 5575 (05°28.33'N, 120°02.45'E), Mt. Dromedario, north of Tawi-Tawi Group, Sulu Archipelago, 576 m, 23 Sep. 1909.

**(223) *Coelorhynchusvelifer* Gilbert & Hubbs, 1920: 452, fig. 10**

= *Coelorinchusvelifer* Gilbert & Hubbs, 1920.

**Holotype.**USNM 78216 (male, 251.0 TL), Albatross station 5294, Escarceo Lighthouse, south Luzon, 446 m, 24 Jul. 1908.

**Paratypes.**USNM 149270 (1), same data as holotype; USNM 148887 (13), same locality as holotype, 316 m, 23 Jul. 1908; CAS-SU 25430 (20), CAS-SU 25431 (37) and USNM 149271 (3) Matocot Point, Verde Island and Batangas Bay, Luzon, 315–402 m, 22–24 Jul. 1908; USNM 149269 (5), Tagolo Point Lighthouse, northern Mindanao, 10 Aug 1909;; USNM 149272 (1), Matocot Point, Verde Island and Batangas Bay, Luzon, 402 m, 8 Jun. 1908.

**Remarks.**USNM 148887 specimens were disintegrated.

**(224) *Coelorhynchusweberi* Gilbert & Hubbs, 1920: 503, figs 25, 26**

= *Coelorinchusweberi* Gilbert & Hubbs, 1920.

**Holotype.**USNM 78225 (315.0), Albatross station 5325, Hermanos Island, off northern Luzon, 410 m, 12 Nov. 1908.

**(225) *Coryphaenoidessemiscaber* Gilbert & Hubbs, 1920: 410, fig. 6**

**Holotype.**USNM 83625 (251.0 TL), Albatross station 5215, Palanog Lighthouse, east of Masbate Island, 1105 m, 21 Apr. 1908.

**Paratypes.**USNM 148882 (1), same data as holotype; CAS-SU 25452 (2, 110.0–213.0), Balicasag Island, between Cebu and Siquijor islands, 609 m, 20 Aug. 1909; USNM 148883 (1), Albatross station 5124, Origon Point, east of Mindoro Is, 514 m, 2 Feb. 1908.

**(226) *Hymenocephalusbarbatulus* Gilbert & Hubbs, 1920: 539, fig. 34**

**Holotype.**USNM 83652 (98.0), Albatross station 5238, Lambajon Point, Davao Oriental, off east of Mindanao, 695 m, 12 May 1908.

**Paratype.** Uncat. (1, 57.0), same data as holotype.

**Remarks.** The original description did not provide a catalog number for the paratype and was not found in USNM record. The holotype was not found during the 1980 inventory (Smithsonian Museum Archive 2024).

**(227) *Hymenocephalusgracilis* Gilbert & Hubbs, 1920: 522, fig. 31**

= *Hymenogadusgracilis* (Gilbert & Hubbs, 1920).

**Holotype.**USNM 78228 (96.0), Albatross station 5292 (13°28.75'N, 121°01.20'E), Escarceo Lighthouse, off southern Luzon, 296 m, 23 Jul. 1908.

**Remarks.** Radiographs of the holotype are available in the USNM record.

**(228) *Hymenocephaluslongiceps* Smith & Radcliffe in Radcliffe, 1912: 111, pl. 23 (fig. 3)**

= *Hymenocephaluslongibarbis* (Günther, 1887).

**Holotype.**USNM 72928 (216.0), Albatross station 5459 (13°10.35'N, 123°59.90'E), Legaspi Lighthouse, east of Luzon, 368 m, 8 Jun. 1909.

**Paratypes.**USNM 149297 (2), same data as holotype.

**Remarks.** The original description did not mention paratypes but were found in USNM record (Smithsonian Museum Archive 2024).

**(229) *Hymenocephaluslongipes* Smith & Radcliffe in Radcliffe, 1912: 109, pl. 23 (fig. 1)**

**Holotype.**USNM 72926 (175.0), Albatross station 5421 (10°33.50'N, 122°26.00'E), between Panay and Guimaras islands, 251 m, 30 Mar. 1909.

**(230) *Hymenocephalusnascens* Gilbert & Hubbs, 1920: 535, fig. 33**

**Holotype.**USNM 78229. Sibuko Bay, Borneo.

**Paratypes.**USNM uncat. (10), South China Sea, off southern Luzon, 366–721 m; USNM uncat. (12), Marinduque Island, 618 m; USNM uncat. (1), east of Luzon, 700 m; USNM uncat. (1), northern Mindanao, 774 m.

**Remarks.** The original description did not provide catalog numbers for paratypes, only the total number of specimens. No specimens from the Philippines are listed in the USNM record (Smithsonian Museum Archive 2024). Dr. Tomio Iwamoto mentioned the presence of paratypes in lots deposited at CAS, but none of these specimens originate from the Philippines.

**(231) *Hymenocephalustorvus* Smith & Radcliffe in Radcliffe, 1912: 110, pl. 23 (fig. 2)**

**Holotype.**USNM 72927 (160.0), Albatross station 5548 (06°00.33'N, 120°45.58'E), Jolo Lighthouse, Sulu Archipelago, 424 m, 17 Sep. 1909.

**Paratypes.**USNM 148961 (4), USNM 149033 (11), USNM 149466 (1), and USNM 149469 (1), Albatross stations 5535-37, Apo Island, between Cebu and Siquijor islands, 465–567 m, 19 Aug. 1909; USNM 148963 (16), Albatross station 5260, Balanja Point, off SE of Mindoro Island, 428 m, 3 Jun. 1908; USNM 148964 (2), Albatross station 5222, San Andreas Island, between Marinduque and Luzon islands, 357 m, 24 Apr. 1908; USNM 148959 (9), Albatross station 5506; USNM 148960 (6), Albatross station 5501; 149464 (8), Albatross station 5222; USNM 149465 (3), Albatross station 5505; and USNM 149468 (1), Albatross station 5504, Mabacalan Point Lighthouse, northern Mindanao, 366–479 m, 4–5 Aug. 1909; USNM 149467 (1) and USNM 149476 (1), Albatross stations 5112-13, Sombrero Island, off southern Luzon, 291–326 m, 17 Jan. 1908; USNM 149470 (2), Albatross station 5198, Balicasag Island, west of Bohol Island, 402 m, 9 Apr. 1908; USNM 149471 (3), Albatross station 5294, Escarceo Lighthouse, southern Luzon, 446 m, 24 Jul. 1908; USNM 149472 (1), Albatross station 5498, Lauis Point Lighthouse, between Cebu and Bohol islands, 320 m, 25 Mar. 1909; USNM 149457 (15), Albatross station 5282 and USNM 149473 (1), Albatross station 5279, Malavatuan Island, southern Luzon, 214–454 m, 17–18 Jul 1908; USNM 149474 (2) Albatross station 5371 and USNM 149475 (1), Albatross station 5368, Tayabas Lighthouse, Marinduque Island, 152–331 m, 23–24 Feb. 1909; USNM 148962 (12) Albatross station 5363 and USNM 149589 (6), Albatross station 5365, Cape Santiago Lighthouse, Balayan Bay, Luzon, 329–391 m, 20–22 Feb. 1909.

**Remarks.** The original description did not mention any paratype but they were found in the USNM records. Radiographs of the holotype are available in the USNM record. (Smithsonian Museum Archive 2024).

**(232) *Lionurusdecimalis* Gilbert & Hubbs, 1920: 560, fig. 40**

= *Sphagemacrurusdecimalis* (Gilbert & Hubbs, 1920).

**Holotype.**USNM 82668 (155.0), Albatross station 5348, Tabonan Point, Palawan Passage, 686 m, 27 Dec. 1908.

**Paratype.**USNM 82667 (1, ca. 155.0), same data as holotype.

**(233) *Macrouruscamurus* Smith & Radcliffe in Radcliffe, 1912: 122, pl. 27 (fig. 2)**

= *Coryphaenoidescamurus* (Smith & Radcliffe, 1912).

**Holotype.**USNM 72939 (102.0), Albatross station 5428 (09°13.00'N, 118°51.25'E), 30^th^ of June Island, eastern Palawan, 2021 m, 3 Apr. 1909.

**(234) *Macrourusdubius* Smith & Radcliffe in Radcliffe, 1912: 117, pl. 25 (fig. 3)**

= *Coryphaenoidesdubius* (Smith & Radcliffe, 1912).

**Holotype.**USNM 72934 (425.0), Albatross station 5511 (08°15.33'N, 123°57.00'E), Camp Overton Lighthouse, northern Mindanao, 750 m, 7 Aug. 1909.

**(235) *Macrourushyostomus* Smith & Radcliffe in Radcliffe, 1912: 121, pl. 27 (fig. 1)**

= *Mataeocephalushyostomus* (Smith & Radcliffe, 1912).

**Holotype.**USNM 72938 (280.0), Albatross station 5470 (13°37.33'N, 123°41.15'E), Atulayan Island, Lagonoy Gulf, east of Luzon Island, 1024 m, 18 Jun. 1909.

**(236) *Macrouruslucifer* Smith & Radcliffe in Radcliffe, 1912: 113, pl. 24 (fig. 1)**

= *Lucigaduslucifer* (Smith & Radcliffe, 1912).

**Holotype.**USNM 72929 (217.0), Albatross station 5516 (08°46.00'N, 123°32.33'E), Tagolo Point Lighthouse, northern Mindanao, 320 m, 9 Aug. 1909.

**Paratypes.**USNM 148885 (8), same locality and date as the holotype, 333 m.

**(237) *Macrourusmacronemus* Smith & Radcliffe in Radcliffe, 1912: 115, pl. 24 (fig. 3)**

= *Kuronezumiamacronema* (Smith & Radcliffe, 1912).

**Holotype.**USNM 72931 (343.0), Albatross station 5424 (09°37.08'N, 121°12.62'E), Cagayancillo Island, Palawan, 622 m, 31 Mar. 1909.

**Paratype.**CAS-SU 25233 (1), Philippines.

**Remarks.** No paratype was also mentioned but was found in Böhlke (1953) and the CAS records without a specific locality.

**(238) *Macrourusmicrops* Smith & Radcliffe in Radcliffe, 1912: 116, pl. 25 (fig. 2)**

= *Coryphaenoidesmicrops* (Smith & Radcliffe, 1912).

**Holotype.**USNM 72933 (430.0), Albatross station 5470 (l13°37.33'N, 123°41.15'E), Atulayan Island, Lagonoy Gulf, east of Luzon, 1024 m, 18 Jun. 1909.

**Paratypes.**CAS-SU 25446 (2, 114.0), Hermanos Island, Cagayan, northern Luzon, 410 m, 12 Nov. 1908.

**(239) *Macrourusnigromarginatus* Smith & Radcliffe in Radcliffe, 1912: 114, pl. 24 (fig. 2)**

= *Lucigadusnigromarginatus* (Smith & Radcliffe, 1912).

**Holotype.**USNM 72930 (180.0), Albatross station 5569 (05°33.25'N, 120°15.33'E), Simaluc Island, Sulu Archipelago, 554 m, 22 Sep. 1909.

**Paratypes.**USNM 148897 (1), Albatross station 5505, Macabalan Point Lighthouse, northern Mindanao, 402 m, 5 Aug. 1909; USNM 148898 (1), Albatross station 5575, Mt. Dromedario, north of Tawi Tawi Group, Sulu Archipelago, 576 m, 23 Sep. 1909; USNM 148899 (1), Albatross station 5265, Matocot Point, Verde Island Passage and Batangas Bay, Luzon, 247 m, 6 Jun. 1908; USNM 148900 (1), Albatross station 5216, Anima Sola Island, Ragay Gulf, between Burias and Luzon islands, 393 m, 22 Apr. 1908; USNM 148901 (2), Albatross station 5172, Jolo Lighthouse, Sulu Archipelago, 582 m, 5 Mar. 1908; USNM 148902 (1), Albatross station 5198, Balicasag Island, western Bohol, 402 m, 9 Apr. 1908; USNM 148903 (1), Albatross station 5418, Lauis Point Lighthouse, between Cebu and Bohol islands, 291 m, 25 Mar. 1909; USNM 148904 (1), Albatross station 5261, Balanja Point, off SE Mindoro Island, 265 m, 4 Jun. 1908; USNM 148906 (1), Albatross station 5565, Dammi Island, between Jolo and Tawi-Tawi islands, Sulu Archipelago, 444 m, 21 Sep. 1909; USNM 148908 (1), Albatross station 5508, Camp Overton Lighthouse, northern Mindanao, 494 m, 5 Aug. 1909; USNM 148889 (3), Albatross station 5519, USNM 148907 (3), Albatross station 5516 and USNM 148909 (3), Albatross station 5523, Tagolo Point Lighthouse, northern Mindanao, 320–333 m, 9–10 Aug. 1909.

**(240) *Macrourusparadoxus* Smith & Radcliffe, 1912: 115, pl. 25 (fig. 1)**

= *Coryphaenoidesrudis* Günther, 1878.

**Holotype.**USNM 72932 (585.0), Albatross station 5428 (09°13.00'N, 118°51.25'E), 30^th^ of June Island, east of Palawan, 2021 m, 3 Apr. 1909.

**(241) *Macrourusproximus* Smith & Radcliffe in Radcliffe, 1912: 119, pl. 26 (fig. 2)**

= *Nezumiaproxima* (Smith & Radcliffe, 1912).

**Holotype.**USNM 72936 (292.0), Albatross station 5202 (10°12.00'N, 125°04.17'E), Limasaua Island, Sogod Bay, southern Leyte, 918 m, 10 Apr. 1908.

**Paratypes.**USNM 135338 (1), same locality and date as holotype, 1013 m; USNM 135339 (1), Albatross station 5527, Balicasag Island, Bohol, 717 m, 11 Aug. 1909.

**Remarks.** The original description did not mention the paratype’s locality but was found in the USNM records (Smithsonian Museum Archive 2024).

**(242) *Malacocephalusluzonensis* Gilbert & Hubbs, 1920: 541, fig. 35**

**Holotype.**USNM 83626 (56.5 AL), Albatross station 5440, San Fernando Point Lighthouse, off west of Luzon, 315 m, 10 May 1909.

**Paratype.**USNM 135347 (1), Albatross station 5476, San Bernardino Lighthouse, east of Luzon, 494 m, 24 Jun. 1909.

**(243) *Mataeocephalusnigrescens* Smith & Radcliffe in Radcliffe, 1912: 125, pl. 28 (fig. 2)**

= *Mataeocephalusacipenserinus* (Gilbert & Cramer, 1897).

**Holotype.**USNM 72942 (270.0), Albatross station 5492 (09°12.75'N, 125°20.00'E), Diuata Point, Bohol Sea, between Leyte and Mindanao islands, 1344 m, 1 Aug. 1909.

**Paratypes.**USNM 149308 (1), same data as holotype; USNM 149306-07 (1, 1), and USNM 149309 (4), Albatross stations 5423-25, Cagayancillo Island, Palawan, 622–929 m, 31 Mar. 1909; USNM 149310 (1), Albatross station 5515, Camp Overton Lighthouse, northern Mindanao, 1280 m, 8 Aug. 1909; USNM 149312 (1), Albatross station 5348, Tabonan Point, Palawan Passage, 686 m, 27 Dec. 1908; USNM 149313 (3), Albatross station 5219, Mompog Island, between Marinduque and Luzon islands, 969 m, 23 Apr. 1908.

**(244) *Trachonurusrobinsi* Iwamoto, 1997: 942, fig. 1**

**Holotype.**CAS-SU 25438 (female, 207.0 TL), Albatross station 5494 (09°06.50'N, 125°18.67'E), Diuata Point, between Leyte and Mindanao islands, 1240 m, 2 Aug. 1909.

**Paratypes.**CAS-SU 69755 (5), same data as holotype; CAS-SU 23944 (1), Malabrigo Island, east of Mindoro, 518 m, 2 Feb. 1908; USNM 99439 (1) Albatross station 5513, and USNM 149767 (3), Albatross station 5423, Camp Overton Lighthouse, northern Mindanao, 814–924 m, 7 Aug. 1909; USNM 149766 (2), Albatross station 5407, Ponson Island, Camotes Sea, Leyte, 640 m, 17 Mar. 1909; USNM149771 (1), Albatross station 5528, Balicasag Island, between Siquijor and Bohol islands; USNM 149774 (1), Albatross station 5425, Cagayancillo Island, Palawan, 905 m, 31 Mar. 1909.

**Remarks.** A photograph and a radiograph of the holotype are available in the CAS record.

**(245) *Ventrifossadivergens* Gilbert & Hubbs, 1920: 549, fig. 37**

**Holotype.**USNM 78230. Sibuko Bay, Borneo.

**Paratypes.**USNM 113235 (1), Albatross station 5269, Matocot Point, Verde Island Passage and Batangas Bay, Luzon, 402 m, 8 Jun. 1908; USNM 148975 (3), Albatross station 5296; USNM 149459 (3), Albatross station 5289; USNM 149361 (11), Albatross station 5289; and USNM 149363 (5), same locality as USNM 113235, 315–391 m, 22–24 Jul. 1908; USNM 148988 (1), Albatross station 5284, Malavatuan Island, south Luzon, 772 m, 20 Jul. 1908; and USNM 150400 (1), Albatross station 5329, Font Island, off northern Luzon, 388 m, 19 Nov. 1908.

**(246) *Ventrifossanigrodorsalis* Gilbert & Hubbs, 1920: 546, fig. 36**

**Holotype.**USNM 83627 (214.0) Albatross station 5502 (08°37.62'N, 124°35.00'E), Macabalan Point Lighthouse, northern Mindanao, 391 m, 4 Aug. 1909.

**Paratypes.**USNM 148974 (5) and USNM 149302 (14), same data as holotype; USNM 148795 (2), USNM 161465 (1), USNM 148986 (2) and USNM 149601 (5), San Andreas Island, between Marinduque and Luzon islands, 353–357 m, 24 Apr. 1908; USNM 149030 (11), Tayabas Lighthouse, Marinduque Island, 347 m, 2 Mar. 1909; USNM 149300 (6), Camp Overton Lighthouse, northern Mindanao, 494 m, 5 Aug. 1909; CAS-SU 25441 (4) and USNM 149458 (7), Cape Santiago Lighthouse, Balayan Bay, Luzon, 329–392 m, 20–22 Feb. 1909; USNM 135693 (1) and USNM 149481 (2), Atalaya Point, Batag Island, northern Samar, 563–700 m, 3 Jun. 1909; USNM 148989 (9), USNM 149483 (1) and USNM 149603 (1), Capitancillo Lighthouse, between Cebu and Leyte islands, 346 m, 18 Mar. 1909; USNM 149485 (1), Balicasag Island, western Bohol, 402 m, 9 Apr. 1908; USNM 149507 (1), Tabonan Point, Palawan Passage, 686 m, 27 Dec. 1908; USNM 149605 (2), Jolo Lighthouse, Sulu Archipelago, 582 m, 5 Mar. 1908; USNM 149489 (1), USNM 149608 (3) and USNM 161472 (2), Bagatao Island Lighthouse, between Burias and Luzon islands, 413 m, 11 Mar. 1909; USNM 149482 (2), USNM 149486 (1), USNM 149604 (2), USNM 149606 (3), USNM 161464 (2), USNM 161466 (3) and USNM 161468 (4), Macabalan Point Lighthouse, northern Mindanao, 366–413 m, 4–5 Aug. 1909; USNM 149362 (4), USNM 149484 (1) and USNM 161467 (1), Apo Island, between Cebu and Siquijor islands, 510–567 m, 19–20 Aug. 1909; USNM 149602 (1), USNM 149607 (1) and USNM 161470 (2), Tagolo Point Lighthouse, northern Mindanao, 366 m, 10–20 Aug. 1909; USNM 99449 (1), USNM 149488 (1), USNM 161469 (1) and USNM 161471 (1), Ponson Island, Dupon Bay, 347–545 m, 17 Mar. 1909; USNM 161473 (1), Sombrero Island, off southern Luzon, 291 m, 17 Jan. 1908; USNM 161474 (1), Philippines, 7 Nov 1907 – 29 Jan 1910; CAS-SU 25450 (3), Origon Point, east of Mindoro Island, 514 m, 2 Feb. 1908.

**Remarks.**USNM 148795 specimens are in very poor condition, exact count was difficult to determine.

##### Family Trachyrincidae (244)

**(247) *Macrouroidesinflaticeps* Smith & Radcliffe in Radcliffe, 1912: 139, pl. 31 (fig. 2)**

**Holotype.**USNM 72950 (147.0), Albatross station 5450 (13°23.25'N, 124°00.50'E), East Point, near Batan Island, Lagonoy Gulf, Luzon, 746 m.

**Remarks.** The original description did not indicate the collection date.

##### Family Moridae (246)

**(248) *Physiculusnigrescens* Smith & Radcliffe in Radcliffe, 1912: 105, pl. 22 (fig. 1)**

**Holotype.**USNM 72923 (274.0), Albatross station 5296 (13°40.15'N, 120°57.75'E), Matocot Point, Verde Island Passage, Luzon, 384 m, 24 Jul. 1908.

#### ﻿﻿ORDER HOLOCENTRIFORMES (52)

##### Family Holocentridae (253)

**(249) *Holocentruszebra* Marion de Procé, 1822: 132**

= *Epinephelussexfasciatus* (Valenciennes, 1828).

Manila Bay, Luzon.

**Remarks.** No type known. Unused senior synonym of *Epinephelussexfasciatus* (Valenciennes, 1828); declared *nomen oblitum* (Parenti and Randall 2020). The original description did not indicate the collection date.

**(250) *Myripristisrobusta* Randall & Greenfield, 1996: 43, pl. 5C**

**Holotype.** BPBM 23473, Bagalangit, Calumpan Peninsula, Batangas, Luzon, 45 m, 28 Jul. 1978.

**(251) *Myripristisschultzei* Seale, 1910: 504**

= *Myripristisviolacea* Bleeker, 1851.

**Holotype.** BSMP 3899 (160.0), Samal Island, Davao Gulf, Mindanao, 4 May 1908.

**Remarks.** Having been deposited in BSM, the type specimen was presumed lost.

**(252) *Ostichthysalamai* Matsunuma, Fukui & Motomura, 2018: 24, figs 1b, 2b, 4, 5g–i, 8f, 9b, 13a–d, 17**

**Holotype.** KAUM-I 51657 (119.0), 10°41.0'N, 122°35.0'E, off Iloilo, Panay Island, Philippines.

**Paratypes.** UPVMI 442 (1, 157.0), collected with the holotype; KAUM-I. 52612 (1, 122.0), off Iloilo, Panay Island (Central Fish Market), 13 Feb. 2013; KAUM-I. 56011 (1, 179.0), KAUM-I. 56012 (1, 145.0), KAUM-I. 56013 (1, 147.0), NSMT-P 130434 [ex KAUM-I. 56010] (1, 171.0), off Iloilo, Panay Island (Central Fish Market), 20 Aug. 2013; USNM 440362 [ex KAUM-I. 56017] (1, 127.0), off Iloilo, Panay Island (Central Fish Market), 21 Aug. 2013; AMS I. 47400-001 [ex KAUM-I. 91856] (1, 127.0), off Iloilo, Panay Island (Miagao Fish Market), 13 Sept. 2016.

**Remarks.** Samples were purchased in Central Fish Market Iloilo City, and Miag-ao Fish Market, Iloilo.

##### Family Anoplogastridae (254)

**(253) *Anoplogasterbrachycera* Kotlyar, 1986: 544 [142], fig. 4**

**Holotype.** ZMMU P-15945 (60.0), 07°35.00'N, 121°20.00'E, Sulu Sea, west of Zamboanga, Mindanao, 1000 m.

**Paratype.** ZMMU P-15946 (1, 20.0), same data as holotype.

**Remarks.** The original description did not indicate the collection date.

##### Family Trachichthyidae (258)

**(254) *Gephyroberyxphilippinus* Fowler, 1938: 38, fig. 11**

= *Gephyroberyxdarwinii* (Johnson, 1866).

**Holotype.**USNM 93345 (155.0), Albatross station 5516 (08°46.00'N, 123°32.33'E), off Tagolo Point Lighthouse, northern Mindanao, 320 m, 9 Aug. 1909.

**Paratypes.**USNM 93401 (7), same data as holotype.

**(255) *Hoplostethusmelanopterus* Fowler, 1938: 36, fig. 9**

**Holotype.**USNM 93329 (170.0), Albatross station 5373 (13°40.00'N, 121°31.17'E), off Tayabas Lighthouse, Marinduque Island, 618 m, 2 Mar. 1909.

**Paratypes.**CAS-SU 140192 [ex USNM 93330] (2), USNM 93335 (4), USNM 93338 (1), CAS-SU 40192 (2), Diuata Point, between Leyte and Mindanao islands, 1240–1344 m, 1–2 Aug. 1909; USNM 93325 (1) and USNM 93342 (6), Tayabas Lighthouse, Marinduque Island, 618 m, 2 Mar. 1909; USNM 93327 (1), Matocot Point, southern Luzon, 315 m, 22 Jul. 1908; USNM 93331 (2) and USNM 93332 (4), Camp Overton Lighthouse, northern Mindanao, 774–924 m, 7 Aug. 1909; USNM 93333 (5), Limasaua Island, Sogon Bay, Leyte Island, 144 m, 10 Apr. 1908; USNM 93334 (5), Origin Point, east of Mindoro Island, 514 m, 2 Feb. 1908; CAS-SU 40191 [ex USNM 93336] (1), USNM 93328 (1), USNM 93340 (1) and USNM 99030 (3), Balicasag Island, between Cebu and Siquijor islands, 717–803 m, 11–19 Aug. 1909; USNM 93337 (1), Apo Island, between Negros and Siquijor islands, 468 m, 19 Aug. 1909; USNM 93341 (2), Palanog Lighthouse, east of Masbate Island, 1105 m, 21 Apr. 1908.

**(256) *Hoplostethusmetallicus* Fowler, 1938: 37, fig. 10**

**Holotype.**USNM 93344 (123.0), Albatross station 5189 (09°56.33'N, 123°15.00'E), Pescador Island, Tañon Strait, east of Negros Island, 549 m, 1 Apr. 1908.

**Paratypes.**USNM 93391 (4) and USNM 93395 (17), same data as holotype; USNM 93396 (1), Philippines; USNM 93397 (1), Albatross station 5297, Matocot Point, south Luzon 362 m, 24 Jul. 1908.

**Remarks.** The specific locality of paratype USNM 93396 was not provided in the original description or USNM record.

**(257) *Hoplostethusrobustispinus* Moore & Dodd, 2010: 139, figs 1–3**

**Holotype.** YPM 10130 (male, 340.0), 14°18.00'N, 123°21.00'E, east of Calagua Island, eastern Luzon, 648–660 m, 27 Sep. 1995.

**(258) *Paratrachichthyslatus* Fowler, 1938: 40, fig. 12**

= *Aulotrachichthyslatus* (Fowler, 1938).

**Holotype.**USNM 93346 (70.0), Albatross station 5273, Corregidor Lighthouse, southern Luzon, 208 m, 14 Jul. 1908.

**Paratypes.**USNM 93412 (1), same data as holotype; USNM 93394 (1), same date and locality as holotype, 216 m; USNM 93411 (1), Albatross station 5376, Tayabas Lighthouse, Marinduque Island, 165 m, 2 May 1909; USNM 93413 (1), Albatross station 5241 and USNM 93420 (4), Albatross station 5243, Uanivan Island, Pujada Bay, Mindanao, 393–399 m, 14–15 May 1908.

#### ﻿﻿ORDER BERYCIFORMES (54)

##### Family Melamphaidae (265)

**(259) *Scopelogadusunispinis* Ebeling & Weed, 1963: 21, fig. 11**

**Holotype.** ZMUC P4174. Indonesia.

**Paratypes.**USNM 151303 (1), Albatross station 5492, Diuata Point, between Leyte and Mindanao islands, 1344 m, 1 Aug. 1909; USNM 151304 (3), Albatross station 5494, same locality as preceding, 1240 m, 2 Aug. 1909; USNM 151305 (5), Albatross station 5215, Palanog Lighthouse, east of Masbate Island, 1105 m, 21 Apr. 1908; USNM 151306 (1), Albatross station 5379, Mompog Island, Marinduque, 1682 m, 4 Mar. 1909; USNM 151307 (1), Albatross station 5410, Bagacay Point Lighthouse, between Cebu and Leyte islands, 704 m, 18 Mar. 1909; USNM 151308 (1), Albatross station 5544, Coronado Point, northern Mindanao, 1388 m, 6 Sep. 1909; USNM 151309 (2), Albatross station 5533, Balicasag Island, between Cebu and Siquijor islands, 790 m, 19 Aug. 1909.

#### ﻿﻿ORDER OPHIDIIFORMES (55)

##### Family Carapidae (267)

**(260) *Echiodonanchipterus* Williams, 1984: 415, figs 1A, 4**

**Holotype.**USNM 258905 (male), 11°35.75'N, 123°55.53'E, southwest of Caduruan Point, between Negros and Masbate islands, 0–79 m, 6 Jun. 1978.

**Remarks.** A radiograph of the holotype is available in the USNM record.

**(261) *Encheliophisvermicularis* Müller, 1842: 323**

**Neotype.**USNM 298307 (female, 130.0 TL), 13°49.0'N, 120°37.0'E, Calatagan Lagoon, Batangas, Luzon, 0–1 m, 8 Mar. 1980.

**Remarks.** The original type specimens could not be found in any European museum and were considered lost. A neotype was designated by Markle and Olney (1990) and additional specimens were used for additional description. Radiographs of the neotype are available in the USNM record.

##### Family Ophidiidae (268)

**(262) *Bassozetusrobustus* Smith & Radcliffe in Radcliffe, 1913: 156, pl. 11 (fig. 3)**

**Holotype.**USNM 74140 (360.0), Albatross station 5349 (10°54.00'N, 118°26.33'E), Tabonan Point, Palawan Passage, 1335 m, 27 Dec. 1908.

**Remarks.** Radiographs of the holotype are available in the USNM record.

**(263) *Dicrolenelongimana* Smith & Radcliffe in Radcliffe, 1913: 144, pl. 8 (fig. 1)**

**Holotype.**USNM 74130 (255.0), Albatross station 5488 (10°00.09’”N, 125°06.75'E), San Ricardo Point, Panaon Island, between Leyte and Mindanao islands, 1412 m, 31 Jul. 1909.

**Paratypes.**BMNH 1939.4.1.2–3 [ex USNM] (2) and USNM 99093 (1), Bagacay Point Lighthouse, between Cebu and Leyte islands, 704 m, 17–18 Mar. 1909; USNM 99075 (1), Palanog Lighthouse, east of Masbate Island, 505 m, 21 Apr. 1908; USNM 99076 (1), Capitancillo Island Lighthouse, between Leyte and Cebu islands, 333 m, 16 Mar. 1909; USNM 99077 (1), Origin Point, east of Mindoro Island, 505–514 m, 2 Feb. 1908; USNM 99078 (5), USNM 99079 (1), USNM 99080 (4), USNM 99097 (1) and USNM 99099 (3), Diuata Point, between Leyte and Mindanao islands, 1240–1785 m, 1–2 Aug. 1909; USNM 99094 (4) and USNM 99081 (1), Ponson Island, Dupon Bay, Leyte, 545–640 m, 17 Mar. 1909; USNM 99084 (1), Camp Overton Lighthouse, northern Mindanao, 750 m, 7 Aug. 1909; USNM 99090 (1), Limasaua Island, Sogod Bay, Leyte, 1013 m, 10 Apr. 1908; USNM 99095 (1), Balicasag Island, between Cebu and Siquijor islands, 790 m, 19 Aug. 1909; USNM 99096 (1), Cagayancillo Island, Palawan, 929 m, 31 Mar. 1909; USNM 99101 (1), Mompog Island, between Marinduque and Luzon islands, 969 m, 23 Apr. 1908.

**Other catalog number.**BMNH 1939.4.1.2 (NHMUK:ecatalogue:2517667).

**Remarks.** Radiographs of the holotype are available in the USNM record.

**(264) *Dicrolenetristis* Smith & Radcliffe in Radcliffe, 1913: 145, pl. 8 (fig. 2)**

**Holotype.**USNM 74131 (255.0), Albatross station 5467 (13°35.45'N, 123°37.30'E), Atulayan Island, Lagonoy Gulf, Luzon, 878 m, 18 Jun. 1909.

**Paratypes.**BMNH 1939.4.1.4–5 [ex USNM] (2), Atulayan Island, east of Luzon, 1041 m, 1 Jan. 1909; USNM 99071 (1), same locality as BMNH 1939.4.1.4–5, 18 Jun. 1909.

**Other catalog number.**BMNH 1939.4.1.4–5 (NHMUK:ecatalogue:251766).

**(265) *Glyptophidiumeffulgens* Nielsen & Machida, 1988: 302, fig. 11**

= *Glyptophidiumargenteum* Alcock, 1889.

**Holotype.**USNM 99158 (female, 202.0), Albatross station 5410, Bagacay Point Lighthouse, east of Cebu Island, 705 m, 18 Mar. 1909.

**Paratypes.**USNM 272001 (1 female, 212.0+) and ZMUC P77783 (1 male, 228.0), same data as holotype.

**Remarks.** Radiographs of the holotype are available in the USNM record.

**(266) *Glyptophidiumlucidum* Smith & Radcliffe in Radcliffe, 1913: 161, pl. 12 (fig. 3)**

**Holotype.**USNM 74144. Between Gillolo and Kayoa Islands, Indonesia.

**Paratype.**USNM 99109 (1), Albatross 5348, Tabonan Point, Palawan Passage, 686 m, 27 Dec. 1908.

**Remarks.** The original description did not provide information of paratype but was found in the USNM record.

**(267) *Glyptophidiumoceanium* Smith & Radcliffe in Radcliffe, 1913: 162, pl. 12 (fig. 4)**

**Holotype.**USNM 74145 (210.0), Albatross station 5444 (12°43.85'N, 124°58.83'E), northern Samar Island, 563 m, 3 Jun. 1909.

**Paratypes.** Uncat. (1), Albatross station 5298, Batangas Bay, Luzon, 256 m; Uncat. (1) Albatross station 5331, west of Luzon, 326 m.

**Remarks.** The original description provided no catalog numbers for paratypes. The USNM record provided Atalaya Point, Batag Island on the east coast of Luzon, between San Bernardino Strait and San Miguel Bay as the holotype locality. Radiographs of the holotype are available in the USNM records.

**(268) *Homostolusacer* Smith & Radcliffe in Radcliffe, 1913: 147, pl. 8 (fig. 3)**

**Holotype.**USNM 74132 (195.0), Albatross station 5508 (08°17.40'N, 124°11.79'E), Camp Overton Lighthouse, northern Mindanao, 494 m, 5 Aug. 1909.

**Remarks.** Radiographs of the holotype are available in the USNM records.

**(269) *Hypopleuroncaninum* Smith & Radcliffe in Radcliffe, 1913: 165, pls 13 (fig. 2), 14**

**Holotype.**USNM 74147. Between Gillolo and Kayoa Islands, Indonesia.

**Paratypes.**USNM 99198 (1, 18.9), Albatross station 5523, Tagolo Point Lighthouse, northern Mindanao, 10 Aug. 1909; USNM 99202 (1, 22.0), Albatross station 5372, Tayabas Lighthouse, Marinduque Island, 274 m, 24 Feb. 1909.

**Remarks.** The original description did not mention the catalog numbers of the paratypes but were found in the USNM records.

**(270) *Luciobrotulabartschi* Smith & Radcliffe in Radcliffe, 1913: 171, pl. 16 (fig. 2)**

**Holotype.**USNM 74151 (260.0), Albatross station 5348 (10°57.75"N, 118°38.25"E), Tabonan Point, Palawan Passage, 686 m, 27 Dec. 1908.

**Remarks.** Radiographs of the holotype are available in the USNM records.

**(271) *Mastigopterusimperator* Smith & Radcliffe in Radcliffe, 1913: 159, pl. 12 (fig. 1)**

**Holotype.**USNM 74142 (535.0), Albatross station 5495 (09°06.50'N, 125°00.33'E), Diuata Point, between Leyte and Mindanao islands, 1785 m, 2 Aug. 1909.

**Remarks.** Radiographs of the holotype are available in the USNM records.

**(272) *Monomitopusmicrolepis* Smith & Radcliffe in Radcliffe, 1913: 150, pl. 9 (fig. 3)**

**Holotype.**USNM 74156 (250.0), Albatross station 5410 (10°28.75'N, 124°05.50'E), Bagacay Point Lighthouse, between Cebu and Leyte islands, 704 m, 18 Mar. 1909.

**Paratypes.**USNM 99051 (5), Albatross station 5290, Matocot Point, southern Luzon, 391 m, 22 Jul. 1908; USNM 99053 (1), Albatross station 5295, Escarceo Lighthouse, southern Luzon, 422 m, 24 Jul. 1908.

**Remarks.** Radiographs of the holotype are available in the USNM records.

**(273) *Monomitopuspallidus* Smith & Radcliffe in Radcliffe, 1913: 148, pl. 9 (fig. 1)**

**Holotype.**USNM 74133 (195.0), Albatross station 5259 (11°57.50'N, 121°42.25'E), Caluya Island, between Mindoro and Panay islands, 571 m, 3 Jun. 1908.

**Paratypes.**BMNH 1939.4.1.7 [ex USNM] (1), Cagayancillo Island, Palawan, 1 Feb. 1903; USNM 99261 (0) and USNM 99263 (1), same locality as BMNH 1939.4.1.7, 622–905 m, 31 Mar. 1909; USNM 99262 (1), Albatross station 5378, Mompog Island, Marinduque, 722 m, 4 Mar. 1909; USNM 99264 (1), Albatross station 5487, San Ricardo Point, between Leyte and Mindanao islands, 1339 m, 31 Jul. 1909.

**Other catalog number.**BMNH 1939.4.1.7 (NHMUK:ecatalogue:2517670).

**Remarks.** Radiographs of the holotype are available in the USNM record. Two species share BMNH 1939.4.1.7 as the catalog number in the NHM record.

**(274) Neobythites (Watasea) fasciatus Smith & Radcliffe in Radcliffe, 1913: 142, pl. 7 (fig. 4)**

= *Neobythitesfasciatus* Smith & Radcliffe, 1913.

**Holotype.**USNM 74129 (193.0), Albatross station 5290 (13°40.15'N, 120°59.50'E), Matocot Point, Batangas Bay, Luzon, 391 m, 22 Jul. 1908.

**Paratypes.**BMNH 1939.4.1.9 [ex USNM] (1), USNM 99056 (1), USNM 99065 (1), USNM 99064 (2) and USNM 99259 (1), Tagolo Point Lighthouse, northern Mindanao, 309–333 m, 9–10 Aug. 1909; USNM 99052 (1), Albatross station 5162, Tinakta Island, Sulu Archipelago, 421 m, 22 Feb. 1908; USNM 99055 (2), Albatross station 5325, Hermanos Island, off northern Luzon, 410 m, 12 Nov. 1908; USNM 99240 (1), Albatross station 5290, Matocot Point, southern Luzon, 391 m, 22 Jul. 1908.

**Other catalog number.**BMNH 1939.4.1.9 (NHMUK:ecatalogue:2517672).

**Remarks.**USNM 99240 was not mentioned as a paratype in the original description, but it is considered a paratype in the original USNM ledger entry. Radiographs of the holotype are available in the USNM record.

**(275) *Neobythiteslongipes* Smith & Radcliffe in Radcliffe, 1913: 139, pl. 7 (fig. 1)**

**Holotype.**USNM 74126 (302.0), Albatross station 5550 (06°02.00'N, 120°44.67'E), Jolo Lighthouse, Sulu Archipelago, 472 m, 17 Sep. 1909.

**Paratypes.**USNM 99236 (1), same data as the holotype; BMNH 1939.4.1.10 [ex USNM] (1) and USNM 99091 (1), Albatross station 5549, same locality as the holotype, 481 m, 1 Apr. 1903; USNM 99069 (1), Albatross station 5574, Simaluc Island, northern Tawi-Tawi Island, Sulu Archipelago, 622 m, 23 Sep. 1909; USNM 99070 (2), USNM 99092 (2) and USNM 99229 (1), Albatross station 5564, Dammi Island, between Jolo and Tawi-Tawi islands, 432–444 m, 21 Sep. 1909; USNM 99073 (1), Albatross station 5580, Sibutu Island Peak, between Sibutu Island and Borneo, 296 m, 25 Sep. 1909; USNM 99089 (1), Albatross station 5575, Mt. Dromedario, northern Tawi-Tawi, Sulu Archipelago, 576 m, 23 Sep. 1909; USNM 99204 (1), Albatross station 5326, Hermanos Island, off northern Luzon, 421 m, 12 Nov. 1908.

**Remarks.**USNM 99089 and 99236 were not mentioned as paratypes in the original description, but they are considered paratypes in the original USNM ledger entry. A radiograph of the holotype is available in the USNM record. BMNH 1939.4.1.10 was not found in NHM’s record.

**(276) Neobythites (Watasea) purus Smith & Radcliffe in Radcliffe, 1913: 141, pl. 7 (fig. 3)**

= *Neobythitespurus* Smith & Radcliffe, 1913.

**Holotype.**USNM 74128 (161.0), Albatross station 5392 (12°12.58'N, 124°02.80'E), near Destacado Island, between Samar and Masbate islands, 247 m, 13 Mar. 1909.

**Paratypes.**USNM 308953 (1), Albatross station 5393, Pangalanan Point, Talajit Island, between Samar and Masbate islands, 249 m, 13 Mar. 1909.

**Remarks.** Radiographs of the holotype are available in the USNM record.

**(277) Neobythites (Watasea) unimaculatus Smith & Radcliffe in Radcliffe, 1913: 140, pl. 7 (fig. 2)**

= *Neobythitesunimaculatus* Smith & Radcliffe, 1913.

**Holotype.**USNM 74127. Sibuko Bay, Borneo.

**Paratypes.**USNM 76678 (1), Albatross station 5520, Tagolo Point Lighthouse, northern Mindanao, 187 m, 10 Aug. 1909.

**Remarks.** The original description did not provide further information of one specimen but was found in the USNM record.

**(278) *Spottobrotulaamaculata* Cohen & Nielsen, 1982: 497, fig. 1**

= *Siremboamaculata* (Cohen & Nielsen, 1982).

**Holotype.**USNM 224567 (295.0), 11°38.0'N, 123°52.0'E, southwest of Caduruan Point, between Negros and Masbate islands, 0–90 m, 5 Jun. 1978.

**Paratypes.**USNM 224568 (2, 165.0–405.0) and ZMUC P77717 (1, 297.0), same data as holotype.

**Remarks.** A radiograph of the holotype is available in the USNM record.

**(279) *Tenuicephalusmultitrabs* Schwarzhans & Møller, 2021: 77, figs 4B, 39, 40**

**Holotype.**CAS 83066-3 (153.0+), 13°20.50'N, 124°16.50'E, Lagonoy Gulf, Luzon, 1037–1100 m, 24 Sep. 1995.

**Paratypes.**CAS 83066-2 (156.0), same data as holotype.

**(280) *Tenuicephalussquamilabrus* Schwarzhans & Møller, 2021: 81, figs 2, 42, 43**

**Holotype.** AMS I.36456-007 (175.0), 13°21.00'N 124°12.00'E, Lagonoy Gulf, Luzon, 1037–1100 m.

**Remarks.** The original description did not indicate the collection date.

**(281) *Umaliusphilippinus* Herre & Herald, 1950: 312, fig. 1**

= *Sirembojerdoni* (Day, 1888).

**Holotype.**USNM 112107, Manila Bay, Luzon, 24–46 m, 27 Jun. 1947.

##### Family Bythitidae (269)

**(282) *Bythiteslepidogenys* Smith & Radcliffe in Radcliffe, 1913: 172, pl. 16 (fig. 3)**

= *Cataetyxlepidogenys* (Smith & Radcliffe, 1913).

**Holotype.**USNM 74152 (79.0), Albatross station 5214 (12°25.30'N, 123°37.25'E), Palanot Lighthouse, east of Masbate Island, 399 m, 21 Apr. 1908.

**Remarks.** Radiographs of the holotype are available in the USNM record.

**(283) *Cataetyxplatycephalus* Smith & Radcliffe in Radcliffe, 1913: 169, pl. 16 (fig. 1)**

= *Pseudonussquamiceps* (Lloyd, 1907).

**Holotype.**USNM 74150. Mareh Island, Moluccas Passage, Indonesia.

**Paratypes.**USNM 398909 (1, 78.0), Albatross station 5515, Camp Overton Lighthouse, northern Mindanao, 1280 m, 8 Aug. 1909.

**(284) *Diplacanthopomabrunnea* Smith & Radcliffe in Radcliffe, 1913: 167, pl. 13 (fig. 3)**

= *Diplacanthopomabrunneum* Smith & Radcliffe, 1913.

**Holotype.**USNM 74148 (200.0), Albatross station 5348 (10°57.75'N, 118°38.25'E), Tabonan Point, Palawan Passage, 686 m, 27 Dec. 1908.

**Remarks.** Radiographs of the holotype are available in the USNM record.

**(285) *Grammonusrobustus* Smith & Radcliffe in Radcliffe, 1913: 168, pl. 13 (fig. 4)**

**Holotype.**USNM 74149 (143.0), Albatross station 5409 (10°38.00'N, 124°13.13'E), between Cebu and Leyte islands, 346, 18 Mar. 1909.

**Remarks.** Radiographs of the holotype are available in the USNM record.

**(286) *Hephthocaracrassiceps* Smith & Radcliffe in Radcliffe, 1913: 174, pl. 17 (figs 1–2)**

**Holotype.**USNM 74154. Buton Strait, Indonesia.

**Paratypes.**BMNH 1939.4.1.1 [ex USNM] (1), Mompog Island, between Marinduque and Luzon islands, 969 m, 1 Apr. 1897; USNM 99122 (1), USNM 99165 (2) and USNM 99234 (7), same locality with BMNH 1939.4.1.1, 23 Apr. 1908; USNM 99123 (1) and USNM 99139 (1), Cagayancillo Island, Palawan, 929 m, 31 Mar. 1909; USNM 99151 (1), USNM 99210 (1), USNM 99212 (1) and USNM 99218 (8), San Ricardo Point, Panaon Island, between Leyte and Mindanao islands, 1339–1412 m, 31 Jul. 1909; USNM 99121 (1), USNM 99124 (1), USNM 99134 (4), USNM 99214 (2) and USNM 99177 (3), Camp Overton Lighthouse, northern Mindanao, 750–1280 m, 7–8 Aug. 1909; USNM 99135 (1), USNM 99219 (6) and USNM 99233 (5), Limasaua Island, Sogod Bay, southern Leyte, 1,013 m, 10 Apr. 1908; USNM 99137 (USNM 99209, 6 m) and USNM 99220 (2), Diuata Point, between Leyte and Mindanao islands, 1240–1344 m, 1–2 Aug. 1909.

**Other catalog number.**BMNH 1939.4.1.1 (NHMUK:ecatalogue:2517665).

**Remarks.** The original description mentioned “Buton, Philippines” as the holotype locality but Buton is located in Indonesia. Paratypes were also not mentioned in the original description but were found in USNM and BMNH’s records (NHM 2024; Smithsonian Museum Archive 2024). Four specimens share BMNH 1939.4.1.1 as the catalog number in NHM record.

**(287) *Microbrotulapolyactis* Anderson, 2005: 38, fig. 2**

= *Microbrotulabentleyi* Anderson, 2005.

**Holotype.** AMS I.20779-128. Cape York Peninsula, Queensland, Australia.

**Paratypes.**USNM 227221 (male, 39.0), 09°36.90'N, 123°10.10'E, off East of Bais, Negros Island, 0–37 m, 17 Jun. 1978; USNM 227224 (1 female, 26.3 + 4 male, 21.5–33.5), Caceres Reef, Cebu Island, 24–31 m, 18 May 1979.

**Remarks.** Both *M.polyactis* and *M.bentleyi* were described at the same time. Schwarzhans and Nielsen (2011) examined the type specimens from the two species, but none of the characters used to separate the two species were validated. Thus, they concluded that there is a relatively large degree of intraspecific variability in *M.bentleyi*, and selected *bentleyi* over *polyactis*, as it appears first in the original description.

**(288) *Xenobythitesarmiger* Smith & Radcliffe in Radcliffe, 1913: 173, pl. 16 (fig. 4)**

= *Bellottiaarmiger* (Smith & Radcliffe, 1913).

**Holotype.**USNM 74153 (76.0), Albatross station 5504 (08°35.33'N, 124°36.00'E), Macajalar Bay, northern Mindanao, 366, 5 Aug. 1909.

**Remarks.** Radiographs of the holotype are available in the USNM record.

##### Family Dinematichthyidae

**(289) *Alionematichthysplicatosurculus* Møller & Schwarzhans, 2008: 109, figs 14–15**

**Holotype.**USNM 384198 (male, 79.0), 10°34.75'N, 122°30.50'E, Pulang Duta, Sinabsapan, Guimaras Island, 0–4 m, 24 Sep. 1995.

**Paratypes.**USNM 394981 [ex USNM 384198] (1 male, 45.0 + 3 females, 40.0–62.0), same data as holotype; USNM 366596 (female, 4.0), near Giligaon, north of Maloh, Negros Island, 0–2 m, 26 Apr. 1979.

**(290) *Alionematichthyssuluensis* Møller & Schwarzhans, 2008: 122, figs 24, 25**

**Holotype.** AMS I.40109-028 (male, 40.0), 12°21.70'N, 121°27.57'E, southern tip of Buyamao Island, off SE Mindoro Island, 0–20 m, 30 May 2000.

**Paratypes.** AMS I.40149-028 (1 male, 30.0 + 5 females, 30.0–42.0 + 3 juveniles, 13.0–17.0), same data as holotype; USNM 263691 (1 male, 43.0 + 5 females, 41.0–45.0) and USNM 376186 (2 males, 32.0–34.0 + 1 female, 41.0), Solino Island, Zamboanga, Mindanao, 0–5 m, 3–4 May 1979; USNM 263694 (6 males + 12 females, 27.0–49.0), Cocoro Island, Cuyo, Palawan; USNM 376216 (4 males + 1 female, 29.0–45.0), USNM 376179 (3 males + 6 females, 26.0–48.0) and USNM 366602 (female, 34.0), Negros Island; USNM 366601 (male, 64.0), Apo Island, Negros Island; USNM 374183 (male, 31.0), Talisayan Point, San Joaquin, Lawigan, Iloilo, Panay Island, 0–7 m, 25 Sep. 1995; USNM 376181 (3 males, 37.0–44.0), Putic Island, Cuyo, Palawan, 0–4.6 m, 2 May 1978; USNM 376182 (2 males, 34.0–44.0 m), near Giligaon, north of Maloh, Negros Island, 0–2 m, 26 Apr. 1979.

**(291) *Brotulinellataiwanensis* Schwarzhans, Møller & Nielsen, 2005: 80, figs 4, 5**

**Holotype.**USNM 221048. south of Chin-chiao-wan, south end of Taiwan.

**Paratypes.**ANSP 163510 [ex. USNM 300086] (2 females, 35.0–49.0 + 1 juvenile, 31.0), White Beach past Mahatae, Batan Island, Batanes, 0–6.5 m, 2 Apr. 1987; USNM 318065 (male, 36.5), same locality as ANSP 163510; USNM 374178 (1 male, 38.0 + 4 females, 32.0–60.0) and ZMUC P771469 (female, 32.0), same locality as ANSP 163510, 2 Apr. 1987; USNM 374176 (2 males, 37.0–40.0 + 1 female, 50.0 + 1 juvenile, 23.0), Maybag Island, Babuyan Islands, 8 Mar. 1990.

**(292) *Diancistruskarinae* Schwarzhans, Møller & Nielsen, 2005: 119, figs 36, 37**

**Holotype.** BPBM 36712. Bunkaken, Sulawesi, Indonesia.

**Paratypes.** AMS I.18677-019 (female, 82.0), Philippines, 1974; BPBM 28527 (female, 67.0), Sumilon Island, SE of Cebu, 3 Jun. 1981; USNM 263661 (1 male, 75.0; 1 female, 35.0) and ZMUC P 771477 (female, 75.0), 09°0.00'N, 118°0.00'E, West Sulu Sea, Palawan, 7 Aug. 1979; ROM 55147 (female, 48.0), 09°N, 123°E, Visayan islands, Jun. 1988.

**Remarks.** The original description did not provide a specific locality for one paratype (AMS I.18677-019). One of the three species of USNM 263661 was donated to ZMUC (Smithsonian Museum Archive 2024).

**(293) *Diancistrusmachidai* Schwarzhans, Møller & Nielsen, 2005: 131, figs 47, 48**

**Holotype.**USNM 372962. Tallabassi Bay, Sulawesi, Indonesia.

**Paratypes.**USNM 99224 (male, 41.0), Surigao, Mindanao, 8 May 1908; USNM 263686 (1 male, 42.0), Cuyo Island, Palawan, 23 May 1978; USNM 300088 (1 male, 60.0) and USNM 374181 (1 male, 34.0), White Beach, Batan Island, Batanes, 1–2 May 1987; USNM 374193 (female, 47.0), NW of Paliton Village, Siquijor Island, 10 May 1978.

**Remarks.** In the USNM record, one of 4 specimens of USNM 300088 was removed to USNM 384594, and two of three specimens were removed to USNM 388305. However, USNM 384594 is a nontype, identified as *Diancistrusfuscus*, and removed from USNM 263688. Similarly, USNM 388305 specimens are also nontype identified as *Diancistrus* sp.

**(294) *Diancistrusspringeri* Schwarzhans, Møller & Nielsen, 2005: 147, figs 62, 63**

**Holotype.** AMS I.34501-023. Off Eailiti, Flores, Indonesia.

**Paratypes.** AMS I.40161-016 (1 male, 35.0 + 3 females, 25.0–48.0), off Mindoro Island, 3 Jun. 2000; USNM 263695 (1 male, 42.0 + 1 female, 26.0 + 2 juveniles, 20.0–23.0), Pescador Island, Tañon Strait, west of Cebu Island, 18–24 m, 7 May 1979.

**(295) *Paradiancistruscuyoensis* Schwarzhans, Møller & Nielsen, 2005: 157, figs 73–74**

**Holotype.**USNM 263688 (male, 36.0), 10°53.00'N, 121°11.00'E, west of Cocoro Island, Cuyo, Palawan, 0–21 m, 26 May 1978.

**Paratypes.**USNM 365840 (female, 57.0), Pamilican Island, south of Bohol Island, 0–33 m, 12 Jun. 1978.

**(296) *Ungusurculusphilippinensis* Schwarzhans & Møller, 2007: 90, figs 44, 45**

**Holotype.** WAM P.31397-010 (male, 37.0), off Talampetan, Busuanga Island, Palawan, 1 Feb. 1998.

**Paratypes.** WAM P.31397-020 (female, 40.0), same data as holotype; USNM 346843 (4 males, 36.0–43.0 + 2 females, 37.0–41.0), Guimaras Island, 24 Sep. 1995; CAS 46045 (male, 47.0), near Giligaon, north of Maloh, southern tip of Negros Island 1948; USNM 374184 (4 males, 30.0–45.0 + 4 females, 24.0–44.0 + 1 juvenile, 15.0) and USNM 374186 (6 males, 33.0–41.0 + 10 females, 27.0–46.0 + 1 juvenile, 25.0), same locality as CAS 46045, 0–3 m, 24–26 Apr. 1979; USNM 374190 (8 males, 30.0–48.0 + 6 females, 34.0–52.0) and ZMUC P771625–26 (1 male, 46.0 + 1 female, 48.0), Maloh, NE Negros Island, 0–3 m, 18 May 1979.

**Remarks.** The original description included the geographical coordinate of the type locality (12°06.00'N, 110°51.00'E) which corresponds to a location off the east coast of Vietnam. However, these coordinates did not match the name of the locality mentioned. In the USNM records, two of 16 specimens were donated to ZMUC (Smithsonian Museum Archive 2024). The original description cited 14 specimens but ZMUC returned 15 specimens.

**(297) *Ungusurculuswilliamsi* Schwarzhans & Møller, 2007: 97, figs 50–51**

**Holotype.**USNM 346941 (male, 45.0), 10°34.00'N, 122°30.00'E, Pulang Duta, Sinabsapan, Guimaras Island, 0–4 m, 24 Sep. 1995.

**Paratypes.**USNM 384197 (1 male, 35.0 + 8 females, 21.0–45.0 + 1 juvenile, 21.0) and ZMUC P771627–28 (1 male, 35.0 + 1 female, 42.0), same data as holotype.

**Remarks.** In the USNM record, 11 of 17 specimens from USNM 346941 were removed to USNM 384197 (2 were donated to ZMUC), and 5 of 6 specimens were removed to USNM 384198. However, USNM 384198 is the holotype of *A.plicatosurculus*.

#### ﻿﻿ORDER BATRACHOIDIFORMES (56)

##### Family Batrachoididae (272)

**(298) *Halophrynehutchinsi* Greenfield, 1998: 697, figs 1–4**

**Holotype.**USNM 150899 (female, 91.8), Pamantungan Reef or Quinituary Reef, Catbalogan, Samar Island, 1–6 m, 5 Apr. 1908.

**Paratypes.**USNM 150927 (1, 97.2), Ragay Gulf, Luzon, 1–9 m, 11 Mar. 1909; CAS 126908 (11, 97.1–141.4) and FMNH 47500 (1, 116.1), Culion Island, Palawan, Apr. – May 1931; CAS-SU 38260 (1, 87.1), Busuanga Island, Palawan, 29 Jun. 1940; CAS-SU 20462 (1, 74.3), Cuyo Island, Palawan.

**Remarks.**USNM record indicated 15 Apr. 1908 as the collection date. A radiograph of the holotype is available in the USNM record (Smithsonian Museum Archive 2024).

#### ﻿﻿ORDER KUTRIFORMES (57)

##### Family Apogonidae (274)

**(299) *Amiaalbomarginata* Smith & Radcliffe in Radcliffe, 1912: 438, pl. 35 (fig. 2)**

= *Jaydiaalbomarginata* (Smith & Radcliffe, 1912).

**Holotype.**USNM 68402 (102.0), fish market in Cavite, Luzon.

**Remarks.** The original description or USNM did not indicate the collection date. Radiographs are available in the USNM record.

**(300) *Amiaangustata* Smith & Radcliffe in Radcliffe, 1911: 253, fig. 1**

= *Ostorhinchusangustatus* (Smith & Radcliffe, 1911).

**Holotype.**USNM 68399 (85.0), Malanipa Island, east of Zamboanga, Mindanao, 3–5 m, 8 Sep. 1909.

**Remarks.** Radiographs of the holotype are available in the USNM record.

**(301) *Amiaatrogaster* Smith & Radcliffe in Radcliffe, 1912: 439, pl. 35 (fig. 3)**

= *Ostorhinchusatrogaster* (Smith & Radcliffe, 1912).

**Holotype.**USNM 70249 (59.0), Albatross station 5442 (16°30.60'N, 120°11.10'E), San Fernando Point Lighthouse, west of Luzon, 82 m, 10 May 1909.

**Paratypes.**USNM 163227 (7, 46.0–59.0), taken with holotype.

**(302) *Amiacardinalis* Seale, 1910: 519**

**Holotype.** BSMP 5463 (40.0), Puerto Princesa, Palawan Island, 21 Aug. 1908.

**Remarks.** Uncertain status (Greenfield 2001; Fraser and Randal 2002; Mabuchi et al. 2014). The type specimen was presumed destroyed.

**(303) *Amiacavitensis* Jordan & Seale, 1907: 16, fig. 5**

= *Ostorhinchuscavitensis* (Jordan & Seale, 1907).

**Holotype.**CAS-SU 9241 (69.9), Cavite, Luzon, 1 Jun. 1900.

**Remarks.** A photograph and a radiograph of the holotype are available in the CAS record.

**(304) *Amiacompressa* Smith & Radcliffe in Radcliffe, 1911: 246, pls 20–21**

= *Ostorhinchuscompressus* (Smith & Radcliffe, 1911).

**Holotype.**USNM 68398 (102.0), Bisucay Island, Cuyo, Palawan, 2–5 m, 9 Apr. 1909.

**Paratypes.** 200 specimens from 45 localities.

**Remarks.** The original description mentioned many paratypes from different localities but did not give other information. No other specimens were also noted in the USNM record. Localities mentioned spanned from Port Matalvi on the west of Luzon and Quinalasag Island on the east, southward throughout the Philippine Archipelago to Borneo and the Moluccas in Indonesia.

**(305) *Amiadiencaea* Smith & Radcliffe in Radcliffe, 1912: 431, pl. 34 (fig. 1)**

= *Apogonsemiornatus* Peters, 1876.

**Holotype.**USNM 70243 (41.0), Sulade Island, Jolo, Sulu Archipelago, 7 Nov. 1907.

**(306) *Amiadiversa* Smith & Radcliffe in Radcliffe, 1912: 434, pl. 37 (fig. 1)**

= *Ostorhinchusdiversus* (Smith & Radcliffe, 1912).

**Holotype.**USNM 70246 (female, 78.0), a small stream in Canmahala Bay, Ragay Gulf, Luzon, 11 Mar. 1909.

**(370) *Amiagilberti* Jordan & Seale, 1905: 777, fig. 3**

= *Zoramiagilberti* (Jordan & Seale, 1905).

**Holotype.**USNM 51941 (41.9), southern Negros Island, 1901.

**Paratypes.**USNM 211818 [ex USNM 51941] (1) and CAS-SU 9135 (1), taken with the holotype.

**Remarks.** The original description did not indicate the collection date of the holotype. Radiographs of the holotype are available in the USNM record. USNM 211818 was separated from the holotype.

**(308) *Amiagriffini* Seale, 1910: 117, pl. 2 (fig. 2)**

= *Ostorhinchusgriffini* (Seale, 1910).

**Holotype.** BSMP 5701 (125.0), Bantayan Island, Cebu.

**Paratypes.** BSMP 5696 (1, 124.0) and BSMP 5698 (1, 135.0), same locality as holotype.

**Remarks.** The original description did not indicate the collection date of the type specimens. All type specimens were presumed destroyed.

**(309) Amia (Amioides) grossidens Smith & Radcliffe in Radcliffe, 1912: 440, pl. 36 (fig. 1)**

= *Amioidesgrossidens* (Smith & Radcliffe, 1912).

**Holotype.**USNM 70250 (101.0), Albatross station 5442 (16°30.60'N, 120°11.10'E), off San Fernando Point Lighthouse, west of Luzon, 83 m, 10 May 1909.

**(310) *Amiajenkinsi* Evermann & Seale, 1907: 73, fig. 9**

= *Ostorhinchusjenkinsi* (Evermann & Seale, 1907).

**Holotype.**USNM 55907 (86.4), Bulan, Sorsogon, Luzon.

**Remarks.** The original description did not indicate the collection date.

**(311) *Amiamagnifica* Seale, 1910: 507**

**Holotype.** BSMP 5192 (40.0), Balabac Island, Palawan, 10 Aug. 1908.

**Paratypes.** BSMP (numerous).

**Remarks.** Uncertain status (Mabuchi et al. 2014). All type specimens were presumed destroyed.

**(312) *Amiamelas* Fowler, 1918: 17, fig. 8**

= *Apogonmelanopterus* Fowler & Bean, 1930.

**Holotype.**ANSP 47491, Philippines.

**Remarks.** Subjectively invalid; secondarily preoccupied by *Apogonmelas* Bleeker, 1848 in *Amia*; replaced by *Apogonichthysmelanopterus* Fowler & Bean, 1930. A photograph and radiograph of the holotype are available in ANSP record. The original description did not indicate the collection date and exact type locality.

**(313) *Amianigrocincta* Smith & Radcliffe in Radcliffe, 1912: 435, pl. 37 (fig. 2)**

= *Ostorhinchusnigrocincta* (Smith & Radcliffe, 1912).

**Holotype.**USNM 70247 (91.0), Albatross station 5143 (06°05.83'N, 121°02.25'E), Jolo Island, Sulu Archipelago, 35 m, 15 Feb. 1908.

**Paratypes.**USNM 122267 (2), same data as holotype.

**(314) *Amiaparvula* Smith & Radcliffe in Radcliffe, 1912: 432, pl. 34 (fig. 2)**

= *Ostorhinchusparvulus* (Smith & Radcliffe, 1912).

**Holotype.**USNM 70244 (39.0), Tataan Pass, Tawi-Tawi Group, Sulu Archipelago, 21 Feb. 1908.

**Paratypes.** Uncat. (35), Saboon Island, Ragay Bay, Luzon.

**Remarks.** The original description mentioned many specimens and selected the largest as the holotype. Among the samples, 35 (the largest is 3 cm) were from Saboon Island in Ragay Bay (Gulf) but provided no catalog numbers for these paratypes. Several females were also noted that contained eggs. USNM 171156 contained 35 specimens from Saboon Island, Ragay Gulf collected on 10 Mar. 1909. However, the USNM records did not mention whether USNM 171156 contained paratypes mentioned in the original description.

**(315) *Amiaradcliffei* Fowler, 1918: 25, fig. 11**

= *Ostorhinchusradcliffei* (Fowler, 1918).

**Holotype.**ANSP 47500 (90.0), Philippines.

**Paratypes.**ANSP 47501 [ex ANSP 47501–04] (4, 75.0–90.0), Philippines.

**Remarks.** The original description did not provide specific localities and collection dates. Based on the ANSP’s record, the type specimens were mixed and some were identified as *Apogonangustatus* by G.R. Allen in 1989.

**(316) *Amiarobusta* Smith & Radcliffe in Radcliffe, 1911: 254, pl. 24; fig. 2**

= *Ostorhinchuscookii* (Macleay, 1881).

**Holotype.**USNM 68400 (87.0), Jolo reefs, Sulu Archipelago.

**Paratypes.** Uncat. (150).

**Remarks.** The original description and the USNM record did not mention the collection date including other information of paratypes. Paratypes came from 11 localities in the Philippines and Celebes, principally from southeast Luzon. USNM 149764 (Canimo Island, Camarines Norte), USNM 169502 (Ulugan Bay), and USNM 169505 (Jolo Island) specimens were collected during the Albatross Philippine Expedition in 1907–1910, but USNM records did not indicate if these are paratypes of *A.robusta*/ *O.cookii*. A radiograph and photograph of the holotype are available in the USNM record.

**(317) *Amiasealei* Fowler, 1918: 20, fig. 9**

= *Ostorhinchussealei* (Fowler, 1918).

**Holotype.**ANSP 47492 (66.0), Philippines.

**Paratypes.**ANSP 47493 [ex ANSP 47493–99] (7, 63.0–68.0), Philippines.

**Remarks.** The original description did not provide specific localities and collection dates. Type specimens are mixed based on ANSP’s record.

**(318) *Amiastriata* Smith & Radcliffe in Radcliffe, 1912: 437, pl. 35 (fig. 1)**

= *Jaydiastriata* (Smith & Radcliffe, 1912).

**Holotype.**USNM 68403 (89.0), Albatross station 5442, San Fernando Point Lighthouse, west of Luzon, 82 m, 10–11 May 1909.

**Paratypes.**USNM 93410 (11), same data as holotype.

**Remarks.** Radiographs of the holotype are available in the USNM record.

**(319) *Amiauninotata* Smith & Radcliffe in Radcliffe, 1912: 436, pl. 34 (fig. 3)**

= *Apogonichthyoidesuninotatus* (Smith & Radcliffe, 1912).

**Holotype.**USNM 70248 (55.0), Bisucay Island, Cuyo, Palawan, 2–5 m, 9 Apr. 1909.

**Paratypes.**USNM 339043 (1, 55.0), Tara Island, Coron, Palawan, 3–6 m, 15 Dec. 1908; Uncat. (1, 39.0), station 5179.

**Remarks.** The original description mentioned two paratypes without catalog number, but only one was found in the USNM record. The locality of the un-cataloged paratype could be in Romblon Lighthouse. Radiographs of the holotype are available in the USNM record.

**(320) *Amiaversicolor* Smith & Radcliffe in Radcliffe, 1911: 257, fig. 3**

= *Siphamiaversicolor* (Smith & Radcliffe, 1911).

**Holotype.**USNM 68401 (39.0), Cataingan Bay, Masbate Island.

**Paratypes.** FAKU [ex USNM 112269] (3), shores between Luzon, Sibutu Island, and northern Balabac Strait, Palawan, 18–309 m.

**Remarks.** The collection dates were not indicated in the original description.

**(321) *Amiawilsoni* Fowler, 1918: 22, fig. 10**

= *Ostorhinchuswilsoni* (Fowler, 1918).

**Holotype.**ANSP 47505 (72.0), Philippines.

**Remarks.** The original description did not provide a specific locality and collection date. Photos and radiographs are available in ANSP’s record.

**(322) *Apogonbryx* Fraser, 1998: 987, fig. 1**

= *Ostorhinchusbryx* (Fraser, 1998).

**Holotype.**CAS 34408 (42.5), south of Barrio Nonong Casto, Balayan Bay, Luzon, 146–155 m, 25 Jun. 1966.

**(323) *Apogonfranssedai* Allen, Kuiter & Randall, 1994: 33, fig. 5**

= *Ostorhinchusfranssedai* (Allen, Kuiter & Randall, 1994).

**Holotype.** NCIP 6118. Maumere Bay, Flores, Indonesia.

**Paratypes.**USNM 298668 (2, 31.5–35.5), Y’Ami Island, Batanes, 12–18 m, 25 Apr. 1987.

**Remarks.** Radiographs of the paratype are available in the USNM record.

**(324) *Apogongularis* Fraser & Lachner, 1984: 632, figs 1, 2**

= *Ostorhinchusgularis* (Fraser & Lachner, 1984).

**Holotype.**USNM 225672. Southern Red Sea, Yemen.

**Paratype.**USNM 225680 (1), southeast Tanguingui Island, between Negros and Masbate islands, 0–69.5 m, 5 Jun. 1978.

**(325) *Apogonmonospilus* Fraser, Randall & Allen, 2002: 182, fig. 1A–C**

= *Ostorhinchusmonospilus* (Fraser, Randall & Allen, 2002).

**Holotype.**USNM 357485 [ex BPBM 22140] (73.0), Dumaguete, Negros Island, 20 m, 27 Aug. 1977.

**Paratypes.** BPBM 28538 (5, 74.0–78.0), Dumaguete, Negros Island, 21 m, 3 Jun. 1981; CA-SU 69804 (1, 67.0), same locality as BPBM 28538, 9 Jun. 1931; USNM 169638 (4, 64.0–70.0), Albatross station 5136, Jolo Island, Sulu Archipelago, 41 m, 14 Feb. 1908.

**Remarks.** Radiographs of USNM 169638 are available in the USNM record.

**(326) *Apogonocellatus* Weber, 1913: 231**

= *Apogonichthysocellatus* (Weber, 1913).

**Syntypes.**BMNH 10022 [ex ZMA] (2, 25.0–39.0), Sanguisiapo and Nord-Ubian, Sulu Archipelago.

**Remarks.** The collection dates were not indicated in the original description.

**(327) *Apogonperlitus* Fraser & Lachner, 1985: 38, fig. 15**

= *Zoramiaperlita* (Fraser & Lachner, 1985).

**Holotype.** BPBM 15476. Kayangel Lagoon, Palau Island.

**Paratypes.**USNM 211850 [ex USNM 183742] (2), Tonquil Island, east of Gumila Reef, Sulu Archipelago, 14 Sep. 1909; USNM 211851 [ex USNM 205844] (14, 22.0–35.0), Makesi Island, Puerto Princesa, Palawan Island, 2–4 m, 5 Apr. 1909; USNM 183748 (1), Endeavor Strait, near Chase Head, Palawan Island, 22 Dec. 1908.

**Remarks.** Number of specimens mentioned on original description was different from the USNM records: USNM 211850 (20), USNM 183748 (25).

**(328) *Apogonpleuron* Fraser, 2005: 6, figs 1, 2**

= *Ostorhinchuspleuron* (Fraser, 2005).

**Holotype.**USNM 357488 (70.5), 11°28.70'N, 123°45.75'E, SE Tanguingui Island, Visayan Sea, 0–69 m, 5 Jun. 1978.

**Paratypes.**USNM 349175 [ex USNM 268252] (2, 65.0–70.0), 11°39.37'N, 123°38.27'E, NW of Tanguigui Island, Visayan Sea, 62 m, 20 May 1978; USNM 268253 (1, 57.0), Carigara Bay, Samar Sea, 50–70 m, 1980; USNM 357487 [ex USNM 332329] (6, 59.0–64.0), Sicogon Island, Visayan Sea, 0–47 m, 4 Jun. 1978; USNM 332331 (1, 104.0), 11°37.12'N, 123°54.75'E, southwest of Caduruan Point, Visayan Sea, 91 m, 6 Jun. 1978.

**Remarks.** The USNM record indicated 8 Jun. 1978 as the collection date for USNM 349175.

**(329) *Apogonposterofasciatus* Allen & Randall, 2002: 124, fig. 6**

**Holotype.** BPBM 15669. Tanavula Point, Florida Island, Solomon Islands.

**Paratype.**USNM 349197 (1, 49.0), Balicasag Island, Bohol Island, 0–24 m, 10 Jun. 1978.

**(330) *Apogonrufus* Randall & Fraser, 1999: 627, pl. 3**

= *Pristiconrufus* (Randall & Fraser, 1999).

**Holotype.** BPBM 9558. Malakal Pass, Palau Island.

**Paratypes.**USNM 349036 (1, 42.2), Puerto Princesa, Palawan Island, 12–18.5 m, 13 Jul. 1979; USNM 346183 (1, 64.6), off Lusaran Point, Guimaras Island, 14–20 m, 28 Sep. 1995.

**(331) *Apogonselas* Randall & Hayashi, 1990: 399, figs 1–2**

= *Ostorhinchusselas* (Randall & Hayashi, 1990).

**Holotype.** BPBM 32629. off Nagada Harbor, Madang Province, Papua New Guinea.

**Paratypes.** YCM-P 19451–53 (5, 22.1–35.2), El Nido, Palawan Island, 4 m, 20 Mar 1983.

**(332) *Apogonichthyslandoni* Herre, 1934: 40**

= *Foalandoni* (Herre, 1934).

**Holotype.**CAS-SU 29083, reef in Cebu Harbor, Cebu Island.

**Remarks.** The collection dates were not indicated in the original description. A photograph and a radiograph of the holotype are available in CAS’ record.

**(333) *Apogonichthysmelanopterus* Fowler & Bean, 1930: 17**

**Holotype.**ANSP 47491 (55.0), Philippines.

**Remarks.** Uncertain status in *Ostorhinchus* (Mabuchi et al. 2014). A replacement name for *Amiamelas* Fowler, 1918 which is pre-occupied by *Apogonmelas* Bleeker, 1848 in *Amia*. The original description did not mention a specific locality and collection date. Photos and radiographs are available in ANSP’s record.

**(334) *Apogonichthysmentalis* Evermann & Seale, 1907: 74, fig. 10**

= *Rhabdamiagracilis* (Bleeker, 1856).

**Holotype.**USNM 55905 (female, 63.5), Bacon, Sorgoson, Luzon.

**Paratype.**CAS-SU 2000 (1, 63.5), same data as holotype.

**Remarks.** The collection date was not indicated in the original description. Photographs of the holotype are available in the USNM record.

**(335) *Archamiabiguttata* Lachner, 1951: 588, pl. 18 (fig. D)**

= *Taeniamiabiguttata* (Lachner, 1951).

**Lectotype.**USNM 56156, Bacon, Sorsogon, Philippines.

**Paralectotypes.**USNM 360647 [ex 56156] (16), same data as lectotype; USNM 437454 (1) and USNM 112160 (39), Bolinao Bay, west of Luzon, 3–4 m, 10 May 1909; USNM 437457 (1) and USNM 112159 (33), stream at Maagnas, Lagonoy Gulf, east of Luzon, 17 Jun. 1909; USNM 112148 (4), Butauanan Island, east of Luzon, 3 m, 13 Jun. 1909; USNM 112149 (1), Rapurapu Island, east of Luzon, 3–5 m, 22 Jun. 1909; USNM 112150 (1), Dasol Bay, west coast of Luzon, 9 May 1909; USNM 112151 (1), Mahinog, Camiguin Island, 4–6 m, 3 Aug. 1909; USNM 112152 (1), Casogoran (Malhon Island), between Samar and Leyte, within vicinity of Surigao Strait, 3–5 m, 27 Jul. 1909; USNM 112158 (25), San Roque, between Samar and Leyte, within vicinity of Surigao Straight, 2–5 m, 29 Jul. 1909; USNM 112153 (1), Bisucay Islan, Cuyo, Palawan, 2–5 m, 9 Apr. 1909; USNM 112155 (1), west of Bancao-bancao Point, Puerto Princesa, Palawan, Island, 1–6 m, 5 Apr. 1909; USNM 112156 (3), Bolalo Bay, Malampaya Sound, Palawan Island, 21 Dec. 1908; USNM 112157 (5), Port Ciego, North Balabac Strait, Palawan, 4 m, 3 Jan. 1909; USNM 112154 (1), Batangas Market, Luzon, 7 Jun. 1908;

**Remarks.** Some of the specimens were based on *Amiamacropterus* Bleeker, 1875 and other references and specimens (Lachner 1951). A lectotype was designated by Gon and Randall (2003). A paralectotype (USNM 360647) was separated from the lectotype (Smithsonian Museum Archive 2024).

**(336) *Archamiadispilus* Lachner, 1951: 586, pl. 17 (fig. C)**

= *Taeniamiadispilus* (Lachner, 1951).

**Holotype.**USNM 112041. Soo Wan Bay, Taiwan.

**Paratypes.**USNM 112079 (1, 57.0), Generale Island, off northeast Mindanao, 4–6 m 9 May 1908; USNM 112080 (1, 63.0) Butauanan Island, Camarines Sur, Luzon, 3 m, 13 Jun. 1909; USNM 56157 (4, 36.5–58.0) and USNM 126368 (3, 54.0–63.0), Bacon, Sorsogon, Luzon, 1903.

**Remarks.**USNM 56157 was erroneously cited as 57147 in the original description (Smithsonian Museum Archive 2024).

**(337) *Cheilodipterusnigrotaeniatus* Smith & Radcliffe in Radcliffe, 1912: 442, pl. 37 (fig. 3)**

**Holotype.**USNM 70252 (80.0), coral reefs on the northeast shore of Sacol Island, east of Zamboanga, 4–5 m, 9 Sep. 1909.

**Paratypes.** Uncat. (2, 75.0–80.0), Tutu Bay, Jolo and Sacol Island, Sulu Archipelago.

**Remarks.** The original description provided no catalog number for paratypes. However, the USNM records indicate two catalog numbers (USNM 112306–07) have the same collection locality as the paratypes but did not indicate if these are type specimens. A radiograph of the holotype is available in the USNM record.

**(338) *Cheilodipteruszonatus* Smith & Radcliffe in Radcliffe, 1912: 443, pl. 38 (fig. 1)**

**Holotype.**USNM 70253 (66.0), Rita Island, Ulugan Bay, Puerto Princesa, Palawan Island, 6–12 m, 29 Dec. 1908.

**Paratype.** Uncat. (1, 60.0), Endeavor Strait, northwest of Palawan Island.

**Remarks.** The original description provided no catalog number for the paratype. However, the USNM records indicate one specimen (USNM 112308) with a similar collection locality to the paratype but did not indicate if this is a type specimen. Radiographs of the holotype are available in the USNM record.

**(339) *Foafo* Jordan & Seale, 1905: 779**

**Lectotype.**CAS-SU 9672 (38.1), Manila Bay, Cavite, Luzon.

**Remarks.** One of the syntypes was recently found and designated as the lectotype (Fraser and Randall 2011). The original description did not indicate the collection date.

**(340) *Gymnapogonjanus* Fraser, 2016: 433, figs 1–3**

**Holotype.**USNM 314651 (62.5), southeast of Sicogon Island, Visayan Sea, between Negros and Masbate islands, 24 m, 9 Jun. 1978.

**Paratypes.**USNM 150854 (1, 55.9), north of Nababuy Island, San Juanico Strait, east of Leyte Island, 1–3 m, 13 Apr. 1908.

**(341) *Henicichthysphilippinus* Herre, 1939: 300, pl. 34 (fig. 2)**

= *Gymnapogonphilippinus* (Herre, 1939).

**Holotype.**CAS-SU 34379 (31.0), tidepool at Nasugbu, Batangas, Luzon.

**Paratypes.**CAS-SU 34380 (3) and USNM 15164 [ex CAS-SU 34380] (1), tidepool at Nasugbu, Batangas, Luzon, 11 Dec. 1936; CAS-SU 34381 (4), tidepool in Dumaguete, Negros Island, 26 Dec. 1936.

**Remarks.** The original description or CAS did not indicate the collection date. A photograph and a radiograph of the holotype are available in the CAS record.

**(342) *Jaydiaerythrophthalma* Gon, Liao & Shao, 2015: 287, figs 2a, b, e**

**Holotype.**PNM 15192 [ex ASIZP 68202] (male, 50.5), 16°02.67'N, 121°55.32'E, west of Cape San Ildefonso, Aurora, Luzon, 194–203 m, 1 Jun. 2007.

**Paratypes.** ASIZP 68318 (2 females, 56.9–58.6), southwest of Cape San Ildefonso, Aurora, Luzon, 364–469 m, 2 Jun. 2007; CAS 236504 (2 females, 50.3–57.05), CAS 236506 (12, 24.9–48.5) and USNM 435707 [ex CAS 236506] (2 females, 34.9–41.2), between Luzon and Mindoro islands, 115–172 m, 1 Jun. 2011; SAIAB 200712 (2 female and male, 34.65–46.5), off north of Lubang Island, between Luzon and Mindoro island, 25 Mar. 2015.

**(343) *Mionorusmydrus* Jordan & Seale, 1905: 778, fig. 4**

**Holotype.**USNM 51946 (19.8), Bais, Negros Island, 1901.

**Remarks.** Uncertain as *Ostorhinchusmydrus* (Jordan & Seale, 1905) (Mabuchi et al. 2014). Further study is needed to verify its status.

**(344) *Neamiaarticycla* Fraser & Allen, 2006: 1, figs 1–3**

**Holotype.** AMS I.25121-005. Lizard Island, Great Barrier Reef, Queensland, Australia.

**Paratype.**USNM 370291 (1, 23.5), west of Mindoro Island, 30 m, 3 Jun. 2000.

**Remarks.** A photograph of the paratype is available in the USNM record.

**(345) *Neamiaoctospina* Smith & Radcliffe in Radcliffe, 1912: 441, pl. 36 (fig. 2)**

**Holotype.**USNM 70251 (37.0), Rasa Island, Mantaguin Bay, Narra, Palawan Island, 3 m, 1 Apr. 1909.

**Remarks.** Photographs of the holotype are available in the USNM record.

**(346) *Nectamiasimilis* Fraser, 2008: 37, figs 3C, 6A, 14, 18**

**Holotype.**USNM 213123. Kabaena Island, Tallabassi Bay, Sulawesi, Indonesia.

**Paratypes.** ROM 54320 (9, 16.0–50.0), Tanon Strait, Negros Island, 6–12 m, 19 May 1987.

**Remarks.** The original description mentioned the “Banda Sea” as the holotype, but the USNM record indicated “Tallabassi Bay” as type locality.

**(347) *Nectamiaviria* Fraser, 2008: 41, figs 3B, 6C, 7, 16, 18**

**Holotype.**USNM 319149 (57.4), Sangay Siapo Island, Tawi Tawi, Sulu Archipelago, 1.5–4.6 m, 20 May 1988.

**Paratypes.**USNM 344935 (136, 18.0–58.0), taken with holotype; ROM 54324 (14, 41.0–53.0 mm), Sumilon Island, Cebu; RW87-37, 4–8 m, 21 May 1987.

**Remarks.** In the USNM record, the holotype originally had 144 specimens; 7 were identified as *Apogonsavayensis* and re-cataloged as USNM 341621; 136 paratypes of *Apogonviria* and re-cataloged as USNM 344935. Radiographs and photographs of holotypes are available in the USNM record.

**(348) *Ostorhinchustricinctus* Allen & Erdmann, 2012: 1114, figs 1, 2, 4**

= *Apogontricinctus* (Allen & Erdmann, 2012).

**Holotype.** WAM P.33008-009 (male, 56.5), 11°07.82'N, 119°19.81'E, southwest corner of Entalula Island, Bacuit Bay, El Nido, Palawan, 22 m, 13 Jun. 2008.

**Paratypes.** WAM P.33008-036 (2, 31.7–36.0), taken with holotype.

**(349) *Pseudamiahayashii* Randall, Lachner & Fraser, 1985: 11, fig. 4**

**Holotype.** BPBM 15025. Ngargol Island, Palau Islands.

**Paratypes.**USNM 262691 (1, 56.3), Apo Island, Negros Island, 0–30.5 m, 6 Jun. 1978; USNM 262692 (1, 58.8), Siquijor Island, 0–6 m, 9 May 1978; USNM 262693–94 (2, 46.5–52.0), Pamilican Island, Bohol, 0–33.5 m, 12 Jun. 1978; USNM 268650 (4, 47.0–62.0), Bararin Island, Cuyo, Palawan, 0–13.7 m, 23 May 1978.

**(350) *Pseudamiazonata* Randall, Lachner & Fraser, 1985: 17, pl. 1 (fig. d); figs 5, 6**

**Holotype.** BPBM 28501 (female, 74.9), cave in 30.5 m, east off Tambuli Beach Hotel, Mactan Island, Cebu, 30 May 1981.

**Paratypes.** BM(NH) 1985.1.28.7 (1, 75.2) and BPBM 30350 (1, 38.7), taken with holotype.

**(351) *Siphamiaargentea* Lachner, 1953: 421, fig. 71**

**Holotype.**USNM 112042 (41.5), Albatross station 5356 (08°08.17'N, 117°19.25'E), Balabac Lighthouse, northern Balabac Strait, Palawan, 106 m, 5 Jan. 1909.

**Paratypes.**USNM 112089 (2, 20.5–28.0), Albatross station 5137 and USNM 112087 (1, 34.5), Albatross station 5145, Jolo Island, Sulu Archipelago, 142 m, 15 Feb 1908; USNM 112088 (3, 32.0–43.5), same locality as USNM 112089, 5 Mar. 1908; USNM 112090 (1, 25.5), Albatross station 5182, Antonio Island, off eastern Panay Island, 27 Mar. 1908.

**(352) *Siphamiacuprea* Lachner, 1953: 423, fig. 72**

= *Siphamiatubifer* Weber, 1909.

**Holotype.**USNM 112043 (male, 23.5), Cataingan Bay, Masbate Island, 18 Apr. 1908.

**Paratypes.**USNM 112093 (32, 16.0–29.0), taken with holotype; USNM 112091 (2, 24.0–27.0), Albatross station 5138, Jolo Island, Sulu Archipelago, 14 Feb. 1908; USNM 112092 (2, 24.0–28.0), Usada Island, south end of Jolo, Sulu Archipelago, 5 Mar. 1908; USNM 12098 (2, 27.0–29.0), Pangasinan Island, Jolo, Sulu Archipelago, 18 Feb. 1908; FMNH 62609 (2), same locality as USNM 12098, 13 Feb. 1908.

**(353) *Siphamiaelongata* Lachner, 1953: 419, fig. 70**

**Holotype.**USNM 112045 (female, 36.0), Canmahala Bay, Ragay Gulf, Luzon, 11 Mar. 1909.

**Paratypes.**USNM 112099 (32, 16.0–36.0), same data as holotype; USNM 112095 (1, 24.0), Albatross station 5169, Sibutu Island, Sulu Archipelago, 27 Feb. 1908; USNM 112096 (1, 24.0) and USNM 112097 (1, 32.0), Albatross station 5142, Jolo Island, Sulu Archipelago, 15 Feb. 1908; USNM 112098 (1, 31.0), Albatross station 5342, Tagolo Point Lighthouse, northern Mindanao, 9 Aug. 1909.

**(354) *Siphamiaovalis* Lachner, 1953: 427, fig. 74**

= *Siphamiatubifer* Weber, 1909.

**Holotype.**USNM 112044 (28.0), Albatross station 5140 (06°08.76'N, 121°03.00'E), Jolo Lighthouse, Sulu Archipelago, 139 m, 14 Feb. 1908.

**Paratypes.**USNM 112101 (3, 23.5–28.0), taken with holotype; USNM 112100 (2, 29.0–30.5), Albatross station 5149, Sirun Island, Siasi, Sulu Archipelago, 18 m, 18 Feb. 1908.

#### ﻿﻿GOBIIFORMES (58)

##### Family Eleotridae (278)

**(355) *Asterropteryxeveretti* Boulenger, 1895: 186**

= *Hypseleotriseveretti* (Boulenger, 1895).

**Syntypes.**BMNH 1894.6.30.172–177 (6), Palawan Island.

**Other catalog number.** NHMUK:ecatalogue:3119296.

**Remarks.** The original description mentioned a specimen measuring 65.0 TL but not the collection date.

**(356) *Borodafrancoi* Roxas & Ablan, 1940: 303, pl. 2**

= *Bunakagyrinoides* (Bleeker, 1853).

**Holotype.**BSM 31947, Dagupan River, Pangasinan, Luzon.

**Paratype.** Uncat. (1).

**Remarks.** The original description provided no information on collection date and catalog number for the paratype. All type specimens were lost (Kottelat 2013).

**(357) *Bunakapinguis* Herre, 1927c: 61, pl. 27 (fig. 2)**

= *Bunakagyrinoides* (Bleeker, 1853).

**Holotype.** BSMP 10594 (male, 170.0) Dumaguete River, Negros Island, 8 Mar. 1922.

**Paratypes.** BSMP (female, 167.0), Pulangi River near Reina Regente, Cotabato, Mindanao; BSMP (male, 228.0), Abra River, near Bangued, Abra; BSMP (female, ~ 228.0), Lapid Lapid River, Tawi Tawi, Sulu Archipelago, 26 Jul. 1924.

**Remarks.** All type specimens were lost but cataloged by Koumans (1940) (Kottelat 2013).

**(358) *Bunakasticta* Herre, 1942a: 119**

= *Bunakagyrinoides* (Bleeker, 1853).

**Holotype.**CAS-SU 36534, Tagaloan River, Misamis Oriental, Mindanao, 21 Aug. 1940.

**Remarks.** A photograph and a radiograph of the holotype are available in CAS’s record.

**(359) Eleotris (Giuris) laglaizei Sauvage, 1880: 15 [15**]

= *Giurislaglaizei* (Sauvage, 1880).

**Holotype.**MNHN A-1690 (135.0), Manila, Luzon, 1876.

**(360) *Hypseleotrisagilis* Herre, 1927c: 38, pl. 2 (fig. 3)**

= *Giurislaglaizei* (Sauvage, 1880).

**Holotype.** BSMP 10143, a creek flowing into Lake Mainit, Surigao, Mindanao.

**Paratypes.** BSMP (13), same data as holotype.

**Remarks.** The collection dates were not indicated in the original description. All type specimens were lost (Kottelat 2013).

**(361) *Hypseleotrisbipartita* Herre, 1927c: 39, pl. 3 (fig. 1)**

**Holotype.** BSMP (male, 33.5), creek at Barrio Puru, Lagaspi, Albay, Luzon, 3 Feb. 1926.

**Paratypes.** BSMP (27 males, 24.0–33.0 + 8 females, 22.0–37.0), Rawis River, Legaspi, Albay, Luzon, 3 Feb. 1926.

**Remarks.** Questionably a synonym of *Hypseleotrisleuciscus* (Bleeker, 1853) (Kottelat 2013); *Species inquirenda* (Keith and Mennesson 2023). All type specimens were lost (Kottelat 2013).

**(362) *Hypseleotrispangel* Herre, 1927c: 42, pl. 3 (fig. 2)**

**Syntypes.** BSMP 207 (20, 32.0–47.0), Cavite, Luzon.

**Remarks.***Species inquirenda* in *Hypseleotris* (Kottelat 2013; Parenti 2021; Keith and Mennesson 2023); Jamandre (2023) considered it as valid. The collection date was not indicated in the original description. All type specimens were lost (Kottelat 2013).

**(363) *Hypseleotrisquisumbingi* Roxas & Ablan, 1940: 301, pl. 1**

**Holotype.**BSM 31952, Lingayen Gulf (via San Fernando Market), La Union, Luzon.

**Paratypes.**BSM (4).

**Remarks.***Species inquirenda* in *Hypseleotris* (Kottelat 2013; Keith and Mennesson 2023). Jamandre (2023) consider it as valid. The original description did not indicate the collection date. All type specimens were lost (Kottelat 2013).

**(364) *Luzoneleotrisnasugbua* Herre, 1938: 60, fig. 1**

= *Xenisthmuspolyzonatus* (Klunzinger, 1871).

**Holotype.**CAS-SU 32974, Tide pool at Nasugbu, Batangas, Luzon, 11 Dec. 1936.

**Remarks.** The original description did not indicate the collection date.

##### Family Butidae (279)

**(365) *Borodaalbo-oculata* Herre, 1927c: 58**

= *Oxyeleotrisalbooculata* (Herre, 1927).

**Holotype.** BSMP 10577 (female, 86.0), small freshwater stream at Taytay, Palawan Island, May 1913.

**Paratypes.** BSMP (12, 22.0–60.0), same data as holotype.

**Remarks.** All type specimens were lost (Kottelat 2013).

**(366) *Borodaexpatria* Herre, 1927c: 59, pl. 5 (fig. 1)**

= *Bostrychusexpatria* (Herre, 1927).

**Holotype.** BSMP 11468 (76.0), probably from Lake Manguao, Taytay, Palawan Island.

**Paratypes.** BSMP (5, 65.0–144.0), same data as holotype.

**Remarks.** The collection date was not indicated in the original description. All type specimens were lost (Kottelat 2013).

**(367) *Butisleucurus* Jordan & Seale, 1905: 794, fig. 13**

= *Butisamboinensis* (Bleeker, 1853).

**Holotype.**USNM 51953 (73.0), southern Negros Island, 1901.

**Remarks.** Radiographs and photographs of holotypes are available in the USNM record.

**(368) *Paloapolylepis* Herre, 1927c: 56, pl. 4 (fig. 3)**

= *Odonteleotrismacrodon* (Bleeker, 1853).

**Holotype.** BSMP (68.0), Iloilo Market, Panay Island.

**Paratype.** BSMP (1, 73.0), San Jose, Antique, Panay Island.

**Remarks.** The collection date was not indicated in the original description. All type specimens were lost (Kottelat 2013).

**(369) *Paloavilladolidi* Roxas & Ablan, 1940: 304, pl. 3**

**Holotype.**BSM 31946, Dagupan, Pangasinan, Luzon.

**Remarks.** The collection date was not indicated in the original description. The type specimen was lost (Kottelat 2013).

**(370) *Parviparmastraminea* Herre, 1927c: 82, pl. 6 (fig. 2)**

**Holotype.** BSMP (65.0), Saug River, southeast of Cotabato, Mindanao.

**Remarks.** The collection date was not indicated in the original description. The type specimen was lost (Kottelat 2013).

##### Family Oxudercidae (281)

**(371) *Apocryptodonlomboyi* Ablan, 1940: 373, pl. 1**

= *Apocryptodonmadurensis* (Bleeker, 1849).

**Holotype.** BSMP 31128, Dagupan, Pangasinan, Luzon.

**Paratypes.** BSMP (26, 50.0–65.0), same data as holotype.

**Remarks.** The collection dates were not indicated in the original description. All type specimens were lost (Kottelat 2013).

**(372) *Apocryptodonmontalbani* Herre, 1927c: 277, pl. 22 (fig. 2)**

= *Apocryptodonmadurensis* (Bleeker, 1849).

**Holotype.** BSMP 12390 (44.0), Zarraga, Iloilo, Panay Island, 15 Aug. 1925.

**Remarks.** The type specimen was lost (Kottelat 2013).

**(373) *Apocryptodonsealei* Herre, 1927c: 278**

= *Apocryptodonmadurensis* (Bleeker, 1849).

**Holotype.** BSMP 176 (52.0), Manila market, Luzon, Jun. 1908.

**Remarks.** The type specimen was lost (Kottelat 2013).

**(374) *Apocryptodontaylori* Herre, 1927c: 279, pl. 22 (fig. 3)**

= *Apocryptodonmadurensis* (Bleeker, 1849).

**Holotype.** BSMP 12067 (34.0), Odiongan, Tablas Island, Romblon.

**Remarks.** The collection dates were not indicated in the original description. The type specimen was lost (Kottelat 2013).

**(375) *Brachyamblyopusolivaceus* Herre, 1927c: 329, pl. 25 (fig. 3)**

= *Caragobiusurolepis* (Bleeker, 1852).

**Syntypes.** BSMP 13024 (7, 41.0–55.0), La Libertad, Negros Island.

**Remarks.** The collection dates were not indicated in the original description. The type specimen was lost (Kottelat 2013).

**(376) *Brachygobiusaggregatus* Herre, 1940: 361, pl. 4**

**Lectotype.**CAS-SU 32990 (male, 11.5), Dumaguete, Negros Island, 26 Dec. 1936.

**Paralectotypes.**CAS-SU 18082 (2 females, 13.5–15.0) and CAS-SU 32991 (124, 11.0–44.0), same data as holotype; BMNH 1938.12.1.193–198 (6), same locality as holotype.

**Other catalog number.**BMNH 1938.12.1.193–198 (NHMUK:ecatalogue:2517101).

**Remarks.** A lectotype was designated by Böhlke (1953). A photograph and a radiograph of the holotype are available in the CAS record.

**(377) *Caecogobiuscryptophthalmus* Berti & Ercolini, 1991: 130, figs 1–5**

**Holotype.** MSNVR 1262 (61.0), Calbiga Cave system, Samar Island, Jan. – Feb. 1987.

**Paratypes.** MSNVR 1262a (1, 57.5), MSNVR 1262b (female, 42.5) and ZSM 27189 (1, 58.5), same data as holotype.

**(378) *Caecogobiuspersonatus* Larson & Husana, 2018: [2**]

**Holotype.**PNM 15353 [ex ZRC 56329] (49.0, male), Ugnope Cave system, Mindanao.

**Paratypes.** ZRC 56329 (1, 49.0, male); PNM 15354 (1, 42.0, male); AMS I.47860-001 (1, 39.0, male); same collecting data as holotype.

**(379) *Calamianaillota* Larson, 1999: 260, figs 1–4**

= *Eugnathogobiusillotus* (Larson, 1999).

**Holotype.** ZRC 39268. Sungei Buloh, Singapore.

**Paratypes.**USNM 316045 (1, 27.0), Sorsogon Fish Market, Sorsogon Bay, Luzon, 29–30 Apr. 1976; CAS 38650 (1, 36.5), Capiz, Panay Island, 3 Aug. 1940.

**(380) *Calamianamagnoris* Herre, 1945c: 80**

= *Eugnathogobiuskabilia* (Herre, 1940).

**Holotype.**CAS-SU 39881 (female, 30.0), Coron, Busuanga Island, Palawan, 1 Jul. 1940.

**Remarks.***Calamianamagnoris* Herre, 1945 and *Ganthogobiusaliceae* Smith, 1945 were published in the same year but precedence was given to *C.magnoris* (published 3 Jun. 1945) over *G.aliceae* (published 13 Nov. 1945) (Larson 2009). A photograph and a radiograph of the holotype are available in the CAS record.

**(381) *Caragobiustyphlops* Smith & Seale, 1906: 81**

= *Caragobiusurolepis* (Bleeker, 1852).

**Holotype.**USNM 55619 (57.2), Rio Grande River, Mindanao, 255 m, Oct. 1903.

**Paratypes.**CAS-SU 20008 (1) and USNM 126384 [ex USBF 1485] (1), (50.8–47.2), same data as holotype.

**Remarks.** The original description mentioned four paratypes.

**(382) *Gnatholepisdavaoensis* Seale, 1910: 537**

= *Gnatholepisophthalmotaenia* (Bleeker, 1854).

**Holotype.** BSMP 3858 (45.0), Samal Island, Davao Gulf, Mindanao.

**Neotype.** BPBM 18670, Southern end of Hou Pi Hoo, Taiwan, 0.0.2 m.

**Remarks.** The holotype was presumed destroyed and a neotype was designated by Randall and Greenfield (2001). The original description or USNM record did not indicate the collection date.

**(383) *Gnatholepisgemmeus* Herre, 1927c: 135, pl. 9 (fig. 3)**

= *Gnatholepisophthalmotaenia* (Bleeker, 1854).

**Holotype.** BSMP, Samal Island, Davao Gulf, Mindanao.

**Paratypes.** BSMP (24, 25.0–45.0).

**Neotype.** BPBM 18670, Southern end of Hou Pi Hoo, Taiwan, 0.0.2 m.

**Remarks.** The original description or USNM record did not indicate the collection date.

All original types have been lost (Kottelat 2013) and a neotype was designated by Randall and Greenfield (2001).

**(384) *Gnatholepisturneri* Roxas & Ablan, 1940: 78, pl. 1**

**Holotype.** BSMP 41993, Lingayen Gulf, Pangasinan Province, Luzon.

**Remarks.** The collection date was not indicated in the original description. Larson and Buckle (2012) revised the genus and commented that it is possibly a species of *Acentrogobius*. The type specimen was presumed destroyed, and further collection of specimens is needed to confirm its status.

**(385) *Gnatholepisvolcanus* Herre, 1927c: 131**

**Holotype.** BSMP 10569 (101.0), Taal Lake, Batangas, Luzon.

**Remarks.***Species inquirenda* in *Exyrias* Jordan & Seale, 1906 (Kottelat 2013; Larson and Jaafar 2022). Larson and Wright (2003) though it might be an *Exyrias*, and Larson and Buckle (2012) added this to their table of nominal species. All type specimens were lost (Kottelat 2013). Jamandre (2023) consider it valid, but collection of additional specimens and further study are needed to confirm its status.

**(386) *Gobiusdispar* Peters, 1868: 264**

= *Redigobiusdispar* (Peters, 1868).

**Lectotype.** ZMB 6702 (male, 42.0), Luzon.

**Paralectoypes.** ZMB 33908 (6, 35.5–38.0), ZMB 6703 (4, 30.0–36.0), ZMB 6700 (11, 40.0–41.0), ZMB 6702 (8), Lake Batu; ZMB 6705 (8, 31.0–34.0), Kalabos stream; ZMB 6737–38 (2), Lake Buhi; BMNH 1868.7.10.12–15 (4, 33.0–44.0). All from Luzon.

**Other catalog number.**BMNH 1868.7.10.12–15 (NHMUK:ecatalogue:3102247).

**Remarks.** The collection date was not indicated in the original description. Type specimens were previously designated as syntypes. The lectotype and paralectotypes were designated by Larson (2010).

**(387) *Gobiuslacrymosus* Peters, 1868: 265**

= *Stenogobiusophthalmoporus* (Bleeker, 1853).

**Syntypes.**BMNH 1868.7.10.10–11 (2) and BMNH 1870.3.29.4 (1), Luzon; MNHN 0000-6159 (2), Polilio Island, east of Luzon, 1869; ZMB 6679 (4) and ZMB 6680 (1), Quingoa River, Bulacan, Luzon.

**Other catalog number.**BMNH 1868.7.10.10–11 (NHMUK:ecatalogue:3102245), BMNH 1868.7.10.10–11 (NHMUK:ecatalogue:3103152).

**Remarks.** The original description did not designate a holotype and mentioned that the largest specimen has 135.0 TL. Several specimens were collected from Quionga River in Bulacan, Luzon. However, based on MNHN record, MNHN 0000-6159 was collected in Polilio Island, east of Luzon in 1869.

**(388) *Gobiuslitturatus* Steindachner, 1861: 289, pl. 1 (figs 4, 5)**

= *Awaouslitturatus* (Steindachner, 1861).

**Holotype.** NMW 29507, Philippines.

**Remarks.** The original description did not provide a specific locality and collection date.

**(389) *Gobiussternbergi* Smith, 1902a: 169, fig.**

= *Redigobiusdispar* (Peters, 1868).

**Syntypes.**USNM 50536 (3, 20.0–27.0), Lake Buhi, Camarines Sur, Luzon, 5 Jul 1901.

**Remarks.** The original description mentioned six specimens. Koumans (1940) provided a short description and taxonomic remark. Radiographs of the syntypes are available in the USNM record.

**(390) *Lentipesmindanaoensis* Chen, 2004: 38, figs 1–2**

**Holotype.** NMMB P 4821 (46.0), small creeks east of Mindanao, Feb. 2003.

**(391) *Lentipespalawanirufus* Maeda & Palla, 2021: 21, figs 20–22**

**Holotype.** NSMT-P 136936 (male, 44.8), Estrella Falls, Narra, Palawan Island, 13 May 2016.

**Paratypes.** NSMT-P 136937 (female, 49.2), URM-P 48910–12 (3 males, 25.8–45.9), WPU-PPC-P 36–38 (3 males, 39.4–46.3), same data as holotype; URM-P 48915, URM-P 48917–19 (3 males, 33.2–46.3 + 1 female, 45.2) and WPU-PPC-P 42–48 (5 males, 33.7–47.2), same locality as holotype, 29 May 2018; URM-P 48913 (male, 31.4) and WPU-PPC-P 40–41 (2 males, 24.5–30.7), Olanguan Falls, Puerto Princesa, Palawan Island, 16 May 2016.

**(392) *Microsicydiumatro-purpureum* Herre, 1927c: 296**

= *Stiphodonatropurpureus* (Herre, 1927).

**Neotype.** ZRC 38392 (male, 33.1), Leyte Island, 29 Jun. 1993.

**Remarks.**Koumans (1940) noted no specimens in the bottle. The holotype and BSMP 13234 (3) from Irig River, Philippines were destroyed and the neotype was designated by Watson and Kottelat (1995).

**(393) *Microsicydiumformosum* Herre, 1927c: 297, pl. 23 (fig. 3)**

= *Stiphodonatropurpureus* (Herre, 1927).

**Holotype.** BSMP 12443 (40.0), Titunod River, Kolambugan, Lanao del Norte, Mindanao, 26 May 1921.

**Remarks.**Koumans (1940) provided a taxonomic remark. The type specimen was lost (Kottelat 2013).

**(394) *Microsicydiumpulchellum* Herre, 1927c: 299, pl. 23 (fig. 4)**

= *Stiphodonpulchellus* (Herre, 1927).

**Neotype.**CAS-SU 26360 (male, 50.5), Tanjay River, 20–30 km northwest of Dumaguete, Negros Island, 15 Jun 1931.

**Remarks.** The original type specimens were dried up, broken, and too bad for comparison with the description (Koumans, 1940). The neotype was designated by Maeda et al. (2012). A photograph and a radiograph of the neotype are available in the CAS record.

**(395) *Mirogobiuslacustris* Herre, 1927c: 93**

= *Gobiopteruslacustris* (Herre, 1927).

**Syntypes.** BSMP (many), ZMA 115.798 (5), CAS-SU 15490 (79) and MNHN 1932-0206 (5), Laguna de Bay,, Luzon.

**Remarks.** The original BSMP specimens were lost (Kottelat 2013). Photographs and radiographs are available in the CAS record.

**(396) *Mirogobiusstellatus* Herre, 1927c: 92, pl. 6 (fig. 4)**

= *Gobiopterusstellatus* (Herre, 1927).

**Syntypes.** BSMP 13054 (110, 10.2–21.0), mountain lake beside Sitio Lanigay, Polangui, Albay, 25 Jan. 1926.

**Remarks.**Koumans (1940) provided a taxonomic remark. All type specimens were lost (Kottelat 2013).

**(397) *Mistichthysluzonensis* Smith, 1902b: 30**

**Syntypes.**USNM 50303 (18) and USNM 50304 (15), Lake Buhi, Camarines Sur, Luzon, 5 Jul. 1901.

**Remarks.**Koumans (1940) provided a taxonomic remark. One specimen of USNM 50304 was sent to Dr. Smith. Radiographs of the syntypes are available in the USNM record.

**(398) *Mistichthysmindanensis* Herre, 1944b: 109**

= *Gobiopterusmindanensis* (Herre, 1944).

**Syntypes.**CAS-SU 39882 (9 males, 9.5–10.5) and CAS-SU 39883 (27 females, 9.5–13.0) puddles around the base of nipa palms near the Fisheries Station at Zamboanga, Mindanao, 10 Sep. 1940.

**Remarks.** The original description did not designate a holotype, and the type specimens have since been mixed. Thus, the entire lot was considered syntypes (Böhlke 1953). Photographs and radiographs of the syntypes are available in the CAS record.

**(399) *Mistichthyspanayensis* Herre, 1944b: 108**

= *Gobiopteruspanayensis* (Herre, 1944).

**Lectotype.**CAS-SU 36819 (male, 11.0), saltwater nipa swap, near Capiz, Panay Island, 3 Aug 1940.

**Paralectotypes.**CAS-SU 36820 (73 males, 9.0–11.5), CAS-SU 36821 (105 females, 8.5–12.5), and USNM 123653 (6 males + 6 females), same locality as the lectotype.

**Remarks.** The original description mentioned a female (12.0) and male (11.0) type specimens, together with other paratypes (73 males and 103 females). The male type is separated in the collection (CAS-SU 36819), while the female type is mixed with the other females (CAS-SU 36821). The male type specimen was designated as lectotype and other specimens as paralectotypes by Böhlke (1953). A photograph and a radiograph of the holotype are available in the CAS record.

**(400) *Mugilogobiusluzonensis* Roxas & Ablan, 1940: 307, pl. 6**

**Holotype.** BSMP 31950, Luzon Island.

**Remarks.** The original description did not indicate the collection date. Larson (2001) suggested it may be a *Mugilogobius*, but could also be a *Pseudogobius* (Larson and Hammer 2021). The type specimen was lost, presumed destroyed during the World War II (Kottelat 2013).

**(401) *Pandakapusilla* Herre, 1927c: 197, pl. 15 (figs 1, 2)**

**Syntypes.** BSMP 12806 (27, 13.0–16.5), nearshore at Sitankai, Tawi Tawi Group, Sulu Archipelago, 11 Jul. 1908.

**Remarks.** All type specimens were lost (Kottelat 2013).

**(402) *Pandakapygmaea* Herre, 1927c: 198, pl. 15 (fig. 3)**

**Lectotype.**CAS-SU 23761 (9.8), Philippines (possibly Malabon), 1907.

**Paralectotypes.**CAS-SU 18143 (8), same data as lectotype.

**Remarks.** Herre did not select a type specimen in his original description. All BSMP type specimens were presumed lost, leaving only SU specimens remaining. The lectotype and paralectotypes were selected by Böhlke (1953). A photograph and a radiograph of the holotype are available in the CAS record.

**(403) Parapocryptes (Paeneapocryptes) mindanensis Herre, 1927c: 262, pl. 20 (fig. 4)**

= *Oxyurichthysnotonema* (Weber, 1909).

**Holotype.** BSMP 13226 (33.0), south of Cotabato, Mindanao.

**Remarks.** The original description did not indicate the collection date. Koumans (1940) provided a taxonomic remark. All type specimens were presumed destroyed.

**(404) *Rhinogobiuscarpenteri* Seale, 1910: 535**

**Neotype.** KIZ 2016003044 (male, 47.3), 16°27.97'N, 120°33.86'E, Wangan River, Balili River drainage, La Trinidad, Benguet, 1245 m elevation, 12–13 Aug. 2016.

**Remarks.** All original type specimens were lost (Kottelat 2013), and a neotype was designated by Endruweit (2017).

**(405) *Rhinogobiusestrellae* Maeda, Kunishima & Palla in Maeda, Shinzato, Koyanagi, Kunishima, 2021: 84, figs 1a–d, 2, 4, 5**

**Holotype.** NSMT-P 140091 (male, 40.6), Estrella Falls, Narra, Palawan Island, 13 May 2016.

**Paratypes.** NSMT-P 140092 (female, 38.9), URM-P 49295–301 (1 male, 40.7 + 6 females, 36.1–40.9), WPU-PPC-P 50–54 (1 male, 37.5 + 4 females, 35.5–41.1), same data as holotype; URM-P 49303–06 (2 males, 37.8–39.6 + 2 females, 36.4–45.3) and WPU-PPC-P 55–59 (3 males, 37.3–38.4 + 2 females, 33.2–37.8), same locality as holotype, 28 May 2018; URM-P 49302 (female, 43.0), creek in front of the Estrella Village, Barangay Hall, 28 May 2018; all samples from Narra, Palawan Island.

**(406) *Rhinogobiusschultzei* Herre, 1927c: 185**

**Syntypes.** BSMP 12407 (1) and BSMP 26833 (1) (28.0–37.0), River at Fabrica, Negros Island.

**Remarks.** The original description did not indicate the collection date. All type specimens were lost (Kottelat 2013). Larson (2001) suggested it could be a *Mugilogobius* or *Stigmatogobius*, but could also be a *Pseudogobius* (Suzuki et al. 2017; Larson and Hammer 2021). Collection of specimens and further study is needed to confirm its status.

**(407) *Rhinogobiustandikan* Maeda, Kobayashi & Palla in Maeda, Shinzato, Koyanagi, Kunishima, 2021: 89, figs 1e–h, 6–8**

**Holotype.** NSMT-P 140093 (male, 43.6), Cayulo River, Puerto Princesa, Palawan Island, 1 Jun. 2018.

**Paratypes.**WPU-PPC-P 65–69 (3 males, 43.6–6.1 + 2 females, 39.6–41.3), same data as holotype; URM-P 49316–23 (2 males, 40.8–44.8 + 6 females, 35.6–45.2), NSMT-P 140094 (female, 45.7), URM-P 49307–315 (7 males, 36.1–46.9 + 2 females, 37.3–38.9), and WPU-PPC-P 60–64 (5 females, 30.8–36.5), same locality as holotype, 31 May 2018.

**(408) *Sicyopteruscrassus* Herre, 1927c: 307, pl. 24 (fig. 2)**

= *Sicyopteruscynocephalus* (Valenciennes, 1837).

**Syntypes.** BSMP 10619 (1) and BSMP 26974–77 (4, 97–128.0), Craan River, south of Cotabato, Mindanao, 27 Mar. 1921.

**Remarks.**Koumans (1940) provided a taxonomic remark. All type specimens were lost (Kottelat 2013).

**(409) *Sicyopterusextraneus* Herre, 1927c: 311, fig. 4**

= *Sicyopteruslagocephalus* (Pallas, 1770).

**Syntypes.** BSMP 10588 (1) and BSMP 26884–87 (4, 60.5–74.0), Cabalian, Leyte Island, 28 May 1921.

**Remarks.**Koumans (1940) provided a taxonomic remark. All type specimens were lost (Kottelat 2013).

**(410) *Sicyopterusfuliag* Herre, 1927c: 309, fig. 3**

= *Sicyopteruscynocephalus* (Valenciennes, 1837).

**Holotype.** BSMP 10619, Panacanawan River, Lamug, Peña Blanca, Cagayan, Luzon, 17 May 1923.

**Paratypes.** BSMP 26874–77 (6, 72.0–105.0).

**Remarks.**Koumans (1940) provided a short description and taxonomic remark. All type specimens were lost (Kottelat 2013).

**(411) *Sicyopteruslacrymosus* Herre, 1927c: 303, pl. 24 (fig. 1)**

= *Sicyopteruslongifilis* de Beaufort, 1912.

**Syntypes.** BSMP 12995 (1) and BSMP 26205–12 (8, 47.0–68.0) Ba Kalaba (Abra River, Bangued, Abra), 15 Nov. 1925; BSMP 10618 (1, 58.0) Titunod River, Kolambugan, Lanao, Mindanao, 26 May 1921.

**Remarks.** Herre mentioned 19 co-types measuring 50.0–63.0, but Koumans (1940) mentioned only 10. Koumans (1940) provided a taxonomic remark. All type specimens were lost (Kottelat 2013).

**(412) *Sicyopteruspanayensis* Herre, 1927c: 313, fig. 5**

= *Sicyopteruslongifilis* de Beaufort, 1912.

**Lectotype.** BSMP 13137, San Jose, Antique, Panay Island.

**Paralectotypes.** BSMP 26920–24 (5, 86.0–91.0 TL), same data as lectolotype.

**Remarks.** The original description did not indicate the collection date. Koumans (1940) provided taxonomic remark and designated the lecto- and paralectotypes. All type specimens were lost (Kottelat 2013).

**(413) *Sicyopusauxilimentus* Watson & Kottelat, 1994: 358, fig. 5**

**Holotype.** ZRC 38286/CMK 10047 (male, 29.2), Lagu Lagu creek ~ 2 km from the sea, the south margin of Visayan State College of Agriculture (now Visayas State University), ~ 7 km north of Baybay, Leyte Island, 23 Mar. 1991.

**Paratype.** ZRC 38287 [ex. CMK 10015] (1 female, 25.9), same locality as holotype, 11 Jul. 1993.

**(414) *Sicyopuscebuensis* Chen & Shao, 1998: 98, figs 1–3**

= *Sicyopusauxilimentus* Watson & Kottelat, 1994.

**Holotype.** ASIZP 057825 (male, 37.5), Uling brook of the Naga River, Cebu Island, 17 Nov. 1997.

**Paratype.** ASIZP 057826 (female, 39.1), same data as holotype.

**(415) Stenogobius (Insularigobius) kyphosus Watson, 1991: 639, fig. 29a, b**

**Holotype.**USNM 99878 (male, 90.4), Mahinog River, Camiguin Island, 3 Aug. 1909.

**Paratypes.** AMS 1.25439-001 (1 male + 1 female, 79.5–82.9), ANSP 156992 [ex USNM 99878] (1 male + 1 female, 72.2–80.2), ROM 48510 (3 females, 54.2–87.7) and USNM 274590 (1 male + 1 female, 80.2–83.2), same data as holotype; USNM 99929 (1 female + 1 juvenile, 16.8–45.8), Baganga Bay, Mindanao, 13 May 1909; USNM 99930 (female, 52.2), Nonucan River near Camp Overton, northern Mindanao, 6 Aug. 1909; USNM 120323 (female, 53.9), Malaga River, Leyte Island, 30 Jul 1909.

**Remarks.** Two specimens mixed with holotype were removed and re-cataloged as USNM 274590 and became paratypes (Smithsonian Museum Archive 2024). A radiograph of the holotype is available in the USNM record. No specimens are available for ANSP 156992 based on ANSP’s record.

**(416) *Stigmatogobiuselegans* Larson, 2005: 354, figs 5–6**

**Holotype.**USNM 314469 (female, 37.0), dry seasonal pool, main channel of Imurung River, Barrio San Miguel, Cagayan, Luzon, 0–2 m, 2 May 1989.

**Paratypes.**USNM 376152 (29, 6.0–38.0), NTM 15807-001 (2, 36.0–41.0), same data as holotype; USNM 314213 (41, 5.5–37.0), main channel of Imurung River, Barrio San Miguel, Bagao, Cagayan, Luzon, 2 May 1989.

**Remarks.** The holotype was originally mixed with other specimens, eight of which were exchanged to Doug Hoese of the Australian Museum, Sydney, two specimens were donated to the Northern Territory Museum, and the 29 specimens were removed to USNM 376152 and became paratypes.

**(417) *Stiphodonolivaceus* Watson & Kottelat, 1995: 8, figs 4–7**

= *Stiphodonpulchellus* (Herre, 1927).

**Holotype.** ZRC 38396 (male, 50.2), Hilosig Creek, 1.3 km north of Mahaplag junction, Leyte Island, 6 Jul. 1993.

**Paratypes.** ZRC 38397 (1 male, 49.8; 2 females 24.7–53.5), CMK 9986 (2 males, 36.0–42.7; 4 females 23.2–45.3), same data as holotype.

**(418) *Stiphodonpalawanensis* Maeda & Palla, 2015: 382, figs 1, 3–5**

**Holotype.**WPU-PPC-P 5 (male, 62.2), Balsahan stream in the Iwahig Prison and Penal Farm, Puerto Princesa, Palawan Island, 18 May 2015.

**Paretypes.**WPU-PPC-P 6–9 (1 male, 59.4 + 3 females, 57.5–62.1), same data as holotype; CMK 11966 (2 males, 37.3–43.1 + 8 females, 28.5–47.2), Malatgao River, Narra, 29 Sep. 1994; CMK 11974 (3 females, 41.9–48.5), Estrella Falls, Narra, 29 Sep. 1994; NSMT-P 45091–92, and NSMT-P 45094 (2 males, 27.7–38.8 + 1 female, 36.6), Iwahig River, Puerto Princesa, 13 Nov. 1988; URM-P 31440 (2 females, 38.6–40.7), Iraan River, 5 Aug. 1985; URM-P 31438 (female, 27.0), Nagsagoiri River, 9 Aug. 1985; URM-P 31439 (6 males, 34.1–36.8 + 7 females, 32.4–36.2), Papait River, 9 Aug. 1985; URM-P 31441 (6 males, 31.3–41.4 + 3 females, 30.7–39.1), Tagbariri, 9 Aug. 1985; URM-P 48659–62 (2 males, 46.2–51.1 + 2 females, 41.1–47.2), URM-P 48663–66 (2 males, 59.7–63.9 + 2 females, 58.5–63.6) and WPU-PPC-P 2–4 (2 males, 52.6–59.2 + 1 female, 46.3), Barake stream (tributary of Aborlan River), Magbabadil, Aborlan, 15 May 2015; all samples from Palawan Island.

**(419) *Stiphodonsurrufus* Watson & Kottelat, 1995: 13, fig. 8**

**Holotype.** ZRC 38394, Lagu Lagu creek, south of Visayan State College of Agriculture (now VSU)., ~ 7 km north of Baybay, Leyte Island.

**Paratypes.** CMK 9782 (3), CMK 9831 (1) and ZRC 38395 (2).

**Remarks.** The original description did not indicate the collection date.

**(420) *Taenioidescaniscapulus* Roxas & Ablan, 1938: 261, pls 1, 2**

**Holotype.** BSMP 41349 (256.5 TL) Government Experimental Fish Farm, Hinigaran, Negros Island, 15 Nov. 1936.

**Remarks.** The type specimen was lost (Kottelat 2013).

**(421) *Tamankamaculata* Aurich, 1938: 154**

= *Tamankasiitensis* Herre, 1927.

**Syntypes.** ZMH (10.0–49.0 TL), Lake Timpuk, Jolo, Sulu Archipelago.

**Remarks.** The original description did not indicate the collection date. All type specimens were lost (Kottelat 2013).

**(422) *Tamankamindora* Herre, 1945d: 75**

= *Mugilogobiusmertoni* (Weber, 1911).

**Holotype.**CAS-SU 39885 (23.0), mangrove swamp at Hacienda Waterous, Mangarin, Mindoro Island, 20–22 Jul. 1940.

**Remarks.** A photograph and a radiograph of the holotype are available in the CAS record.

**(423) *Tamankaphilippina* Herre, 1945d: 75**

= *Mugilogobiuscavifrons* (Weber, 1909).

**Holotype.**CAS-SU 39884 (male, 22.0), mangrove swamp at Hacienda Waterous, Mangarin, Mindoro Island, 20–22 Jul. 1940.

**Remarks.** A photograph and a radiograph of the holotype are available in the CAS record.

**(424) *Tamankasiitensis* Herre, 1927c: 220, pl. 17 (fig. 3)**

**Neotype.**USNM 87128 (male), Lake Siit, Jolo Island, Sulu Archipelago, 11 Jun. 1921.

**Paralectotypes.**CAS-SU 26368 (82), same locality as holotype, 18 Jul. 1929; CAS-SU 38624 (64), Lake Panamao, Sulu Archipelago, 12 Sept 1940.

**Remarks.** The original type specimens (BSMP 11452) were destroyed and a neotype was designated by Larson (2001) from USNM paratypes. Eleven paratypes (USNM 355555 [ex USNM 87128]) were mentioned available by Larson (2001) and one of the specimens should have been designated as the lectotype and the rest as paralectotypes.

**(425) *Tamankatagala* Herre, 1927c: 222**

**Holotype.** BSMP 820 (31.0), Malabon, Rizal, Luzon, Jul. 1907.

**Paratype.** BSMP 804 (1 female, 33.0), same data as holotype.

**Remarks.** All type specimens were lost (Kottelat 2013). Possibly a *Mugilogobius*, but could also be synonym of *Mugilogobiuscavifrons* (Weber, 1909), (Larson 2001; Kottelat 2013). Further examination of specimens is needed to confirm its status.

**(426) *Tamankatalavera* Herre, 1945a: 4**

= *Mugilogobiuscavifrons* (Weber, 1909).

**Holotype.**CAS-SU 36824 (male, 33.0), nipa swamp near Capiz, Panay Island, 3 Aug. 1940.

**Paratypes.**CAS-SU 36825 (4 males, 20.0–31.0 + 4 females, 28.0–29.0 + 2 juveniles, 11.0–14.0, same data as holotype.

**Remarks.** A photograph and a radiograph of the holotype are available in the CAS record.

**(427) *Tamankaumbra* Herre, 1927c: 223**

= *Eugnathogobiusumbra* (Herre, 1927).

**Holotype.** BSMP 10600 (75.0 TL), Palawan Island, Jun. 1910.

**Paratypes.** BSMP 26893–98 (6, 42.0–60.0), same data as holotype.

**Remarks.** All type specimens were lost (Kottelat 2013).

**(428) *Tukugobiusbucculentus* Herre, 1927c: 121, pl. 8 (fig. 4)**

= *Rhinogobiusbucculentus* (Herre, 1927).

**Holotype.** BSMP 11543 (62.0), creek at Station Fe, Nueva. Vizcaya, Luzon, 18 May 1924.

**Paratypes.** BSMP 12399 (1) and BSMP 26563–70 (9) (34.0–78.0 TL) same data as the holotype.

**Remarks.** All type specimens were lost (Kottelat 2013).

**(429) *Tukugobiusphilippinus* Herre, 1927c: 124**

= *Rhinogobiusphilippinus* (Herre, 1927).

**Syntypes.** BSMP 12406 and BSMP 26469–89 (many), Irid River, Santa Ines, Rizal and Banaban River, Angat, Bulacan; both in Luzon, 29 Sep. 1925.

**Remarks.** Herre described the species based on 14 specimens (33.0–53.0) from the Irid River and 22 co-types measuring 30.0–60.0 from the Banaban River. Koumans (1940) provided a taxonomic remark. All type specimens were lost (Kottelat 2013).

**(430) *Vaimosabikolana* Herre, 1927c: 151, pl. 11 (fig. 2)**

= *Redigobiusbikolanus* (Herre, 1927).

**Syntypes.** BSMP 13232 (6, 23.0–26.0), creek at Barrio Puru, Legaspi, Albay, 4 Feb. 1926.

**Remarks.**Koumans (1940) provided a taxonomic remark. All type specimens were lost (Kottelat 2013).

**(431) *Vaimosacagayanensis* Aurich, 1938: 169, fig. 22**

= *Mugilogobiuscagayanensis* (Aurich, 1938).

**Lectotype.** ZMH H420, ZMH 420a (25.0), Singuan and Sulu Lakes, Cagayan Sulu, 14 May 1932.

**Paralectotypes.** ZMH H420 (2, 18.5–20.0), same data as lectotype.

**Remarks.** The syntypes are in poor condition (Larson 2001). The largest specimen was isolated and designated as lectotype (ZMH H420) by Ladiges et al. (1958) and ZMH 420a by Larson (2001).

**(432) *Vaimosacardonensis* Herre, 1940: 358, pl. 2**

= *Redigobiustambujon* (Bleeker, 1854).

**Holotype.**CAS-SU 32980 (male, 21.0), Cardona, north of Laguna de Bay, Luzon, Aug. 1936.

**Paratypes.**CAS-SU 32981 (30), same data as holotype; BMNH 1938.12.1.208–212 (5) Cardona, Laguna de Bay, Luzon; (14.0–21.0).

**Other catalog number.**BMNH 1938.12.1.208–212 (NHMUK:ecatalogue:2517105).

**Remarks.** A photograph and a radiograph of the holotype are available in the CAS record.

**(433) *Vaimosafusca* Herre, 1940: 359, pl. 3**

= *Mugilogobiusfuscus* (Herre, 1940).

**Holotype.**CAS-SU 32984 (female, 32.0), Tide pool at Dumaguete, Negros Island, 24 Dec. 1936.

**Paratypes.**CAS-SU 32985 (12), same data as holotype; BMNH 1938.12.1.213–214 (2), Cardona, Laguna de Bay, Luzon; CAS-SU 32986 (1), Guinlo, Malampaya Sound, Taytay, Palawan Island, 9 May 1927.

**Other catalog number.**BMNH 1938.12.1.213–214 (NHMUK:ecatalogue:2517106).

**Remarks.** A male paratype (24.0) and 15 paratypes (12.5–29.0) from Dumaguete were mentioned in the original description but no catalog numbers were provided. The types were inferred from Böhlke (1953). A photograph and a radiograph of the holotype are available in the CAS record.

**(434) *Vaimosalayia* Herre, 1953b: 769**

= *Mugilogobiusmertoni* (Weber, 1911).

**Holotype.**USNM 202503 [ex UW 18959] (male, 31.0), Laiya, Batangas, Luzon.

**Paratypes.**USNM 202573 [ex UW 7554] (3 females, 23.0–29.0), same locality as holotype, 30 Jun. 1948.

**Remarks.** The original description or USNM record did not indicate the collection date. A radiograph of the holotype is available.

**(435) *Vaimosamacrognathos* Herre, 1927c: 145, pl. 10 (fig. 2)**

= *Redigobiustambujon* (Bleeker, 1854).

**Syntypes.** BSMP 13059 (13, 15.5–31.5), Taal Lake, Batangas, Luzon, 8 Nov. 1925.

**Remarks.**Koumans (1940) provided taxonomic remark and designated a lectotype from syntypes. The type specimens were lost (Kottelat 2013).

**(436) *Vaimosamicrostomia* Seale, 1910: 538**

**Holotype.** BSMP 827 (33.0), Malabon, Rizal, Luzon, 18 Jul. 1907.

**Remarks.** The type specimen was presumed lost during the World War II (Kottelat 2013). Possibly a *Redigobius* or *Pseudogobius* (Larson 2001; Larson and Hammer 2021).

**(437) *Vaimosamindora* Herre, 1945b: 32**

= *Eugnathogobiusmindora* (Herre, 1945).

**Holotype.**CAS-SU 36826 (male, 23.0), brackish swamp on Hacienda Waterous, Mangarin, Mindoro Island, 21 Jul. 1940.

**Paratypes.**CAS-SU 36827 (1 male, 20.0 + 1 female, 21.0), same data as holotype.

**Remarks.** A photograph and a radiograph of the holotype are available in the CAS record.

**(438) *Vaimosamontalbani* Herre, 1936b: 359, pl. 1 (fig. 3)**

= *Redigobiusbikolanus* (Herre, 1927).

**Holotype.**CAS-SU 30967 (20.0), Lake Naujan, Mindoro Island, 28 Nov. 1933.

**Paratypes.**CAS-SU 30967 (59), same data as holotype.

**Remarks.** The holotype and paratypes were mixed in the same jar and one possibly the holotype (Böhlke 1953). A photograph and radiographs of the type specimens are available in the CAS record.

**(439) *Vaimosapiapensis* Herre, 1927c: 147, pl. 10 (fig. 3)**

= *Pseudogobiuspoicilosoma* (Bleeker, 1849).

**Syntypes.** BSMP (12, 21.5–29.0), Piapi Creek, Dumaguete, Negros Island, 5 Mar. 1922.

**Remarks.** All type specimens were lost (Kottelat 2013).

**(440) *Vaimosarivalis* Herre, 1927c: 149, pl. 11 (fig. 1)**

**Syntypes.** BSMP 13061 (16, 8.0–28.0), Talakop Creek; BSMP 13602 (8, 16.0–33.0), Hinagianan River, both from Camarines Sur, Luzon, both taken on 16 Jan. 1926.

**Remarks.**Larson (2001) and Kottelat (2013) suggested it could be a *Redigobius* or *Eugnathogobius*. All type specimens were presumed destroyed during the World War II (Kottelat 2013).

**(441) *Vaimosasapanga* Herre, 1927c: 152, pl. 11 (fig. 3)**

= *Redigobiustambujon* (Bleeker, 1854).

**Syntypes.** BSMP 13229 (21, 16.0–26.0), Sapanga Creek, Angat, Bulacan, Luzon, 24 Sep. 1925.

**Remarks.**Koumans (1940) noted 19 specimens only and provided a taxonomic remark. All type specimens were lost (Kottelat 2013).

**(442) *Vaimosatessellata* Herre, 1927c: 153, pl. 12 (fig. 1)**

= *Pseudogobiuspoicilosoma* (Bleeker, 1849).

**Syntypes.** BSMP 12999 (5, 23.5–38.0), Titunod River, Kolambugan, Lanao, Mindanao, 26 May 1921.

**Remarks.**Koumans (1940) mentioned type and syntypes (27.0–47.0). All type specimens were lost during the World War II (Kottelat 2013). Larson (2010) suggested it could be a *Redigobius* or a *Pseudogobius*.

**(443) *Vaimosavilla* Herre, 1927c: 154, pl. 12 (fig. 2)**

**Holotype.** BSMP (36.0), Villa, Iloilo, Panay Island, 8 Jul. 1925.

**Paratype.** BSMP 13228 (1, 35.0), Molo, Iloilo, Panay Island, 15 Aug. 1925.

**Remarks.**Larson (2010) suggested it could be a *Mugilogobius*. Uncertain as *Mugilogobiusvilla* (Herre, 1927) (Kottelat 2013). All type specimens were lost during the World War II (Kottelat 2013).

**(444) *Vaimosazebrinus* Herre, 1950: 74**

= *Eugnathogobiusmindora* (Herre, 1945).

**Holotype.**USNM 202515 [ex UW 19695] (male, 25.0) Laiya, Batangas, Luzon, 30 Jun. 1948.

**Paratypes.**USNM 202572 [ex UW 7539] (3 females, 24.0–25.0), same data as the holotype.

**Remarks.** Radiographs of the holotype are available in the USNM record.

##### Family Gobiidae (282)

**(445) *Aioliopsbrachypterus* Rennis & Hoese, 1987: 75, figs 2a, 10a, 12**

**Holotype.** NSMT-P 44087 (male, 19.0), Mini Rock Island, El Nido, Palawan, 5 Jun. 1984.

**Paratypes.** LICPP 1984275 (7, 17.0–2.00), taken with holotype.

**(446) *Aioliopsmegastigma* Rennis & Hoese, 1987: 76, figs 2b, 13**

**Holotype.** NSMT-P 44114 (female, 23.7), El Nido, Palawan Island, 6 Mar. 1983.

**Paratypes.** AMS I.25435-001 (2, 16.0–17.0), CAS 57923 (1, 18.0), USNM 246731 (8, 18.0–20.0) and USNM 257054 (11, 16.0–18.0), Bararin Island, Cuyo, Palawan, 0–15 m, 23 May 1978; LICPP 1984269 (4, 16.0–20.0), Mini Rock Island, El Nido, Palawan, 5 Jun. 1984.

**(447) *Amblyeleotrislatifasciata* Polunin & Lubbock, 1979: 247, fig. 5**

**Holotype.**BMNH 1978.2.28.4 (65.1) passage between Cabulan and Vandanon islands, Cebu Strait, 15 m, 21 Aug. 1976.

**Other catalog number.** NHMUK:ecatalogue:2544343.

**(448) *Amblyeleotrisrandalli* Hoese & Steene, 1978: 382, figs 1–3**

**Holotype.** BPBM 20809 (female, 73.2), west of Sumilon Island, Cebu, 22 m, 29 Aug. 1977.

**(449) *Amblyeleotrisrhyax* Polunin & Lubbock, 1979: 239, fig. 1**

**Holotype.**BMNH 1978.2.28.8. New Britain, Bismarck Archipelago, Papua New Guinea.

**Paratype.** AMS I.20688-001 (1, 70.0), Maribago, Mactan Island, Cebu, 35 m, 6 Aug. 1976.

**(450) *Amblygobiuscalvatus* Allen & Erdmann, 2016: 13, figs 1A, 2–4**

**Holotype.** WAM P.32885-002 (male, 42.6) Big Lagoon, Miniloc Island, off El Nido, Palawan, 18–20 m, 14 Jun. 2009.

**Paratypes.**USNM 432516 (2, 37.6–42.9) and WAM P.32885-013 (6, 23.7–48.0), taken with holotype.

**(451) *Amblygobiusinornatus* Herre, 1927c: 228**

= *Bathygobiusfuscus* (Rüppell, 1830).

**Holotype.** BSMP 13223 (37.0), tide pool on the Martin ranch, Siasi, Sulu Archipelago, 21 Jun. 1921.

**Remarks.** The type specimen was lost (Kottelat 2013).

**(452) *Amblygobiusinsignis* Seale, 1910: 116, pl. 2 (fig. 1)**

= *Cryptocentroidesinsignis* (Seale, 1910).

**Holotype.** BSMP 5779 (57.0), Bantayan Island, Cebu, May 1909.

**Remarks.** The type specimen was lost (Kottelat 2013).

**(453) *Amblygobiuslinki* Herre, 1927c: 231, pl. 18 (fig. 4)**

**Holotype.** BSMP 24146 (52.0), wharf at Bungau, Sulu Archipelago.

**Paratype.** BSMP 4022 (1, 35.0), Caldera Bay, Mindanao.

**Remarks.** The collection dates were not indicated in the original description. All type specimens were lost (Kottelat 2013).

**(454) Amblygobiusperpusillusvar.buanensis Herre, 1927c: 230, pl. 18 (fig. 2)**

= *Amblygobiusbuanensis* Herre, 1927.

**Syntypes.** BSMP 5106 (1) and BSMP (1), Puerto Princesa, Palawan Island; Buan Island, off east of Tawi Tawi, Sulu Archipelago.

**Remarks.** The collection dates were not indicated in the original description. All type specimens were lost (Kottelat 2013).

**(455) *Andameleotrispalustris* Herre, 1945a: 2**

= *Parioglossuspalustris* (Herre, 1945).

**Holotype.**CAS-SU 36808 (male, 23.0), pool in a nipa swamp, near the Fisheries Station, Zamboanga, Mindanao, 10 Sep. 1940.

**Paratypes.**CAS-SU 18473 (1) and CAS-SU 36809 (3, 18.0–19.0), same data as holotype.

**Remarks.** A photograph and a radiograph of the holotype are available in the CAS record.

**(456) *Aparriusmoloanus* Herre, 1927c: 207, pl. 16 (fig. 3)**

= *Acentrogobiusmoloanus* (Herre, 1927).

**Syntypes.** BSMP 12369 and BSMP 24389–92 (5, 43.0–64.0), Molo, Iloilo, Panay Island, 15 Aug. 1925.

**Remarks.** The original description mentioned 12 specimens. All type specimens were lost (Kottelat 2013).

**(457) *Aparriussabagensis* Roxas & Blanco, 1940: 165, pl. 1**

= *Stenogobiusophthalmoporus* (Bleeker, 1853).

**Holotype.** BSMP 41994, Cagayan River, Barrio Catayaoan, Lallo, Cagayan, Luzon, 31 Jan. 1939.

**Paratype.** BSMP (30, 29.0–58.0).

**Remarks.** All type specimens were lost (Kottelat 2013).

**(458) *Bathygobiusblancoi* Roxas & Ablan, 1940: 306, pl. 5**

= *Bathygobiusmeggitii* (Hora & Mukerji, 1936).

**Holotype.** BSMP 31949, Dagupan, Pangasina, Luzon Island.

**Remarks.** The collection dates were not indicated in the original description. The type specimen was lost (Kottelat 2013).

**(459) *Bathygobiusbravoi* Herre, 1927c: 112, pl. 8 (fig. 1)**

= *Bathygobiusfuscus* (Rüppell, 1830).

**Lectotype.** BSMP 13019, coral reef pool at Romblon, Romblon.

**Remarks.** The collection dates were not indicated in the original description. The lectotype has been designated by Koumans (1940). The type specimen was lost (Kottelat 2013).

**(460) *Bathygobiuslaoe* Roxas & Ablan, 1940: 306, pl. 4**

= *Bathygobiuscyclopterus* (Valenciennes, 1837).

**Holotype.** BSMP 31948, Dagupan, Pangasinan, Luzon. Remarks: The collection dates were not indicated in the original description. The type specimen was lost (Kottelat 2013).

**(461) *Bathygobiusramosuscurticeps* Ginsburg, 1947: 281**

= *Bathygobiusramosus* Ginsburg, 1947.

**Holotype.**USNM 30739. Cape San Lucas, Baja California, Mexico.

**Paratype.**USNM 261376 (4), Bararin Island, Cuyo, Palawan, 0–14 m, 23 May 1978; USNM 261376 (10), Tagauayan Island, Cuyo, Palawan, 0–14 m, 25 May 1978.

**Remarks.** The original description mentioned 50 specimens (23.0–92.0) from the Pacific coast of Mexico and 16 specimens (19.0–86.0) from Ecuador and Colombia but did not mention the Philippines as a type locality. The paratypes were seen in the USNM record.

**(462) *Biatluzonica* Seale, 1910: 532**

= *Amblyeleotrisfontanesii* (Bleeker, 1853).

**Holotype.** BSMP 2040 (190.0), east of Luzon, Jun. 1905.

**Remarks.** The type specimen was lost (Kottelat 2013).

**(463) *Bryaninopsamplus* Larson, 1985: 66, figs 5–6**

**Holotype.** AMS I.22916-001. Palfrey Island, Great Barrier Reef, Australia.

**Paratypes.** AMS I.21913-001 (20, 22.0–46.0), Anilao Beach, Batangas, Luzon, 20 m, 23 Apr. 1980.

**(464) *Bryaninopsisis* Larson, 1985: 89, fig. 17**

**Holotype.** AMS I.25301.001. Palfrey Island, Great Barrier Reef, Australia.

**Paratypes.**USNM 269837 (2, 14.5–16.5), Tagauayan Island, Cuyo, Palawan, 0–14 m, 25 May 1978.

**(465) *Bryaninopsloki* Larson, 1985: 81, figs 13–14**

**Holotype.** AMS I.24072.001. Between Bird and South islands, Great Barrier Reef, Australia.

**Paratypes.**USNM 265176 (1 female, 14.0), Tagauayan Island, 0–14 m, 25 May 1978; USNM 261374 (1 male, 15.0), Bararin Island, 0–17 m, 24 May 1978; all from Cuyo, Palawan.

**(466) *Bryaninopsnatans* Larson, 1985: 77, figs 10–12**

**Holotype.** AMS I.24067-001. Palfrey Island, Great Barrier Reef, Australia.

**Paratypes.**USNM 261377 (10, 10.5–14.5), Tagauayan Island, Cuyo, Palawan, 0–14 m 24 May 1978; USNM 261376 (4, 12.0–23.0), Bararin Island, Cuyo, Palawan, 0–14 m, 23 May 1978; AMS I.21915-076 (2, 12.0–15.0), Sombrero Island, Batangas, Luzon, 6 m, 24 Apr. 1980; CAS 53209 (3, 11.0–14.0), off San Carlos Research Station, Mactan Island, Cebu, 14–21 m, 29 Apr. 1980.

**(467) *Callogobiusclitellus* McKinney & Lachner, 1978: 211, fig. 5**

**Holotype.**USNM 209249. Bay in Krankett Island, Madang Harbor, Papua New Guinea.

**Paratype.**USNM 99574 (1 male, 37.6), Philippines.

**Remarks.** The original description did not provide a specific locality and collection date. This paratype was a former Fowler manuscript as the holotype of *Callogobiusphilippinus* (Smithsonian Museum Archive 2024).

**(468) *Callogobiuscrassus* McKinney & Lachner, 1984: 627, fig. 1**

**Holotype.**USNM 220088. Massas Island, Papua New Guinea.

**Paratypes.**USNM 220086 (1 male, 17.5), station SP 78-38, Balicasag Island, west of Luzon, 0–24 m, 10 Jun. 1978; USNM 220087 (1 male, 17.2), station SP 78–39, same locality as above, 11 Jun. 1978.

**(469) *Callogobiushastatus* McKinney & Lachner, 1978: 206, figs 1–4**

**Holotype.**USNM 216811. Koror Island, Madalai District, Palau Islands.

**Paratypes.**USNM 99293 (1 male, 28.9) and USNM 99294 (5 males, 18.7–27.7; 7 females, 18.6–24.4), Mactan Island, reef opposite Cebu Island, 7 Apr. 1908; AMS I.19603-001 (1 male, 25.3, 1 female, 22.2), same data as USNM 99293; USNM 99570 (1 female, 18.8), Cascade River, Murcielagos Bay, Mindanao Island, 20 Aug. 1909; USNM 139323 (1 female, 20.5), Mactan Island, Cebu, 31 Aug. 1909.

**Remarks.**USNM 99293–94 and 99570 were former Fowler manuscript paratypes of *Callogobiusadae*. Two specimens of USNM 99294 were removed and exchanged to an Australian Museum in Sydney. USNM 139323 was former Fowler manuscript paratype of *Callogobiusleopardus*.

**(470) *Chlamydesleytensis* Herre, 1927c: 118, pl. 8 (fig. 3)**

= *Bathygobiuscotticeps* (Steindachner, 1879).

**Holotype.** BSMP 9550 (~57.0), Cabalian, Leyte Island, 23–28 May 1921.

**Paratypes.** BSMP 10585 (1) and BSMP 26919 (1) (~57.0), same data as holotype.

**Remarks.** All type specimens were lost (Kottelat 2013). Koumans (1940) did not designate a lectotype, contrary to what is mentioned in Fricke et al. (2024).

**(471) *Coronogobiusstriatus* Herre, 1945c: 81**

= *Trimmastriatum* (Herre, 1945).

**Holotype.**CAS-SU 39854 (21.0), little dock at Coron, Busuanga Island, Palawan, 22 Jun. 1940.

**Remarks.** A photograph and a radiograph of the holotype are available in CAS’ record.

**(472) *Coryphopterusgracilis* Randall, 2001b: 208, fig. 4**

= *Fusigobiusgracilis* (Randall, 2001).

**Holotype.** BPBM 22296. Sesoko Island, Okinawa, Ryukyu Islands, Japan.

**Paratypes.** ROM 49507 (2, 20.3–33.6), 5 km north of Tambuli Beach Resort, Mactan Island, Cebu, 9 Aug. 1985; ROM 53186 (7, 14.1–35.6), mouth of Bais Bay, Negros Island, 17 May 1987; ROM 53187 (2, 22.2–27.2), west of Bohol Island, 21 May 1987.

**(473) *Coryphopterushumeralis* Randall, 2001b: 212, figs 5, 6**

= *Fusigobiushumeralis* (Randall, 2001).

**Holotype.** BPBM 32955. South Malé Atoll, Maldives.

**Paratypes.** ROM 53304 (2, 19.2–25.9), west of Tonga Point, Siquijor Island, 9 May 1987; ROM 53306 (3, 25.7–26.0), south end of Cebu Island, 20 May 1987.

**(474) *Coryphopterusmaximus* Randall, 2001b: 215, figs 10, 11**

= *Fusigobiusmaximus* (Randall, 2001).

**Holotype.** BPBM 28539 (female, 54.7), off South Sea Resort Hotel, Dumaguete, Negros Island, 21 m, 3 Jun. 1981.

**Paratypes.**USNM 360976 (1, 46.0), same locality as holotype, 15–21 m, 4 Jun. 1981; ROM 49451 (3, 35.5–47.7), northwest of Sumilon Island, Cebu, 11 Aug. 1985.

**(475) *Coryphopterusmelacron* Randall, 2001b: 218, figs 12–14**

= *Fusigobiusmelacron* (Randall, 2001).

**Holotype.** BPBM 31581. Tulamben, Bali, Indonesia.

**Paratype.** BPBM 28466 (1, 30.7), west of Caban Island, Batangas, Luzon, 28–30 m, 25 May 1981.

**(476) *Coryphopteruspallidus* Randall, 2001b: 221, figs 16, 17**

= *Fusigobiuspallidus* (Randall, 2001).

**Holotype.** BPBM 33580. Chesterfield Bank, Coral Sea, Australia.

**Paratype.**MNHN 2000-760 (1, 60.4), Sumilon Island, Cebu, 15 m, 3 Jun. 1981.

**(477) *Creissonvalidus* Jordan & Seale, 1907: 43, fig. 16**

= *Acentrogobiusjanthinopterus* (Bleeker, 1853).

**Holotype.**CAS-SU 9251 (120.7), Cavite, Luzon, 1 Jun. 1900.

**Remarks.** A photograph and a radiograph of the holotype are available in the CAS record.

**(478) *Cristatogobiuslophius* Herre, 1927c: 170, pl. 13 (fig. 1)**

**Syntypes.** BSMP 12106 (2, 22.0–25.0), wharf at Bungao, Tawi Tawi, Sulu Archipelago, 8 Jun. 1921.

**Remarks.** All type specimens were lost (Kottelat 2013).

**(479) *Cryptocentruscebuanus* Herre, 1927c: 240, pl. 19 (fig. 2)**

**Holotype.** BSMP 12025 (89.0), Cebu Island.

**Remarks.** The collection date was not indicated in the original description. The type specimen was lost (Kottelat 2013).

**(480) *Cryptocentruscyanospilotus* Allen & Randall, 2011: 556, figs 1–5**

**Holotype.** BPBM 31446. Ngargol Island, Palau Islands.

**Paratype.** WAM P.32885-004 (1 male, 40.2), north side of Big Lagoon, Miniloc Island, El Nido, Palawan, 8 m, 14 Jun. 2007.

**(481) *Cryptocentrusvagus* Herre, 1927c: 243, pl. 19 (fig. 3)**

**Holotype.** BSMP 12138 (44.0), Mindoro or Mindanao.

**Remarks.** Uncertain status (Hoese and Larson 2004). The original description did not indicate the collection date and mentioned an unknown origin of the type, either from Mindoro or Mindanao. Herre’s drawing in looks like a *Myersina* species. The type specimen was presumed destroyed and no other specimens were collected after the World War II (Larson pers. comm.).

**(482) *Ctenogobiusculionensis* Herre, 1934: 84**

= *Cryptocentruscaeruleomaculatus* (Herre, 1933).

**Holotype.**CAS-SU 26387 (48.0 TL), reef in Culion Harbor, Palawan, 3 May 1931.

**Remarks.** A photograph and a radiograph of the holotype are available in the CAS record.

**(483) *Ctenogobiusnuchipunctatus* Herre, 1934: 85**

= *Silhouetteanuchipunctata* (Herre, 1934).

**Holotype.**CAS-SU 26246, tide pool at Dumaguete, Negros Island, 18 Jun. 1931.

**Paratypes.**CAS-SU 69108 (5, 34.0 TL), same data as holotype.

**Remarks.** The holotype cannot be separated from other paratypes in the same bottle (Böhlke 1953). A photograph and a radiograph of the holotype are available in the CAS record.

**(484) *Ctenogobiusvilladolidi* Herre, 1936b: 361, pl. 2 (fig. 4)**

= *Silhouetteanuchipunctata* (Herre, 1934).

**Holotype.**CAS-SU 30957 (31.0), Tide pool near Dumaguete, Negros Island, 23 Nov. 1933.

**Paratypes.** FMNH 47063 (2), FMNH 47092 (2) and CAS-SU 69107 (10) (17.0–31.0), same data as holotype.

**Remarks.** A photograph and a radiograph of the holotype are available in the CAS record.

**(485) *Drombusmaculipinnis* Fowler, 1918: 69, fig. 27**

= *Callogobiusmaculipinnis* (Fowler, 1918).

**Holotype.**ANSP 47549 (50.0), Philippines.

**Remarks.** The original description did not provide a specific locality and collection date. Koumans (1940) noted that the specimen is closely allied or identical with *Callogobiussclateri* based on ANSP record.

**(486) *Drombuspalackyi* Jordan & Seale, 1905: 797, fig. 15**

**Holotype.**USNM 51954 (47.0), southern Negros Island, 1901.

**Remarks.**Koumans (1940) initially placed it in *Acentrogobius* and provided a short taxonomic remark.

**(487) *Eviotaasymbasia* Greenfield & Jewett, 2016: 590, figs 2–4, 5a**

**Holotype.**USNM 229476 (male, 15.0), Cuyo Island, Palawan, 1 m, 21 May 1978.

**Paratypes.**USNM 437288 (4 males, 12.7–14.1 + 1 female, 14.6) and CAS 238219 (2 males, 14.0 + 2 females, 13.5–14.0) taken with holotype; ROM 101093 (2 males, 11.6–11.7 + 1 female, 12.0), Bararin Island, Cuyo, Palawan, 0–13.7 m, 23 May 1978; USNM 229470 (male, 16.8), Solino Island, Zamboanga, Mindanao, 0–4.6 m, 3 May 1979; USNM 229471 (male, 14.8), Tagauayan Island, 0–10 m, 10 May 1978; USNM 229473 (3 males, 13.4–14.2 + 1 female, 15.1), Putic Island, Cuyo, Palawan, 0–4.6 m, 22 May 1978; USNM 229475 (female, 12.0) and USNM 229477 (female, 12.2), Siquijor Island, 0–10 m, 9–10 May 1978.

**Remarks.**USNM specimens were not found in the USNM record and possibly not yet deposited.

**(488) *Eviotabifasciata* Lachner & Karnella, 1980: 108, figs 62–64**

**Holotype.**USNM 219276 (male, 22.5), Bararin Island, Cuyo, Palawan, 0–14 m, 23 May 1978.

**Paratypes.**USNM 219272 (3 males, 21.8 + 1 female, 15.3), same data as holotype.

**(489) *Eviotabipunctata* Greenfield & Jewett, 2016: 595, figs 7–11**

**Holotype.**USNM 225176 (male, 14.0), northwest of Putic Island, Cuyo, Palawan, 0–4.6 m, 22 May 1978.

**Paratypes.**USNM 225175 (5, 14.2–17.0), CAS 47920 (2, 15.3–15.6), AMS I.22210-001 (2, 15.8–17.0), ANSP 146760 (2, 15.4–17.2), same data as holotype; USNM 225174 (9, 11.6–13.1), CAS 47919 (1, 13.9), AMS I.22207-001 (2, 12.4–13.5), USNM 225173 (1, 11.9), Maloh, Negros Island, 0–3.1 m, 18 May 1979; USNM 225171 (3, 13.0–14.7), west of Solino Island, Zamboanga, Mindanao, 0–4.6 m, 3 May 1979; USNM 225170 (4, 13.7–15.4), Cocoro Island, Cuyo, Palawan, 0–3 m, 26 May 1978; BPBM 26540 (1, 12.8), SE Ajong, Negros Island, 0–2.4 m, 8 Jun. 1978; USNM 225172 (1, 17.2), Port Siyt, Negros Island, 0–2 m, 14 Jun. 1978.

**Remarks.**USNM specimens were not found in the USNM record and their location are unknown.

**(490) *Eviotairrasa* Karnella & Lachner, 1981: 272, figs 4, 5**

**Holotype.**USNM 220566 (male, 16.0), Cocoro Island, 0–3 m, 25 May 1978.

**Paratypes.**USNM 220954 (5, 12.3–17.6), same data as holotype; USNM 220582 (1, 17.4), Putic Island, 2 May 1978; all from Cuyo, Palawan,

**(491) *Eviotaminuta* Greenfield & Jewett, 2014: 13, figs 1–5**

**Holotype.**USNM 230090 (female, 12.9), 09°03.10'N, 122.00.00°E, Maloh, Negros Island, 0–3.1 m, 18 May 1979.

**Paratypes.**USNM 230088 (6 males, 11.4–12.7 + 2 females, 10.1–11.7), taken with holotype; USNM 230089 (female, 12.0), west of Solino Island, Zamboanga, Mindanao, 0–4.6 m; AMNH 55059 (female, 13.5), Port Siyt, southern Negros Island, 0–3.1 m; AMS I.23989-001 (1, 10.0), ANSP 150919 [ex USNM] (1 male, 13.2) and CAS 52734 (1 male 12.1 + 1 female, 12.0), Putic Island, Cuyo, Palawan, 0–4.6 m.

**(492) *Eviotapunctulata* Jewett & Lachner, 1983: 793, figs 6, 7**

**Holotype.**USNM 224550. Vurosewa Island, Fiji.

**Paratype.**USNM 224541 (4, 15.3–18.3), SP 78-25, Tagauayan Island, Cuyo, Palawan, 0–2 m, 25 May 1978; CAS 47910 (3, 13.9–16.0), SP 78-20, northwest of Bararin Island, Cuyo, Palawan, 0–13.7 m, 23 May 1978; AMS I.22209-001 (2, 15.1–18.3), SP 78-17, Cuyo Island, Palawan, 0.6–1.2 m, 21 May 1978.

**(493) *Eviotasealei* Herre, 1927c: 73**

**Holotype.** BSMP 7372 (17.0 TL), Puerto Galera, Mindoro Island.

**Remarks.** The type specimen is presumed destroyed during the World War II and its description does not look like an *Eviota* species. Dave Greenfield did not include the species in any of his reviews of the genus, suggesting that he likely did not consider it a valid species of the genus (Larson pers. comm.).

**(494) *Eviotaspilota* Lachner & Karnella, 1980: 54, figs 29–31**

**Holotype.**USNM 219853. Ninigo Island, Papua New Guinea.

**Paratypes.**USNM 219438 (female, 19.0) and USNM 219439 (male, 22.8), Bararin Island, Cuyo, Palawan, 0–14 m, 23–24 May 1978.

**(495) *Eviotasparsa* Jewett & Lachner, 1983: 802, figs 11–13**

**Holotype.**USNM 227483. Tutuila Island, American Samoa.

**Paratypes.**USNM 227485 (1, 13.4), Cocoro Island, Cuyo, Palawan, 0–21 m, 26 May 1978; AMNH 55055 (1, 16.7), Tagauayan Island, Cuyo, Palawan, 0–13.7 m; AMS I.23987-001 (1, 17.2), Siquijor Island, 0–10.7 m; USNM 227481 (4, 15.6–17.9), Siquijor Island, 26–30 m, 15 May 1979.

**(496) *Eviotopsstorthynx* Rofen, 1959: 237, figs 1–3**

= *Eviotastorthynx* (Rofen, 1959).

**Holotype.**CAS-SU 52108 (male, 17.3), Bungau, Tawi-Tawi Group, Sulu Archipelago, 17 Sep. 1940.

**Paratypes.**CAS-SU 39853 (male, 14.8), same data as holotype; CAS-SU 38510 (female, 12.6), Coron, Busuanga Island, Palawan, 24 Jun. 1940.

**Remarks.** A photograph and a radiograph of the holotype are available in the CAS record.

**(497) *Exyriasakihito* Allen & Randall, 2005: 232, figs 1–6**

**Holotype.** NSMT-P 71648. Amitori Bay, Iriomote Island, Okinawa, Japan.

**Paratype.** BLIH 19830335 (1, 34.9), El Nido, Palawan Island, 6–25 Mar. 1983.

**(498) *Exyriasferrarisi* Murdy, 1985: 8, pl. IIA, figs 1–3**

**Holotype.**USNM 260640, Bolinao, Pangasinan, Luzon, 1–2 m, 12 Feb. 1980.

**Paratype.**USNM 265012 (1), same data as holotype.

**Remarks.** Originally, USNM 260640 contained two specimens but one was removed and designated as the paratype (Smithsonian Museum Archive 2024).

**(499) *Fusigobiussignipinnis* Hoese & Obika, 1988: 282, figs 1–7**

**Holotype.** AMS I. 20730-008. Lizard Island, Queensland, Australia.

**Paratypes.** AMS I.21907-21 (1, 29.0), Anilao, 15–21 m, 22 Apr. 1980; AMS I.21914-050 (3, 23.0–32.0), Caban Island, 9–25 m, 24 Apr. 1980; AMS I.21918-063 (2, 27.0–33.0), Caban Island, 11–29 m, 25 Apr. 1980; AMS I.21922-021 (3, 27.0–32.0), 18–21 m, 26 Apr. 1980; all from Batangas, Luzon.

**(500) *Galeraproducta* Herre, 1927c: 104, pl. 7 (fig. 3)**

= *Callogobiusproducta* (Herre, 1927).

**Holotype.** BSMP 7417 (70.0 TL), Puerto Galera, Mindoro Island, May 1912.

**Remarks.** The type specimen was lost (Kottelat 2013).

**(501) *Gladiogobiusensifer* Herre, 1933: 23**

**Holotype.** FMNH 17412. Waigeo Island, West Papua, Indonesia.

**Paratypes.**CAS-SU 25499 (2, 28.0–34.0) and CAS-SU 26389 (2, 39.0), Culion Island, Palawan, 3 May 1931.

**(502) *Glossogobiusaglestes* Jordan & Seale, 1905: 798, fig. 16**

= *Psammogobiusbiocellatus* (Valenciennes, 1837).

**Holotype.**USNM 51948 (71.1), southern Negros Island, 1901.

**Remarks.** The original description or USNM record did not indicate the collection date.

**(503) *Gnatholepiscalliurus* Jordan & Seale, 1905: 796, fig. 14**

= *Arcygobiusbaliurus* (Valenciennes, 1837).

**Holotype.**USNM 51944 (63.5), southern Negros Island, 1901.

**Paratypes.**CAS-SU 9129 (1) and USNM 372624 (2), same locality with the holotype.

**Remarks.** Originally, USNM 51944 contained three specimens, but two were removed and designated as paratypes (USNM 372624). A photograph of the holotype is available in the USNM record (Smithsonian Museum Archive 2024).

**(504) *Gobiodonaoyagii* Shibukawa, Suzuki & Aizawa, 2013: 147, figs 2a–I, 3, 3A**

**Holotype.** NSMT-P 111422. Barasu, Iwomote-jima Island, Ryukyu Islands, Japan.

**Paratypes.** AMS I.22953-006 (2, 24.3–26.7), Cebu Aquatics, Cebu Island, 1980.

**(505) *Gobiodonfulvus* Herre, 1927c: 292**

**Syntypes.** BSMP 12560 (16, 18.0–36.0), Calapan, Mindoro Island, 17 Jan 1923.

**Remarks.** All type specimens were presumed destroyed.

**(506) *Gobiomorphusillotus* Herre, 1927c: 45, pl. 3 (fig. 4)**

= *Callogobiussclateri* (Steindachner, 1879).

**Holotype.** BSMP 11531 (29.0), Polillo Island, Luzon, Jul. 1920.

**Remarks.** The type specimen was lost (Kottelat 2013).

**(507) *Gobiosomainsignum* Herre, 1927c: 289, pl. 27 (fig. 3)**

= *Schismatogobiusinsignis* (Herre, 1927).

**Syntypes.** BSMP 12105 (19, 23.0–47.0 TL), Dumaguete River, Negros Island, 8 Mar. 1922.

**Remarks.** The type specimen was lost (Kottelat 2013).

**(508) *Gobiosomamarmoratum* Peters, 1868: 267**

= *Schismatogobiusmarmoratus* (Peters, 1868).

**Holotype.** ZMB 6756, Loquilócun, Samar Island.

**Remarks.** The original description did not indicate the collection date.

**(509) *Gobiosomapallida* Herre, 1934: 91**

**Holotype.**CAS-SU 28609 (20.5), Sitankai Island, Sulu Archipelago, Aug. 1931.

**Paratypes.**CAS-SU 16962 (3, 20.0–33.0 TL), same locality as holotype, 27 Aug. 1931.

**Remarks.** A photograph and a radiograph of the holotype are available in CAS’s record.

**(510) *Gobitrichinotusradiocularis* Fowler, 1943: 86, fig. 22**

**Holotype.**USNM 99549 (32.0), Malabang River, Eastern Illana Bay, southern Mindanao, 2 m, 21 May 1908.

**Paratype.**USNM 99550 (1, 21.0), same data as holotype.

**Remarks.** Radiographs of the holotype are available in the USNM record.

**(511) *Gobiusargulus* Peters, 1868: 266**

**Syntypes.** ZMB 5201 (2), coral reef near Paracali, Luzon.

**Remarks.** Unknown status in Fricke et al. (2024). Further study is needed to confirm its status. The original description did not indicate the collection date.

**(512) *Gobiusbothriorrhynchus* Herzenstein, 1896: 3**

= *Callogobiusbothriorrhynchus* (Herzenstein, 1896).

**Holotype.** ZIN 9684, Philippines.

**Remarks.** The original description did not provide a specific locality and collection date. The holotype and only specimen was in poor condition. It appears similar to at least one species of the *Callogobiushasseltii* syntypes, but cannot confirm with confidence because of its position (Delventhal and Mooi 2023).

**(513) *Gobiuscalderae* Evermann & Seale, 1906: 511, fig. 3**

= *Istigobiusornatus* (Rüppell, 1830).

**Holotype.**USNM 55625 (63.5), Caldera Bay, Zamboanga, Mindanao, 1904.

**Paratype.**MCZ 36018 [ex USNM 55625] (1, 40.0), same data as holotype.

**Remarks.** The original description mentioned four specimens (53.3–69.9) from the type locality including 53625 as the holotype. However, according to the USNM record, USNM 53625 is assigned to a different species, while USNM 55625 contains three specimens of *G.caldera*, indicating that these are syntypes. One of the specimens was sent to MCZ. A photograph of one of the type specimens is also available in the USNM record (Smithsonian Museum Archive 2024).

**(514) *Gobiuselmeri* Herre, 1940: 358, pl. 1**

= *Bathygobiuscocosensis* (Bleeker, 1854).

**Holotype.**CAS-SU 32989 (28.0), Tide pool at Nasugbu, Batangas, Luzon, 11 Dec. 1936.

**(515) *Gobiusobscuripinnis* Peters, 1868: 263**

= *Glossogobiusobscuripinnis* (Peters, 1868).

**Syntypes.**BMNH 1868.7.10.7–9 (3), MNHN 0000-6161 [ex ZMB in 1869] (3, 46.0–51.0) and ZMB 6498 (4, 81.0–86.0), Bicol River, Albay and Kolabos Brook at Daraga, Albay, Luzon.

**Remarks.** The original description did not indicate the collection date. BMNH 1868.7.10.7–9 was not found in NMH’s record. The sizes were inferred from Akihito and Meguro (1975) and Hoese and Allen (2009).

**(516) *Gobiuspanayensis* Jordan & Seale, 1907: 42, fig. 15**

= *Bathygobiuspanayensis* (Jordan & Seale, 1907).

**Holotype.**CAS-SU 9250 (60.0), Panay Island, 1900.

**Remarks.**Koumans (1940) provided a short description and taxonomic remark. A photograph and a radiograph of the holotype are available in the CAS record.

**(517) *Gobiusrufus* Marion de Procé, 1822: 132**

Manila Bay, Luzon.

**Remarks.** No type known. The original description did not indicate the collection date. An overlooked available name. A *species inquirenda* in Parenti (2021) and have unknown status in Fricke et al. (2024). Further study is needed to verify its status.

**(518) *Gobiusviganensis* Steindachner, 1893: 150**

= *Yongeichthysviganensis* (Steindachner, 1893).

**Syntypes.** NMW 30143 (1) and NMW 30144 (1), Vigan, Ilocos Sur, Luzon.

**Remarks.** The original description did not indicate the collection date.

**(519) *Gralleniarubrilineata* Allen & Erdmann, 2017: 38, figs 3D, 5C, 16–19**

**Holotype.** WAM P.34264-001 (male, 15.2), Ligpo Island, Anilao, Batangas, Luzon, 12–15 m, 4 Jun. 2014.

**Paratypes.**USNM 432520 (20, 9.6–15.1) and WAM P.34264-003 (55, 8.9–15.8), taken with the holotype.

**Remarks.**USNM 432520 was not found in the USNM record and possibly not yet deposited there.

**(520) *Herreolusphilippinus* Herre, 1945b: 14**

= *Parioglossusphilippinus* (Herre, 1945).

**Holotype.**CAS-SU 36812 (female, 24.0), Santa Maria, Zamboanga, Mindanao, 21 Sep. 1940.

**Paratypes.**CAS-SU 36813 (male, 21.0) and CAS-SU 36814 (3 females, 21.0–24.0), same data as holotype.

**Remarks.** A photograph and a radiograph of the holotype are available in the CAS record.

**(521) *Illanacacabet* Smith & Seale, 1906: 80, fig.**

= *Glossogobiusbicirrhosus* (Weber, 1894).

**Holotype.**USNM 55622 (male, 82.6), Rio Grande River, Mindanao, Oct. 1903.

**Paratypes.** BSMP 10603 [ex 4258] (1) and USNM 126396 [ex USBF 1484 and 4242] (3), same data as the holotype.

**Remarks.** The original description mentioned six specimens. Koumans (1940) provided a short description and taxonomic remark in his catalog. BSMP paratype was presumed destroyed.

**(522) *Intonsagobiuskuderi* Herre, 1943: 93**

= *Callogobiuskuderi* (Herre, 1943).

**Holotype.**CAS-SU 36815 (male, 35.0), coral in a lagoon amid a group of small isles ~ 8 miles west of Jolo Island, Sulu Archipelago, 16 Sep. 1940.

**Paratypes.**CAS-SU 36816 (1 male, 31.0 + 2 females, 28.0–34.0), same data as holotype.

**Remarks.** A photograph and a radiograph of the holotype are available in the CAS record.

**(523) *Intonsagobiusvanclevei* Herre, 1950: 73**

= *Callogobiusvanclevei* (Herre, 1950).

**Holotype.**USNM 202513 [ex UW 7577] (female, 39.0), Dumaguete, Negros Island, 8 Jul. 1948.

**Remarks.** A radiograph of the holotype is available in the USNM record.

**(524) *Itbayanuda* Herre, 1927c: 288, pl. 23 (fig. 2)**

= *Kelloggellacardinalis* Jordan & Seale, 1906.

**Holotype.** BSMP 13079 (18.8), Itbayat Island, north of Luzon, 21 Nov 1921.

**Remarks.** The type specimen was presumed destroyed.

**(525) *Lophogobiusnonatoae* Ablan, 1940: 376, pl. 2**

= *Cristatogobiusnonatoae* (Ablan, 1940).

**Holotype.** BSMP 31129, fishponds in Dagupan, Pangasinan, Luzon.

**Paratypes.** BSMP (49, 18.0–49.0), same data as holotype.

**Remarks.** The original description did not indicate the collection date of the type specimens. All type specimens were lost (Kottelat 2013).

**(526) *Macgregorellabravoi* Herre, 1940: 363, pl. 5**

= *Gobiopsisbravoi* (Herre, 1940).

**Holotype.**CAS-SU 33120 (30.0), Nasugbu, Batangas, Luzon, 11 Dec. 1936.

**Paratypes.**CAS-SU 33121 (4, 24.0–28.0) and CAS 28066 (1, 32.0), same data as holotype.

**Remarks.** A photograph and a radiograph of the holotype are available in the CAS record.

**(527) *Macgregorellaintonsa* Herre, 1927c: 100, pl. 7 (fig. 2)**

= *Callogobiusokinawae* (Snyder, 1908).

**Syntypes.** BSMP 3575 [= BSMP 12807 and BSMP 25374] (1 male, 52.0 + 1 female, 40.5), Near Saub, south of Cotabato, Mindanao; BMNH 1933.3.11.593–598 (6), Dumaguete, Negros Island.

**Other catalog number.**BMNH 1933.3.11.593–598 (NHMUK:ecatalogue:2511762).

**Remarks.**Koumans (1940) provided a taxonomic remark. BSMP specimens were lost (Kottelat 2013).

**(528) *Macgregorellamoroana* Seale, 1910: 533**

= *Callogobiushasseltii* (Bleeker, 1851).

**Holotype.** BSMP 3575 (54.0), Jolo Island, Sulu Archipelago, 30 Apr. 1907.

**Remarks.**Koumans (1940) provided a taxonomic remark. The type specimen was lost (Kottelat 2013).

**(529) *Mangarinuswaterousi* Herre, 1943: 94**

**Holotype.**CAS-SU 36817 (33.5), mangrove swamp at Hacienda Waterous, Mangarin Bay, Mindoro Island, 20 Jul. 1940.

**Paratypes.**CAS-SU 36818 (3, 32.0–33.0), same data as holotype.

**Remarks.** A photograph and a radiograph of the holotype are available in the CAS record.

**(530) *Mapomearnsi* Evermann & Seale, 1906: 510 fig. 2**

= *Bathygobiuscyclopterus* (Valenciennes, 1837).

**Holotype.**USNM 55624 (63.5), Zamboanga, Mindanao, 1994.

**Paratype.** Bur. Fish. 1495 (1, 29.4), same locality as the holotype.

**Remarks.** The original description did not indicate the collection date. A radiograph of the holotype is available in the USNM record.

**(531) *Marscaeruleo-maculatus* Herre, 1933: 22**

= *Cryptocentruscaeruleomaculatus* (Herre, 1933).

**Holotype.**CAS-SU 25502 (male, 33.0), tidal flats at Jolo Island, Sulu Archipelago, 2 Aug. 1931.

**Paratypes.**CAS-SU 16959 (5, 23.0–37.0), same data as holotype.

**Remarks.**Koumans (1940) provided a taxonomic remark. A photograph and a radiograph of the holotype are available in the CAS record.

**(532) *Marshaydeni* Herre, 1936b: 363, pl. 2 (fig. 6)**

= *Cryptocentrushaydeni* (Herre, 1936).

**Holotype.**CAS-SU 30962 (32.0), tide flats at Bais, Negros Island, 23 Nov. 1933.

**Remarks.** A photograph and a radiograph of the holotype are available in the CAS record.

**(533) *Mindorogobiuslopezi* Herre, 1945b: 13**

= *Acentrogobiusmoloanus* (Herre, 1927).

**Holotype.**CAS-SU 36822 (27.0), mangrove swamp at Hacienda Waterous, Mangarin Bay, Mindoro Island, 20–22 Jul. 1940.

**Paratypes.**CAS-SU 36823 (2, 22.0–26.0), same data as holotype.

**Remarks.** A photograph and a radiograph of the holotype are available in the CAS record.

**(534) *Myersinamacrostoma* Herre, 1934: 90**

**Holotype.**CAS-SU 26770 (25.0 TL), reef in Culion Harbor, Culion Island, Palawan, 3 May 1931.

**Remarks.** A photograph and a radiograph of the holotype are available in the CAS record.

**(535) *Oplopomusvergens* Jordan & Seale, 1907: 44, fig. 17**

= *Oplopomuscaninoides* (Bleeker, 1852).

**Holotype.**USNM 93209, Cavite, Luzon.

**Paratypes.**CAS-SU 9256 (1), CAS-SU 20100 (1) and USNM 53072 (2) (69.9–81.3), same locality as holotype.

**Remarks.**Koumans (1940) noted that USNM 93209 contained two specimens. On the bottle labels, 53072 and 53071 co-type was indicated. However, Böhlke’s (1953) catalog stated that USNM 93209 was mistakenly recorded as 53071 which was preoccupied by a type of *Blenniusthysanius* (Smithsonian Museum Archive 2024). The original description, USNM, or CAS records did not indicate the collection date of the holotype. Radiographs of the holotype are available.

**(536) *Oxymetoponcyanoctenosum* Klausewitz & Condé, 1981: 71, figs 1, 10–17, 19e**

**Holotype.** SMF 15390, probably from off Cebu Island.

**Remarks.** The original description did not indicate the collection date.

**(537) *Oxyurichthysviridis* Herre, 1927c: 260**

= *Oxyurichthysophthalmonema* (Bleeker, 1856).

**Syntypes.** BSMP 12011 and BSMP 12111–13 (4, 72.0–86.0), Manila market, Luzon.

**Remarks.** The original description did not indicate the collection date. Koumans (1940) provided a taxonomic remark. All type specimens were lost (Kottelat 2013).

**(538) *Oxyurichthysvisayanus* Herre, 1927c: 254**

= *Oxyurichthyslonchotus* (Jenkins, 1903).

**Syntypes.** BSMP 12405 and BSMP 26096–106 (12, 52.0–75.0), Cebu Island, 16 Sep. 1925.

**Remarks.**Koumans (1940) provided a taxonomic remark. All type specimens were lost (Kottelat 2013).

**(539) *Parioglossusnudus* Rennis & Hoese, 1985: 180, figs 10, 24**

**Holotype.**CAS 36930. Auluptagel Island, Palau Islands.

**Paratypes.** LICPP 1977056 (1, 17.0), Malipano, Samal Island, southern Mindanao, 5 m; USNM 261553 (1, 22.0), SP 78-11, just off Bonbonon Point, southern tip of Negros Island, 0–12 m, 13 May 1978; USNM 261557 (1, 19.0), SP 78-38, west of Balicasag Island, Bohol, 0–24 m, 10 Jun. 1978.

**Remarks.** Based on the USNM record, five specimens of USNM 261557 were exchanged to Charleston Museum.

**(540) *Pleurogobiusboulengeri* Seale, 1910: 536**

= *Priolepiscincta* (Regan, 1908).

**Holotype.** BSMP 5505 (35.0), Puerto Princesa, Palawan Island.

**Remarks.** The original description did not indicate the collection date. The type specimen was presumed destroyed.

**(541) *Pleurosicyamicheli* Fourmanoir, 1971: 499, fig. 8**

**Neotype.** AMS I.21918-071 (female, 17.5) Caban Island, Verde Island Passage, southwest Luzon, 1980.

**Remarks.** The original genus was spelled *Pluerosycia*. The original holotype was not found at MNHN and was presumed lost (Bauchot et al. 1991). A neotype was designated by Larson (1990).

**(542) *Pogonoculiuszebra* Fowler, 1938: 134**

= *Ptereleotriszebra* (Fowler, 1938).

**Holotype.**USNM 99048 (95.0), Dasol Bay, Pangasinan, Luzon, 8 May 1909.

**Remarks.** A radiograph of the holotype is available in the USNM record.

**(543) *Priolepisagrena* Winterbottom & Burridge, 1993b: 2057, figs 1–3**

**Holotype.** ROM 53204 (24.8), 09°26.20'N, 123°23.10'E, west of Sumilon Island, Cebu, 15–26 m, 20 May 1987.

**Paratype.** AMS I.21918-041 (1 female, 25.3), Caban Island, Luzon, 25 Apr. 1980.

**(544) *Priolepisfallacincta* Winterbottom & Burridge, 1992: 1940, figs 1, 6, 7**

**Holotype.** ROM 53146 (24.4), 09°36'54"N, 123°11'06"E, mouth of Bais Bay, Negros Island, 24.4–36.6 m, 15 May 1987.

**Paratypes.** ROM 53147 (2, 8.4–8.9), mouth of Bais Bay, Negros Island, 3–5.2 m, 17 May 1987; USNM 135750 (1, 22.8), Mompog Island, 3 Mar. 1909; USNM 308388 (1, 13.3), north of Maloh, near Giligaon, Negros Island, 0–2 m, 26 Apr. 1979; USNM 313358 (1, 12.6), west of Bonbonon, southern tip of Negros Island, 0–1 m, 2 May 1978.

**(545) *Priolepiskappa* Winterbottom & Burridge, 1993a: 501, figs 9, 10**

**Holotype.** ROM 58052. Anjouan Island, Comoro Islands.

**Paratypes.**USNM 293349 (1, 15.6), Y’Ami Island, Batanes, 12–18 m, 25 Apr. 1987.

**(546)] *Priolepisvexilla* Winterbottom & Burridge, 1993a: 511, figs 23, 24**

**Holotype.** BPBM 34187. Cape Batu Badiri, Ambon Bay, Molucca Islands, Indonesia.

**Paratypes.**USNM 308387 [ex USNM 261974] (1, 14.0), LK 79-7, west side of Solino Island, north of Mindanao, 0–5 m, 3 May 1979.

**(547) *Pseudogobiusverticalis* Larson & Hammer, 2021: 74, fig. 29**

**Holotype.** ZRC 61150 [ex NTM S.15344-016]. Sungei Buloh, Singapore.

**Paratypes.**CAS-SU 69916 (36, 18.0–22.0), Coron, Busuanga Island, Palawan, 22 Jun. 1940.

**(548) *Ptereleotrisdispersus* Herre, 1927c: 83, pl. 6 (fig. 3)**

= *Ptereleotrisevides* (Jordan & Hubbs, 1925).

**Holotype.** BSMP (81.0), Santo Domingo de Basco, Batan Island, Batanes, Luzon.

**Paratype.** BSMP (1, 54.0), south of Cotabato, Mindanao.

**Remarks.** All type specimens are presumed destroyed.

**(549) *Ptereleotrisgrammica* Randall & Lubbock, 1982: 41, figs 1–3**

**Holotype.** BPBM 21159. Sesoko Island, Ryukyu Islands, Japan.

**Paratype.** NSMT-P 21080 (1, 86.0), Manila.

**Remarks.** The original description did not indicate the collection date.

**(550) *Ptereleotriskallista* Randall & Suzuki, 2008: 96, figs 4, 5**

**Holotype.** BPBM 40881, from aquarium fish supplies in Manila, Luzon.

**Paratype.** NSMT P 79557 (1), Luzon, 1 Dec. 2007.

**Remarks.** The original description did not indicate the collection date.

**(551) *Pteroculiopsguttatus* Fowler, 1938: 133**

= *Amblyeleotrisguttata* (Fowler, 1938).

**Holotype.**USNM 99045 (69.0), Port Banalakan, Marinduque Island, 4–7 m, 23 Feb. 1909.

**Remarks.** A radiograph of the holotype is available in the USNM record.

**(552) *Rhinogobiusdecoratus* Herre, 1927c: 181, pl. 13 (fig. 3)**

= *Istigobiusdecoratus* (Herre, 1927).

**Neotype.**USNM 254289, 09°04.42'N, 123°16.08'E, west side of Apo Island, Bohol Sea, 0–6.1 m, 6 Jun. 1978.

**Remarks.** The original syntypes (BSMP 13056 [1], 26881–82 [1, 1]) were destroyed, but seen by Koumans (1940) who provided a taxonomic remark in his work. A neotype was designated by Murdy and Hoese (1985).

**(553) *Rhinogobiusflavoventris* Herre, 1927b: 276, pl. 3 (fig. 1)**

= *Silhouetteaflavoventris* (Herre, 1927).

**Syntypes.** BSMP (37, 25.0–37.0), Taal Lake, Luzon.

**Remarks.** The original description did not indicate the collection date. All type specimens were lost (Kottelat 2013).

**(554) *Rhinogobiuslungi* Jordan & Seale, 1907: 41, fig. 13**

= *Yongeichthysnebulosus* (Forsskål, 1775).

**Holotype.**USNM 53069, Iloilo, Panay Island.

**Paratype.**CAS-SU 9248 (1), Panay Island, 1900.

**Remarks.** The original description mentioned three specimens measuring 38.1–99.1. USNM 53069 contained two specimens and the locality and number of specimens were added based on jar labels. The USNM record indicated USNM 53069 as syntypes with 2 specimens (Smithsonian Museum Archive 2024).

**(555) *Rhinogobiusmultifasciatus* Herre, 1927c: 190, pl. 14 (fig. 1)**

= *Acentrogobiusmultifasciatus* (Herre, 1927).

**Syntypes.** BSMP (65, 25.0–54.0), Jaro River, Iloilo, Panay Island, 23 Jul. 1925.

**Remarks.** All type specimens were lost (Kottelat 2013).

**(556) *Rhinogobiusocyurus* Jordan & Seale, 1907: 42, fig. 14**

= *Drombusocyurus* (Jordan & Seale, 1907).

**Holotype.**CAS-SU 9249 [=USNM 53070] (male, 30.7), Cavite, Luzon, 1 Jun. 1900.

**Remarks.** Probably two catalog numbers were assigned to one specimen. The original description listed USNM 53070, but some text mentioned two catalog numbers (CAS-SU 9249 and USNM 53070). The USNM 53070 is not found in the USNM record.

**(557) *Rhinogobiusperpusillus* Seale, 1910: 534**

= *Amblygobiusdecussatus* (Bleeker, 1855).

**Holotype.** BSMP 4022 (45.0), Zamboanga, Mindanao.

**Paratypes.** BSMP 1276 and BSMP 5106.

**Remarks.** The original description did not indicate the collection dates and the locality of paratypes. All type specimens were lost (Kottelat 2013).

**(558) *Rhinogobiussuluensis* Herre, 1927c: 193, pl. 14 (fig. 3)**

= *Yongeichthyssuluensis* (Herre, 1927).

**Syntypes.** BSMP (6, 25.0-35.0), Bungau, Tawi Tawi Group, Sulu Archipelago, 8 Jun. 1921.

**Remarks.**Koumans (1940) provided a taxonomic remark. All type specimens were lost (Kottelat 2013).

**(559) *Schismatogobiusroxasi* Herre, 1936b: 362, pl. 2 (fig. 5)**

= *Schismatogobiusinsignis* (Herre, 1927).

**Holotype.**CAS-SU 30968 (44.0), San Jose, Antique, Panay Island, Feb. 1936.

**Remarks.**Koumans (1940) provided a taxonomic remark.

**(560) *Schismatogobiussaurii* Keith, Lord, Hadiaty & Hubert in Keith, Lord, Darhuddin, Limmon, Sukmono, Hadiaty and Hubert, 2017: 198, figs 2–3**

**Holotype.** MZB 23794. Tukad Banyuraras, West Bali, Indonesia.

**Paratype.**MNHN 2016-0299 (1 female, 31.2), Alegre River, Panay Island.

**Remarks.** The original description did not indicate the collection date.

**(561) *Smilogobiusinexplicatus* Herre, 1934: 88**

= *Cryptocentrusinexplicatus* (Herre, 1934).

**Holotype.**CAS-SU 25500, puddle on the Sitankai reef, Sulu Archipelago, 10 Aug. 1931.

**Paratype.**CAS-SU 16957 (1), same data as holotype.

**Remarks.**Koumans (1940) provided a taxonomic remark and mentioned two specimens measuring 53.0–71.0 TL as types for CAS-SU 25500. Böhlke (1953) mentioned CAS-SU 16957 as a paratype. A photograph and a radiograph of the holotype are available in CAS’ record.

**(562) *Smilogobiusobliquus* Herre, 1934: 89**

= *Cryptocentrusleptocephalus* Bleeker, 1876.

**Holotype.**CAS-SU 25501 (52.0), reef in Culion Harbor, Palawan, 3 May 1931.

**Paratypes.**CAS-SU 16958 (6), same data as holotype.

**Remarks.**Koumans (1940) mentioned seven types and paratypes measuring 30.0–67.0 but did not specify the size of the type specimen and mentioned only one catalog number (CAS-SU 25501). Böhlke (1953) mentioned CAS-SU 16958 as paratypes with six specimens with the same data as the holotype. A photograph and a radiograph of the holotype are available in CAS’ record.

**(563) *Stonogobiopsnematodes* Hoese & Randall, 1982: 13, pl. 3 (B–C); figs 1, 4**

**Holotype.** BPBM 27854 (female, 37.2), off south of Seas Resort Hotel, Dumaguete, Negros Island, 20 m, 5 Jun. 1981.

**(564) *Tomiyamichthysgomezi* Allen & Erdmann, 2012: 1174, figs 1–5**

**Holotype.** WAM P.32998-004 (male, 44.3), 11°21.10'N, 119°31.08'E, Rawis, El Nido, Palawan Island, 13 m, 19 Jun. 2008.

**Paratype.** WAM P.32998-011 (female, 37.4), taken with holotype.

**(565) *Trimmaagrena* Winterbottom & Chen, 2004: 103, fig. 1**

**Holotype.** ROM 53126 (female, 20.8), south of Sumilon Island, Cebu, 3–10.7 m, 20 May 1987.

**Paratypes.** ROM 74054 (6, 12.6–23.8), taken with holotype; ROM 49216 (6, 16.9–29.3), northwest of Sumilon Island, Cebu, 12–18 m, 11 Aug. 1985; ROM 1153CS, west of Tonga Point, Siquijor Island, 0.91–3.7 m, 9 May 1987; ROM 1157CS, just inshore of RW87-36, Bohol Strait, 3.7–7.6 m, 21 May 1987.

**(566) *Trimmaannosum* Winterbottom, 2003: 2, figs 1–2**

**Holotype.** ROM 73126 [ex ROM 40061]. Cagilai Island off Ovalau Island, Viti Levu, Bau Waters, Fiji.

**Paratypes.** ROM 49225 (2, 16.2–20.9), northwest of Sumilon Island, Cebu, 0–2 m, 11 Aug. 1985; ROM 218 53149 (4, 16.5–21.5), Tonga Point, Siquijor Island, 1–3.7 m, 9 May 1987; ROM 53151 (14, 9.5–16.5), mouth of Bais Bay, Negros Island, 3.0–5.2 m, 17 May 1987.

**(567) *Trimmabenjamini* Winterbottom, 1996: 57, figs 1–4**

**Holotype.** ROM 53038 (male, 21.2), 09°12.28'N, 123°27.23'E, Tonga Point, Siquijor Island, 22 May 1987.

**Paratypes.** ROM 53025–37 (2, 4, 2, 11, 7, 8, 4, 3, 10, 36, 26, 4, 35), same locality as holotype 7–22 May 1987; ROM 49008 (1), north of Tambuli Beach Resort, Mactan Island, Cebu, 9 Aug. 1985; ROM 49227 (3), off Hudson Beach, Mactan Island, Cebu, 7 Aug. 1985; ROM 49228 (17), drop-off near Point, south of Hudson Beach, Cebu, 8 Aug. 1985; ROM 53033 (10) and ROM 53034 (36), mouth of Bais Bay, Negros Island, 15–17 May 1987; ROM 53035 (26), north of Bais Bay near main channel, Negros Island, 19 May 1987; ROM 53036 (4) and ROM 53037 (35), west of Sumilon Island, Cebu, 20–21 May 1987; ROM 49226 (2), northwest of Sumilon Island, Cebu, 11 Aug. 1985; ROM 53219 (76), same locality as precedence, 20 May 1987; USNM 26490 (10), off east of Mactan Island, Cebu, 0–30 m, 3 Jun. 1978 ; USNM 264696 (5), south of San Juan, Siquijor Island, 0–10.7 m, 10 May 1978.

**Remarks.** Four specimens of USNM 26490 were removed and placed in Other catalog numbers.

**(568) *Trimmacana* Winterbottom, 2004: 8, figs 1–3, 4A–B, 5A**

**Holotype.** ROM 73776 (male, 22.3), 09°12.28'N, 123°27.23'E, Tonga Point, Siquijor Island, 22 May 1987.

**Paratypes.**MCZ 164729 (1, 21.5), ROM 53070–73 (1, 1, 7, 5) and ROM 53076 (3), same locality as holotype, 8–22 May 1987; ROM 49215 (2), drop-off near Point, south of Hudson Beach, Cebu, 8 Aug. 1985; USNM 264713 (1), Caceres Reef near Huisan Point, east of Cebu Island, 30 m, 18 May 1979; USNM 313351 (6), off Buyong Beach, Mactan Island, Cebu, 30 m, 3 Jun. 1978; USNM 313528 (6), northwest of Pescador Island, Cebu, 18–24 m, 7 May 1979; USNM 313538 (4), 2 km west of Siquijor, Siquijor Island, 24–30 m, 14 May 1979; USNM 313545 (1), Balicasag Island, Bohol, 24 m, 10 Jun. 1978.

**Remarks.**USNM 313545 was removed from USNM 4674 (Smithsonian Museum Archive 2024).

**(569) *Trimmahalonevum* Winterbottom, 2000: 62, fig. 3**

**Holotype.** ROM 72215. Gippiwall Point, Normanby Island, Papua New Guinea.

**Paratypes.**USNM 243184 (14, 10.5–22.8), LK 79-16, 1 km west of Larena, Siquijor Island, 26–30 m, 15 May 1979; USNM 243923 (47, 9.2–18.3), SP 78-45, east of Bais, Negros Island, 0–37 m, 17 Jun. 1978; USNM 244115 (10, 11.2–20.1), SP 78-10, off Bonbonon Point at southern tip of Negros Island, 0–18.3 m, 13 May 1978; USNM 262621 (1, 12.2), SP 78-46, Ajong, Negros Island, 0–2 m, 18 Jun. 1978; USNM 264605 (8), SP 78-36, southeast of Apo Island, Negros, 0–39.5 m, 7 Jun. 1978; USNM 264712 (3), JL-6, southwest of Apo Island, Negros, 29–37 m, 11 May 1979; USNM 295242 (28), Anilao, Batangas, Luzon, 22 m, 26 Apr. 1980; USNM 313352 [ex USNM 243497] (1), SP 78-30, east of Mactan Island, Cebu, 0–30 m, 3 Jun. 1978; USNM 313356 [ex USNM 243939] (11, 10.2–19.1), SP 78-39, west of Balicasag Island, Bohol, 0–41 m, 11 Jun. 1978; USNM 313550 [ex USNM 264679] (4, 15.9–18.8), SP 78-29, east of Mactan Island, Cebu, 0–40 m, 2 Jun. 1978; USNM 313558 [ex USNM 264703] (1, 15.5), SP 78-41, off southwest tip of Pamilacan Island, Bohol, 0–33.5 m, 12 Jun. 1978; USNM 313564 [ex USNM 295228] (1, 16.5), Caban Island, Batangas, Luzon, 0–1 m, 25 Apr. 1980.

**Remarks.** The USNM record shows that USNM 243184 specimens were combined with USNM 313541 and 313531; USNM 243923 combined with USNM 262618; USNM 244115 combined with USNM 313553 and 246364; USNM 264605 3 of 11 removed to USNM 313535, the description says 9 specimens; USNM 264712 combined with USNM 313536; USNM 295242 in poor condition; combined with USNM 295322, 295284, 295292, 295355, and 295287; USNM 313352 description says two specimens.

**(570) *Trimmanasa* Winterbottom, 2005: 34, figs 3B, 4B, 5–6**

**Holotype.** ROM 53043 (female, 19.7), 09°12.00'N, 123°28.00'E, Tonga Point, Siquijor Island, 8 May 1987.

**Paratypes.** ROM 53044–46 (1, 3, 1) and ROM 60243 (1), same locality as holotype, 8–14 May 1987; ROM 49229 (10), northwest of Sumilon Island, Cebu, 11 Aug 1985; ROM 53047 (2) and ROM 53048 (2), Bais Bay, Negros Island, 15–17 May 1987; USNM 243945 (2), east of Bais, Negros Island, 0–37 m, 17 Jun. 1978; USNM 246366 (6) and USNM 262620 (1), off southwest tip of Pamilacan Island, Bohol, 0–24 m, 12 Jun. 1978; USNM 246368 (4), off Buyong Beach, Mactan Island, Cebu, 0–30 m, 3 Jun. 1978; USNM 246372 (3), east of Mactan Island, Cebu, 0–40 m, 2 Jun 1978; USNM 243917 (5) and USNM 246772 (21), Balicasag Island, Bohol, 0–41 m, 10–11 Jun. 1978; USNM 263538 (1), northwest Pescador Island, Cebu, 30 m, 8 May 1979; USNM 264603 (9), Caceres Reef near Huisan Point, east of Cebu, 24–30 m, 18 May 1979.

**(571) *Trimmastobbsi* Winterbottom, 2001: 20, figs 1–2**

**Holotype.** ROM 72488. Port de Goro, New Caledonia.

**Paratypes.**USNM 264521 (3), SP 78-36, southeast of Apo Island, Negros, 0–39.5 m, 7 Jun. 1978.

**(572) *Trimmatommacropodus* Winterbottom, 1989: 2404, figs 1, 2, 7**

**Holotype.** AMS I.19456-113. Lizard Island, Great Barrier Reef, Australia.

**Paratype.** ROM 1179CS (1, 12.7), Tagauayan Island, Cuyo, Palawan, 0–2.5 m, 25 May 1978.

**(573) *Trimmatomsagma* Winterbottom, 1989: 2406, figs 3, 4, 7**

**Holotype.** ROM 53118 (female, 12.2), off the mouth of Bais Bay, Negros Is, 24–36 m, 15 May 1987.

**Paratypes.** ROM 53117 (1, 12.2), Tonga Point, Siquijor Island, 15–21 m, 12 May 1987; USNM 243908 (1, 15.1), southeast of Apo Island, Negros, 0–30 m, 6 Jun. 1978; USNM 263486 (2, 8.8–12.1) and USNM 264737 (12, 8.1–17.6), Pescador Island, Cebu, 18–30 m, 7–8 May 1979; USNM263498 (1, 10.2), Caceres reef near Huisan Point, Cebu, 24–30 m, 18 May 1979; USNM 263513 (1, 13.3), west of Balicasag Island, Bohol, 0–24 m, 10 Jun. 1978.

**(574) *Valencienneabella* Hoese & Larson, 1994: 15, pl. I.**

**Holotype.** AMS I.24821-001 [ex BPBM 20806]. Sesoko Island, Ryukyu Islands, Japan.

**Paratypes.** URM 8127 (1, 44.0), El Nido, Palawan Island, 6–25 Mar. 1983; USNM 264411 (1, 24.0), reef 500 m west of White Beach, Puerto Princesa Bay, Palawan Island, 32 m, 14 Nov. 1979.

**(575) *Valencienneaparva* Hoese & Larson, 1994: 37, pls 3A, 5H**

**Holotype.** AMS I.19108-083. Lizard Island Lagoon, Queensland, Australia.

**Paratypes.**USNM 168231 (1, 26.0), Canmahala Bay, Ragay Gulf, south of Luzon, 1 m, 11 Mar. 1909; USNM 258664 [ex USNM 161053] (2, 28.9–42.0), same locality and collection date as preceding, 1–9 m.

**(576) *Vanderhorstianobilis* Allen & Randall, 2006: 40, figs 1–4**

**Holotype.** WAM P.31402 (43.0), Halsey Harbour, Culion Island, Palawan.

**Remarks.** The original description did not indicate the collection date.

**(577) *Yabotichthysnocturnus* Herre, 1945a: 3**

= *Amblygobiusnocturnus* (Herre, 1945).

**Holotype.**CAS-SU 36828 (male, 38.0), off Yabot’s Camp, near San José, Busuanga Island, Palawan, 27 Jun. 1940.

**Paratypes.**CAS-SU 36829 (20, 23.0–34.0) and USNM 123652 [ex CAS-SU 36829] (4), same data as holotype.

**Remarks.** A photograph and a radiograph of the holotype are available in the CAS record.

#### ﻿﻿ORDER MUGILIFORMES (59)

##### Family Mugilidae (291)

**(578) *Mugilbanksi* Seale, 1910: 501, pl. 5**

= *Crenimugilheterocheilos* (Bleeker, 1855).

**Holotype.** BSMP 1412 (190.0), Siquijor Island, 7 Sep. 1908.

**Remarks.** The type specimen was presumed destroyed.

**(579) *Mugiljoloensis* Seale, 1910: 500, pl. 4**

= *Plicomugillabiosus* (Valenciennes, 1836).

**Holotype.** BSMP 2379 (125.0), Jolo Island, Sulu Archipelago, Feb. 1908.

**Remarks.** The type specimen was presumed destroyed.

**(580) *Mugillepidopterus* Fowler, 1918: 9, fig. 4**

= *Planilizasubviridis* (Valenciennes, 1836).

**Holotype.**ANSP 47483 (206.0), Philippines.

**Remarks.** The original description or ANSP did not provide a specific locality and collection date. Photographs and radiographs are available in ANSP record.

**(581) *Mugilogilbyi* Fowler, 1918: 5, fig. 2**

= *Planilizasubviridis* (Valenciennes, 1836).

**Holotype.**ANSP 47479 (210.0), Philippines.

**Paratype.**ANSP 47480 (1, 205.0), same as holotype.

**Remarks.** The original description or ANSP did not provide a specific locality and collection date.

**(582) *Mugilphilippinus* Fowler, 1918: 7, fig. 3**

= *Planilizasubviridis* (Valenciennes, 1836).

**Holotype.**ANSP 47481 (347.0), Philippines.

**Paratype.**ANSP 47482 (1, 254.0), same as holotype.

**Remarks.** The original description or ANSP did not provide a specific locality and collection date.

**(583) *Mugilruthveni* Fowler, 1918: 3, fig. 1**

= *Planilizasubviridis* (Valenciennes, 1836).

**Holotype.**ANSP 47478 (240.0), Philippines.

**Remarks.** The original description or ANSP did not provide a specific locality and collection date. Photographs are available in ANSP’s record.

**(584) *Myxusphilippinus* Roxas, 1934: 424, pl. 1 (fig. 9), pl. 2 (fig. 1)**

= *Planilizalauvergnii* (Eydoux & Souleyet, 1850).

**Holotype.** BSMP 28473, Lumbucan Island, near Balabac Island, Palawan.

**Paratypes.** BSMP 28469 (1).

**Remarks.** The original description did not indicate the collection date. All type specimens were lost (Kottelat 2013).

#### ﻿﻿ORDER CICHLIFORMES (60)

##### Family Pholidichthyidae (293)

**(585) *Brotulophisargentistriatus* Kaup, 1858: 93**

= *Pholidichthysleucotaenia* Bleeker, 1856.

**Holotype.**MNHN 1994-0574 (108.0), off south of Sulu Island, Sulu Archipelago.

#### ﻿﻿ORDER BLENNIIFORMES (61)

##### Family Tripterygiidae (294)

**(586) *Ceratobregmahelenae* Holleman, 1987: 175, fig. 2**

**Holotype.** WAM P.26098-012. Shipwreck on northern coast, Christmas Island.

**Paratypes.**USNM 280191 (2, 21.3–26.4), Cuyo Island, Palawan, 0–2 m, 25 May 1978; USNM280190 (3, 26.9–31.0), west of Siquijor Island, 0–11 m, 1 May 1978.

**Remarks.** The original description mentioned two specimens for USNM 280191, but four specimens were found in the lot, including 1 cleared and stained specimen.

**(587) *Enneapterygiusbahasa* Fricke, 1997: 170, fig. 29**

**Holotype.**USNM 259168. Heron Island, Queensland, Australia.

**Paratypes.**USNM 259129 (31, 17.2–22.2), Cocoro Island, Cuyo, Palawan, 0–3 m, 25 May 1978.

**(588) *Enneapterygiusfuscoventer* Fricke, 1997: 210, fig. 38**

**Holotype.**USNM 259131 (male, 22.8), 10°55.08'N, 121°02.05'E, Putic Island, northwest of Cuyo Island, Palawan, 0–4.5 m, 2 May 1978.

**Paratypes.** SMNS 19172 (2, 21.4–22.2) and USNM 345509 [ex USNM 259131] (15, 21.1–22.8) same data as holotype; USNM 259136 (1 male, 22.2), Cocoro Island, Cuyo, Palawan, 25 May 1978; USNM 293711 (1 male, 20.5), Songsong Bay, Batan Island, Batanes, 1.5–3 m 2 May 1987; USNM 295566 (9, 18.0–22.6), near Desquid Point, Balugan Bay, Batan Island, Batanes, 0–3 m, 23 Apr. 1987.

**Remarks.** The holotype originally was mixed with 18 specimens, and 17 specimens were removed to USNM 345509 which became paratypes. A photograph and radiograph of the holotype are available in the USNM record.

**(589) *Enneapterygiusolivaceus* Dewa, Tashiro & Motomura, 2023: 334, figs 1–3, 5a**

**Holotype.** KAUM-I 101347. Off Shinaha Beach, Yoron-jima Island, Amami Islands, Japan.

**Paratype.**USNM 273903 (1 female, 16.7), 09°03.10'N, 122°59.70'E Maloh, Negros Island, 18 May 1979; WAM P.31397-012 (1 male, 20.3), 12°06.00'N, 119°51.00'E south of Talampulan Island, Calamian Islands, Palawan, 3–5 m, 11 Feb. 1998.

**(590) *Enneapterygiuspallidoserialis* Fricke, 1997: 264, figs 54–55**

**Holotype.**USNM 279812 (male, 22), 10°55.08'N, 121°02.05'E, Putic Island, northwest of Cuyo Island, Palawan, 0–4.5 m, 2 May 1978.

**Paratypes.** SMNS 19171 (2, 21.8–23.7), USNM 227494 [ex USNM 222323] (1, 21.6), USNM 258845 (89, 16.9–25.7) and USNM 345513 [ex USNM 279812] (218, 14.1–23.8), same data as holotype; USNM 287086 (7, 9.9–25.0), southwest of Siayan Island, Batanes, 1–4 m, 8 May 1986.

**Remarks.** The holotype was originally mixed with 233 specimens and 220 specimens were removed to USNM 345513 which became paratypes. One specimen was re-identified as *E.tutuilae* and re-cataloged as USNM 345514, and 11 specimens were re-identified as *Helcogrammafuscipectoralis* and re-cataloged to USNM 345515. A photograph and radiograph of the holotype are available in the USNM record. Two specimens of USNM 345513 were donated to SMNS.

**(591) *Enneapterygiussimilis* Fricke, 1997: 326, fig. 68**

**Holotype.**USNM 293726 (male, 24.6), 20°20.17'N, 121°49.17'E, Ibahos Island, Batanes, Luzon, 2–5 m, 3 May 1987.

**Paratypes.** SMNS 18699 (2, 22.8–23.7) and USNM 344099 [ex USNM 293726] (21, 16.2–25.9), same data as holotype; USNM 273895 (1 male, 21.2) and USNM 273898 (1, 15.2–21.4), west of Solino Island, Zamboanga, Mindanao, 0–5 m, 3–4 May 1979; USNM 273897 (12, 13.8–21.8), Maloh, Negros Island, 0 m, 18 May 1979; USNM 273906 (14, 16.9–21.8), same locality as USNM 273897, 0–3 m, 24 Apr. 1979; USNM 273907 (male, 19.5), near Senora Asuncion, north of Dumaguete, Negros Island, 0–1.6, 23 Apr. 1979; USNM 273915 (41, 13.3–22.7), west of Alibay, Aliguay Island, Zamboanga, Mindanao, 0–3 m, 2 May 1979; USNM 293407 (5, 20.1–22.9), approx. 0.25 mile southeast of Djojo Point, Batan Island, Batanes, 2 May 1987; USNM 293784 (9, 16.4–21.5), Chawa Point, Batan Island, Batanes, 9–12 m, 1 May 1987; USNM 293793 (3, 14.3–25.6), Y’Ami Island, Batanes, 2–5 m, 26 Apr. 1987; USNM 293894 (5, 19.2–26.4), Mahatae, Batan Island, Batanes, 0–6.5 m, 22 Apr. 1987; USNM 293907 (2, 15.7–21.7), Baluarte Bay, Batan Island, Batanes, 0–4 m, 22 Apr. 1987; USNM 317986 (2 males, 20.8–23.7), Maybag Island, Babuyan Group, 8 Mar 1990; USNM 344068 (1, 20.6), Lawigan, San Joaquin, Iloilo, Panay Island, 0–7 m, 25 Sep. 1995.

**Remarks.** The holotype was originally mixed with 23 specimens; 21 specimens were re-cataloged as USNM 344099; two specimens were exchanged to SMNS. A photograph and radiograph of the holotype are available in the USNM record.

**(592) *Helcogrammaalbimacula* Williams & Howe, 2003: 154, figs 1, 2**

**Holotype.**USNM 273934 (male, 34.9), 09°04.50'N, 123°16.40'E, Apo Island, opposite west end of Chanos Pond, Negros Island, 0–2.5 m, 18 May 1979.

**Paratypes.** AMS I.35024-001 (2, 26.9–30.4), BPBM 36434 (2, 31.2–33.5), CAS 81761 (2, 25.4–30.9), and USNM 315775 (42, 25.5–37.8), all taken with holotype.

**Remarks.** Six specimens from USNM 315775 were exchanged to AMS, BPBM, and CAS. A photograph and radiograph of the holotype are available in the USNM record.

**(593) *Helcogrammaaquila* Williams & McCormick, 1990: 1021, fig. 3**

**Holotype.**USNM 298405 (male, 39.7), 20°24.75'N, 121°55.03'E, White Beach, past Mahatae, Batan Island, Batanes, 0–6 m, 22 Apr. 1987.

**Paratypes.** AMS 1.29427-001 (1, 38.1), ANSP 164964 [ex USNM] (1, 37.6), and BPBM 32855 (1, 36.6 mm), same data as holotype; USNM 293945 (male, 36.1); Balugan Bay, near Desquid Point, Batan Island, Batanes, 0–3 m, 23 Apr. 1987; USNM 297370 (4, 35.7–39.2 mm), ~ 0.5 km southeast of Diojo Point, Batan Island, Batanes, 0–5 m, 24 Apr. 1987.

**Remarks.** A photograph and radiograph of the holotype are available in the USNM record.

**(594) *Helcogrammadesa* Williams & Howe, 2003: 161, figs 8, 9**

**Holotype.**USNM 222317 (male, 33.6), 10°52.90'N, 121°12.23"E, Cocoro Island Cuyo, Palawan, 0–3 m, 25 May 1978.

**Paratypes.** AMS I.35025-001 (male, 32.7), BPBM 36433 (female, 29.1) and USNM 222320 [some ex USNM 222317] (18, 23.8–34.8), taken with holotype; USNM 222898 (5, 22.7–27.2), Tagauayan Island, Cuyo, Palawan, 0–2.4 m, 25 May 1978.

**Remarks.** The holotype was originally mixed with 13 specimens; one was exchanged to BPBM, 11 specimens were combined with USNM 222320. A photograph and radiograph of the holotype are available in the USNM record.

**(595) *Helcogrammafuscopinna* Holleman, 1982: 115, fig. 4**

**Holotype.** SAIAB [ex RUSI] 954. Sodwana Bay, Zululand, KwaZulu-Natal, South Africa.

**Paratypes.**USNM 227742 (3, 35.0–36.4), west of Apo Island, Negros, 0–6 m, 6 Jun. 1978; USNM 227744 (2, 31.7–39.3), Putic Island, Cuyo, Palawan, 0–4.6 m, 22 May 1978.

**Remarks.**USNM 227742 was re-identified as *Helcogrammaalbimacula* by JT Williams. USNM 227744 contains non-type specimens of *Helcogrammadesa*.

**(596) *Helcogrammahabena* Williams & McCormick, 1990: 1026, fig. 6**

**Holotype.**USNM 300194 [ex USNM 300196] (male, 34.7), 20°27.92'N, 121°57.20'E, SE of Diojo Point, Batan Island, Batanes, 3–6 m, 2 May 1987.

**Paratypes.**USNM 300196 (18, 20.9–42), same data as holotype; USNM 293946 (10, 13.6–41.1), AMS 1.29428-001 [ex USNM 293946] (2, 29.8–37.1), ANSP 164965 [ex USNM 293946] (2, 27.3–28.7) and BPBM 32856 [ex USNM 293946] (2, 28.7–32.5), Balugan Bay, near Desquid Point, Batan Island, Batanes, 0–3 m, 23 Apr. 1987; USNM 300193 (14, 11.8–36.7), Ibahos Island, Batanes, 1.5–4.5 m, 3 May 1987; USNM 300195 (16, 22.7–31.9), NW of Itbayat Island, Batanes, 0–4.5 m, 24 Apr. 1987.

**Remarks.** The original description mentioned 21 specimens for USNM 300196 and 12 specimens for USNM 300193, but it was an error based on the USNM record. USNM 293946 originally had 16 specimens, but six were sent to AMS, BPBM, and ANSP. A photograph and radiograph of the holotype are also available.

**(597) *Helcogrammarhinoceros* Hansen, 1986: 344, fig. 7 (left), 15**

**Holotype.**USNM 222370 [ex USNM 221917] (male, 27.0), 10°55.08'N, 121°02.05'E, northwest of Putic Island, Cuyo, Palawan, 0–4.6 m, 22 May 1978.

**Paratypes.**USNM 221917 (6 males, 17.0–26.0) and USNM 221921 (3 males, 17.5–29.0 + 3 females, 22.5–27.0), same data as holotype; USNM 221920 (2 males, 25.2–28.5), Cocoro Island, Cuyo, Palawan, 26 May 1978.

**Remarks.** The original description mentioned two males and four females for USNM 221917. A photograph and radiograph of the holotype are available in the USNM record.

**(598) *Helcogrammaspringeri* Hansen, 1986: 345, fig. 16**

**Holotype.**USNM 229368. Off Tandjung Suli, Ambon Island, Molucca Islands, Indonesia.

**Paratypes.**USNM 228932 (1 male 22.5 + 1 female 27), Pagnagtaran Point, Sulu Sea, Puerto Princesa, Palawan Island, 6–12 m, 2 Jul. 1979; USNM 263389 (1 male, 27.5), Cuyo Island, Palawan, 0.6–1.2 m; USNM 285799 (1 male, 25.1), Cuyo Island, Palawan, 21 May 1978.

**Remarks.** The original description listed two specimens, but only one specimen was found inside USNM 263389 when it was cataloged. A specimen was found to be out of one of the jars of paratypes, but JT Williams was unsure which paratypic lot it belonged to. The separated specimen was re-cataloged as USNM 285799 and a paratype of *H.springeri* (Springer and Orell 1996).

**(599) *Helcogrammastriata* Hansen, 1986: 349, fig. 18**

**Holotype.**USNM 221667. Miyakejima, Izu Islands, Japan.

**Paratypes.**USNM 221669 (5 males, 23.6–25.2 + 4 females, 23–25.6), Ajong, Negros Island, 0–3 m.

**Remarks.** The original description or USNM record did not indicate the collection date.

**(600) *Tripterygiumphilippinum* Peters, 1868: 269**

= *Enneapterygiusphilippinus* (Peters, 1868).

**Lectotype.** ZMB 6708 (male, 18.7), 13°07.0'N, 124°15.0'E, ~ 20 km northeast of Sorsogon, at the south entrance of Albay Gulf, Luzon, Oct. 1859.

**Paralectotype.** ZMB 32768 [ex ZMB 6708] (female, 17.1), same data as lectotype.

**Remarks.** The original genus should have been *Tripterygion*. The syntypes were missing and found again but a lectotype was already designated by Fricke (1997).

**(601) *Uclaxenogrammus* Holleman, 1993: 5, figs 5, 6**

**Holotype.** ROM 45272. Lizard Island, Great Barrier Reef, Australia.

**Paratypes.** AMS I.21903-033 (2, 27.3–29.1), Santiago Island; USNM 263647 (4, 33.7–36.0), USNM 267932 (1, 33.8) and USNM 263650 (5, 35.0–37.0), Bararin Island, Cuyo, Palawan, 0–17 m, 23–24 May 1978; USNM 230395 (1, 39.5), Caban Island, Batangas, Luzon, 24 Apr. 1980; USNM 263641 (1, 39.5), USNM 263643 (2, 25.0–27.6) and USNM 263646 (1, 38.8), Cuyo Island, Palawan, 0–1 m, 21–24 May 1978; USNM 263645 (3, 18.0–36.4), Tagauayan Island, Cuyo, Palawan, 0–14 m, 25 May 1978; USNM 267930 (2, 39.1–41.6), Cocoro Island, Cuyo, Palawan, 0–21 m, 26 May 1978; USNM 263644 (2, 34.3–43.0), San Juan, Siquijor Island, 0–6 m, 9 May 1978; USNM 267937 (1, 32.5), Balicasag Island, Bohol, 0–24 m, 10 Jun. 1978.

##### Family Blenniidae (296)

**(602) *Atrosalariashosokawai* Suzuki & Senou, 1999: 260, figs 1–4**

**Holotype.** YCM-P 34441. Amami-Oshima Island, Kagoshima, Japan.

**Paratypes.**USNM 297591 (1, 48.0), south of San Juan, Siquijor Island, 0–10.7, 10 May 1978; USNM 297595 (1, 58.0), west of Siquijor Town, Siquijor Island, 24–30 m, 14 May 1979; USNM 297594 (1, 61.1), White Beach reef, Puerto Princesa Bay, Palawan, Island, 6–12 m, 3 Jul. 1979.

**(603) *Blenniusthysanius* Jordan & Seale, 1907: 47, fig. 19**

= *Parablenniusthysanius* (Jordan & Seale, 1907).

**Syntypes.**CAS-SU 9252 (1, 71.0 TL) and USNM 53071 (1, 52.6), Cavite, Luzon, 1 Jun. 1900.

**Remarks.** The published catalog number of a syntype (USNM 53072) was an error, and it should be USNM 53071. Springer et al. (1991) provided a taxonomic remark. Photographs and radiographs of the type specimens are available in the USNM and the CAS records.

**(604) *Cirripectesauritus* Carlson, 1981: 408, figs 1, 2, 3c**

**Holotype.** BPBM 20478. English Harbor, Tabuaeran [= Fanning Island], Line Islands.

**Paratype.**USNM 222490 (1, 31.8), SP 79-20, rocks at the base of the cliff just west of Chanos pond, Apo Island, off southern Negros, 0–2.4 m, 18 May 1979.

**(605) *Cirripectesspringeri* Williams, 1988: 62, pl. 4 (B, C)**

**Holotype.** BPBM 22121, west of Sumilon Island, Cebu, 2 m, 25 Aug. 1977.

**Paratypes.** ROM 53380 (1), northwest of Sumilon Island, Cebu, 20 May 1987; USNM 228284 (2), Port Siyt, S Negros Island, 0–3 m, 28 Apr. 1979; ROM 53378 (4), ROM 53379 (2) and USNM 228287 (8), near Tonga Point, Siquijor Island, 0–1 m, 9–15 May 1979; USNM 228288 (1), west of Solino Island, Zamboanga, Mindanao, 0–5 m, 3 May 1979.

**(606) *Cirripectesviriosus* Williams, 1988: 73, fig. 21**

**Holotype.**USNM 289070 (male, 115.0), 20°26.0'N, 121°57.92'E, Near Desquid Point, Balugan Bay, Batan Island, Batanes, 0–3 m, 23 Apr. 1987.

**Paratype.**USNM 283820 (1), same locality as holotype, 6 m, 29 May 1985.

**Remarks.** A photograph and radiograph of the holotype are available in the USNM record.

**(607) *Ecseniusdilemma* Springer, 1988: 88, pl. 11 (figs 2–4); fig. 46**

**Holotype.**USNM 231319 [ex USNM 225053] (male, 31.3), Sombrero Island, Batangas, Luzon, 0–10 m, 23 Apr. 1980.

**Paratypes.**USNM 225053 (10, 15.0–30.0), taken with holotype; AMS I.21908-021 (2, 17.0–21.0), same locality with the holotype, 1–34 m; AMS I.21918-003 (2, 18.0–25.0), AMS I.21918-004 (1, 19.0), Caban Island, Batangas, Luzon, 11–29 m, 25 Apr. 1980; BPBM 26848 (1, 29.0) and BPBM 28481 (1, 28.0), same locality as AMS I.21918-003, 7–30 m, 25 May 1981; USNM 220973 (6, 14.0–23.0), Liloan Point, southern Cebu Island, 13–19 m, 29 Apr. 1979; USNM 220974 (3, 13.0–36.0), northwest of Pescador Island, Cebu, 18–24 m, 7 May 1979; AMS I.21915-045 (2, 17.0–28.0), USNM 225052 (2, 18.0–19.0), USNM 228919 (1, 16.0) and USNM 228919 (1, 16.0), Sombrero Island, Batangas, Luzon, 0–10 m, 23–24 Apr. 1980.

**Remarks.** A photograph and radiograph of the holotype are available in the USNM record.

**(608) *Ecseniuskurti* Springer, 1988: 117, fig. 63**

**Holotype.**USNM 227416 (male, 35.0), Bararin Island, Cuyo, Palawan, 0–14 m, 23 May 1978.

**Paratypes.**USNM 219305 (7, 12.0–32.0), taken with holotype; USNM 219303 (2, 30.0–33.0), Tagauayan Island, 0–2 m, 25 May 1978; USNM 219311 (3, 26.0–30.0), Bararin Island, 0–17 m, 24 May 1978; all taken from Cuyo, Palawan.

**Remarks.** A photograph and radiograph of the holotype are available in the USNM record.

**(609) *Ecseniusmonoculus* Springer, 1988: 57, pl. 7 (fig. 4), fig. 32d**

**Holotype.**USNM 261243 [ex USNM 219309] (male, 36.0), northwest Putic Island, Cuyo, Palawan, 22 May 1978.

**Paratypes.**USNM 219309 (18, 16.0–42.0) and USNM 226575 (6, 13.0–38.0), taken with holotype; USNM 219308 (1, 43.0), Tagauayan Island, Cuyo, Palawan, 0–2.4 m, 25 May 1978; USNM 225050 (7, 16.0–33.0), Bolinao, Pangasinan, Luzon, 0–2 m, 17 Apr. 1980; USNM 226577 (5, 15.0–30.0), Maloh, Negros Island, 0–3.1 m, 18 May 1979; USNM 226579 (31, 22.0–38.0), near Maloh, Negros Island, 24 Apr. 1979; USNM 226581 (7, 23.0–49.0), Cocoro Island, Cuyo, Palawan, 0–3 m, 26 May 1978; USNM 227537 (1, 24.0) and USNM 226578 (15, 14.0–35.0), near Giligaon, north of Maloh, Negros Island, 0–1.8 m, 26 Apr. 1979; USNM 226580 (1, 28.0), Sombrero Island, Batangas, Luzon, 30–31 Jan. 1980; AMS 1.21909-001 (2, 43.0), USNM 228928 (1, 30.0), USNM 228926 (2, 27.0–41.0) and USNM 225049 (5, 27.0–43.0), Sombrero Island, Batangas, Luzon, 0–5 m, 23–25 Apr. 1980.

**Remarks.** Photographs and a radiograph of the holotype are available in the USNM record.

**(610) *Ecseniusstigmatura* Fowler in Chapman & Schultz, 1952: 514, fig. 90**

**Holotype.**USNM 99379 (female, 46.5), Albatross station 5566 (05°52.20'N, 120°31.00'E), Dammi Island, between Jolo and Tawi Tawi islands, Sulu Archipelago, 446 m, 21 Sep. 1909.

**Paratypes.**USNM 111878 [ex USNM 99379] (1 female, 37.0) same data as holotype; USNM 122444 (female, 36.2) Cataingan Bay, east of Masbate Island, 18 Apr. 1908.

**Remarks.**Springer et al. (1991) remarked that the published type locality is erroneous, and the correct locality of the holotype and one paratype (USNM 111878) is probably Tomahu Island, off the west of Bouro Island, Indonesia. They also noted that Schultz prepared the species description but could not remember how the locality was decided. The authorship was attributed to Fowler as he recognized the specimens as undescribed species and many jars of Albatross specimens at USNM contain Fowler’s handwritten labels with manuscript names. A photograph and radiograph of the holotype are available in the USNM record.

**(611) *Ecseniustricolor* Springer & Allen, 2001: 158, figs 9, 10**

**Holotype.**USNM 348659 (female, 41.7), 11°40.67'N, 119°56.60'E, southwest tip of Culion Island, Palawan, 12–15 m, 19 Feb. 1998.

**Paratypes.** AMS I.38809-001 (2) and CAS 200240 (2), same data as holotype; USNM 222137 (7), north of Rita Island, Ulugan Bay, Puerto Princesa, Palawan Island, 9–14 m, 6 Aug. 1979.

**Remarks.** Four of five original type specimens were mixed with holotype and re-cataloged as paratypes (AMS I.38809-001 and CAS 200240). USNM 222137 was originally designated as paratypes of *Ecseniusmelarchus* McKinney & Springer, 1976 (Smithsonian Museum Archive 2024).

**(612) *Hypleurochilusloxias* Jordan & Seale, 1905: 802, fig. 20**

**Holotype.**USNM 51952 (28.5), Negros Island, 1901.

**Remarks.** A printed tag reading “type” was tied to the holotype but is now removed and loose in a jar. The holotype has a marking of a string used to tie on a tag. A photograph and radiograph of the holotype are available in the USNM record (Smithsonian Museum Archive 2024).

**(613) *Meiacanthusabditus* Smith-Vaniz, 1987: 15, figs 7b, 8b**

**Holotype.**USNM 276511 [ex USNM 122384] (female, 29.3), Albatross station 5561 (05°50.75'N, 120°01.25'E), Teomabal Island, Sulu Archipelago, 0 m, 19 Sep. 1909.

**Paratypes.**USNM 122384 (28, 10.2–14.1), same data as holotype; USNM 99370 (1, 48.3), Albatross station 5148, Sirun Island, Siasi, Sulu Archipelago, 31 m, 16 Feb. 1908.

**Remarks.** The original description mentioned Tutu Bay in Jolo Island as the holotype locality with 3–6 m but did not concur with the USNM record. USNM 122384 originally contained 29 specimens. A photograph and radiograph of the holotype are available in the USNM record.

**(614) *Petroscirteseretes* Jordan & Seale, 1905: 801, fig. 19**

= *Petroscirtesvariabilis* Cantor, 1849.

**Holotype.**USNM 51949 (77.0), south of Negros Island, 1900–1901.

**Paratypes.**MCZ 35987 [ex USNM 51949] (1, 38.0), CAS-SU 9132 (2) and USNM 210617 [ex USNM 51949] (2), same locality with holotype.

**Remarks.** The type specimens were originally mixed but were separated and re-cataloged into different paratypes. Six of the type specimens originally described were specimens of *Petroscirtesvariabilis* (Smithsonian Museum Archive 2024). A photograph and radiograph of the holotype are available in the USNM record.

**(615) *Petroscirtesfeliciana* Herre, 1942b: 112**

= *Omobranchusferox* (Herre, 1927).

**Holotype.**CAS-SU 36671 (55.0), mangrove swamp beside the Fisheries Station at Cagayan, Misamis, Mindanao, 20 Aug. 1940.

**Paratype.**CAS-SU 36672 (1, 45.0), same data as holotype.

**Remarks.** A photograph and a radiograph of the holotype are available in the CAS record.

**(616) *Petroscirtesferox* Herre, 1927b: 277, pl. 3 (figs 2, 3)**

= *Omobranchusferox* (Herre, 1927).

**Neotype.**CAS-SU 67264 (male, 38.1), Lake Bombon (Taal Lake), Batangas, Luzon, May 1931.

**Remarks.** The original syntypes (BSMP 60, type localities: Ambulong, Talisay, and around Volcano Island, Taal Lake) were presumed destroyed and a neotype was designated by Springer and Gomon (1975) from one of the 19 specimens measuring 14.5–41.7. This was originally cataloged as CAS-SU 28446. A photograph and a radiograph of the holotype are available in the CAS record.

**(617) *Petroscirtesvulsus* Jordan & Seale, 1907: 47, fig. 20**

= *Petroscirtesvariabilis* Cantor, 1849.

**Holotype.**CAS-SU 9253 (56.4), Manila, Luzon.

**Remarks.** The original description or CAS record did not indicate the collection date. A photograph and a radiograph of the holotype are available in the CAS record.

**(618) *Petroscirteswaterousi* Herre, 1942b: 112**

= *Omobranchusferox* (Herre, 1927).

**Holotype.**CAS-SU 36673 (33.0), swamp at Waterous Hacienda, Mangarin, Mindoro Island, 20–22 Jul. 1940.

**Remarks.** A photograph and a radiograph of the holotype are available in the CAS record.

**(619) Plagiotremus (Plagiotremus) iosodon Smith-Vaniz, 1976: 128, fig. 162**

**Holotype.**USNM 99392 (male, 38.0), Albatross station 5561, Teomabal Island, Jolo Island, Sulu Archipelago, 0 m, 18 Sep. 1909.

**Paratype.**USNM 205372 [ex USNM 99392] (1, 40.0), taken with holotype.

**Remarks.** The type specimens constitute syntypes of the unpublished manuscript of Fowler syntypes and was labeled as *Taeniaspidontusbrachyrhinus* (Smithsonian Museum Archive 2024). A photograph and radiograph of the holotype are available in the USNM record.

**(620) *Praealticusstriatus* Bath, 1992: 280, figs 60–71, 87e–l**

**Holotype.**USNM 317941 [ex USNM 296249], Cocoro Island, Cuyo, Palawan, 0–3 m, 26 May 1978.

**Paratypes.** BPBM 35000 (2), SMF 18067 (2), CAS 76305 (2) and USNM 296249 (99), same data as the holotype.

**Remarks.** All type specimens were removed from USNM 296249 (originally with 106 specimens). A photograph and radiograph of the holotype are available in the USNM record (Smithsonian Museum Archive 2024).

**(621) *Salariasbilineatus* Peters, 1868: 269**

= *Praealticusbilineatus* (Peters, 1868).

**Holotype.** ZMB 6707, a coral reef, east of Lauang, Samar Island.

**Remarks.** The original description did not indicate the collection date.

**(622) *Salariascolei* Herre, 1934: 96**

= *Istiblenniuscolei* (Herre, 1934).

**Holotype.**CAS-SU 25520, reef in Culion Harbor, Palawan, 2 May 1931.

**Paratype.**CAS-SU 25521 (1), same data as holotype.

**Remarks.** A photograph and a radiograph of the holotype are available in the CAS record.

**(623) *Salariasdeani* Jordan & Seale, 1905: 799, fig. 17**

= *Blenniellabilitonensis* (Bleeker, 1858).

**Holotype.**USNM 51950 (53.3 TL), south of Negros Island, 1901.

**Remarks.** A photograph and radiographs of the holotype are available in the USNM record.

**(624) *Salariasfowleri* Herre, 1936b: 364, pl. 2 (fig. 7)**

= *Litobranchusfowleri* (Herre, 1936).

**Holotype.**CAS-SU 30971 (31.0), tide pool near Dumaguete, Negros Island, 23 Nov. 1933.

**Paratypes.**CAS-SU 69720 [ex CAS-SU 30971] (6), USNM 203763 [ex CAS-SU 30971] (1) (16.0–28.0), same data as holotype.

**Remarks.** The original description did not provide catalog numbers. All type specimens were mixed in one bottle (CAS-SU 30971), but Böhlke (1953) reported the holotype and seven paratypes. A photograph and a radiograph of the holotype are available in CAS record.

**(625) *Salariasholomelas* Günther, 1872: 399**

= *Atrosalariasholomelas* (Günther, 1872).

**Holotype.**BMNH 1872.10.18.108 (76.2), Cebu Island.

**Other catalog number.** NHMUK:ecatalogue:3104681.

**Remarks.** The original description or BMNH record did not indicate the collection date.

**(626) *Salariasmartini* Herre, 1942d: 2**

= *Istiblenniuscolei* (Herre, 1934).

**Holotype.**CAS-SU 38253 (male, 119.0), reef at Estancia, Iloilo, Panay Island, 27 Jul. 1940.

**Paratypes.** AMS IB.3444 (6), FMNH 40727–33 (7), USNM 323836 [ex CAS-SU 38249] (2), CAS-SU 38249 (100), CAS-SU 38254 (10) and USNM 123655 (3 males, 3 females), same locality with the holotype.

**Remarks.** The original description mentioned 227 type specimens but provided no catalog numbers, all of which were deposited in SU Böhlke (1953) identified SU 38253 as the holotype and 10 paratypes (SU 38254). Springer et al. (1991) examined 53 paratypes (CAS-SU 38249); six paratypes (AMS IB.3444) are at the Australian Museum. A photograph and a radiograph of the holotype are available in the CAS record.

**(627) *Salariasmontanoi* Sauvage, 1880: 219 [9**]

= *Alticusmontanoi* (Sauvage, 1880).

**Holotype.**MNHN A-2199 (7.0), 13°00.00'N, 123°49.98'E, Albay, Luzon.

**Remarks.** The original description or MNHN record did not indicate the collection date.

**(628) *Salariasobscurus* Bath, 1992: 225, figs 1–6, 11a–g**

**Holotype.**USNM 218705, 10°51.33'N, 121°00.23'E, Cuyo Island, Palawan, 1 m, 21 May 1978.

**Paratypes.** BPBM 34522 (1), CAS 74736 (1), SMF 18063 (1), USNM 279624 (2) and USNM 315774 [ex USNM 218705] (21), taken with the holotype.

**Remarks.** The holotype was originally mixed with 21 paratypes, but paratypes were moved to USNM 315774. A photograph and radiograph of the holotype are available in the USNM record (Smithsonian Museum Archive 2024).

**(629) *Salariasperiopthalmusvisayanus* Herre, 1934: 97**

= *Blenniellabilitonensis* (Bleeker, 1858).

**Neotype.**CAS-SU 28435 (49.8, male), Dumaguete, Negros Island, 27 Jun. 1931.

**Paralectotypes.**CAS-SU 28436 (10), Culion Island, Palawan, 2 May 1931; BMNH 1933.3.11, 713–715 (3), same locality as holotype; CAS-SU 69690 (6).

**Other catalog number.**BMNH 1933.3.11.713–715 (NHMUK:ecatalogue:2511800).

**Remarks.** The type specimen was not separable from paratypes and a neotype was designated by Springer and Williams (1994). A photograph and a radiograph of the holotype are available in the CAS record.

**(630) *Salariasreyi* Sauvage, 1880: 219 [9**]

= *Andamiareyi* (Sauvage, 1880).

**Holotype.**MNHN A-2201 (7.0), Luzon Island.

**Remarks.** The original description or MNHN record did not indicate the collection date.

**(631) *Salariasundecimalis* Jordan & Seale, 1905: 800, fig. 18**

= *Salariasguttatus* Valenciennes, 1836.

**Holotype.**USNM 51942 (41.6), southern Negros, 1901.

**Paratypes.**CAS-SU 9133 (2) and USNM 138302 [ex part of USNM 51942] (1), same locality with the holotype.

**Remarks.** A photograph and radiograph of the holotype are available in the USNM record.

**(632) *Salariaszamboangae* Evermann & Seale, 1906: 512, fig. 4**

= *Istiblenniusdussumieri* (Valenciennes, 1836).

**Holotype.**USNM 55623 (72.2), Caldera Bay, Zamboanga, Mindanao, 1904.

**Paratypes.**USNM 291687 [ex USNM 55623] (1) and CAS-SU 10007 [ex USNM 291687] (1), same data as the holotype.

**Remarks.** According to Springer et al. (1991), the original description mentioned that the paratypes were cataloged at different institutions, but all type specimens were cataloged as USNM 55623. The holotype was separated and assigned to the original catalog number, while the two specimens were designated as paratypes and re-cataloged as USNM 291687. One of these specimens was meant to be designated to Stanford University as SU 10007. However, Springer et al. (1991) noted that Böhlke (1953) made an error in noting that one paratype was cataloged as SU 10007. Similarly, the SU 10007 was indicated as a co-type in the original description, but the SU ledger indicated this number is assigned to a different species (*Sebastodespinniger*). A photograph and radiograph of the holotype are available in the USNM record.

#### ﻿﻿ORDER GOBIESOCIFORMES

##### Family Gobiesocidae (300)

**(633) *Flabellicaudaalleni* Fujiwara, Conway & Motomura, 2021: 757, figs 1A, 2A, 3, 4A–E, 5, 6A–C, 7A, 8A–D, 10–13**

**Holotype.**USNM 360264 (26.2), 10°51'12"N, 121°00'14"E, west of Cuyo Island, Palawan, 0.6–1.2 m, 21 May 1978.

**Paratypes.** KAUM-I. 146168 (1, 20.9), KAUM-I. 146169 (1, 18.2), KAUM-I. 146170 (1, 17.1) and USNM 451066 [ex USNM 360264] (5, 15.0–23.1), same data as holotype; FMNH 127786 (19, 12.5–26.5), north of Cabilauan Island, Coron, Palawan, 5 Mar. 2003; ROM 55178 (1, 11.6), ROM 55181 (1, 11.1) and ROM 55183 (1, 19.2), Pasihagon, Siquijor Island, 10.4–18.8 m, 10–14 May 1987; ROM 55185 (4, 11.5–23.8), mouth of north Bais Bay, Negros Island, 23.1 m, 17 May 1987; USNM 360265 (2, 12.4–13.3), San Juan, Siquijor Island, 0–6.1 m, 9 May 1978.

**Remarks.** The holotype was originally mixed with some paratypes and re-cataloged as USNM 451066; three specimens were donated to KAUM.

**(634) *Lepadichthysspringeri* Briggs, 2001: 499, fig. 1**

= *Lepadichthysmisakius* (Tanaka, 1908).

**Holotype.**USNM 359726 (26.1), 13°42.00'N, 120°49.00'E, northeast corner of Sombrero Island, Batangas, Luzon, 0–10 m, 23 Apr. 1980.

**Paratype.**USNM 359727 (1, 18.9), Tangauayan Island, Cuyo, Palawan, 0–2 m, 25 May 1978.

**(635) *Propheralloduslongipterus* Fujiwara & Motomura, 2018: [9], figs 4c, d, 5b, 6d–f, 7**

**Holotype.**ANSP 163524 [ex USNM 298202] (17.0), 20°48.00'N, 121°50.00'E off Itbayat Island, west coast of Batanes, 0–4.6 m, 24 Apr. 1987.

**Paratypes.** KAUM-I. 114615 (1, 17.8) and USNM 298202 (1, 22.8), same data as holotype.

#### ﻿﻿ORDER ATHERINIFORMES (63)

##### Family Atherionidae (305)

**(636) *Atherionelymusaphrozoicus* Schultz in Schultz et al., 1953: 295**

= *Atherionelymus* Jordan & Starks, 1901.

**Holotype.**USNM 143305 [ex USNM 143303] (31.0), Tara Island, Coron, Palawan, 15 Dec. 1908.

**Paratypes.** FMNH 62599 [ex USNM 140303] (1) and USNM 143303 [ex USNM 136803] (13, 25.5–33.0), taken with holotype.

**Remarks.** One specimen of USNM 143303 was exchanged to CNHM (FMNH?). USNM 136803 specimens are paratypes of *Allanettacrenolepis*.

##### Family Dentatherinidae (306)

**(637) *Dentatherinamerceri* Patten & Ivantsoff, 1983: 331, fig. 1**

**Holotype.**USNM 210180. Tanjung Liang, Piru Bay, Molucca Islands, Indonesia.

**Paratype.**USNM 220142 (2, 19.7–21.4), SP 78-44, northwest side of the entrance to Port Siyt, Negros Island, 0–2 m, 14 Jun. 1978; USNM 230370 (4, 23.6–26.0), SP 78-18, northwest Putic Island, Cuyo, Palawan, 0–4.6 m, 22 May 1978.

**Remarks.** Based on the USNM records indicated that 220142 originally contained seven specimens, but five were re-cataloged as USNM 230377; USNM 230370 originally contained 51 specimens, but 47 were re-cataloged as230371 and 230181. All specimens re-cataloged were non-types.

##### Family Phallostethidae (307)

**(638) *Gulaphallusamaricola* Villadolid & Manacop, 1935: 194, pl. 1 (figs 1, 2)**

= *Neostethusamaricola* (Villadolid & Manacop, 1935).

**Holotype.** College Agric. Univ. Philippines, Tidal slough at Pasay, Rizal, Luzon.

**Paratype.** College Agric. Univ. Philippines (126).

**Remarks.** The original description did not indicate the collection date. All type specimens were lost (Kottelat 2013).

**(639) *Gulaphalluseximius* Herre, 1925b: 509, pls 1, 2, figs 1, 2**

**Lectotype.**CAS-SU 24474 (male, 33.5), Creek at Santa Fe, Nueva Vizcaya, Luzon, 18 May 1924.

**Paralectotypes.**CAS-SU 18149 (3), same data as holotype.

**Remarks.** The original description did not designate a type specimen. Most of the original type specimens deposited in the Philippine Bureau of Science were destroyed during World War II, and a lectotype was designated by Böhlke (1953).

**(640) *Gulaphallusfalcifer* Manacop, 1936: 375, pl. 1 (figs 1, 1a, 2, 2a)**

**Holotype.** BSMP 31778 (male, 27.5), Barrio Laput, Mexico, Pampanga, Luzon, 27 Aug. 1935.

**Paratypes.** BSMP [ex FGA] 31779 (1, 26.5) and BSMP [ex FGA] 31780 (9 males + 8 males, 20.0–34.0), same data as holotype.

**Remarks.** All type specimens were lost (Kottelat 2013).

**(641) *Gulaphallusmirabilis* Herre, 1925b: 511, pl. 2 (figs 3–5)**

**Syntypes.**USNM 104412 [ex UW 849] (2) and BSMP, Molawin Creek, Los Baños, Laguna, Luzon.

**Remarks.** The original description examined 25 males measuring 23.0–33.0 and 23 females measuring 25.0–32.0 from the Ibo River mouth, flowing into Angat River, Bulacan, but did not mention the collection dates and if these are type specimens. USNM 104412 was labeled as “paratype” in the USNM record. All BSMP specimens were lost (Kottelat 2013).

**(642) *Mirophallusbikolanus* Herre, 1926b: 540, pl. 3 (figs 1–6)**

= *Gulaphallusbikolanus* (Herre, 1926).

**Lectotype.**CAS-SU 24475 (male, 19.5), Lake Bato, Camarines Sur, Luzon, 30 Jan. 1926.

**Paralectotypes.**CAS-SU 18148 (2) and MNHN 1927-0194 (2, 21.1–24.1), same data as the holotype.

**Remarks.** The original description did not designate a type specimen and most of the specimens deposited in the Philippine Bureau of Science were destroyed during World War II. A lectotype was designated by Böhlke (1953). While Böhlke (1953) designated only the CAS specimens as paralectotypes, some of the MNHN specimens were also identified as paralectotypes based on MNHN records. A photograph and a radiograph of the lectotype are available in the CAS record.

**(643) Neostethus (Sandakanus) coronensis Herre, 1942c: 152**

= *Neostethusborneensis* Herre, 1939.

**Holotype.**CAS-SU 36542, mangrove swamp near the dock at Coron Island, Palawan, 28 Jun. 1940.

**Paratypes.**CAS-SU 36543 (109), same data as holotype.

**Remarks.** A photograph and a radiograph of the holotype are available in the CAS record.

**(644) Neostethus (Sandakanus) panayensis Herre, 1942c: 153**

= *Gulaphalluspanayensis* (Herre, 1942).

**Holotype.**CAS-SU 36539 (male, 19.0), near Capiz, Panay Island, 3 Aug. 1940.

**Paratypes.**CAS-SU 36940 (4 males, 15.0–18.5 + 16 females, 18.0–24.0), same data as holotype; CAS-SU 36541 (6 males, 17.0–19.0 + 17 females, 21.0–24.0), Nipa palms at Estancia, Iloilo, Panay Island, 27 Jul. 1940.

**Remarks.** A photograph and a radiograph of the holotype are available in the CAS record.

**(645) Neostethus (Sandakanus) zamboangae Herre, 1942c: 153**

**Holotype.**CAS-SU 36544, saltwater puddles near fishery station at Zamboanga, Mindanao, 6 Sep. 1940.

**Paratypes.**CAS-SU 36545 (240), same data as holotype.

**Remarks.** A photograph and a radiograph of the holotype are available in the CAS record.

**(646) *Neostethusrobertsi* Parenti, 1989: 272, figs 12–13**

**Holotype.**CAS 50723 (male, 22.3), tidal flat, ~ 12 km north of San Carlos, Pangasinan, Luzon, 16 Mar 1976.

**Paratypes.**CAS 64254 (40, 9.0–23.0), taken with holotype.

**Remarks.** A photograph and a radiograph of the holotype are available in the CAS record.

**(647) *Neostethusvilladolidi* Herre, 1942c: 150**

**Holotype.**CAS-SU 36537, mangrove swamp beside Fishery Experiment Station at Cagayan de Misamis, Mindanao, 20 Aug. 1940.

**Paratypes.**CAS-SU 36538 (58), same data as holotype.

**Remarks.** A photograph and a radiograph of the holotype are available in the CAS record.

**(648) *Plectrostethuspalawanensis* Myers, 1935: 5**

= *Neostethuspalawanensis* (Myers, 1935).

**Holotype.**USNM 93421 (male, 23.0), mouth of Caiholo River, Ulugan Bay, Puerto Princesa, Palawan Island, 29 Dec. 1908.

**Paratypes.**USNM 93422 (1 female, 19.5) and USNM 93423 (9), same data as holotype.

**Remarks.**USNM 93422 is an allotype (=paratype). USNM 93423 originally contained 10 specimens; one was cleared and stained.

**(649) *Solenophallusctenophorus* Aurich, 1937: 272, figs 1b, 6, 7(II–V)**

= *Neostethusctenophorus* (Aurich, 1937).

**Syntypes.** Uncat. (male, 24.0) and (female, 20.0), from a tributary of Laguna de Bay, Luzon.

**Remarks.** The original description did not provide catalog numbers and collection date. The type specimens were lost (Kottelat 2013).

**(650) *Solenophallusthessa* Aurich, 1937: 264, figs 1a, 3–5, 7I**

= *Neostethusthessa* (Aurich, 1937).

**Syntypes.** Uncat. (male, 26.0) and (female, 29.0), Lake Mainit, Surigao, Mindanao, 165 m elevation.

**Remarks.** The original description did not provide catalog numbers and collection date. The type specimens were lost (Kottelat 2013).

##### Family Atherinidae (308)

**(651) *Allanettacrenolepis* Schultz in Schultz et al., 1953: 302, fig. 46**

= *Atherinomoruscrenolepis* (Schultz, 1953).

**Holotype.**USNM 143304 [ex USNM 136803] (50.5), Tara Island, Coron, Palawan, 15 Dec. 1908.

**Paratypes.** FMNH 62598 [ex USNM 136803] (3) and USNM 136803 (62), (34.0–57.8), taken with the holotype; USNM 188065 (11, 33.5–54.5), Papatog Island, Tawi Tawi, Sulu Archipelago, 23 Feb. 1908.

**Remarks.**USNM 136803 originally had 142 mixed specimens; three were exchanged to CNHM (FMNH?), 27 (*Allanetta*) were removed to USNM 143585, one (*Pranesuspinguis*) to USNM 143302, 14 (*Atherionelymusaphrozoicus*) to USNM 143303, one to USNM 143304 (holotype), one (*A.elymusaphrozoicus*) to USNM 143305, 26 (*Pranesus*) to USNM 147097, and two (*Atherinomorusaetholepis*) to USMNM 361938.

**(652) *Atherinabalabacensis* Seale, 1910: 498, pl. 3 (fig. 2)**

= *Doboatherinabalabacensis* (Seale, 1910).

**Neotype.**USNM 136802 (64.0), San Miguel Harbor, Ticao Island, Masbate, 21 Apr. 1908.

**Remarks.** The original type specimens were lost (Kottelat 2013), and a neotype was designated by Sasaki and Kimura (2019). Originally mixed with other specimens but six were re-cataloged as USNM 136802.

**(653) *Atherinalineata* Günther, 1872: 398**

= *Atherinomorusendrachtensis* (Quoy & Gaimard, 1825).

**Lectotype.**BMNH 1872.10.18.58 (71.0), Cebu Island.

**Paralectotypes.**BMNH 1872.10.18.59 (1, 72.0), same locality as lectolotype.

**Other catalog number.**BMNH 1872.10.18.58 (NHMUK:ecatalogue:3104767); BMNH 1872.10.18.58 (NHMUK:ecatalogue:3104768).

**Remarks.** The collection date was not indicated in the original description. Kimura et al. (2001) noted that Günther (1872) based his description on two specimens from Cebu which are identical to *Atherinomorusendrachtensis*. A lectotype was designated by Kimura et al. (2001) with two paralectotypes regarded as a different species.

**(654) *Atherinapanatela* Jordan & Richardson, 1908: 243, fig. 6**

= *Hypoatherinapanatela* (Jordan & Richardson, 1908).

**Holotype.**CAS-SU 20203 (101.6), Calayan Island, Cagayan, Luzon.

**Remarks.** The collection date was not indicated in the original description.

**(655) *Atherinaregina* Seale, 1910: 496, pl. 3 (fig. 1)**

= *Atherinomorusregina* (Seale, 1910).

**Holotype.** BSMP 2082 (80.0), Culion Island, Palawan, 7 Oct. 1907.

**Paratypes.** BSMP 2083 (2), Busuanga Island, Palawan.

**Remarks.** All type specimens were presumed destroyed.

**(656) *Atherinomorusaetholepis* Kimura, Iwatsuki & Yoshino, 2002: 240, fig. 1**

= *Doboatherinaaetholepis* (Kimura, Iwatsuki & Yoshino, 2002).

**Holotype.**MNHN 2001-1247. Baguala Bay, Ambon Island, Molucca Islands, Indonesia.

**Paratypes.** FRLM 11805, 11807–09 (4, 55.0–58.0), El Nido, Palawan Island, 13 May 1992; FRLM 12101 (1, 46.0), Calawit Island, Busuanga, Palawan, 18 May 1992; USNM 361938 [ex USNM 136803] (2, 46.0–48.0), Tara Island, Coron, Palawan, 15 Dec. 1908.

**Remarks.**USNM 361938 specimens were originally paratypes of *Allanettacrenolepis* Schultz, 1953.

#### ﻿﻿ORDER BELONIFORMES (64)

##### Family Adrianichthyidae (309)

**(657) *Aplocheilusluzonensis* Herre & Ablan, 1934: 275, pl. 1**

= *Oryziasluzonensis* (Herre & Ablan, 1934).

**Holotype.** Philippines Fish and Game Administration Collection 41062 (33.0), creek and rice fields at Solsona, Ilocos Norte, Luzon, Oct. 1933.

**Paratypes.** FMNH 47042 (4) and CAS-SU 29079 (112, 11.8–39.0), same locality as holotype, 4 Nov. 1933.

**Remarks.** Many specimens (500+) measuring 9.0–30.0 were mentioned in the original description. Böhlke (1953) mentioned that CAS-SU 29079 specimens were taken on 4 Nov. 1933, but Parenti (2008) mentioned different collection dates (3–4 Oct. 1933). The holotype was considered lost (Kottelat 2013).

##### Family Exocoetidae (310)

**(658) *Cypseluruscaudimaculatus* Fowler, 1934: 328, fig. 82**

= *Cypselurusopisthopus* (Bleeker, 1865).

**Holotype.**USNM 93070 (62.0 TL), Albatross station 5561, Teomabal Island, Jolo, Sulu Archipelago, 0 m, 19 Sep. 1909.

**Paratypes.**USNM 93071 (7), taken with holotype.

##### Family Hemiramphidae (311)

**(659) *Oxyporhamphusbrevis* Seale, 1910: 495, pl. 2**

= *Melapedalionbreve* (Seale, 1910).

**Holotype.** BSMP 5301 (145.0), Paawacan, Palawan Island, 14 Aug. 1908.

**Paratypes.** BSMP (13), same data as holotype.

**Remarks.** The type locality could be “Panacan” in Narra, Palawan. All type specimens were lost (Kottelat 2013).

##### Family Zenarchopteridae (312)

**(660) Dermogenys (Rhamphodermogenys) bakeri Fowler & Bean, 1922: 15, fig. 3**

= *Nomorhamphusbakeri* (Fowler & Bean, 1922).

**Holotype.**USNM 84275 (32.6), Zamboanga, Mindanao, 27 Feb. 1914.

**(661) *Dermogenyspalawanensis* Meisner, 2001: 237, figs 35–36**

**Holotype.** ZRC 46170 (male, 33.2), Estrella Falls, Narra, Palawan Island, 29 Sep. 1994.

**Paratypes.** CMK 11972 (11 males, 23.2–36.3 + 10 females, 23.3–48.6 + 2 undet.) and ZRC 46171 (female, 44.0), taken with holotype; USNM 138672 (1 male, 31.4 + 7 females, 37.1–48.6), stream near village at Chase Head, Endeavor Stream, Taytay, 22 Dec. 1908; USNM 138673 (9 males, 25.6–32.1 + 8 females, 25.7–44.9), USNM 150809 (male, 30.9), river at Nakoda Bay, Quezon, 31 Dec. 1908; USNM 138668 (male, 29.2), Ulugan Bay, Puerto Princesa, 1945; all taken from Palawan Island.

**(662) *Dermogenyspectoralis* Fowler, 1934: 326, fig. 80**

= *Nomorhamphuspectoralis* (Fowler, 1934).

**Holotype.**USNM 93068 (female, 33.7), Bubbucan, Luzon, 17 Dec. 1907.

**Paratypes.**USNM 93069 (3, 15.6–31.1), taken with holotype.

**Remarks.** The original description mentioned the catalog number of the holotype as USNM 93058, but the USNM record showed it is USNM 93068 (Smithsonian Museum Archive 2024).

**(663) *Dermogenysphilippinus* Ladiges, 1972: 210, pl. 10**

= *Nomorhamphusphilippinus* (Ladiges, 1972).

**Holotype.** ZMH H4534 (male), Kulaman Plateau, Cebu Island.

**Paratypes.** ZMH H4535 (3), Philippines.

**Remarks.** ZMH’s record indicated different catalog numbers for holotype (135226) and paratypes (135227). Brembach (1991) noted two different spellings (*philippinius* and *philippinus*) and selected the spelling of *philippinus*. The original description did not indicate the collection dates and no specific location was provided for paratypes.

**(664) *Dermogenysrobertsi* Meisner, 2001: 236, figs 34, 35**

**Holotype.**CAS 137633 (male, 26.0), Wayan River at Bario San Nicolas, Busuanga Island, Palawan, 21 Jun. 1940.

**Paratypes.**CAS 169838 (female, 33.1) and CAS 169839 (1 male, 28.7 + 18 females, 27.5–47.6), taken with holotype; CAS 137635 (3 males, 29.3–33.4 + 8 females, 37.6–56.6), 28 Aug. 1940 and CAS 55259 (5 males, 20.8–25.5 + 5 females, 22.5–28.2 + 4 undet.); same locality as holotype, 29 Jun. 1984; CAS 128481 (1 male, 31.1 + 13 females, 31.5–50.7), Baldat, Culion Island, 28 Apr, 1931; FMNH 50935 (2 males, 23.1–31.0), San Pedro, Culion Island, 30 Mar. 1947; all were taken from Palawan.

**Remarks.**CAS specimens were not found in the CAS records.

**(665) Dermogenysviviparusvar.mindanensis Herre, 1944a: 86 [48**]

= *Nomorhamphusviviparus* (Peters, 1865).

**Syntypes.**CAS-SU 37631 (74), outlet of Lake Mainit, Jabonga, Agusan, Mindanao, 1944.

**Remarks.** A photograph and a radiograph of one of the syntypes are available in the CAS record.

**(666) *Hemirhamphuscotnog* Smith, 1902a: 170**

= *Zenarchopteruscotnog* (Smith, 1902).

**Holotype.**USNM 50537 (75.3), Lake Buhi, Camarines Sur, Luzon, 5 Jul. 1901.

**Remarks.** The original genus should have been spelled as *Hemiramphus*.

**(667) Hemirhamphus (Zenarchopterus) philippinus Peters, 1868: 273**

= *Zenarchopterusphilippinus* (Peters, 1868).

**Syntypes.** ZMB 5110 (4), Luzon Island; ZMB 5111 (2), Calbigan, Samar Island; ZMB 6719 (3), Quinoa River, near Calumpit, Bulacan, Luzon.

**Remarks.** The collection date was not indicated in the original description.

**(668) *Hemirhamphusviviparus* Peters, 1865a: 132**

= *Nomorhamphusviviparus* (Peters, 1865).

**Syntype.** ZMB 6267 (1), Basey River, Samar Island.

**Remarks.** The collection date was not indicated in the original description. The original genus should have been spelled *Hemiramphus*.

**(669) *Nomorhamphusmanifesta* Meisner, 2001: 270, figs 64, 65**

= *Nomorhamphusmanifestus* Meisner, 2001.

**Holotype.**CAS-SU 129706 (male, 37.2), Lopez, Solsona, Ilocos, Luzon, 12 Apr. 1934.

**Paratypes.**CAS-SU 169840 (female, 66.3) and CAS-SU 169841 (196 males, 32.9–38.2 + 1 female, 42.8–66.7), taken with holotype.

**Remarks.** The type specimens were not found in the CAS records.

**(670) *Nomorhamphuspinnimaculata* Meisner, 2001: 271, figs 66, 67**

= *Nomorhamphuspinnimaculatus* Meisner, 2001.

**Holotype.** ZRC 46173 (male, 41.0), creek at the east end of Tunga, Leyte Island, 7 Jul. 1993.

**Paratypes.** ZRC 46174 (female, 39.7) and CMK 9980 (37 males, 29.2–40.5 + 22 females, 24.6–50.0 + 30 undet.), taken with holotype; CMK 9984 (20 males, 28.8–41.0 + 9 females, 27.3–47.2 + 6 undet.), Hillosig Creek, 1.3 km north of Mahaplag junction on a road from Baybay to Tacloban, Leyte Island, 9 Jul. 1993.

**(671) *Nomorhamphusrossi* Meisner, 2001: 272, figs 68, 69**

**Holotype.**USNM 333262 (male, 40.3) Intel River, Barrovia Barangay hot springs, Baggao, Cagayan, Luzon, 5 May 1989.

**Paratypes.**USNM 363186 [ex USNM 333262] (female, 100.1); USNM 363187 [ex USNM 333262] (47 males, 39.3–46.6 + 72 females, 62.9–86.2 + 143 undetermined), same data as the holotype.

**Remarks.** Originally, the holotype was mixed with other specimens.

**(672) *Zenarchopterusbasudensis* Fowler, 1934: 326, fig. 79**

= *Zenarchopteruskampeni* (Weber, 1913).

**Holotype.**USNM 93061 (male, 136.0), Canimo Pass, Basud River, Camarines Norte, Luzon, 15 Jun. 1909.

**Paratypes.**USNM 93062 (2 males + 3 females, 102.0–120.0), taken with holotype.

**Remarks.** A radiograph of the paratype is available in the USNM record.

**(673) *Zenarchopteruscagayensis* Herre, 1926b: 537**

= *Zenarchopterusphilippinus* (Peters, 1868).

**Syntypes.** Uncat. (9, 100.0–110.0), Pinacanauan River, Tuguegarao, Cagayan, Luzon.

**Remarks.** The original description did not provide catalog numbers and collection date. The type specimens were possibly lost (Kottelat 2013).

**(674) *Zenarchopterusmagatensi* s Herre, 1934: 26**

= *Zenarchopterusphilippinus* (Peters, 1868).

**Holotype.**CAS-SU 25509, branches of Magat River and its tributary creeks at Bagabay, Nueva Vizcaya, Luzon, 26 May 1931.

**Paratypes.**BMNH 1933.3.11.152–161 (10), taken with holotype.

**Other catalog number.**BMNH 1933.3.11.152–161 (NHMUK:ecatalogue:2511508).

**Remarks.**Böhlke (1953)’s catalog mentioned that the holotype and paratypes were mixed under the same catalog number (SU 25509), and the holotype cannot be easily separated from the other type specimens. The same information is found in the CAS record which recorded 24 specimens (61.0–66.0 TL); 47 presumed non-types were removed to SU 68630. A photograph and a radiograph of the holotype are available in the CAS record.

##### Family Belonidae (313)

**(675) *Rhaphiobelonedammermani* Fowler, 1934: 323, fig. 76**

= *Strongyluraleiurus* (Bleeker, 1850).

**Holotype.**USNM 93065 (85.7 BL), Taal anchorage, Balayan Bay, Luzon. 0 m, 20 Feb. 1909.

**Paratypes.**USNM 93066 (2, 56.4–77.9), taken with holotype.

**(676) *Rhaphiobelonerobusta* Schultz in Schultz et al., 1953: 164, pl. 17 (fig. a)**

= *Strongyluraincisa* (Valenciennes, 1846).

**Holotype.**USNM 141749. Off Yugui Island, Rongelap Atoll, Marshall Islands.

**Paratypes.**USNM 93064 (1), Port Dupon, Luzon, 17 Mar. 1909; USNM 93067 (1, 36.3), Pandanon Island, 24 Mar. 1909; USNM 93072 (1, 85.1), Maculabo Island, southern Luzon, 13 Jun. 1909; USNM 93073 (2), Varadero Bay, Mindoro Island, 2 m, 23 Jul. 1908.

**Remarks.** One paratype (USNM 93064) was mentioned in the original description, but it was not mentioned in the catalogs of Ibarra and Stewart (1987) and Collette et al. (1992). It was also not found in USNM record.

**(677) *Tylosurusphilippinus* Herre, 1928: 31, pl. 2**

= *Tylosuruspunctulatus* (Günther, 1872).

**Holotype.** BSMP 11084 (452.0), Coron Island, Palawan.

**Paratype.** BSMP (1, 462.0), same data as holotype.

**Remarks.** The original description did not indicate the collection date. All type specimens are presumed destroyed.

#### ﻿﻿ORDER CARANGIFORMES (67)

##### Family Carangidae (332)

**(678) *Caranxauriga* Seale, 1910: 505, pl. 6**

= *Caranxtille* Cuvier, 1833.

**Holotype.** BSMP 30 (230.0), Manila, Luzon, 21 May 1907.

**Remarks.** Objectively invalid, pre-occupied by *Caranxauriga* De Vis, 1884, replaced by *Citulavirga* Ogliby, 1915 and *Caranxmanilensis* Roxas & Martin, 1937. The type specimen was presumed destroyed.

**(679) *Caranxbutuanensis* Seale, 1910: 506, pl. 7**

= *Caranxsexfasciatus* Quoy & Gaimard, 1825.

**Holotype.** BSMP 1896 (120.0), Butuan Bay, Mindanao, 25 Sep. 1907.

**Remarks.** The type specimen was presumed destroyed.

**(680) *Caranxdeani* Jordan & Seale, 1905: 775, fig. 2**

= *Carangichthysoblongus* (Cuvier, 1833).

**Holotype.**USNM 51951 (152.4), south of Negros Island, 1901.

**Paratype.** Uncat. (1).

**Remarks.** The original description mentioned one paratype but did not provide any information. A radiograph of the holotype is available in the USNM record.

**(681) *Caranxfreeri* Evermann & Seale, 1907: 63, fig. 4**

= *Selarboops* (Cuvier, 1833).

**Holotype.**USNM 55913 (231.1), San Fabian, Pangasinan, Luzon.

**Remarks.** The original description or USNM record did not indicate the collection date.

**(682) *Caranxmanilensis* Roxas & Martin, 1937: 92**

= *Caranxtille* Cuvier, 1833.

**Holotype.** BSMP 30 (230.0), Manila, Luzon, 21 May 1907.

**Remarks.** Objectively invalid, pre-occupied by *Caranxauriga* De Vis, 1884, replaced by *Citulavirga* Ogliby, 1915 and *Caranxmanilensis* Roxas & Martin, 1937. The type specimen was presumed destroyed.

**(683) *Caranxrastrosus* Jordan & Snyder, 1908: 37, pl. 51**

= *Atropusarmatus* (Forsskål, 1775).

**Lectotype.** FMNH 55363 [ex CM 411]. Kaohsiung, Taiwan.

**Paralectotype.** Uncat. (1, 342.9), Cavite, Luzon.

**Remarks.** Only the lectotype from Taiwan has been cataloged, but not the paralectotype. The paralectotype was designated by Ho and Shao (2011), but Kottelat (2013) considered it invalid. No catalog number was provided for the paralectotype.

**(684) *Caranxscutatus* Marion de Procé, 1822: 133**

Manila Bay, Luzon.

**Remarks.** No type known. The original description did not indicate the collection date. An overlooked available name. Unknown status in Fricke et al. (2024) and further collection of specimen is needed to verify its status.

**(685) *Citulahalli* Evermann & Seale, 1907: 65, fig. 5**

= *Parastromateusniger* (Bloch, 1795).

**Holotype.**USNM 55914 (63.5), San Fabian, Pangasinan, Luzon.

**Remarks.** The original description or USNM record did not indicate the collection date.

**(686) *Citulavirga* Ogilby, 1915: 134**

= *Caranxtille* Cuvier, 1833.

**Holotype.** BSMP 30 (230.0), Manila, Luzon, 21 May 1907.

**Remarks.** A replacement name for *Caranxauriga* Seale, 1910. *Caranxmanilensis* was also designated as a replacement name by Roxas and Martin (1937) but priority was given to *Citulavirga*. The type specimen was presumed destroyed.

**(687) *Eleriaphilippina* Jordan & Seale, 1905: 774, fig. 1**

= *Scomberoidestala* (Cuvier, 1832).

**Holotype.**USNM 51945 (120.6), south of Negros Island, 1901.

**Paratypes.**CAS-SU 9131 (2) and USNM 75829 (1), Negros Island.

**(688) *Hynnismomsa* Herre, 1927e: 235, pl. 1**

= *Alectisindica* (Rüpell, 1830).

**Holotype.** BSMP 15216, Manila Market, Luzon.

**Remarks.** The original description did not indicate the collection date. The type specimen was presumed destroyed).

**(689) *Uluarichardsoni* Jordan & Snyder, 1908: 39, pl. 53**

= *Atropusmentalis* (Cuvier, 1833).

**Lectotype.** FMNH 55365 [ex CM 413]. Kaohsiung, Taiwan.

**Paralectotype.**CAS-SU 9713 (1), Cavite, Luzon, 1901.

**Remarks.** The original description mentioned a co-type (syntype) from Taiwan, and other specimens from Cavite, Luzon. The co-type from Taiwan was designated as the lectotype and a type from Cavite as the paralectotype (Ho and Shao 2011).

**(690) *Uraspispectoralis* Fowler, 1938: 46, fig. 17**

= *Uraspisuraspis* (Günther, 1860).

**Holotype.**USNM 98820 (215.0), Manila Market, Luzon, 11 Jul. 1908.

**Remarks.** A radiograph of the holotype is available in the USNM record.

#### ﻿﻿ORDER ANABANTIFORMES (69)

##### Family Osphronemidae (339)

**(691) *Osphromenusinsulatus* Seale, 1910: 530**

= *Trichopodustrichopterus* (Pallas, 1770).

**Holotype.** BSMP 4951 (73.0), lake on Cagayan-Sulu Island, Sulu Archipelago.

**Paratypes.** BSMP (many).

**Remarks.** The original description did not indicate the collection date. All type specimens were lost (Kottelat 2013).

##### Family Channidae (340)

**(692) *Ophiocephalusvagus* Peters, 1868: 260**

= *Channastriata* (Bloch, 1793).

**Syntypes.** ZMB 1397–98 [ex MNHN] (1, 1), ZMB 1399 (1), ZMB 6511–21 (1, 3, 1, 2, 1, 1, 2, 9, 2, 1, 8), ZMB 6524 (1), ZMB 6528 (1), ZMB 6531 (1), ZMB 6539 (1), ZMB 6547 (1), ZMB 6725–26 (1, 2), ZMB 7160 (1), ZMB 15593 (20), and ZMB 31912 (1).

**Remarks.** The original description mentioned many localities but did not specify the locality of each syntype and collection date. Localities mentioned include Lakes Bato and Buhi and Calumpit in Luzon, and Samar and Leyte islands.

#### ﻿﻿ORDER PLEURONECTIFORMES (70)

##### Family Citharidae (345)

**(693) *Brachypleuropsaxillaris* Fowler, 1934: 341, fig. 95**

= *Citharoidesmacrolepidotus* Hubbs, 1915.

**Holotype.**USNM 93080 (195.0TL), Albatross station 5117, Sombrero Island, Balayan Bay and Verde Island Passage, Luzon, 216 m, 21 Jan. 1908.

**Remarks.** Radiographs of the holotype are available in the USNM record.

##### Family Paralichthyidae (347)

**(694) *Pseudorhombusmegalops* Fowler, 1934: 329, fig. 83**

**Holotype.**USNM 93082 (220.0 TL), Albatross station 5392 (12°12.58'N, 124°02.80'E), Tubig Point, between Samar and Masbate islands, 247 m, 13 Mar. 1909.

**Paratypes.**USNM 93550 (2), same data as holotype.

**(695) *Platophryspalad* Evermann & Seale, 1907: 105, fig. 21**

= *Pseudorhombusdupliciocellatus* Regan, 1905.

**Holotype.**USNM 55898 (393.7), Bulan, Sorsogon, Luzon.

**Remarks.** The original description or USNM record did not indicate the collection date.

##### Family Bothidae (349)

**(696) *Arnoglossusmaculipinnis* Fowler, 1934: 329, fig. 84**

= *Engyprosoponmaldivense* (Regan, 1908).

**Holotype.**USNM 93093 (101.0 TL), Albatross station 5140 (06°08.75'N, 121°03.0'E), Jolo Island, Sulu Archipelago, 139 m, 14 Feb. 1908.

**Paratypes.**USNM 93569 (1), Albatross station 5134, Balukbaluk Island, near Basilan, Sulu Archipelago, 46 m, 7 Feb. 1908; USNM 93570 (2), Albatross station 5480, Tabuc Point, Surigao Strait, between Samar and Leyte islands, 113 m, 29 Jul. 1909.

**Remarks.** The original description mentioned USNM 93098 as the holotype, but it is erroneous as this is a specimen of *Myliobatiscalifornica* based on the USNM record. The correct catalog number is USNM 93093. Radiographs of the holotype are available in the USNM record.

**(697) *Asterorhombusfilifer* Hensley & Randall, 2003: 2, figs 1, 2, 3A**

**Holotype.** BPBM 34871. Midway Atoll, Hawaiian Islands.

**Paratypes.** SAIAB (RUSI) 52650 (1 male, 63.2), Bolinao, Pangasinan, Luzon, 30–35 m, 8 Oct. 1995; USNM 260363 (1 female, 46.5), east of Bais, Negros Island, 0–37 m, 17 Jun. 1978.

**Remarks.** The USNM record indicated that 260363 was discarded while on loan to Dannie Hensley.

**(698) *Bothusbrunneus* Fowler, 1934: 331, fig. 85**

= *Arnoglossusbrunneus* (Fowler, 1934).

**Holotype.**USNM 93074 (183.0 TL), Albatross station 5453, Legaspi Lighthouse, east of Luzon, 267 m, 7 Jun. 1909.

**Paratypes.**USNM 93075 (3) and USNM 93538 (1), same locality as the holotype; USNM 93533 (2), Albatross station 5273, Corregidor Lighthouse, southern Luzon, 208 m, 14 Jul. 1908; USNM 93534 (4), Albatross station 5121, Malabrigo Lighthouse, east of Mindoro Island, 198 m, 2 Feb. 1908; USNM 93535 (1), Albatross station 5418; USNM 93536 (1), Albatross station 5417; USNM 93537 (2), Albatross station 5411; and USNM 93539 (1), Albatross station 5412, Lauis Point Lighthouse, between Cebu and Bohol islands, 265–302 m, 23–25 Mar. 1909; USNM 93540 (2), Albatross station 5255, Dumalag Island, Davao Gulf, Mindanao, 183 m, 18 May 1908; USNM 93542 (1), Albatross station 5376, Tayabas Island, Marinduque Islands, 165 m, 2 Mar. 1909; USNM 93543 (1), Albatross station 5117, Sombrero Island, between Balayan Bay and Verde Island Passage, Luzon, 216 m, 21 Jan. 1908; USNM 93541 (1) and USNM 93544 (3), Albatross stations 5243-44, Uanivan Island, Pujada Bay, Mindanao, 313–399 m, 15 May 1908.

**Remarks.** Radiographs of the holotype are available in the USNM record.

**(699) *Bothusobliquioculatus* Fowler, 1934: 332, fig. 87**

= *Engyprosoponobliquioculatum* (Fowler, 1934).

**Holotype.**USNM 93077 (78.0 TL), Philippines.

**Paratypes.**USNM 93078 (4), same data as holotype.

**Remarks.** The original description mentioned four paratypes but did not provide the catalog number. The specific locality and collection date was also not listed in the original description or USNM record. All type specimens were taken between 7 Nov. 1907 and 29 Jan. 1910. A photograph and radiograph of the holotype specimens are available in the USNM record.

**(700) *Bothustchangi* Fowler, 1934: 334, fig. 88**

**Holotype.**USNM 93076 (210.0 TL), Albatross station 5475 (12°55.43'N, 124°12.20'E), east of Luzon, 357 m, 24 Jun. 1909.

**Remarks.** Unknown status in Fricke et al. (2024). Further examination of specimens is needed to confirm its status. Radiographs of the holotype are available in the USNM record.

**(701) *Bothusvariegatus* Fowler, 1934: 335, fig. 89**

= *Psettinavariegata* (Fowler, 1934).

**Holotype.**USNM 93091 (92.0), Albatross station 5481 (10°27.50'N, 125°17.17'E), Cabugan Grande Island, between Samar and Leyte islands, 112 m, 30 Jul. 1909.

**Remarks.** Radiographs of the holotype are available in the USNM record.

**(702) *Laeopsclarus* Fowler, 1934: 337, fig. 90**

**Holotype.**USNM 93083 (105.0 TL), Albatross station 5412 (10°09.25'N, 123°52.0'E), Lauis Point Lighthouse, between Cebu and Bohol islands, 296 m, 23 Mar. 1909.

**Remarks.** Radiographs of the holotype are available in the USNM record.

**(703) *Laeopscypho* Fowler, 1934: 337, fig. 91**

= *Neolaeopsmicrophthalmus* (von Bonde, 1922).

**Holotype.**USNM 93085 (149.0 TL), Albatross station 5519 (08°47.00'N, 123°31.25'E), Tagolo Point Lighthouse, off northern Mindanao, 333 m, 9 Aug. 1909.

**Paratypes.**USNM 93566 (1), Albatross station 5376, Sombrero Island, between Balayan Bay and Verde Island Passage, Luzon, 216 m, 21 Jan. 1908; USNM 93567 (1), Albatross station 5117, Tayabas Lighthouse, Marinduque Island, 216 m, 2 Mar. 1909.

**Remarks.** The original description mentioned only one paratype but the USNM record indicates two paratypes under this name. Radiographs of the holotype are available in USNM record.

**(704) *Laeopsgracilis* Fowler, 1934: 338, fig. 92**

= *Japonolaeopsgracilis* (Fowler, 1934).

**Holotype.**USNM 93084 (165.0 TL), Albatross station 5212 (12°04.25'N, 124°04.60'E), east of Masbate Island, Luzon, 198 m, 20 Apr. 1908.

**Remarks.** Radiographs of the holotype are available in the USNM record.

##### Family Poecilopsettidae (351)

**(705) *Nematopschui* Fowler, 1934: 338, fig. 93**

= *Nematopsmacrochirus* Norman, 1931.

**Holotype.**USNM 93087 (82.0 TL), Albatross station 5110, off southern Luzon, 247 m, 15 Jan 1908.

**Remarks.** The original description mentioned China Sea as the type locality and was taken on 15 Jan. 1908. However, USNM record indicated that the type specimen was taken from Cebu Market, Cebu Island on 5 Apr. 1908. Radiographs of the holotype are available in the USNM record (Smithsonian Museum Archive 2024).

**(706) *Poecilopsettadorsialta* Guibord & Chapleau, 2001: 1081, fig. 1**

**Holotype.**USNM 150696 (male, 98.8), Albatross station 5421 (10°33.50'N, 122°26.00'E), Lusaran Point, between Panay and Guimaras islands, 250 m, 30 Mar. 1909.

**Paratypes.**USNM 363487 [ex USNM 150696] (male, 103.9), same data as holotype; USNM 361865 [ex USNM 138005] (male, 96.6), Albatross station 5516, Tagolo Point Lighthouse, northern Mindanao, 320 m, 9 Aug. 1909; USNM 138008 (male, 96.8), Albatross station 5247, Dumalag Island, Davao Gulf, Mindanao, 247 m, 18 May 1908; USNM 138013 (male, 111.6), Albatross station 5372, Tayabas Bay, Marinduque Island, 275 m, 24 Feb. 1909; USNM 138015 (1 male, 79.3 + 1 female, 75.4), Albatross station 5256, Utara Point, Bongo Island, east of Illana Bay, southern Mindanao, 289 m, 22 May 1908.

**(707) *Poecilopsettamegalepis* Fowler, 1934: 340, fig. 94**

= *Poecilopsettaplinthus* (Jordan & Starks, 1904).

**Holotype.**USNM 93094 (128.0 TL), Albatross station 5117 (13°52.37'N, 120°46.37'E), Sombrero Island, Balayan Bay and Verde Island Passage, Luzon, 216 m, 21 Jan. 1908.

**Paratypes.**USNM 93572 (1), Albatross station 5369, Tayabas Lighthouse, Marinduque Island, 194 m, 24 Feb. 1909; USNM 93571 (1) and USNM 93573-75 (3, 2, 3), Albatross station 5411, Lauis Point Lighthouse, between Cebu and Bohol islands, 265–302 m, 23–25 Mar. 1909; USNM 93576 (1), Albatross station 5353, Cape Melville Lighthouse, northern Balabac Strait, Palawan, 271 m, 1 Jan. 1909; USNM 93577 (1), Albatross station 5255, Dumalag Island, Davao Gulf, Mindanao, 183 m, 18 May 1908; USNM 93578 (1), Albatross station 5279, Malavatuan Island, South China Sea, southern Luzon, 214 m, 17 Jul. 1908.

**Remarks.** The original description did not mention the occurrence of paratypes but was found in USNM record. Radiographs of the holotype are available in the USNM records (Smithsonian Museum Archive 2024).

##### Family Samaridae (354)

**(708) *Samariscusluzonensis* Fowler, 1934: 343, fig. 96**

**Holotype.**USNM 93089 (76.0 TL), Albatross station 5442 (16°30.60'N, 120°11.10'E), San Fernando Point Lighthouse, west of Luzon, 83 m, 10 May 1909.

**Remarks.** Radiographs of the holotype are available in the USNM records.

**(709) *Samariscusmacrognathus* Fowler, 1934: 343, fig. 97**

**Holotype.**USNM 93088 (54.0 TL), Albatross station 5442 (16°30.60'N, 120°11.10'E), San Fernando Point Lighthouse, west of Luzon, 83 m, 10 May 1909.

**Remarks.** Radiographs of the holotype are available in the USNM records.

##### Family Soleidae (356)

**(710) *Aseraggodeskimurai* Randall & Desoutter-Meniger, 2007: 312, fig. 7**

**Holotype.** BPBM 22165 (female, 52.6), Dumaguete fish market, Negros Island, 28 Aug. 1977.

**Paratype.**USNM 385814 (1 male, 71.7), same data as holotype.

**(711) *Aseraggodesmatsuurai* Randall & Desoutter-Meniger, 2007: 315, figs 9, 10**

**Holotype.** NSMT-P-71517. Kodek Bay, Lombok, Indonesia.

**Paratypes.**CAS 46099 (1, male, 55.1), Dumaguete, Negros Island, 10 Jul. 1948; USNM 273855 (female, 49.6), Port Siaton, Negros Island, 0–3 m, 28 Apr. 1979; ROM 48617 (female, 37.5), Mactan Island, Cebu, 12–18 m, 8 Aug. 1985.

**(712) *Aseraggodeswinterbottomi* Randall & Desoutter-Meniger, 2007: 325, fig. 22**

**Holotype.** ROM 55042 (male, 41.5), 09°37.00'N, 123°10.00'E, mouth of Bais Bay, Negros Island, 7.5 m, 17 May 1987.

**Paratypes.** BPBM 40444 (male, 46.3), Tonga Point, Siquijor Island, 7.5–15 m, 14 May 1987; ROM 55040 (male, 44.0), same locality as BPBM 40444, 4.5–12 m, 9 May 1987.

**(713) *Brachirusmegalepidoura* Fowler, 1934: 347, fig. 100**

**Holotype.**USNM 93081 (243.0 TL), Albatross station 5204, Mariquitdaquit Island, off east of Leyte Island, 27 m, 11 Apr. 1908.

**Paratypes.**USNM 93552 (2), Albatross station 5461, Caringo Island, east of Luzon, 20 m, 14 Jun. 1909; USNM 93553 (2), Albatross station 5204, Mariquitdaquit Island, off east of Leyte Island, 27 m, 11 Apr. 1908; USNM 93554 (1), Albatross station 5209, Taratara Island, off western Samar, 0–37 m, 14 Apr. 1908.

**Remarks.** Unknown status in Fricke et al. (2024). Further examination of specimens is needed to confirm its status.

**(714) *Pleuronectesargentea* Bonnaterre, 1788: 77**

Philippines.

**Remarks.** No type known. Desoutter et al. (2001) mentioned that the species was based on the *Soleaphilippensisargentea* of Petiver (1702) but was not examined. Unknown status in Fricke et al. (2024). Further study is needed to verify its status.

**(715) *Synapturasorsogonensis* Evermann & Seale, 1907: 106, fig. 22**

= *Brachirusaspilos* (Bleeker, 1852).

**Holotype.**USNM 55916 (228.6), Bacon, Sorsogon, Luzon.

**Remarks.** The original description did not indicate the collection date.

**(716) *Zebriaslucapensis* Seigel & Adamson, 1985: 13, fig. 1**

**Holotype.** LACM 37436-6 (83.5), Hundred Islands, Lucap Bay, Lingayen Gulf, Luzon, 9 Mar. 1978.

**Paratype.** LACM 37346-8 (1, 83.0), same data as holotype.

##### Family Cynoglossidae (357)

**(717) *Cynoglossussuyeni* Fowler, 1934: 347, fig. 101**

**Holotype.**USNM 93086 (155.0 TL), Albatross station 5291 (13°29.67'N, 121°00.75'E), Escarceo Lighthouse, southern Luzon, 316 m, 23 Jul. 1908.

**Remarks.** A radiograph of the holotype is available in the USNM record.

**(718) *Symphurusbathyspilus* Krabbenhoft & Munroe, 2003: 811, fig. 1**

**Holotype.**USNM 113185 (96.6), Albatross station 5503 (08°36.43'N, 124°36.13'E), Macabalan Point Lighthouse, northern Mindanao, 416 m, 4 Aug. 1909.

**Paratypes.** AMS I.43130-001 (3, 86.9–92.4), MNHN 50-1 (1, 100.2), MNHN 50-3 (1, 99.5), MNHN 2003-1078 (3, 87.5–94.1), USNM 138060 (24, 79.4–102.6) and USNM 138061 (16, 71.0–102.4), same data as holotype.

**(719) *Symphurusleptosomus* Lee & Munroe, 2021: 32, fig. 9**

**Holotype.**USNM 379566 (male, 23.6), 13°13.25'N, 120°33.56'E, west Mindoro Island, 15 m, 3 Jun. 2000.

**Remarks.** The USNM record shows that USNM 379566 is a non-type specimen of *Symphurusmicrorhynus*.

**(720) *Symphurusluzonensis* Chabanaud, 1955: 32**

**Holotype.**USNM 138043 (male, 72 .0), Albatross station 5268, Matocot Point, Batangas Bay and Verde Island Passage, Luzon, 311 m, 8 Jun. 1908.

**Remarks.**USNM 163654 was removed from USNM 138043, identified as *Symphurusseptemfasciatus*.

**(721) *Symphurusmarmoratus* Fowler, 1934: 349, fig. 102**

**Holotype.**USNM 93092. Cape Lassa, Flores Sea, Indonesia.

**Paratype.**USNM 93208 (1), Albatross station 5546, Noble Point, Tulayan Island, Jolo, Sulu Archipelago, 252 m, 15 Sep 1909.

**Remarks.** The original description mentioned that the holotype (USNM 93092) was taken at Jolo Island (station D 5561) on 18 Sep. 1909. However, USNM record indicated that USNM 93092 was taken from Cape Lassa, Flores Sea (station D 5661) on 20 Dec. 1909 (Smithsonian Museum Archive 2024).

**(722) *Symphurusschultzi* Chabanaud, 1955: 31**

**Holotype.**USNM 138044 (female), Albatross station 5508, Camp Overton Lighthouse, northern Mindanao, 494 m, 5 Aug. 1909.

**Paratypes.**USNM 138025 (1), Albatross station 5201, Limasaua Island, Sogod Bay, Leyte, 1013 m, 10 Apr. 1908; USNM 138033 (1), Albatross station 5373, Tayabas Lighthouse, Marinduque Island, 618 m, 2 Mar. 1909; USNM 138046 (1), Albatross station 5536, Apo Island, between Negros and Siquijor islands, 510 m, 19 Aug. 1909; USNM 138057 (1), Albatross station 5406, Macabalan Point Lighthouse, northern Mindanao, 479 m, 5 Aug. 1909.

**Remarks.** The original description mentioned 5 specimens (2 males + 3 females, 64.0–70.0) but did not provide the specific size of each specimen; it is also not indicated in USNM record. Two specimens mixed with holotype were re-identified as re-cataloged: one as *Symphurusseptemstriatus* to USNM 163655 and one as *Symphurusbathyspilus* to USNM 163656.

#### ﻿﻿ORDER SYNGNATHIFORMES (71)

##### Family Solenostomidae (359)

**(723) *Solenostomusphantasticus* Herre, 1933: 17**

= *Solenostomusparadoxus* (Pallas, 1770).

**Holotype.**CAS-SU 25514 (female, 72.0), eel grass in Dumaguete, Negros Island, 8 Jun. 1931.

**Remarks.** A photograph and a radiograph of the holotype are available in the CAS record.

##### Family Syngnathidae (360)

**(724) *Bombonialuzonica* Herre, 1927b: 275, pl. 2 (figs 1, 2)**

= *Hippichthysheptagonus* Bleeker, 1849.

**Holotype.** BSMP (male, 94.0), Lake Bombon (Taal), between Ambulong and Talisay, Batangas, Luzon.

**Paratypes.**BMNH 1933.3.11.205–207 (3) and BSMP (~29) (43.0–87.0 + 1 male, 85.0), same data as holotype.

**Other catalog number.**BMNH 1933.3.11.205–207 (NHMUK:ecatalogue:2511526).

**Remarks.** The collection date was not indicated in the original description. BSMP types were lost (Kottelat 2013).

**(725) *Corythroichthyselerae* Evermann & Seale, 1907: 57, fig. 2**

= *Corythoichthyshaematopterus* (Bleeker, 1851).

**Holotype.**USNM 55908 (120.7), Bacon Island, Sorsogon, Luzon.

**Paratypes.**CAS-SU 20001 (1), MCZ 32190 [ex USNM 3898] (1, 104.0), USNM 55921 (1) and USNM 126383 (1), same locality as the holotype; ANSP 33252 [ex USBF 3898] (1), CAS 33313 [ex IU 3898] (1), FMNH 5895 (1) and Mus. in Manila (1), unknown localities.

**Remarks.** The original description or USNM record did not indicate the collection date.

**(726) *Corythroichthysmatterni* Fowler, 1918: 11, fig. 5**

= *Hippichthysheptagonus* Bleeker, 1849.

**Holotype.**ANSP 47484 (138.0), Philippines.

**Remarks.** The original description or ANSP did not provide a specific locality and collection date.

**(727) *Corythroichthyspullus* Smith & Seale, 1906: 75, unnumbered fig.**

= *Hippichthysheptagonus* Bleeker, 1849.

**Holotype.**USNM 55621 (142.2), Rio Grande River, Cotabato, Mindanao, Oct. 1903.

**(728) *Doryichthysphilippinus* Fowler, 1918: 13, fig. 6**

= *Microphisbrachyurus* (Bleeker, 1854).

**Holotype.**ANSP 47485 (173.0), Philippines.

**Remarks.** The original description or ANSP did not provide a specific locality and collection date. Photographs and radiographs are available in ANSP’s record.

**(729) *Doryichthysspaniaspis* Jordan & Seale, 1907: 10, fig. 3**

= *Hippichthyscyanospilos* (Bleeker, 1854).

**Holotype.**CAS-SU 9240 (107.7), Cavite, Luzon, 1900.

**(730) *Doryrhamphusmacgregori* Jordan & Richardson, 1908: 246, fig. 7**

= *Choeroichthyssculptus* (Günther, 1870).

**Holotype.**CAS-SU 20202 (probably female, 38.1), Calayan Island, Cagayan, Luzon.

**Remarks.** The collection date was not indicated in the original description. Böhlke (1953) mentioned that the original description mistakenly gives USNM 20202 for the type.

**(731) *Doryrhamphusnegrosensis* Herre, 1934: 28**

**Holotype.**CAS-SU 25503 (45.0), tide pool near Dumaguete, Negros Island, Jun. 1931.

**Paratypes.**CAS-SU 69776 [ex CAS-SU 25503] (1), same data as holotype.

**Remarks.** A photograph and a radiograph of the holotype are available in the CAS record.

**(732) *Dunckerocampuspessuliferus* Fowler, 1938: 41, fig. 13**

**Holotype.**USNM 93501 (110.0), Albatross station 5146 (05°46.67'N, 120°48.83'E), off Sulade Island, Siasi, Sulu Archipelago, 44 m, 16 Feb. 1908.

**Remarks.** A photograph and a radiograph of the holotype are available in the USNM record.

**(733) *Festucalexprolixus* Dawson, 1984: 371, figs 1–3**

**Holotype.** ZMUC P39741 (36.2), Dana station 3685 III (07°22.00'N, 121°16.00'E), SE Sulu Sea, west of Zamboanga, 0–100 m over 4825 m, 5 Apr. 1929.

**Paratypes.** GCRL 20922 (1, 26.5) taken with holotype; GCRL 20921 (1, 29.6) and ZMUC P. 39742 (1, 29.7), Dana station 3738 III (06°29.0'N, 122°27.0'E), east of Basilan Island, 0–100 m over 3300 m, 1 Jul. 1929.

**Remarks.** The original description or USNM record did not indicate the collection date.

**(734) *Hemithylacusrocaberti* Dumeril, 1870: 600**

= *Belonichthysmento* (Bleeker, 1856).

**Syntypes.**MNHN A-1701 (1 female, 104.5) and MNHN 0000-2085 (2), 14°36.00'N, 120°58.98'E, Manila, Luzon.

**Remarks.** The original description did not indicate the collection date but mentioned the largest specimen at 125.0.

**(735) *Hippocampusbarbouri* Jordan & Richardson, 1908: 247, fig. 8**

**Holotype.**USNM 61683 (101.6), Cuyo Island, Palawan.

**Paratypes.**CAS-SU 20205 (2, 101.6), same data as holotype.

**(736) *Ichthyocampusdavaoensis* Herald in Schultz et al., 1953: 242, fig. 37**

= *Bulbonaricusdavaoensis* (Herald, 1953).

**Holotype.**USNM 112295 (33.0), 06°44.00'N, 125°46.00'E, Davao Gulf, Mindanao, 26 Feb. 1948.

**(737) *Ichthyocampusphilippinus* Fowler, 1938: 43**

= *Festucalexerythraeus* (Gilbert, 1905).

**Holotype.**USNM 94080 (55.0), Albatross station 5160 (05°12.67'N, 119°55.17'E), off Tinakta Island, Sulu Archipelago, 22 m, 22 Feb. 1908.

**(738) *Micrognathusbrachyrhinus* Herald, 1953: 262, fig. 39f**

= *Minyichthysbrachyrhinus* (Herald, 1953).

**Holotype.**USNM 118082. Oahu Island, Hawaiian Islands.

**Paratype.**USNM 137269 (1, 22.0), San Miguel Harbor, Ticao Island, Masbate, 21 Apr. 1908.

**Remarks.** The original description mentioned only one paratype from Ticao Island. However, the CAS record also indicated SU 24763 as a paratype of *M.brachyrhinus* with the same collection data as USNM 137269. However, USNM 137269 is not found in the USNM record, and it is unknown whether the two specimens are identical with two different catalog numbers.

**(739) *Micrognathusmagdamoi* Herre, 1932: 141**

= *Halicampusdunckeri* (Chabanaud, 1929).

**Holotype.**CAS-SU 27716 (male, 110.0), Dumaguete, Negros Island, 8 Jun. 1931.

**Paratype.**CAS-SU 69829 [ex CAS-SU 27716] (1 female, 115.0), same data as holotype.

**Remarks.**Böhlke (1953) indicated that the collection date was 8 Jun. 1929. The CAS records indicate the holotype has embryos, one paratype was removed to SU 69829 and one non-type was removed to SU 69830. A photograph and a radiograph of the holotype are available in the CAS record.

**(740) *Micrognathusnatans* Dawson, 1982: 682, figs 12, 13**

**Holotype.** AMS I.20390-012. Beqa Island, Fiji Islands.

**Paratypes.**USNM 133055 [ex USNM 138828] (1, 33.5), Varadero Bay, Mindoro Island, 0 m, 22 Jul. 1908; USNM 230674 [ex USNM 139102] (1, 33.5), Varadero Bay, Mindoro Island, 20–23 Jul. 1908; USNM 230673 [ex USNM 139080] (1, 33.5), Mansalay Anchorage, off southeastern Mindoro Island, 16 m, 3 Jun. 1908; ZMUC P.39700 (1, 26.0), Dana station 3733 (13°32.00'N, 121°21.00'E), south of Luzon, 0–100 m, 26 Jun. 1929; ZMUC P.39701 (1, 30.0) and ZMUC P.39702 (1, 28.0), Dana station 3734 (11°43.00'N, 121°43.00'E), off northwest tip of Panay Island, 0–200 m, 27 Jun. 1929.

**(741) *Microphiscaudatus* Peters, 1868: 276**

= *Lophocampusretzii* (Bleeker, 1856).

**Syntypes.** ZMB 6646 (1) and ZMB 6686 (1), freshwater stream in Loquilocun, Samar Island; Java, Indonesia.

**Remarks.** The original description mentioned three specimens, two (male and female) from Samar Island but did not provide which of the catalog numbers contained specimens from the Philippines, including the collection dates.

**(742) *Microphisjagorii* Peters, 1868: 280**

**Holotype.** ZMB 6647 (14.6), Loquilócun, Samar Island.

**Remarks.** The original description did not indicate the collection date.

**(743) *Microphispleurostictus* Peters, 1868: 278**

**Syntypes.** ZMB 6633 (8, in 2 bottles), ZMB 6634 (?) Batu, ZMB 6692 (2) Yassot, BMNH 1868.7.10.1 (5), MNHN 0000-6036 (3); All type specimens from Lake Bato in Camarines Sur, and Yassot Creek in Albay, Luzon.

**Other catalog number.**BMNH 1868.7.10.1 (NHMUK:ecatalogue:3102244).

**Remarks.** The original description did not indicate the collection date. Two specimens share BMNH 1868.7.10.1 as catalog number in NHM’s record.

**(744) *Parabelonichthyskellersi* Fowler, 1943: 58, fig. 7**

= *Belonichthysmento* (Bleeker, 1856).

**Holotype.**USNM 108466 (118.0), Jaro River, Iloilo, Panay Island, 3 Apr. 1929.

**Paratypes.**USNM 119443 (1, 92 .0), same locality as holotype, 17 Apr. 1929; USNM 108468 (2, 110.0–118.0), Cebu Island, 17 Apr. 1929.

**(745) *Siokunichthysherrei* Herald in Schultz et al., 1953: 254, fig. 38**

**Holotype.**USNM 112296 (73.5), 07°43.40'N, 122°04.70'E, north of Siocon Bay, NW Zamboanga, Mindanao, 37 m, 18 Feb. 1948.

**Paratypes.** SAIAB [ex RUSI] 146 [ex USNM 112297] (1, 72.5), same data as holotype; USNM 112298 (2, 69.5–72.0), southwest of Panay Island, 22 m, 3 Apr. 1948.

**Remarks.** The original description mentioned USNM 112297 twice with different localities. One is assumed to be USNM 112298 as the locality and several specimens of one paratype specimens were similar to the second USNM 112297 mentioned in the original description.

**(746) *Siokunichthysnigrolineatus* Dawson, 1983: 58, figs 3–5**

**Holotype.**BMNH 1982.6.17.59. Belang-belang Island, Molucca Islands, Indonesia.

**Paratypes.** GCRL 18829 (1 male, 67.5) and USNM 236539 (1 female, 74.0), 300 m southwest of harbor entrance, Bonbonon Bay, Negros Island, 13.1 m, 28 Apr. 1979; GCRL 19192 (1 female, 65.5), Anilao, Batangas, Luzon, 20 m, 31 Jan. 1981.

**(747) *Syngnathusmicronotopterus* Fowler, 1938: 42, fig. 14**

= *Micrognathusmicronotopterus* (Fowler, 1938).

**Holotype.**USNM 94082 (56.0), Canimo Island near Daet Point, Luzon, 15 Jun. 1909.

**Paratypes.**USNM 99007 [ex USNM 94082] (1), same locality as holotype, 15 Jun. 1909; USNM 94087 (1), Batan Island, Batanes, 22 Jul. 1909; USNM 94085 (1), Mactan Island, Cebu, 7 Apr. 1908.

**Remarks.** Two specimens were listed in the ledger for USNM 94082, one being USNM 99007.

**(748) *Trachyrhamphuscaba* Seale, 1910: 503**

= *Halicampusgrayi* Kaup, 1856.

**Holotype.** BSMP 2324 (140.0), Balayan Bay, Luzon, 20 Jan. 1908.

**Remarks.** The type specimen was presumed destroyed.

##### Family Dactylopteridae (365)

**(749) *Dactyloptenatiltoni* Eschmeyer, 1997: 731, figs 2B, 3**

**Holotype.**CAS 32903 (94.0), Buri Point, Ragay Gulf, Luzon, 556–565 m, 15 Nov. 1966.

**Paratypes.**CAS 47964 (1, 94.5), taken with holotype; CAS 33853 (1, 94.0), southeast of Salomague Island, Marinduque, 287–313 m, 20 Oct. 1966; CAS 32799 (1, 73.5), same locality as CAS 33853, 119–132 m, 25 Aug. 1966; CAS 33365 (1, 90.2), Sandoval Point, Catanuan, Quezon, Luzon 128–143 m, 3 Nov. 1966; CAS 32684 (2, 66.8–70.7), southeast of Talaga, Batangas Bay, Luzon, 240–252 m, 26 Jul. 1966; CAS 32807 (1, 74.2), Mainaga Cove, Batangas Bay, Luzon, 159–187 m, 8 Jul. 1966; CAS 32893 (1, 70.6), CAS 47986 (1, 65.5) and AMS 1.26946-001 (1, 74.5), Balayan Bay, Luzon, 155–165 m, 27 Jun. 1966.

##### Family Callionymidae (367)

**(750) *Anaorafowleri* Herre, 1953a: 781**

= *Anaoratentaculata* Gray, 1835.

**Holotype.** BSMP 7302 (male, 34.5), Puerto Galera, Mindoro Island, May 1912.

**Paratype.** BSMP (female, 32.0), same data as holotype.

**Remarks.** A replacement name for *Synchiropustentaculatus* Herre, 1928 which was preoccupied by *Anaoratentaculata* Gray, 1835 when both are in *Anaora*. All type specimens are presumed destroyed.

**(751) *Brachycallionymusmirus* Herre, 1936a: 12**

= *Eleutherochiropercularis* (Valenciennes, 1837).

**Holotype.**CAS-SU 30978. Sulawesi, Indonesia.

**Paratypes.**USNM 98827 (2, 16.0), Romblon, 25 Mar. 1908; USNM 98828 (1, 15.0), Nasugbu Bay, Luzon, 18 m, 15 Jan. 1908.

**(752) *Callionymusacutirostris* Fricke, 1981: 153, fig. 7**

**Holotype.**CAS 32642 (male, 25.2), 2.5 miles west of Calaguaguin Cove, Zambales, Luzon, 64–81 m, 9 Jun. 1966.

**(753) *Callionymusboleogenys* Fowler, 1941: 6, fig. 3**

= *Callionymussimplicicornis* Valenciennes, 1837.

**Holotype.**USNM 99408 (51.0), Pandanan Island, between Cebu and Bohol islands, 40 m, 23 Mar 1909.

**Paratype.**USNM 99409 (1), same data as holotype.

**(754) *Callionymusbrunneus* Fowler, 1941: 11, fig. 7**

= *Callionymusfilamentosus* Valenciennes, 1837.

**Holotype.**USNM 99419 (80.0), near Taal Beach, Balayan Bay and Verde Island Passage, Luzon, 19 Jan. 1908.

**Paratypes.**USNM 99420 (7, 67.0–78.0), same data as holotype; USNM 99421 (2, 34.0–53.0), Subig Bay, southern Luzon, 6 m, 7 Jan. 1908.

**(755) *Callionymusdistethommatus* Fowler, 1941: 18, fig. 11**

= *Callionymusenneactis* Bleeker, 1879.

**Holotype.**USNM 99426 (male, 69.0), Cebu market, Cebu, 28 Aug. 1909.

**Paratypes.**CAS-SU 40196 [ex USNM 99431] (1 male, 41.0), USNM 99431 (1 male, 41.0), 28 Mar. 1909; USNM 99427 (2 males, 45.0), USNM 99428 (3 males, 52.0–58.0), 27–28 Aug. 1909 and USNM 99429 (1 female, 40.0), 20 Mar. 1909, same locality as holotype; USNM 99430 (2 females, 35.0–42.0), Port Matalvi, off western Luzon, 4.5 m, 23 Nov. 1908; USNM 99432 (2 males, (48.0–59.0), Guijulugan, eastern Negros Island, 2 Apr. 1908.

**(756) *Callionymusfimbriatus* Herre, 1934: 94**

= *Anaoratentaculata* Gray, 1835.

**Holotype.**CAS-SU 25516 (female, 27.8), Sitankai Island, Sulu Archipelago, Aug. 1931.

**Remarks.** A photograph and a radiograph of the holotype are available in the CAS record.

**(757) *Callionymusguentheri* Fricke, 1981: 370, figs 15–17**

**Holotype.**BMNH 1879.5.14.567 (female, 87.33), entrance from the Sulu Sea into Basilan Strait, west of Zamboanga, Mindanao, 150 m, 26 Oct. 1874.

**Paratypes.**CAS 32668 (2 females, 94.5–102.0), southeast of Talaga, Batangas Bay, Luzon, 240–252 m, 26 Jul. 1966; CAS 34426 (female, 84.1), south of Bauan, Batangas Bay, Luzon, 161–166 m, 4 Jul. 1966; CAS 34468 (female, 102.8), Lemery Town, Batangas Bay, Luzon, 155–166 m, 27 Jul. 1966; CAS 32897 (7 males + 4 females, 51.2–128.0), Lemery, Balayan Bay, Luzon, 155–166 m, 27 Jun. 1966; CAS 32801 (male, 91.9), northeast of Salomague Island, Marinduque, 260–274 m, 25 Aug 1966; CAS 33879 (female, 112.8) and CAS 34286 (3 males, 100.0–108.3), north of San Andres Island, Marinduque, 251–289 m, 14–15 Dec. 1966; CAS 34154 (7 females, 91.1–129.2), same locality as CAS 33879, 198–205 m, 3 Sep 1966; CAS 34704 (female, 123.2), north of Sayao Bay, Marinduque, 112–128 m; CAS 34205 (female, 127.0), northwest of Baltazar Island, Marinduque, 260–274 m, 10 Dec. 1966; CAS 34278 (4 females, 89.9–124.5), north of Melchor Island, Marinduque, 219–230 m, 11 Dec. 1966; CAS 32997 (female, 107.5), south of Bario Sinisian, Balayan Bay, Luzon, 174–181 m, 15 Jun. 1966; CAS 33703 (female, 122.0), south of Barrio Nomong Castro, Balayan Bay, Luzon, 183–192 m, 25 Jun 1966; CAS 34401 (female, 117.7), southeast of Calaca Town, Balayan Bay, Luzon, 101–119 m, 24 Jun 1966; CAS 32905 (4 males + 3 females, 69.3–117.2), Buri Point, Ragay Gulf, Luzon, 556–565 m, 15 Nov. 1966; CAS 32916 (5 females, 91–118.2), Pusgo Point, Ragay Gulf, Luzon, 110–123 m, 11 Nov. 1966; CAS 33864 (3 females, 90.0–127.3), Caurusan Point, Ragay Gulf, Luzon, 552–563 m, 23 Nov. 1966; CAS 34190 (1 male, 113.2 + 1 female, 99.7), southeast of Alibijaban Island, Ragay Gulf, Luzon, 148–161 m, 6 Nov. 1966; CAS 34197 (1 male + 8 females, 90.2–129.3), Siburio Point, Ragay Gulf, Luzon, 24 Nov. 1966; CAS 34272 (male, 108.0), Nagas Point, Ragay Gulf, Luzon, 543–547 m, 14 Nov. 1966; CAS 33067 (female, 125.2), south of Barrio Salong, Luzon Island, 208–219 m, 18 Jul. 1966; CAS 46966 (4), Sandoval Point, Catanuan, Luzon, 143 m, 3 Nov. 1966.

**Other catalog number.**BMNH 1879.5.14.567 (NHMUK:ecatalogue:3108899).

**(758) *Callionymushildae* Fricke, 1981: 157, figs 8–9**

**Holotype.**CAS-SU 27197 (male, 66.0), Manila Bay, Luzon, 11 Apr. 1931.

**Paratypes.**CAS-SU 68768 (6 males, 51.9–60.2 + 1 female, 49.9), same data as holotype; BMNH 1933.3.1.697–698 (2 males, 55.7–61.8), Manila, Luzon, 1 Mar. 1933; CAS-SU 20671 (2 females, 33.2–37.8), same locality as BMNH 1933.3.1.697–698; CAS 32869 (1 male, 51.9 + 1 female, 42.5) Manila Bay, Luzon, 18–28 m, 9 Apr. 1964; CAS 46978 (male, 50.0), same locality as CAS 32869, 8 Dec. 1953.

**Remarks.** The catalog number BMNH 1933.3.1.697 is occupied by other specimens in NMH’s record. A photograph and a radiograph of the holotype are available in the CAS record.

**(759) *Callionymushudsoni* Fowler, 1941: 8, fig. 5**

= *Callionymusenneactis* Bleeker, 1879.

**Holotype.**USNM 99412 (35.0), Pandanon Island, between Cebu and Bohol islands, 2 m, 23 Mar. 1909.

**Paratypes.** FMNH 48003 (1) and USNM 99414 (1, 38.0), Mantacao Island, Bohol, 8 Apr 1908; USNM 99413 (1, 44.0), Reef opposite Cebu Island, 7 Apr. 1908; USNM 99415 (2, 40.0), Cabugao Bay, Catanduanes Island, Luzon, 9 Jun. 1909; USNM 99416 (1, 29.0), Port San Vicente, Cagayan, off north Luzon, 2 m, 18 Nov. 1908; USNM 99417 (1, 50.0), Cebu market, Cebu Island, 20 Mar. 1909.

**Remarks.**USNM 99414 was not found during the 1980 inventory.

**(760) *Callionymusinversicoloratus* Seale, 1910: 538**

= *Anaoratentaculata* Gray, 1835.

**Holotype.** BSMP 3748 (60.0), Samal Island, Davao Gulf, Mindanao.

**Paratypes.** BSMP (10).

**Remarks.** The original description did not indicate the collection date. All type specimens are presumed destroyed.

**(761) *Callionymuskeeleyi* Fowler, 1941: 14, fig. 9**

**Holotype.**USNM 99425 (80.0), Cebu Island, 17 Apr. 1929.

**(762) *Callionymusleucobranchialis* Fowler, 1941: 19, figs 12–13**

**Holotype.**USNM 99393 (99.0), Albatross station 5442, San Fernando Point Lighthouse, west of Luzon, 83 m, 11 May 1909.

**Paratypes.**USNM 99399 (6, 43.0–97.0), same data as holotype; USNM 99395 (4, 38.0–59.0) and USNM 99400 (1, 60.0), Bacoor, Cavite, Luzon, 15 Jun. 1908; SU 40197 [ex USNM 99401] (2), Manila Bay, Luzon, 12 Dec. 1908.

**Remarks.**Böhlke (1953) and Fricke (1982) referred to some types and other specimens as different species. Some paratypes of this species (SU 40197) were identified as *Callionymushildae* and *C.keeleyi* by Fricke (1982, 2002b).

**(763) *Callionymuslongi* Fowler, 1941: 10, fig. 6**

= *Callionymusfilamentosus* Valenciennes, 1837.

**Holotype.**USNM 99418 (98.0), Albatross station 5152, off Pajumajan Island, Tawi Tawi Group, Sulu Archipelago, 62 m, 18 Feb. 1908.

**Remarks.** Radiographs of the holotype are available in the USNM record.

**(764) *Callionymusmarisinensis* Fowler, 1941: 7**

**Holotype.**USNM 99410. China Sea, vicinity of Hong Kong.

**Paratype.**USNM 99411 (1, 36.0), Albatross station 5157, north of Tinakta Island, Sulu Archipelago, 62 m, 21 Feb. 1908.

**Remarks.** Questionably a synonym of *Callionymusrecurvispinnis* (Li 1966) (Fricke 2002b). Further study is needed to verify its status.

**(765) *Callionymusocellatus* Pallas, 1770: 25, pl. 4 (figs 1–3)**

= *Synchiropusocellatus* (Pallas, 1770).

**Neotype.** SMNS 2163 (male, 72.8), Santa Rosa, Lapu-Lapu, Cebu Island, Mar. 1980.

**Remarks.** A neotype was designated by Fricke (2000), which erroneously indicated 10°19.00'S, 123°57.00'E as the geographical location instead of 10°19.00'N, 123°57.00'E.

**(766) *Callionymusoctostigmatus* Fricke, 1981: 143, figs 1–6**

**Holotype.**CAS 33370 (male, 113.9), Sanderal Point, Catanauan, Luzon, 128–143 m, 3 Nov. 1966.

**Paratypes.**CAS 47766 (3 males, 95.3–102.0), same data as holotype; CAS 32620 (4 males, 70.9–90.1 + 7 females, 56.2–92.4), 5 miles west of Nasugbu Bay, Luzon, 58–103 m, 17 Jun. 1966; CAS 32828 (1 male, 65.5 + 7 females, 55.9–61.0), Nasugbu Bay, Luzon Island, 55–64 m, 18 Jun. 1966; CAS 47768 (male, 94.2), 3 miles south of Fortune Island, Nasugbu Bay, Luzon, 128–178 m, 17 Jun. 1966; CAS 32727 (female, 64.4), CAS 46530 (2 females, 50.3–61.0) and CAS 32747 (5 males, 42.5–50.3 + 2 females, 37.6–38.7), west of Corregidor Island, Bataan, Luzon, 51–66 m, 31 May 1966; CAS 45896 (2 males, 53.5–55.2), near Corregidor, Manila Bay, Luzon, 25 May 1948; CAS 32775 (male, 34.2), 7 miles west of Talaga, Bataan, Luzon, 64–90 m, 1 Jun. 1966; CAS 46531 (1 male, 31.2 mm + 1 female, 30.0), 2.5 miles west of Calaguaguin Cove, Zambales, Luzon, 64–81 m, 9 Jun. 1966; CAS 47780 (female, 86.7), Rogo Point, Ragay Gulf, Luzon, 110–113 m, 1 Nov. 1966; CAS 47781 (female, 89.3), Siburio Point, Ragay Gulf, Luzon, 148–161 m, 6 Nov. 1966.

**(767) Callionymus (Calliurichthys) platycephalus Fricke, 1983: 423, fig. 125**

= *Callionymusplatycephalus* Fricke, 1983.

**Holotype.**USNM 231409 (male, 79.8), east of Sicogon Island, between Negros and Masbate islands, 0–47.6 m, 4 Jun. 1978.

**Paratypes.**USNM 231410 (11 males, 69.8–86.3 + 19 females, 62.3–82.3), same data as holotype.

**(768) *Callionymuspunctilateralis* Fowler, 1941: 13, fig. 8**

= *Callionymusfilamentosus* Valenciennes, 1837.

**Holotype.**USNM 99422 (male, 162.0), Tigbauan, Iloilo, Panay Island, 14 May 1929.

**Paratypes.**USNM 99423 and USNM 99424 (2 males + 2 females, 52.0–84.0), below the mouth of Mindanao River, Cotabato, Mindanao, 20 May 1908.

**(769) *Callionymusscabriceps* Fowler, 1941: 4, fig. 2**

**Holotype.**USNM 99406 (58.0), Jolo Island, Sulu Archipelago, 26 m, 8 Feb. 1908.

**Paratypes.**USNM 99407 (38.0), Surigao, east of Mindanao, 2–9 m, 8 May 1908.

**(770) *Callionymussplendidus* Herre, 1927a: 416, pl. 2**

= *Synchiropussplendidus* (Herre, 1927).

**Holotype.** BSMP (45.0), Bongao, Tawi Tawi, Sulu Archipelago, 3.7 m.

**Remarks.** The original description did not indicate the collection date. The original description misspelled the genus as *Gallionymus* but was corrected as *Callionymus* on a figure. The type specimen was presumed destroyed.

**(771) *Callionymusumbrithorax* Fowler, 1941: 3, fig. 1**

**Holotype.**USNM 99433 (male, 47.0) Albatross station 5345, Cliff Island, Malampaya Sound, Taytay, Palawan Island, 13 m, 26 Dec. 1908.

**Paratype.**USNM 99434 (1 female, 38.0), same data as holotype.

**(772) *Callionymuszaspilus* Herre, 1933: 24**

= *Synchiropuspicturatus* (Peters, 1877).

**Holotype.**CAS-SU 25515 (28.0), Sitankai Island, Sulu Archipelago, 7 Aug. 1931.

**Remarks.** A photograph and a radiograph of the holotype are available in the CAS record.

**(773) *Calliurichthyslinea-thorax* Fowler, 1943: 80, fig. 19**

= *Callionymusneptunius* (Seale, 1910).

**Holotype.**USNM 99504 (115.0), Albatross station 5174, off Jolo Lighthouse, Sulu Archipelago, 37 m, 5 Mar. 1908.

**(774) *Calliurichthysneptunia* Seale, 1910: 539**

= *Callionymusneptunius* (Seale, 1910).

**Holotype.** BSMP 2317 (190.0), Balayan Bay, Luzon, 20 Jan. 1908.

**Paratype.** Uncat. (1, 160.0), same locality as the holotype.

**Remarks.** The original description provided no catalog number for the paratype. All type specimens were presumed destroyed.

**(775) *Eleutherochirmccaddeni* Fowler, 1941: 27, fig. 16**

= *Eleutherochiropercularis* (Valenciennes, 1837).

**Holotype.**USNM 99435 (52.0), Hinunangan Bay, between Samar and Leyte islands, 30 Jul. 1909.

**(776) Synchiropus (Synchiropus) bartelsi Fricke, 1981: 103, fig. 32**

**Holotype.**USNM 225711 (male, 32.5), 09°08.05'N, 123°29.37'E, south of San Juan, Siquijor Island, 0–6 m, 9 May 1978.

**Paratypes.**USNM 225712 (2 females, 17.0–21.9), USNM 225713 (female, 19.4), off Bonbonon Pt., southern tip of Negros Island, 0–12.2 m, 13 May 1978; USNM 227214 (2 females, 15.3–19.7), northeast side of Bararin Island, Cuyo, Palawan, 0–17.4 m, 24 May 1978.

**(777) *Synchiropusdelandi* Fowler, 1943: 81, fig. 20**

**Holotype.**USNM 99524. Mabul Island, Sibuko Bay, Borneo.

**Paratypes.**USNM 99525 (1, 148.0) and USNM 99526 (1, 170.0), Albatross stations 5516-18, Tagolo Point Lighthouse, northern Mindanao, 320–366 m, 9 Aug. 1909; USNM 99527 (1, 130.0), Albatross station 5273, Corregidor Lighthouse, southern Luzon, 208 m, 14 Jul. 1908.

**(778) *Synchiropusgrinnelli* Fowler, 1941: 24, fig. 15**

**Holotype.**USNM 99436 (118.0), Albatross station 5475 (12°55.43'N, 124°22.20'E), San Bernardino Lighthouse, east of Luzon, 357 m, 24 Jun. 1909.

**(779) *Synchiropuspallidus* Fowler, 1941: 23, fig. 14**

= *Synchiropusaltivelis* (Temminck & Schlegel, 1845).

**Holotype.**USNM 99437 (190.0), Albatross station 5393, Panganalan Point, Talajit Island, between Samar and Masbate islands, 249 m, 13 Mar. 1909.

**(780) *Synchiropussycorax* Tea & Gill, 2016: 86, figs 1–4**

**Holotype.** AMS I.47200-001 (male, 39.4), Pagkaliwagan, Jolo Island, Sulu Archipelago, 29 Nov. 2015.

**Paratypes.** AMS I.47200-002 (female, 26.7), CAS 241566 (male, 34.8), USNM 438956 (male, 35.5) and ZRC 54776 (1 female, 22.6 + 1 male, 40.1), taken with holotype.

**(781) *Synchiropustentaculatus* Herre, 1928: 33, pl. 1**

= *Anaoratentaculata* Gray, 1835.

**Holotype.** BSMP 7302 (male, 34.5), Puerto Galera, Mindoro Island, May 1912.

**Paratype.** BSMP (female, 32.0), same data as holotype.

**Remarks.** Subjectively invalid; secondarily preoccupied by *Anaoratentaculata* Gray, 1835 when both are in *Anaora*, replaced by *Anaorafowleri* Herre, 1953. All type specimens are presumed destroyed.

**(782) *Synchiropuszamboangana* Seale, 1910: 540**

**Holotype.** BSMP 4456 (73.0), Zamboanga, Mindanao, 16 Jun. 1908.

**Paratype.** BSMP 3070 (1).

**Remarks.** All type specimens were presumed destroyed.

##### Family Draconettidae (368)

**(783) *Centrodracoabstractum* Fricke, 2002a: 3, fig. 1**

**Holotype.** AMS I.36455-006 (male, 96.8), 12°54.49'N, 124°23.77'E – 12°57.50'N, 124°21.45'E, San Bernardino Strait, east of Luzon, 376–382 m, 23 Sep. 1995.

**Paratypes.** AMS I.36455-006 (2: 1 female, 89.7), same data as holotype.

##### Family Mullidae (403)

**(784) *Mullusmanilensis* Marion de Procé, 1822: 133**

Manila Bay, Luzon.

**Remarks.** No type known. The collection date was not indicated in the original description. An overlooked available name. Nomen dubium. Unknown status in Fricke et al. (2024). Further study is needed to verify its status.

**(785) *Upeneoidesbelaque* Fowler, 1918: 40, fig. 16**

= *Upeneussulphureus* Cuvier, 1829.

**Holotype.**ANSP 47512 (120.0), Philippines.

**Paratypes.**ANSP 47513 [ex 47513–17] (5, 69.0–140.0), same data as holotype.

**Remarks.** All type specimens are in the same jar. The original description or ANSP record did not provide a specific locality and collection dates.

**(786) *Upeneoidesphilippinus* Fowler, 1918: 37, fig. 15**

= *Upeneusvittatus* (Forsskål, 1775).

**Holotype.**ANSP 47508 (180.0), Philippines.

**Paratypes.**ANSP 47509 [ex 47509–11] (3, 120.0–149.0), same data as holotype.

**Remarks.** The original description or ANSP did not provide a specific locality and collection dates.

**(787) *Upeneusasymmetricus* Lachner, 1954: 511, pl. 13 (fig. B)**

**Holotype.**USNM 154659 [ex USNM 145240] (female, 76.0), Pandanon Island, between Cebu and Bohol islands, 24 Mar. 1909.

**Paratypes.**USNM 154660 (2, 73.0–81.0), same data as holotype; USNM 154661 (1, 101.0), Catbalogan, off Western Samar Island, 3 m, 15 Apr. 1908.

**Remarks.** Radiographs and photographs of the holotype are available in the USNM record.

**(788) *Upeneusluzonius* Jordan & Seale, 1907: 25, fig. 9**

**Holotype.**CAS-SU 9244 (120.7), Cavite, Luzon, 1 Jun. 1900.

**Paratypes.**CAS-SU 20101 (2), same data as holotype; USNM 53067 (2), Manila, Luzon.

**Remarks.** The original description mentioned seven specimens. The original description and Böhlke (1953) mentioned USNM 53067 as the holotype. The USNM record indicated it as “types” with 2 specimens and found in 1922. A photograph and a radiograph of the holotype are available in the CAS record.

**(789) *Upeneusnigromarginatus* Bos, 2014: 753, figs 3–5**

**Holotype.** RMNH. PISC 37991 (196.0), 07°18.38'N, 125°41.02'E, Panabo Fish Market, Davao, Mindanao, 26 Jun. 2012.

**Paratypes.** RMNH.PISC 36422 (1, 151.0), RMNH.PISC 36423 (1, 155.0), RMNH.PISC 36424 (1, 156.0) and RMNH.PISC 37992 (1, 155.0), same data as holotype.

**(790) *Upeneusstenopsis* Uiblein & McGrouther, 2012: 63, fig. 1, 2**

**Holotype.** AMS I.20918-017. Coral Sea, off Raine Island, Queensland, Australia.

**Paratypes.**MNHN 2012-0212 (1, 112.0) and MNHN 1984-0802 (1, 101.0), Musorstom 1 station 35 (13°58.98'N, 120°18.00'E), off Quezon Island, west of Luzon, 186–187 m, 23 Mar. 1976.

**Remarks.** These type specimens are on-loan based on MNHN record.

#### ﻿﻿ORDER SCOMBRIFORMES (75)

##### Family Trichiuridae (371)

**(791) *Benthodesmusbenjamini* Fowler, 1938: 45, fig. 16**

= *Benthodesmustenuis* (Günther, 1877).

**Holotype.**USNM 98821 (632.0), Albatross station 5445 (12°4.70'N, 124°59.83'E), Atalaya Point, Batag Island, east of Luzon, 700 m, 3 Jun. 1909.

**Paratypes.**USNM 98823 (1), same locality as the holotype; USNM 98825 (1), Albatross station 5575, Mt. Dromedario, Simaluc Island, Sulu Archipelago, 576 m, 23 Sep. 1909.

**(792) *Benthodesmussuluensis* Parin, 1976: 191**

**Holotype.** ZIN 42638, 08°37.00'N, 119°46.00'E, near Tubbataha Reefs Natural Park, Sulu Sea, Palawan, 200 m.

**Remarks.** The original description did not indicate the collection date.

**(793) *Benthodesmustuckeri* Parin & Becker, 1970: 359, fig. 2**

**Holotype.**USNM 98822 (female, 598.0), Albatross station 5111, Sombrero Island, Balayan Bay and Verde Island Passage, Luzon, 432 m, 16 Jan. 1908.

**Paratypes.**USNM 98824 (1, 614.0), Albatross station 5444, Atalaya Point, Batag Island, northern Samar, 563 m, 3 Jun. 1909; ZIN 39126 (1).

**Remarks.** The original description mentioned that the holotype was collected in Balayan Bay, Luzon at a depth of 554 m. However, the USNM record stated that Parin and Becker (1970) made a mistake in recording Albatross station 5444 as the type locality as this corresponds to that of USNM 98824 which was also examined by Parin and Becker.

##### Family Scombridae (372)

**(794) *Nesogrammuspiersoni* Evermann & Seale, 1907: 61, fig. 3**

= *Grammatorcynusbilineatus* (Rüppell, 1836).

**Holotype.**USNM 55899 (406.4), Bulan, Sorsogon, Luzon.

**Remarks.** The original description or USNM record did not indicate the collection date.

**(795) *Rastrelligerfaughni* Matsui, 1967: 74, figs 1, 5**

**Holotype.**USNM 190018 (male, 188.0), Oyster Inlet, Ulugan Bay, Puerto Princesa, Palawan Island, 28 Dec. 1908.

**Paratypes.**USNM 201340 (6, 172.0–187.0) [ex USNM 190018], same data as holotype; USNM 190019 (1, 182.0), Nasugbu Bay, Luzon, 2–5 m, 16 Jan. 1908; USNM 199938 (11, 193.0–204.0), Manila, Luzon; USNM 199940 [ex USNM 190021] (3, 92.0–113.0), Atulayan Bay, east of Luzon; SIO 66-271 (7, 171.0–191.0) and SIO 65-420 (1, 184.0), Manila market, Luzon.

**Remarks.** The holotype was previously mixed with six other specimens but was re-cataloged to USNM 201340. Radiographs of the holotype are available in the USNM record.

##### Family Ariommatidae (376)

**(796) *Psenesextraneus* Herre, 1951: 341**

= *Ariommaindica* (Day, 1871).

**Holotype.**USNM 202517 [ex UW 7167], Batangas Bay, Luzon, 22 Apr. 1948.

**Remarks.** Specimen was purchased in Batangas fish market. The record was based on the USNM record.

##### Family Chiasmodontidae (379)

**(797) *Pseudoscopeluscephalus* Fowler, 1934: 361, fig. 111**

**Holotype.**USNM 93142 (89.0 TL), Albatross station 5423 (09°38.50'N, 121°11.00'E), Cagayancillo, Palawan, 929 m, 31 Mar. 1909.

##### Family Bramidae (427)

**(798) *Bramaleucotaenia* Fowler, 1938: 44, fig. 15**

= *Bramadussumieri* Cuvier, 1831.

**Holotype.**USNM 98817 (32.0), Albatross station 5134 (06°44.75'N, 121°48.00'E), off Baluk baluk Island, near Basilan Island, Sulu Archipelago, 46 m, 7 Feb. 1908.

#### ﻿﻿ORDER LABRIFORMES

##### Family Labridae (390)

**(799) *Bodianusdictynna* Gomon, 2006: 59, figs 1c, 5d, 38, pls 5J, 6A, B**

**Holotype.**USNM 217870. Maru, Guadalcanal, Solomon Islands.

**Paratypes.**USNM 336613 (2, 26.0–73.2), off Bonbonon Point at southern tip of Negros Island, 0–18 m, 13 May 1978; NMV A15673 (1, 46.1), Liloan Point (Whirlpool Point), southern tip of Cebu Island, 13.4–19.2 m, 29 Apr. 1979; ROM 52964 (4, 40.4–63.8), mouth of Bais Bay, Tañon Strait, Negros, 15 May 1987.

**(800) *Callyodonalbipunctatus* Seale, 1910: 526**

= *Chlorurusspilurus* (Valenciennes, 1840).

**Holotype.** BSMP 4876 (170.0), Sitanki Island, Sulu Archipelago, 18 Jul. 1908.

**Remarks.** The type specimen was presumed destroyed.

**(801) *Callyodonbleekeri* de Beaufort, 1940: 318**

= *Chlorurusbleekeri* (de Beaufort, 1940).

**Syntype.** Philippines.

**Remarks.** The original description did not provide further information.

**(802) *Callyodonelerae* Jordan & Seale, 1907: 31, fig. 11**

= *Scarustricolor* Bleeker, 1847.

**Holotype.**CAS-SU 9246 (317.5), Cavite, Luzon, 1 Jun. 1900.

**Remarks.** A photograph and a radiograph of the holotype are available in the CAS record.

**(803) *Callyodonhadji* Seale, 1910: 525**

= *Scarusquoyi* Valenciennes, 1840.

**Holotype.** BSMP 5367 (225.0), Puerto Princesa, Palawan Island, 19 Aug. 1909.

**Paratype.** BSMP 5494 (1), same locality as holotype.

**Remarks.** All type specimens were presumed destroyed.

**(804) *Callyodonlatifasciatus* Seale & Bean, 1907: 237, fig. 7**

= *Scarustricolor* Bleeker, 1847.

**Holotype.**USNM 57845 (285.8), Zamboanga, Mindanao.

**Paratype.**USNM 61152 (1, 260.4), same locality as holotype.

**Remarks.** The original description or USNM record did not indicate the collection date of the holotype. Radiographs of the holotype specimens are available in the USNM record.

**(805) *Callyodonlineolabiatus* Fowler & Bean, 1928: 457, pl. 47**

= *Scarusniger* Forsskål, 1775.

**Holotype.**USNM 89976 [or ANSP 101792] (183.0), Butauanan Island, Camarines Sur, 12 Jun. 1909.

**Remarks.**Böhlke (1984) listed ANSP 101792 [ex 19185] as the holotype, not USNM 89976. However, the USNM record indicated that 89976 is also the holotype of *C.lineolatus* but listed as missing. Since the specimen of USNM 89976 is missing, it may be within the possession of ANSP.

**(806) *Callyodonogos* Seale, 1910: 527**

= *Scarushypselopterus* Bleeker, 1853.

**Holotype.** BSMP 5414 (225.0), Puerto Princesa, Palawan Island, 20 Aug. 1908.

**Paratype.** BSMP 5411 (1), same locality as holotype.

**Remarks.** All type specimens were presumed destroyed.

**(807) *Callyodonphilippinus* Fowler, 1918: 66, fig. 26**

= *Chlorurusjapanensis* (Bloch, 1789).

**Holotype.**ANSP 47548 (215.0), Philippines.

**Remarks.** The original description or ANSP did not provide a specific locality and collection date. Identification notes such as *Scarusblochi* and *Scarusquoyi* are found inside the lot.

**(808) *Callyodonrostratus* Seale, 1910: 524**

= *Chlorurusspilurus* (Valenciennes, 1840).

**Holotype.** BSMP 2928 (215.0), Zamboanga, Mindanao, 10 Apr. 1908.

**Remarks.** The type specimen was lost (Kottelat 2013).

**(809) *Callyodonvermiculatus* Fowler & Bean, 1928: 472, pl. 49**

= *Scarusfrenatus* Lacepède, 1802.

**Holotype.**USNM 89978. Tidore Island, Molucca Islands, Indonesia.

**Paratypes.**USNM 147402 (1), Tonguil Island, Tambun, Sigambul, southern Zamboanga, Mindanao, 14 Sep. 1909; USNM 157268 (1, 210.0), Cabugan Grande Island, Hinunangan Bay, between Samar and Leyte islands, 3–5 m, 30 Jul. 1909; USNM 160141 (1, 130.0), Bugsuk Island, Balabac, Palawan, 3–5 m, 5 Jan. 1909.

**Remarks.** A radiograph of the holotype is available in the USNM record.

**(810) *Callyodonviridibusius* Fowler & Bean, 1928: 459, pl. 48**

= *Scarustricolor* Bleeker, 1847.

**Holotype.**USNM 89977. Uki Island, Moluccas Islands, Indonesia.

**Paratypes.**USNM 157068 (1, 225.0), Jolo Market, Sulu Archipelago, 6 Mar 1908; USNM 157326 (1, 196.0), Solino Island, northern Mindanao, 3–6 m, 10 Aug 1909.

**Remarks.** The original description mentioned the Iloilo market as the locality of USNM 157068 taken on 1 Jun. 1908. However, USNM record indicated Jolo Market in the Sulu Archipelago as the locality and taken on 6 Mar. 1908. A radiograph of the holotype is available in the USNM record.

**(811) *Cheilinusrostratus* Cartier, 1874: 103**

= *Cheilinusundulatus* Rüppell, 1835.

**Syntypes.** Uncat. (2, 69.0–88.0), Cebu Island.

**Remarks.** The original description did not provide catalog numbers or collection date. All type specimens have unknown location.

**(812) *Choerodonbalerensis* Herre, 1950: 149**

= *Choerodonfasciatus* (Günther, 1867).

**Holotype.**USNM 202508 [ex UW 7990], Baler, Quezon, Luzon, Jul. 1947.

**(813) *Choerodonmargaritiferus* Fowler & Bean, 1928: 197**

**Holotype.**USNM 89966 (135,0), Jolo Market, Sulu Archipelago, 11 Feb. 1908.

**Remarks.** A specimen (USNM 135558) was found on 11 Feb. 1977 with a linen tag (5577) same as USNM 89966 and is thought to be the holotype of this species (Smithsonian Museum Archive 2024).

**(814) *Choerodonmelanostigma* Fowler & Bean, 1928: 199, pl. 16**

= *Choerodonzamboangae* (Seale & Bean, 1907).

**Holotype.**USNM 89967 (208.0), Jolo Market, Sulu Archipelago, 12 Feb. 1908.

**Paratypes.**USNM 93527 (2, 120.0–204.0), same data as holotype.

**(815) *Choeropsmaeander* Cartier, 1874: 102**

= *Choerodonanchorago* (Bloch, 1791).

**Syntypes.** Uncat. (6, 39.0–67.0), Cebu Island.

**Remarks.** The original description did not provide catalog numbers and collection date. The type specimens cannot be located (Gomon 2017).

**(816) *Choeropspalawanensis* Seale, 1910: 523**

= *Choerodonoligacanthus* (Bleeker, 1851).

**Holotype.** BSMP 5501 (235.0), Puerto Princesa, Palawan Island, 22 Aug. 1907.

**Remarks.** The type specimen was presumed destroyed.

**(817) *Choeropsunimaculatus* Cartier, 1874: 102**

= *Choerodonschoenleinii* (Valenciennes, 1839).

**Syntypes.** Uncat. (2, 51.0–57.0), Cavite, Luzon.

**Remarks.** The original description did not provide catalog numbers and collection date. Type specimens appear to be lost (Fricke et al. 2024).

**(818) *Choeropszamboangae* Seale & Bean, 1907: 236**

= *Choerodonzamboangae* (Seale & Bean, 1907).

**Holotype.**USNM 57846 (285.8), Zamboanga, Mindanao.

**Paratype.**USNM 61154 (1, 260.4), same locality as holotype.

**Remarks.** The original description or USNM record did not indicate the collection date of the holotype.

**(819) *Cirrhilabrusbriangreenei* Tea, Pyle & Rocha, 2020: 93, figs 1–4A, B, 5A**

**Holotype.**PNM 15518 [ex CAS 238389] (male, 54.9), 13°39.65'N, 120°49.88'E, Maricaban Island, off Batangas Bay, Luzon, 10 Dec. 2013.

**Paratypes.** AMS I. 49028-001 [ex CAS 238389] (male, 58.2) and CAS 238389 (female, 49.6), taken with holotype; BPBM 41374 [ex CAS 238392] (male, 65.7) and CAS 238392 (female, 59.8), same locality with holotype, 8 Dec. 2013; CAS 242284 (male, 73.6) and CAS 242289 (female, 56.5), Verde Island, Batangas, Luzon, 82–110 m, 12–13 Apr. 2015.

**(820) *Cirrhilabruscyanogularis* Tea, Frable & Gill, 2018: 578, figs 1–6, 9A, 10A**

**Holotype.**PNM 15360 [ex PNM 15354] (male, 44.5), 06°0.0'N, 121°52.0'E, Banguingui Island Sulu Archipelago, 30 m, 17 Jul. 2016.

**Paratypes.** AMS I.47440-001 (male, 44.5) and ZRC 56451 (male, 44.1), SIO 17-27 (male, 54.8), same data as holotype.

**Remarks.** The holotype registration number was corrected in erratum (Tea et al. 2018).

**(821) *Cirrhilabrusisosceles* Tea, Senou & Greene, 2016: 21, figs 1–4**

**Holotype.** KPM-NI 5681. Funauki Bay, Yaeyama Island, Ryukyu Islands, Japan.

**Paratypes.** AMS I.47150-001 (male, 55.3), ZRC 54775 (male, 55.1), USNM 437595 (male, 52.7) and BMNH 2016.5.27.1 (male, 55.7) Fuga Island, Cagayan, northern Luzon, 24–36 m, 20 Nov. 2015.

**Other catalog number.**BMNH 2016.5.27.1 (NHMUK:ecatalogue:6635945).

**(822) *Cirrhilabruslubbocki* Randall & Carpenter, 1980: 18, figs 1A, 2–6**

**Holotype.** BPBM 18458 [not 18485], off marine station of the University of San Carlos, east of Mactan Island, Cebu, 8 m, 26 Jun. 1975.

**Paratypes.** BPBM 18470 (9), same data as holotype; AMS I.20700-001 (1), ANSP 140168 (1) and BMNH 1979.1.3.2 (1), Philippines (from fish collectors); BPBM 22069 (1), WAM P.25517-001 (1, 56.0) and WAM P.25517 002 (1, 45.0), Mactan Island, Cebu, 38 m, 21 Aug 1977; BPBM 22473 (28), CAS 42525 (1), MNHN 1978-0769 (1) and USNM 219336 (1), Caban Island, Batangas, Luzon, 30 m, 28 Jul. 1978; USNM 219335 (1), Mactan Island, Cabu, 0–40 m, 2 Jun. 1978; USNM 219365 (69), Pamilacan Island, Bohol, 0–33.5 m, 12 Jun. 1978; ZUMT 54168 (1).

**Other catalog number.**BMNH 1979.1.3.2 (NHMUK:ecatalogue:2545084).

**(823) *Cirrhilabrusrubripinnis* Randall & Carpenter, 1980: 23, figs 9, 10**

**Holotype.** BPBM 22515 (male, 59.9), 13°41.0'N 120°50.0'E, Caban Island, Verde Island Passage, SW Luzon, 30 m, 28 Jul. 1978.

**Paratypes.** AMS I.20699-001 (1), ANSP 140170 (1), BMNH 1979.1.4.2 (1), BPBM 22470 (16), CAS 42527 (1) and USNM 219338 (1), same data as the holotype; MNHN 1978-0768 (1), off east of Luzon, 30 m, Jul. 1978; ZUMT 54167 (1).

**Other catalog number.**BMNH 1979.1.4.2 (NHMUK:ecatalogue:2545090).

**(824) *Cirrhilabrusshutmani* Tea & Gill, 2017: 78, figs 1–5, 8A**

**Holotype.**PNM 15354 (male, 55.7), Didicas Volcano, Babuyan Island, Cagayan, Luzon, 50–70 m, 25 Aug. 2016.

**Paratypes.** AMS I. 47290-001 (male, 44.2), WAM P.34787-001 (male, 45.2), and ZRC 55885 (male, 39.5), taken with the holotype.

**(825) *Corisphilippina* Fowler & Bean, 1928: 309, pl. 42**

= *Pseudocorisbleekeri* (Hubrecht, 1876).

**Holotype.**USNM 89975 (103.0), Sulade Island, Jolo, Sulu Archipelago, 3–5 m, 17 Sep. 1909.

**Paratype.**USNM 93519 (1, 108.0), same data as the holotype.

**Remarks.** The original description mentioned USNM 93519 measuring 108.0 from Balicasag Island on 11 Aug. 1909. However, the USNM record indicated it was collected with the holotype. The paratype was re-identified as *Pseudocorisyamashiroi* by Smith-Vaniz (Smithsonian Museum Archive 2024).

**(826) *Duymaeriaguttata* Fowler & Bean, 1928: 218**

= *Pteragogusguttatus* (Fowler & Bean, 1928).

**Holotype.**USNM 89969 (72.0), Catbalogan, off western Samar Island, 16 Apr. 1908.

**Paratypes.**USNM 93625 (1, 69.0), Cebu Market, Cebu Island, 4 Apr 1908; USNM 93626 (1, 56.0), Port Uson, west of Pinas Island, Busuanga, Palawan, 17 Dec. 1908.

**(827) *Halichoereschrysus* Randall, 1981: 416, figs 1, 2**

**Holotype.** BPBM 16151. Sandfly Passage, Florida Island, Solomon Islands.

**Paratypes.**ANSP 144094 [ex BPBM 9889] (2, 44.0–53.8) and WAM P.26850-001 (1, 47.2), Philippine Islands (via Modern Pet Shop, Honolulu), 1970; BPBM 8671 (1, 53.5), same locality as preceding, Apr. 1969.

**Remarks.** The original description or ANSP record did not provide a specific locality for ANSP paratypes.

**(828) *Halichoerescymatogrammus* Jordan & Seale, 1905: 786, fig. 8**

= *Halichoeresscapularis* (Bennett 1832).

**Holotype.**USNM 51947 (38.1), south of Negros Island, 1901.

**(829) *Halichoeresdesmogenys* Fowler & Bean, 1928: 276, pls 28, 29**

= *Halichoeresvrolikii* (Bleeker, 1855).

**Holotype.**USNM 89973 (113.0), Anchorage, Dupon Bay, Leyte Island, 17 Mar. 1909.

**Paratype.**USNM 93522 (1, 112.0), Macalajar Bay, northern Mindanao, 2–4 m, 8 Aug. 1909.

**(830) *Halichoereshilomeni* Randall & Allen, 2010: 286, figs 8–12**

**Holotype.** UPLB 498 (male, 95.6), 11°04.32'N, 119°22.30'E, Dibuluan Island, Bacuit Bay, El Nido, Palawan, 1–3 m, 12 Jun. 2008.

**Paratypes.** BPBM 41024 (2, 55.0–91.5), UPLB 495 (1, 75.5), UPLB 500 (1, 45.6), USNM 398628 (2, 73.5–81.3), WAM P.33270-001 (2, 57.7–77.9) and ZRC 52402 (1, 59.4), taken with the holotype.

**(831) *Halichoeresiris* Seale, 1910: 522**

= *Thalassomahardwicke* (Bennett, 1830).

**Holotype.** BSMP 4582 (112.0), Sitanki Island, Sulu Archipelago, 2 Jul. 1908.

**Remarks.** The type specimen was presumed destroyed.

**(832) *Halichoeresleucostigma* Fowler & Bean, 1928: 299, pl. 40**

= *Halichoeresnigrescens* (Bloch & Schneider, 1801).

**Holotype.**USNM 89974 (130.0), Catbalogan, off west of Samar Island, 1–6 m, 15 Apr. 1908.

**Remarks.** The original description mentioned Pujada Bay in Mindanao as the type locality and a collection date of 15 May 1908. The linen tag 13805 still attached to the specimen matches different data (15 Apr 1908, Catbalogan, Samar) (Smithsonian Museum Archive 2024).

**(833) *Halichoeresmelanochir* Fowler & Bean, 1928: 264, pl. 25**

**Holotype.**USNM 89972 (126.0), Lampinigan Island, south of Zamboanga, Mindanao, 2–5 m, 11 Sep. 1909.

**Paratypes.**USNM 93523 (1, 115.0), Albatross station 5172, Jolo Lighthouse, Sulu Archipelago, 582 m, 5 Mar. 1908; USNM 93524 (1, 110.0), Maculabo Island, east of Luzon, 4 Jun. 1909.

**Remarks.** A radiograph of the holotype is available in the USNM record.

**(834) *Halichoeresmelasmapomus* Randall, 1981: 421, figs 3, 4**

**Holotype.** BPBM 16899. Off Gannet Ridge, Pitcairn Island.

**Paratype.** BPBM 18466 (1, 98.9), off marine station of the University of San Carlos, Mactan Island, Cebu, 35 m, 26 Jun. 1975; BPBM 22070 (1, 80.2), same locality as preceding, 38 m, 21 Aug. 1977; USNM 219866 (1, 72.0), SP 78-43, Pamilican Island, Bohol, 0–24 m, 12 Jun. 1978.

**(835) *Halichoeresrichmondi* Fowler & Bean, 1928: 263, pl. 24**

**Holotype.**USNM 89971 (158.0), Inamucan Bay, northern Mindanao, 2–5 m, 8 Aug. 1909.

**Paratype.**USNM 93525 (1, 152.0), Mahinog, Camiguin Island, between Leyte and Mindanao islands, 4–6 m, 3 Aug. 1909.

**(836) *Hemipteronotusnigromaculatus* Herre, 1933: 20**

= *Iniistiustwistii* (Bleeker, 1856).

**Holotype.**CAS-SU 25513 (155.0), Jolo Island, Sulu Archipelago, 2 Aug. 1931.

**Remarks.** A photograph and a radiograph of the holotype are available in the CAS record.

**(837) *Hipposcarusschultzi* Smith, 1959: 277, fig. 8**

= *Hipposcaruslongiceps* (Valenciennes, 1840).

**Holotype.** SAIAB [ex RUSI] 88 [ex USNM 157089] (370.0), Murcielagos Bay, Mindanao, 2–8 m, 9 Aug. 1909.

**Paratypes.** SAIAB [ex RUSI] 611 (1) and USNM 112218 (1), Maculabo Island, east of Luzon, 2–5 m, 14 Jun. 1909.

**Remarks.** The original description mentioned the type specimen (holotype) a catalog number of USNM 9270 but the USNM record indicates it belongs to *Sphyrnatiburo* from the United States. Several specimens were mentioned in the original description but it was not specified whether these were type specimens. Paratypes were found in museum records.

**(838) *Iniistiusbakunawa* Sorgon, Tea, Meren & Nañola, 2023: 512, figs 1–4**

**Holotype.**PNM 15676 (151.2), purchased from public fish market in Loay, Bohol, 20 Apr. 2018.

**Paratypes.** AMS I.50830-001 (2, 129.6–151.9) and PNM 15711 (129.6), Jolo Public Market, Sulu Archipelago, 3 Aug. 2019; KAUM-I. 80684 (172.0), Tigbauan Fish Market, Iloilo, Panay Island, 10 Nov. 2015; USNM 435404 (162.4), Daanbantayan Fish Market, Cebu Island, 29 Jan. 2015; USNM 437745 (155.1 mm) and USNM 4357747 (158.8 mm), Estancia fish landing site, Iloilo, Panay Island, 16 Jul. 2015.

**Remarks.** All type specimens were collected from public markets or fish landing sites.

**(839) *Julistruncatus* Cartier, 1874: 105**

= *Thalassomalunare* (Linnaeus, 1758).

**Holotype.** Uncat. (6.7), Cebu Island.

**Remarks.** The original description did not provide a catalog number and collection date. Type specimen cannot be located (Fricke et al. 2024).

**(840) *Labroidesbicolor* Fowler & Bean, 1928: 224, pl. 18**

**Holotype.**USNM 89970 (110.0), Port Maricaban, southern Luzon, 4–6 m, 21 Jul. 1908.

**(841) *Labrusbaccatus* Marion de Procé, 1822: 132**

= *Halichoeresnigrescens* (Bloch & Schneider, 1801).

Manila Bay, Luzon.

**Remarks.** No type known.

**(842) *Leptojulislambdastigma* Randall & Ferraris, 1981: 93, fig. 5**

**Holotype.** BPBM 26408, 11°56.2'N, 124°30.8'E, Samar Sea, between Samar and Masbate islands, 80–91 m, 13 Mar. 1980.

**Paratypes.**CAS 47360 (1), same data as holotype; BPBM 26420 (1), Carigara Bay, Leyte Island, 60–65 m, 24 Apr 1980; USNM 222103 (1), southwest of Caduruan Point, between Negros and Masbate islands, 0–75 m, 5 Jun. 1978; USNM 226881 (1), east of Sicogon Island, between Negros and Masbate islands, 0–47.6 m, 4 Jun. 1978; USNM 226882 (1), east of Tanguingui Island, between Negros and Masbate islands, 0–71.4 m, 6 Jun. 1978; MNHN 1981-0607 (1), same locality as holotype, 91 m, Mar. 1980.

**(843) *Leptojulisurostigma* Randall, 1996a: 10, fig. 3; pls 3D–F**

**Holotype.**CAS 32618, 5 miles west of Nasugbu Bay, Luzon, 58.5–100.5 m, 17 Jun. 1966.

**Paratypes.** BPBM 33960 (1), Talesayen Bay, Zambales, Luzon, 13 Jun 1966; CAS 33368 (1), Silanguin Bay, Zambales, Luzon, 33 m, 11 Jun. 1966; USNM 338715 (1), 2 miles west of Corregidor Island, west of Luzon, 57–64 m, 31 May 1966; MNHN 1995-1083 (1), Batan (Bataan), Luzon, 51–60 m, 1 Jun. 1966.

**(844) *Macropharyngodonnegrosensis* Herre, 1932: 142**

**Holotype.**CAS-SU 25519 (94.0), Dumaguete, Negros Island, 20 Jun. 1931.

**Remarks.** A photograph and a radiograph of the holotype are available in the CAS record.

**(845) *Novaculopscompressus* Fukui, 2020: 556, figs 1, 2**

**Holotype.** KAUM-I. 52621 (104.2), Iloilo Central Market, Panay Island, 15 Feb. 2013.

**(846) *Oxycheilinussamurai* Fukui, Muto & Motomura, 2016: [4] 215, figs 1–4**

**Holotype.** KAUM-I 83796. Off Nagura, Ishigaki Island, Ryukyu Islands, Japan.

**Paratypes.** KAUM-I. 514661 (1, 99.1), KAUM-I. 51671 (1, 95.3), UPVMI 2096 (1, 76.9), off Iloilo, Panay Island, 22 Dec. 2012.

**Remarks.** All type specimens were purchased at Iloilo Central Market, Iloilo City.

**(847) *Paracheilinusangulatus* Randall & Lubbock, 1981: (21) 26, pl. 2 (fig. D)**

**Holotype.**BMNH 1979.1.3.3 (male, 51.1), Batangas, Luzon, 1 Aug. 1978.

**Paratype.** BPBM 22526 (1 male, 59.9), taken with holotype.

**Other catalog number.**BMNH 1979.1.3.3 (NHMUK:ecatalogue:2545085).

**Remarks.** All type specimens were bought from aquarium fish collectors.

**(848) *Paracheilinuscarpenteri* Randall & Lubbock, 1981: 20, 24, pl. 2 (figs A, B)**

**Holotype.** BPBM 22424 (male, 39.0), east side of the marine laboratory of University of San Carlos, Mactan Island, Cebu, 40 m, 1 Aug. 1978.

**Paratypes.** BPBM 22116 (1, 52.0), same locality as holotype, 24 Aug. 1977; BPBM 21156 (2, 36.5–36.8), same locality as holotype, 36.5–40 m, 7 Apr. 1978; USNM 219334 (12, 17.6–36.5), same locality as holotype, 2 Jun. 1978; BMNH 1979.1.3.4 (1, 60.7), northwest passage between Cabulang and Vandanon islands, 27 m, 17 Aug. 1976; BPBM 22465 (10, 21.6–65.8), ANSP 140166 (1, 39.0), AMS I. 20697-001 (1, 31.3), CAS 42523 (1, 39.8), MNHN 1978-766 (1, 37.9) and ZUMT 54166 (1, 37.9), Caban Island, Batangas, Luzon, 30 m, 28 Jul. 1978.

**Other catalog number.**BMNH 1979.1.3.4 (NHMUK:ecatalogue:2545086).

**Remarks.** The original description mentioned 28 Jun. 1978 as the collection date for BPBM 22465, ANSP 140166, AMS I. 20697-001, CAS 42523, MNHN 1978-766, and ZUMT 54166 but different from the museum records.

**(849) *Paracheilinuslineopunctatus* Randall & Lubbock, 1981: (20) 21, pl. 1 (fig. B)**

**Holotype.** BPBM 22506 (male, 38.8), east off marine laboratory of the University of San Carlos, Cebu Island, 15 m, 24 Aug. 1977.

**Paratypes.** BPBM 22114 (6, 25.3–48.7), same data as holotype; USNM 219335 (4, 26.5–51.2), Buyong Beach, east of Cebu Island, 34–40 m, 2 Jun. 1978; BPBM 22447 (5, 32.4–38.0), ANSP 140167 (1, 37.0), AMS 1.20696-001 (1, 33.2), BMNH 1979.1.4.3 (1, 37.2), CAS 42524 (1, 39.7), MNHN 1978-767 (1, 36.2) and ZUMT 54165 (1, 30.9), Caban Island, SW of Luzon, 30 m, 28 Jul. 1978; BPBM 22507 (1, 50.2), Sumilon Island, Cebu, 17 m, 18 Sep. 1978.

**Other catalog number.**BMNH 1979.1.4.3 (NHMUK:ecatalogue:2545091).

**(850) *Platyglossusalternans* Cartier, 1874: 104**

= *Halichoeresscapularis* (Bennett, 1832).

**Syntypes.** Uncat. (2, 70.0–83.0), Cebu and Panglao, Bohol.

**Remarks.** The original description did not provide catalog numbers and collection date. The type specimens cannot be located.

**(851) *Platyglossuspseudogramma* Cartier, 1874: 103**

= *Halichoeresbicolor* (Bloch & Schneider, 1801).

**Holotype.** Uncat. (85.0), Ubay, Bohol Island.

**Remarks.** The original description did not provide catalog numbers and collection date. The type specimen cannot be located.

**(852) *Platyglossusreticulatus* Cartier, 1874: 104**

= *Halichoeresargus* (Bloch & Schneider, 1801).

**Syntypes.** Uncat. (2, 67.0–75.0), Cebu Island.

**Remarks.** The original description did not provide catalog numbers and collection date. The type specimens cannot be located.

**(853) *Platyglossusubayensis* Cartier, 1874: 104**

= *Halichoeresvrolikii* (Bleeker, 1855).

**Holotype.** Uncat. (80.0), Ubay, Bohol Island.

**Remarks.** The original description did not provide catalog numbers and collection date. The type specimen cannot be located.

**(854) *Pseudojuloidesmesostigma* Randall & Randall, 1981: 68, figs 11, 12**

**Holotype.** BPBM 22259 (male, 69.2), ~ 600 m south of Layag-Layag Point, east of Caban Island, Batangas, Luzon, 35–39 m, 3 Sep. 1977.

**Paratypes.** BPBM 24781 (2, 55.1–69.8), same data as holotype; BPBM 22445 (1, 47.5) and USNM 222677 (1), same locality but on southwest side, 32 m, 28 Jul. 1978.

**(855) *Pseudoscarusmargaritus* Cartier, 1874: 105**

= *Chlorurusspilurus* (Valenciennes, 1840).

**Holotype.** Uncat. (85.0), Cebu Island.

**Remarks.** The original description did not provide catalog numbers or collection date. The type specimen cannot be located.

**(856) *Scaruschameleon* Choat & Randall, 1986: 199, pls 3 (figs C, D); pl. 9 (figs D–H)**

**Holotype.** BPBM 22177 (male, 204.0), east of Sumilon Island, Cebu, 20 m, 26 Aug. 1977.

**Paratypes.** BPBM 29403 (female, 161.0), same data as holotype; MNHN 1984-418 (male, 149.5) Cebu Market, Cebu Island, 31 Jul. 1978.

**(857) *Scarusflavipectoralis* Schultz, 1958: 52, pl. 9 (fig. D)**

**Holotype.**USNM 112217 (212.0), Pagapas Bay, off west of Luzon, 5 m, 20 Feb. 1909.

**Remarks.** A radiograph of the holotype is available in the USNM record.

**(858) *Scarusophthalmistius* Herre, 1933: 21**

= *Cetoscarusocellatus* (Valenciennes, 1840).

**Holotype.**CAS-SU 25507 (143.0), Jolo Island, Sulu Archipelago, 20 Aug. 1931.

**Paratype.**CAS-SU 69813 [ex CAS-SU 25507] (1, 114.0), same data as holotype.

**Remarks.** The holotype and one paratype were mixed, with the holotype being the larger one (Böhlke 1953). A photograph and a radiograph of the holotype are available in the CAS record.

**(859) *Scarusvisayanus* Herre, 1933: 22**

= *Scarustricolor* Bleeker, 1847.

**Holotype.**CAS-SU 25510 (208.0), Taytay Bay, Palawan Island, 28 Aug. 1931.

**Paratypes.**BMNH 1933.3.11.524 (1, 185.0), taken with holotype; CAS-SU 25511 (1, 208.0), Linapacan Island, Palawan, 1 Oct. 1931; CAS-SU 25512 (1, 222.0), reef between Burias and Ticao islands, Masbate, 15 Aug. 1929.

**Other catalog number.**BMNH 1933.3.11.524 (NHMUK:ecatalogue:2511726).

**Remarks.** The SU registry indicated Jul. 1931 as the collection date, while another label on the bottle provided 1931 for paratype CAS-SU 25512 (Böhlke 1953). The original description or CAS record did not indicate the collection date of the holotype. A photograph and a radiograph of the holotype are available in the CAS record.

**(860) *Stethojulislinearis* Schultz in Schultz et al., 1960: 214, pl. 107 (fig. E)**

= *Stethojulisbandanensis* (Bleeker, 1851).

**Holotype.**USNM 112425. Enyu Island, Bikini Atoll, Marshall Islands.

**Paratypes.**USNM 152227 (12, 73.0–82.0), Cebu Fish Market, Cebu Island, 19–20 Mar. 1909; USNM 152228 (1, 86.0), Sulade Island, Jolo, Sulu archipelago, 3–5 m, 17 Sep. 1909; USNM 152229 (1, 87.0), Teomabel Island, Jolo, Sulu archipelago, 3–8 m, 18 Sep. 1909.

**Remarks.** Based on the USNM record, paratype 152227 specimens should be split into two lots since some specimens were collected in two sampling dates.

**(861) *Stethojuliszatima* Jordan & Seale, 1905: 788, fig. 9**

= *Stethojulisinterrupta* (Bleeker, 1851).

**Holotype.**USNM 51943 (59.7), south of Negros Island, 1901.

**Paratype.**CAS-SU 9134 (1), Negros Island, 1900.

**(862) *Terelabruszonalis* Fukui, 2018: 85, figs 1, 2**

**Holotype.**MNHN 2005-0513 (61.3), Musorstom 3 12°07.98'N, 121°16.98'E, off the south coast of Mindoro Island, 73–84 m, 3 Jun. 1985.

**Paratype.** KAUM-I. 115925 (1, 63.6), same locality as holotype.

**(863) *Wetmorellaalbofasciata* Schultz & Marshall, 1954: 446, pl. 12 (fig. E)**

**Holotype.**USNM 93504 (36.4), Mabul Island, Philippines, 2–8 m, 29 Sep. 1909.

**Remarks.** Although the original description mentioned Mabul Island, Philippines as the type locality, it is now under the jurisdiction of Malaysia. We kept this species on this list.

**(864) *Wetmorellaphilippina* Fowler & Bean, 1928: 211, pl. 17**

= *Wetmorellanigropinnata* (Seale, 1901).

**Holotype.**USNM 89968 (56.0), Little Santa Cruz Island, Zamboanga, Mindanao, 4 m, 26 May 1908.

**Paratypes.**USNM 93503 (1, 40.0), Port Langcan, Dumaran Island, Palawan, 2 m, 8 Apr. 1909; USNM 93505 (1, 39.0), Atulayan Island, Lagonoy Gulf, east of Luzon, 2–3 m, 18 Jun. 1909.

**(865) *Wetmorellatriocellata* Schultz & Marshall, 1954: 447, fig. 54**

= *Wetmorellanigropinnata* (Seale, 1901).

**Holotype.**USNM 93529 (39.2), Rapurapu Island, east coast of Luzon, 24 Jun. 1909.

#### ﻿﻿ORDER PERCIFORMES

##### Family Serranidae (421)

**(866) *Chelidopercasantosi* Williams & Carpenter, 2015: 288, fig. 1**

**Holotype.**PNM 15190 [ex USNM 424586] (69.0), Palawan vicinity, 20 Jun. 2013.

**Paratypes.** NMV A16485 (1, 84.0) and USNM 427531 (1, 69.0) [ex NMV A16485], Bohol Sea, off northern Mindanao, 146 m, 22 Nov. 1979.

**Remarks.** The holotype was bought from Iloilo City fish market, but the vendor said it was caught in Palawan waters.

**(867) *Chelidopercatosaensis* Matsunuma, Yamakawa & Williams, 2017: 3, figs 1a, 2–5**

**Holotype.** BSKU 53312. Off Tosa Bay, Kochi, Japan.

**Paratype.**USNM 437819 (1, 69.5), off Guimaras and Panay islands, 17 Jul. 2015.

**Remarks.** The specimen was bought from Iloilo City fish market, Panay Island. The original description indicated that the paratype was collection on 3 Sep. 2015 but different from the USNM record.

##### Family Scorpaenidae (455)

**(868) *Brachypteroisserrulifer* Fowler, 1938: 79, fig. 35**

**Holotype.**USNM 98886 (115.0), Albatross station 5442 (16°30.60'N, 120°11.10'E), off San Fernando Point Lighthouse, west of Luzon, 82 m, 10 May 1909.

**(869) *Hipposcorpaenafilamentosa* Fowler, 1938: 72, fig. 31**

**Holotype.**USNM 98819 (38.0), Albatross station 5253 (07°04.80'N, 125°39.63'E), off Linao Point, Davao Gulf, Mindanao, 51 m, 18 May 1908.

**(870) *Hypomacrusalbaiensis* Evermann & Seale, 1907: 102**

= *Scorpaenodesalbaiensis* (Evermann & Seale, 1907).

**Holotype.**USNM 55902 (63.5), Bacon, Sorsogon, Luzon.

**Paratype.**CAS-SU 20006 (1, 57.2), same locality as holotype.

**Remarks.** The original description or USNM record did not indicate the collection date of the holotype.

**(871) *Hypomacrusbrocki* Schultz in Schultz et al., 1966: 39, figs 138i, 143**

= *Scorpaenodesminor* (Smith, 1958).

**Holotype.**USNM 99782. Talisse Island, Sulawesi, Indonesia.

**Paratypes.**USNM 99783 (1, 38.5), East of Cruz Point, Maribojoc Bay, Bohol Island, 3–6 m, 26 Mar. 1909; USNM 133076 [ex USNM 136434] (1, 33.1), Limbones Cove, Manila Bay, Luzon, 8 Feb. 1909.

**Remarks.** The original description mentioned that paratype USNM 99783 was collected in Romblon reef but differs from the USNM record. Here, we followed the USNM record.

**(872) *Macroscorpiuspallidus* Fowler, 1938: 76, fig. 33**

= *Lioscorpiuslongiceps* Günther, 1880.

**Holotype.**USNM 98890 (169.0), Albatross station 5518 (08°48.00'N, 123°31.00'E), off Tagolo Point Lighthouse, northern Mindanao, 366 m, 9 Aug. 1909.

**Paratypes.**USNM 98990 (2), USNM 98998 (1), USNM 99001 (1), USNM 99003 (1) and USNM 113004 (21), same locality as holotype, 320–366 m, 9–20 Aug. 1909; CAS-SU 40201 (3), USNM 98995 (15), USNM 98991 (1), USNM 98993 (14) and USNM 98994 (14), Macabalan Point Lighthouse, northern Mindanao, 391–413 m, 4 Aug. 1909; USNM 98989 (4), Malabrigo Lighthouse, east of Mindoro Island, 198 m, 2 Feb. 1908; USNM 98992 (6) and USNM 99005 (2), San Fernando Point Lighthouse, west of Luzon, 315–340 m, 10 May 1909; USNM 98996 (1), USNM 98999 (1) and USNM 99004 (1), Lauis Point Lighthouse, between Cebu and Bohol islands, 291–320 m, 23–25 Mar. 1909; USNM 98997 (3), Albatross station 5118, Sombrero Island, Balayan Bay and Verde Island Passage, Luzon, 291 m, 21 Jan. 1908; USNM 99000 (1), Albatross station 5353, Melville Lighthouse, northern Balabac Strait, Palawan, 271 m, 1 Jan. 1909; USNM 99002 (1), Albatross station 5404, Ponson Island, Dupon Bay, Leyte Island, 347 m, 17 Mar. 1909; USNM 99006 (1), Manila Market, Luzon, 24 Jun. 1908;

**(873) *Nemapontinustentacularis* Fowler, 1938: 73, fig. 32**

= *Pontinustentacularis* (Fowler, 1938).

**Holotype.**USNM 98887 (185.0) Albatross station 5519 (08°47.00'N, 123°31.25'E), off Tagolo Point Lighthouse, northern Mindanao, 333 m, 9 Aug. 1909.

**Paratypes.**USNM 99008 (2, 158.0–168.0), Albatross station 5279, Malavatuan Island, southern Luzon, 214 m, 17 Jul. 1908.

**(874) *Nemapteroisbiocellatus* Fowler, 1938: 81, fig. 36**

= *Dendrochirusbiocellatus* (Fowler, 1938).

**Holotype.**USNM 98894 (83.0), Albatross station 5136 (06°04.33'N, 120°59.33'E), off Jolo Lighthouse, Sulu Archipelago, 40 m, 14 Feb. 1908.

**(875) *Phenacoscorpiusmegalops* Fowler, 1938: 70, fig. 30**

**Holotype.**USNM 98903 (109.0), Albatross station 5387 (12°54.67'N, 123°20.50'E), 27 miles SE of Bagatao Island Lighthouse, between Burias and Luzon islands, 382 m, 11 Mar. 1909.

**Paratypes.**CAS-SU 40198 [ex USNM 136466] (2), between Cebu and Bohol islands, 265 m, 23 Mar 1909; USNM 99012 (1), USNM 136371 (5), USNM 136374 (3) and USNM 136383 (1), Tagolo Point Lighthouse, northern Mindanao, 309–366 m, 9 Aug. 1909; CAS-SU 40199 [ex USNM 136467] (1), same locality as USNM 99012, 20 Aug. 1909; CAS-SU 40200 [ex USNM 136468] (1), between Marinduque and Luzon islands, 170 m, 24 Apr. 1908; USNM 136372 (1), Macabalan Point Lighthouse, northern Mindanao, 413 m, 4 Aug. 1909; USNM 136373 (3), Cabugan Grande Island, between Samar and Leyte islands, 123 m, 30 Jul. 1909; USNM 136375 (2), Ponson Island, Dupon Bay, Leyte Island, 347 m, 17 Mar. 1909; USNM 136376 (2), Lauis Point Lighthouse, between Cebu and Bohol islands, 302 m, 25 Mar. 1909; USNM 136377 (5), USNM 136378 (2) and USNM 136385 (1), Capitancillo Island Lighthouse, between Leyte and Cebu islands, 333–346 m, 16 Mar. 1909; USNM 136380 (1), Albatross station 5388, Bagatao Island Lighthouse, between Burias and Luzon islands, 413 m, 11 Mar. 1909; USNM 136381 (1), Albatross station 5371, Tayabas Lighthouse, Marinduque Island, 152 m, 24 Feb. 1909; USNM 136382 (1), Albatross station 5216, Anima Sola Island, between Burias and Luzon islands, 393 m, 22 Apr. 1908; USNM 136386 (1), Albatross station 5477, Tacbuc Point, between Samar and Leyte islands, 88 m, 29 Jul. 1909.

**Remarks.** Radiographs and photographs of the holotype are available in the USNM record.

**(876) *Pteroidichthysacutus* Motomura & Kanade, 2015: 496, figs 1C, D, 3B**

**Holotype.**MNHN 2001-2851. South of Vele, Wallis and Futuna Islands.

**Paratype.** ASIZP 68097 (1, 36.6), Dingalan, Aurora, Luzon, 8 May 2007; MNHN 2010-0535 (1, 36.7), Musorstrom 3, northwest of Tungao Bay, Caluya Island, south of Mindoro, 73–84 m, 3 Jun 1985.

**(877) *Pteroispaucispinula* Matsunuma & Motomura, 2014: [3] 329, figs 2, 3, 11a**

**Holotype.** KBKU 61062. Kashiwa-jima Island, Kochi, Japan.

**Paratypes.** SMF 12808 (1, 70.6), 1972; USNM 99920 (1, 58.4), Albatross station 5163, Observation Island, Tawi-Tawi, Sulu Archipelago, 51 m, 24 Feb. 1908.

**(878) *Scorpaenaamplisquamiceps* Fowler, 1938: 55, fig. 21**

= *Neomerintheamplisquamiceps* (Fowler, 1938).

**Holotype.**USNM 98883 (152.0), Albatross station 5266 (13°44.60'N, 120°59.25'E), off Matocot Point, Verde Island Passage and Batangas Bay, Luzon, 335 m, 8 Jun. 1908.

**Paratypes.**USNM 98986 (2), same data as holotype; USNM 98985 (1), Albatross station 5135, Jolo Lighthouse, Sulu Archipelago, 294 m, 7 Feb. 1908; USNM 98987 (1), Albatross station 5392, Tubig Point, between Samar and Masbate islands, 247 m, 13 Mar. 1909; USNM 98988 (2), Albatross station 5212, Panalangan Point, east of Masbate Island, 198 m, 20 Apr. 1908.

**(879) *Scorpaenagibbifrons* Fowler, 1938: 58, fig. 23**

= *Neomerintheerostris* (Alcock, 1896).

**Holotype.**USNM 98900 (89.0), Albatross station 5482 (10°27.50'N, 125°18.00'E), off Cabugan Grande Island, between Samar and Leyte islands, 123 m, 30 Jul. 1909.

**Remarks.** Radiographs and photographs of the holotype are available in the USNM record.

**(880) *Scorpaenahemilepidota* Fowler, 1938: 63, fig. 26**

= *Scorpaenaneglecta* Temminck & Schlegel, 1843.

**Holotype.**USNM 98884 (202.0), Albatross station 5392 (12°12.58'N, 124°02.80'E), off Tubig Point, between Samar and Masbate islands, 247 m, 13 Mar. 1909.

**(881) *Scorpaenakaufmani* Herre, 1952: 397**

= *Neomerinthekaufmani* (Herre, 1952).

**Holotype.**USNM 202511 [ex UW 7239] (86.0), entrance to Manila Bay, Luzon, 119 m, 2 Oct. 1947.

**Paratype.**USNM 202570 [ex UW 7254] (1, 56.0), Manila Bay, Luzon, 25 May 1948.

**(882) *Scorpaenamcadamsi* Fowler, 1938: 60, fig. 24**

= *Parascorpaenamcadamsi* (Fowler, 1938).

**Holotype.**USNM 98904 (49.0), Albatross station 5174 (06°03.67'N, 120°57.00'E), Jolo Lighthouse, Sulu Archipelago, 37 m, 25 Mar. 1908.

**Paratypes.**USNM 99013 (1) and USNM 133068 [ex 99013] (1) (35.0–45.0), Albatross station 5254, Linao Point, Davao Gulf, Mindanao, 38 m, 8 May 1908.

**Remarks.**USNM 99013 originally had two specimens, but one was re-identified as *Scorpaenaaltirostris* by Schultz, but erroneously removed from the lot and assigne a new catalog number (USNM 133068). Is still considered as a paratype of *S.mcadamsi* (Smithsonian Museum Archive 2024). Radiographs and photographs of the holotype are available in the USNM record.

**(883) *Scorpaenamegalepis* Fowler, 1938: 56, fig. 22**

= *Neomerinthemegalepis* (Fowler, 1938).

**Holotype.**USNM 98897. China Sea, vicinity of Hong Kong.

**Paratypes.** Uncat., South China Sea and the Philippines, 62–82 m.

**Remarks.** The original description mentioned several paratypes from the South China Sea and the Philippines but did not provide any further information, including collection dates. Paratypes were also not found in the USNM records.

**(884) *Scorpaenapallidimacula* Fowler, 1938: 61, fig. 25**

= *Neomerinthepallidimacula* (Fowler, 1938).

**Holotype.**USNM 98889 (98.0), Albatross station 5241 (06°50.75'N, 126°14.63'E), off Uanivan Island, Pujada Bay, Mindanao, 393 m, 14 May 1908.

**Remarks.** Radiographs and photographs of the holotype are available in the USNM record.

**(885) *Scorpaenataeniophrys* Fowler, 1943: 66, fig. 12**

= *Sebastapistestaeniophrys* (Fowler, 1943).

**Holotype.**USNM 99522 (27.0), Canmahana Bay, Ragay Gulf, Luzon, 11 Mar. 1909.

**Paratype.**USNM 99523 (1, 25.0), same data as holotype.

**Remarks.** Radiographs and photographs of the holotype are available in the USNM record.

**(886) *Scorpaenopsellaarmata* Fowler, 1938: 68, fig. 29**

= *Scorpaenaneglecta* Temminck & Schlegel, 1843.

**Holotype.**USNM 98893 (88.0), Albatross station 5117 (13°52.37N, 120°46.37'E), off Sombrero Island, Balayan Bay and Verde Island Passage, Luzon, 216 m, 21 Jan. 1908.

**Remarks.** Radiographs and photographs of the holotype are available in the USNM record.

**(887) *Scorpaenopsiscotticeps* Fowler, 1938: 65, fig. 27**

**Holotype.**USNM 98891 (38.0), Albatross station 5159 (05°11.83'N, 119°54.0'E), off Tinakta Island, Sulu Archipelago, 18 m, 21 Feb. 1908.

**(888) *Scorpaenopsisobtusa* Randall & Eschmeyer, 2002: 37, fig. 10**

**Holotype.**USNM 169475, Albatross station 5165, Observation Island, Sulu Archipelago, 16.5 m, 24 Feb. 1908.

**Paratype.**CAS 54231 (1), Bararin Island, Cuyo, Palawan, 0–17.4 m, 24 May 1978.

**Remarks.** Radiographs and photographs of the holotype are available in the USNM record.

**(889) *Scorpaenopsisstigma* Fowler, 1938: 66, fig. 28**

= *Phenacoscorpiusmegalops* Fowler, 1938.

**Holotype.**USNM 98896 (69.0), Albatross station 5518 (08°48.00'N, 123°31.00'E), 8.7 miles southwest of Tagolo Point Lighthouse, northern Mindanao, 366 m, 9 Aug. 1909.

**Paratypes.**USNM 99012 (1, 68.0), same data as holotype.

**(890) *Scorpaenopsisvittapinna* Randall & Eschmeyer, 2002: 71, pls IX (fig. D), XII (figs G–H)**

**Holotype.** BPBM 27360. Sodwana Bay, KwaZulu-Natal, South Africa.

**Paratypes.**USNM 364998 [ex CAS 54053] (1, 36.6), LK 79-K, Selinog Island, southwest of Zamboanga, Mindanao, 14–21 m, 2 May 1979; AMS I.40818-001 (1), White Beach reef, Puerto Princesa Bay, Palawan Island, 3 Jul. 1979; BMNH 2001.5.2.36 (1), Tagauayan Island, Cuyo, Palawan, 0–13.7 m, 25 May 1978.

**Other catalog number.**BMNH 2001.5.2.36 (NHMUK:ecatalogue:2573017).

**(891) *Sebastapistesnivifer* Jordan & Seale, 1905: 791, fig. 10**

**Holotype.**USNM 52053 (29.2), Bais, Negros Island, 1901.

**Remarks.** Unknown status in Fricke et al. (2024). Further study is needed to confirm its status. The original description mentioned USNM 51954 as the type specimen, but the USNM record shows it is a type specimen of *Drombuspalackyi*. USNM 52053 could be the holotype based on the USNM record. The confusion might be because both lots have the same collection data (Smithsonian Museum Archive 2024).

**(892) *Taenianotesminutus* Marion de Procé, 1822: 132**

Manila Bay, Luzon.

**Remarks.** No type known. An overlooked available name (Fricke et al. 2024).

##### Family Triglidae (461)

**(893) *Acanthostedionrugosum* Fowler, 1943: 76, fig. 17**

= *Satyrichthysclavilapis* Fowler, 1938.

**Holotype.**USNM 99501 (67.0), Albatross station 5121 (13°27.33'N, 121°17.75'E), off Malabrigo Lighthouse, east of Mindoro Island, 198 m, 2 Feb. 1908.

**Paratypes.**USNM 99502 (7, 55.0–63.0), same data as holotype.

**(894) *Bovitriglaacanthomoplate* Fowler, 1938: 113, fig. 54**

**Holotype.**USNM 98869 (150.0), Albatross station 5519 (08°47.0'N, 123°31.25'E), off Tagolo Point Lighthouse, northern Mindanao, 333 m, 9 Aug. 1909.

**Remarks.** Radiographs of the holotype are available in the USNM record.

**(895) *Dixiphichthyshoplites* Fowler, 1938: 120, fig. 57**

= *Pterygotriglahoplites* (Fowler, 1938).

**Holotype.**USNM 98874 (134.0), Albatross station 5516 (08°46.00'N, 123°32.50'E), off Tagolo Point Lighthouse, northern Mindanao, 320 m, 9 Aug. 1909.

**Paratype.**USNM 98982 (1, 128.0), Albatross station 5412, Lauis Point Lighthouse, between Cebu and Bohol islands, 296 m, 23 Mar. 1909.

**(896) *Dixiphistesmacrorhynchus* Fowler, 1938: 118, fig. 56**

= *Pterygotriglamacrorhynchus* Kamohara 1936.

**Holotype.**USNM 98875 (159.0), Albatross station 5519 (08°47.00'N, 123°31.25'E), off Tagolo Point Lighthouse, northern Mindanao, 333 m, 9 Aug. 1909.

**(897) *Dixiphistopsmegalops* Fowler, 1938: 116, fig. 55**

= *Pterygotriglamegalops* (Fowler, 1938).

**Holotype.**USNM 98879 (146.0), Albatross station 5441 (16°38.00'N, 119°57.30'E), off San Fernando Point Lighthouse, west of Luzon, 340 m, 10 May 1909.

**(898) *Gargariscussemidentatus* Smith, 1917: 145**

= *Gargariscusprionocephalus* (Duméril, 1869).

**Holotype.**USNM 78249 (227.0), Albatross station 5517, off Tagolo Point Lighthouse, northern Mindanao, 309 m, 9 Aug. 1909.

**Paratypes.**USNM 98906 (2), same data as holotype; USNM 98908 (1) and USNM 135937 (1), Albatross station 5367, Malabrigo Lighthouse, Verde Island Passage, Luzon, 329 m, 22 Feb. 1909; USNM 98909 (1), Albatross station 5409, Capitancillo Lighthouse, between Cebu and Leyte islands, 346 m, 18 Mar. 1909; USNM 98910 (1) and USNM 98978 (1),Albatross station 5417, Lauis Point Lighthouse, between Cebu and Bohol islands, 291 m, 25 Mar. 1909; USNM 98911 (0, lost), Albatross station 5411, Lauis Point Lighthouse, between Cebu and Bohol Islands, 265 m, 23 Mar. 1909; USNM 98912 (1), Albatross station 5272, Corregidor Lighthouse, southern Luzon, 216 m, 14 Jul. 1098; and USNM 98913 (0), Albatross station 5117, Sombrero Island, between Balayan Bay and Verde Island Passage, Luzon, 216 m, 21 Jan. 1908.

**Remarks.** Size range of paratypes: 65.0–250.0. USNM 98911 and 98913 were presumed lost while on loan (Smithsonian Museum Archive 2024).

**(899) *Heminodusphilippinus* Smith, 1917: 146**

**Holotype.**USNM 78250 (168.0), Albatross station 5517, off Tagolo Point Lighthouse, northern Mindanao, 309 m, 9 Aug. 1909.

**Paratypes.**USNM 98914 (1), taken with the holotype, 320 m; USNM 98915 (1), Albatross station 5576, Mt. Dromedario, north of Tawi Tawi, Sulu Archipelago, 507 m, 23 Sep. 1909.

**Remarks.** Radiographs of the holotype are available in the USNM record.

**(900) *Lepidotriglaargyrosoma* Fowler, 1938: 105, fig. 50**

**Holotype.**USNM 98873 (94.0), Albatross station 5442 (16°30.60'N, 120°11.10'E), off San Fernando Point Lighthouse, west of Luzon, 82 m, 10 May 1909.

**Paratypes.**USNM 98981 (10), same data as holotype.

**Remarks.** The original description did not provide further information for paratypes. USNM 98981 are “unconfirmed paratype” based on the USNM record.

**(901) *Lepidotrigladeasoni* Herre & Kauffman, 1952: 28**

**Holotype.**USNM 202504 [ex UW 18958] (97.0), east entrance of Manila Bay, Luzon, 119 m, 2 Oct. 1947.

**Paratypes.**USNM 202569 [ex UW 11513] (1, 87.0), same data as holotype; UW 011509 (5, 80.0–92.0), same locality as holotype, 1 Oct. 1947.

**(902) *Lepidotriglaeydouxii* Sauvage, 1878: 156**

**Holotype.**MNHN 0000-6809 (140.0), Manila, Luzon.

**Remarks.** The original description or MNHN record did not indicate the collection date.

**(903) *Lepidotriglamacrobrachium* Fowler, 1938: 109, fig. 52**

**Holotype.**USNM 98882 (64.0), Albatross station 5432 (10°37.83'N, 120°12.00'E), Corandagos Island, eastern Palawan, 93 m, 8 Apr. 1909.

**(904) *Lepidotriglapectoralis* Fowler, 1938: 104, fig. 49**

**Holotype.**USNM 98878 (140.0), Albatross station 5517 (08°45.50'N, 123°33.75'E), off Tagolo Point Lighthouse, northern Mindanao, 309 m, 9 Aug. 1909.

**(905) *Lepidotriglapunctipectoralis* Fowler, 1938: 107, fig. 51**

**Holotype.**USNM 98871 (175.0), Albatross station 5135 (06°11.83'N, 121°08.33'E), Jolo Lighthouse, Sulu Archipelago, 295 m, 7 Feb. 1908.

**Paratype.**USNM 98980 (1, 160.0), Albatross station 5392, Tubig Point, between Samar and Masbate islands, 247 m, 13 Mar. 1909.

**(906) *Lepidotriglavenusta* Fowler, 1938: 103, fig. 48**

**Holotype.**USNM 98872 (111.0), Albatross station 5442 (16°30.60'N, 120°11.10'E), off San Fernando Point Lighthouse, west of Luzon, 82 m, 10 May 1909.

**Paratypes.**USNM 98977 (34), same data as holotype.

**(907) *Nemaperistedionorientale* Fowler, 1938: 127, fig. 61**

= *Scalicusorientalis* (Fowler, 1938).

**Holotype.**USNM 98876. Off Makyan Island, between Gillolo and Makyan Islands, Indonesia.

**Paratypes.**USNM 98916 (1, 141.0), Albatross station 5222, San Andreas Island, between Marinduque and Luzon islands, 357 m, 24 Apr. 1908; USNM 98919 (1, 173), Simaluc Island, Tataan, Sulu Archipelago, 2–12 m, 20 Feb. 1908; USNM 98921 (1, 143.0), Albatross 5518, Tagolo Point Lighthouse, northern Mindanao, 9 Aug. 1909.

**Remarks.** The original description did not mention the locality of USNM 98919 which was assumed based on the linen tag number from the USNM record.

**(908) *Otohimetagala* Herre & Kauffman, 1952: 27**

= *Pterygotriglatagala* (Herre & Kauffman, 1952).

**Holotype.**USNM 202506 [ex UW 18957] (93.0), outer Manila Bay, Cavite, Luzon, 117 m.

**Paratypes.**USNM 202565 [ex UW 11515] (3), 117 m, 2 Oct 1947; USNM 202566 [ex UW 11516] (1), 99 m, 1 Oct. 1947, both taken with holotype; UW (5).

**Remarks.** The original description mentioned type specimens without catalog numbers: one paratype (91.0) from the east entrance to Manila Bay, off to Cavite, 99 m, and five paratypes (78.0–98.0) off the coast of Batangas, Luzon, 119 m. USNM records provided catalog numbers for the type specimens (Smithsonian Museum Archive 2024). The original description or USNM record did not indicate the collection date.

**(909) *Paraheminoduskamoharai* Kawai, Imamura & Nakaya, 2004: 126, figs 1–3**

**Holotype.** BSKU 15243 (female, 106.9), 08°11.80'N, 117°58.00'E, off Bataraza, Palawan Island, 285 m, 26 May 1972.

**Paratypes.** BSKU 15242 (female, 107.3) and BSKU 15244 (1, 114.8), taken with holotype.

**(910) *Peristedionamblygenys* Fowler, 1938: 122, fig. 58**

**Holotype.**USNM 98870 (162.0), Albatross station 5442 (16°30.60'N, 120°11.10'E), off San Fernando Point Lighthouse, west of Luzon, 82 m, 10 May 1909.

**Paratypes.**USNM 98924 (1), Malapascua Island, Cebu, 3–6 m, 16 Mar. 1909; USNM 98925 (3), Albatross station 5372, Tayabas Lighthouse, Marinduque Island, 274 m, 24 Feb. 1909; USNM 98926 (1) and USNM 98927 (18), Albatross station 5121, Malabrigo Lighthouse, east of Mindoro Island, 198 m, 2 Feb. 1908.

**(911) *Peristedionlongiceps* Fowler, 1943: 74, fig. 16**

= *Satyrichthyslongiceps* (Fowler, 1943).

**Holotype.**USNM 99516 (195.0), Albatross station 5374 (13°46.75'N, 121°35.13'E), off Tayabas Lighthouse, Marinduque Island, 347 m, 2 Mar. 1909.

**(912) *Peristedionwelchi* Herre, 1925a: 292, pl. 1**

= *Satyrichthyswelchi* (Herre, 1925).

**Holotype.** BSMP (35.0, TL), fish corral on the reef in front of Dumaguete, Negros Island, 6–8 m.

**Remark.** The type specimen was presumed destroyed.

**(913) Pterygotrigla (Otohime) urashimai Richards, Yato & Last, 2003: 12, figs 1H, 13**

= *Pterygotriglaurashimai* Richards, Yato & Last, 2003.

**Holotype.**USNM 202567 [ex UW 11515] (93.6), off Cavite, the southern entrance to Manila Bay, Luzon, 119 m, 2 Oct. 1947.

**Paratype.**USNM 356931 [ex USNM 202567] (1, 82.3), taken with holotype.

**(914) Pterygotrigla (Pterygotrigla) cajorarori Richards & Yato, 2012: 57, figs 2–6**

= *Pterygotriglacajorarori* Richards & Yato, 2012.

**Holotype.**USNM 135940 (198.0), 08°36.43'N, 124°36.13'E, 6.6 miles off Macalaban Point Lighthouse, northern Mindanao, 413 m, 4 Apr. 1909.

**Remarks.** The original description mentioned USNM 135940 as the holotype but was not found in the USNM record. It may not have been deposited yet.

**(915) *Satyrichthysclavilapis* Fowler, 1938: 123, fig. 59**

**Holotype.**USNM 98868 (244.0), Albatross station 5118 (13°48.75'N, 120°41.85'E), Sombrero Island, Balayan Bay and Verde Island Passage, Luzon, 291 m, 21 Jan. 1908.

**Paratypes.**USNM 98939 (5), same data as holotype; USNM 98928 (2), Albatross station 5523, Tagolo Point Lighthouse, northern Mindanao, 10 Aug. 1909; USNM 98929 (2), Albatross station 5110, Corregidor Lighthouse, off southern Luzon, 247 m, 15 Jan. 1908; USNM 98930 (1) and USNM 98938 (1), Albatross stations 5112-13, Sombrero Island, off southern Luzon, 324–326 m, 17 Jan. 1908; USNM 98931 (1), Albatross station 5503, Macabalan Point Lighthouse, northern Mindanao, 413 m, 4 Aug. 1909; USNM 98932 (1), Albatross station 5576, Mt. Dromedario, north of Tawi-tawi, Sulu Archipelago, 507 m, 23 Sep. 1909; USNM 98933 (3), Albatross station 5179, Romblon Lighthouse, Romblon, 68 m, 25 Mar 1908; USNM 98934 (1) and USNM 98936 (2), Albatross stations 5518-19, Tagolo Point Lighthouse, northern Mindanao, 333 m, 9 Aug. 1909; USNM 98935 (2), Albatross station 5247, Dumalag Island, Davao Gulf, Mindanao, 247 m, 18 May 1908; USNM 98937 (1), Albatross station 5374, Tayabas Lighthouse, Marinduque Is, 347 m, 2 Mar. 1909.

**(916) *Satyrichthysmilleri* Kawai, 2013: 429, fig. 7–9**

**Holotype.** HUMZ 193886. Off Java, Indonesia.

**Paratypes.**MNHN 1984-0663 (1), west of Luzon, 550 m, Dec. 1980; USNM 135936 (1), Albatross station 5505, Macabalan Point Lighthouse, northern Mindanao, 402 m, 5 Aug. 1909; USNM 164367 (10) and USNM 164374 (1), Albatross stations 5402-03, Capitancillo Island Lighthouse, between Leyte and Cebu islands, 333–344 m, 16 Mar. 1909; USNM 164368–69 (9, 3), Albatross stations 5411-12, Lauis Point Lighthouse, between Cebu and Bohol islands, 265–296 m, 23 Mar. 1909; USNM 164370 (3), Albatross station 5247, Dumalag Island, Davao Gulf, Mindanao, 247 m, 18 May 1908; USNM 164372 (2), Albatross station 5194, Chocolate Island, off northern Cebu, 271 m, 3 Apr. 1908; USNM 164375 (1), Albatross station 5222, San Andreas Island, between Marinduque and Luzon islands, 357 m, 24 Apr. 1908; USNM 164373 (1), Albatross station 5441, San Fernando Point Lighthouse, west of Luzon, 340 m, 10 May 1909.

##### Family Bembridae (463)

**(917) *Brachybembrasaschemeieri* Fowler, 1938: 94, fig. 43**

**Holotype.**USNM 98881 (67.0), Albatross station 5172 (06°03.25'N, 120°35.50'E), off Jolo Lighthouse, Sulu Archipelago, 582 m, 5 Mar. 1908.

**Remarks.** Radiographs and photographs of the holotype are available in the USNM record.

**(918) *Parabembrasmultisquamata* Kai & Fricke, 2018: 69, figs 1C, 2C**

**Holotype.**MNHN-IC-2008-1516. Big Bay, Espiritu Santo, Vanuatu.

**Paratypes.**MNHN-IC-1984-0687 (1, 170.1), Musortstom 2 station 26, Balayan Bay, southwestern Luzon, 299–320 m, 23 Nov. 1980.

##### Family Platycephalidae (464)

**(919) *Cymbacephalusarmatus* Fowler, 1938: 90, fig. 41**

= *Thysanophrysarmata* (Fowler, 1938).

**Holotype.**USNM 98864 (142.0), Albatross station 5148 (05°35.67'N, 120°47.50'E), off Sirun Island, Sulu Archipelago, 31 m, 16 Feb. 1908.

**(920) *Elatesthompsoni* Jordan & Seale, 1907: 39, fig. 12**

= *Elatesransonnettii* (Steindachner, 1876).

**Holotype.**USNM 53068, Manila, Luzon.

**Paratypes.**CAS-SU 9247 (1) and CAS-SU9975 (1) (152.4–177.8), same locality as the holotype.

**Remarks.** The original description or USNM record did not indicate the collection date of the holotype.

**(921) *Platycephalusfasciatus* Günther, 1872: 397**

= *Cociellapunctata* (Cuvier, 1829).

**Holotype.**BMNH 1872.10.18.117 (177.8), Manila Bay, Luzon.

**Other catalog number.** NHMUK:ecatalogue:3104691.

**Remarks.** The original description or NMH record did not indicate the collection date.

**(922) *Thysanophryschiltonae* Schultz in Schultz et al., 1966: 57, figs 146, 147**

**Holotype.**USNM 141009. Rongelap Island, Rongelap Atoll, Marshall Islands.

**Paratypes.**USNM 99652 (1, 158.0), Albatross station 5235, 7 miles off Nagubat Island, east coast of Mindanao, 227 m, 9 May 1908.

**(923) *Wakiyusablani* Herre, 1953b: 12**

**Holotype.**USNM 202514 [ex UW 19700], off Corregidor Island, southern Luzon, 31–42 m, 25 May 1953.

**Paratypes.**USNM 202568 [ex UW 8505] (8), same data as holotype.

**Remarks.** Unknown status in Fricke et al. (2024). A further study is needed to confirm its status.

##### Family Hoplichthyidae (465)

**(924) *Acanthoplichthyspectoralis* Fowler, 1943: 72, fig. 15**

= *Hoplichthyspectoralis* (Fowler, 1943).

**Holotype.**USNM 99503 (73.0), Albatross station 5440 (16°33.87'N, 119°52.90'E), off San Fernando Point Lighthouse, west of Luzon, 315 m, 10 May 1909.

**(925) *Monhoplichthysgregoryi* Fowler, 1938: 96, fig. 44**

= *Hoplichthysgregoryi* (Fowler, 1938).

**Holotype.**USNM 98862 (202.0), Albatross station 5519 (08°47.00'N, 123°31.25'E), off Tagolo Point Lighthouse, northern Mindanao, 333 m, 9 Aug. 1909.

**Paratypes.**USNM 150813 (6, 86.0–210.0), same data as holotype.

**(926) *Monhoplichthysprosemion* Fowler, 1938: 97, fig. 45**

= *Hoplichthysprosemion* (Fowler, 1938).

**Holotype.**USNM 98863 (173.0), Albatross station 5117 (13°52.37'N, 120°46.37'E), off Sombrero Island, Balayan Bay and Verde Island Passage, Luzon, 216 m, 21 Jan. 1908.

##### Family Anthiadidae

**(927) Anthias (Pseudanthias) luzonensis Katayama & Masuda, 1983: 340, figs 1, 2**

= *Pseudanthiasluzonensis* (Katayama & Masuda, 1983).

**Holotype.** NSMT-P 21435 (98 .0), off Batangas, Luzon, 10 m, 10 May 1981.

**Paratype.** NSMT-P 21436 (1 female, 81.0), taken with holotype.

**(928) Anthias (Mirolabrichthys) parvirostris Randall & Lubbock, 1981: 6, fig. 2**

= *Pyronotanthiasparvirostris* (Randall & Lubbock, 1981).

**Holotype.** BPBM 15605. Alite Reef, off Malaita, Solomon Islands.

**Paratype.**BMNH 1979.1.3.1 (1, 58.9), Taguan River, Puerto Princesa, Palawan Island, 35 m, 17 Aug. 1978.

**Other catalog number.**BMNH 1979.1.3.1 (NHMUK:ecatalogue:2545083).

**(929) *Anthiasrandalli* Lubbock & Allen, 1978: 260, figs 1, 2**

= *Pseudanthiasrandalli* (Lubbock & Allen, 1978).

**Holotype.**BMNH 1977.1.21.1 (male, 69.7), a large cave on vertical drop-off, Baring, Olango Island, Cebu, 40 m, 18 Aug. 1976.

**Paratypes.** AMS I.19756-001 (1, 66.3), MHNG 1551.39 (1, 52.4) and USNM 217534 (1, 56.7), taken with holotype; BMNH 1977.1.21.2 (1, 65.6), same locality as holotype, 3 Aug. 1976; BMNH 1977.1.21.3 (1, 41.1), vertical drop-off, 0.5 km north of Caubyan Daku Island, Camotes Sea, 30 m, 15 Aug. 1976.

**Other catalog number.**BMNH 1977.1.21.1 (NHMUK:ecatalogue:2543330), BMNH 1977.1.21.3 (NHMUK:ecatalogue:2543332).

**Remarks.** The original description mentioned BMNH 1976.1.21.2 and BMNH 1976.1.21.3 but in errors since the NMH record matches *Anthiasrandalli* paratypes with BMNH 1977.1.21.2 and BMNH 1977.1.21.3. Similarly, all NHM records indicated two specimens sharing the same catalog number.

**(930) *Anthiassmithvanizi* Randall & Lubbock, 1981: 9, figs 7, 8**

= *Pyronotanthiassmithvanizi* (Randall & Lubbock, 1981).

**Holotype.** BPBM 15606. Alite Reef, off Malaita, Solomon Islands.

**Paratypes.**USNM 215964 [ex 164978] (14, 41.0–58.0), Cagayancillo Island, Philippines, 1–15 m, 31 Mar. 1909.

**(931) *Mirolabrichthysimeldae* Burgess, 1977: 39**

= *Pyronotanthiaslori* (Lubbock & Randall, 1976).

**Holotype.**USNM 216923, Philippines.

**Paratypes.**USNM 216922 (2), Philippines.

**Remarks.** The original description or USM record did not provide specific localities. Radiographs of the holotype are available in the USNM record.

**(932) *Mirolabrichthystuka* Herre & Montalban, 1927 in Herre 1927a: 413, pl. 1**

**Holotype.** BSMP, Maricaban Island, Luzon.

**Paratypes.** BSMP (3, 84.0–94.0), same locality as holotype.

**Remarks.** The original description did not indicate the collection date. All type specimens were presumed destroyed.

**(933) *Mirolabrichthyswaitei* Fowler, 1931: 228, fig. 16**

= *Luzonichthyswaitei* (Fowler, 1931).

**Holotype.**USNM 89997 (53.0), Port Maricaban, Luzon, 4–6 m, 21 Jul. 1908.

**Paratypes.**USNM 323542 (7) and USNM 329439 [both ex USNM 89997] (1, 47.5), same data as holotype.

**Remarks.** Fowler drew the “type” on his original description. This is considered to be the holotype based on its size. Seven paratypes and one paratype mixed with holotype were re-cataloged as USNM 323542 and USNM 329439, respectively. Randall and McCosker (1992) incorrectly designated USNM 329439 as lectotype, since the holotype is still available and identifiable. Radiographs of the holotype are available in the USNM record (Smithsonian Museum Archive 2024).

**(934) *Plectranthiasforesti* Fourmanoir, 1977: 269, fig. 2**

**Holotype.**MNHN 1976-0377 (72.0), Musorstom 1 station 3 (14°01.02'N, 120°16.02'E, Lubang Island, off west of Luzon, 183–185 m, 19 Mar. 1976.

**Paratypes.** BPBM 20873 (2, 52.0–56.0), same data as holotype.

**(935) *Plectranthiasinermis* Randall, 1980: 135, fig. 11**

**Holotype.** BPBM 22468 (male, 34.2), ca. 13°41.00'N 120°50.00'E, southwest of Caban Island, Batangas, Luzon, 30 m, 28 Jul. 1978.

**Paratypes.** BPBM 22460 (3, 27.7–29.2), same data as holotype.

**(936) *Plectranthiasknappi* Randall, 1996b: 126, fig. 2**

**Holotype.**USNM 219865 (female, 66.0), 11°38.00'N, 123°52.63'E, southwest of Caruruan Point, between northern Negros and Masbate islands, 0–89.7 m, 5 Jun. 1978.

**(937) *Plectranthiasmaculatus* Fourmanoir, 1982: 58, fig. 1**

= *Selenanthiasanalis* Tanaka, 1918.

**Holotype.**MNHN 1981-1438, Musorstom 2 station 2 (14°01.02'N, 120°16.98'E), off Lubang Island, west of Luzon, 184–186 m, 20 Nov. 1980.

**Paratypes.**MNHN 1981-1439 (1), same data as holotype.

##### Family Bembropidae

**(938) *Bembropsfilifer* Fowler, 1938: 92, fig. 42**

= *Bembropsplatyrhynchus* (Alcock, 1894).

**Holotype.**USNM 98866 (260.0), Albatross station 5216 (12°52.00'N, 123°23.50'E), Anima Sola Island, between Burias and Luzon islands, 393 m, 22 Apr. 1908.

**Paratypes.**CAS-SU 8645 (1), CAS-SU 40194 [ex USNM 98973] (1); USNM 98940 (2), Albatross station 5537, Apo Island, Negros, 465 m, 18 Aug. 1909; USNM 98942 (1) and 98950 (2), Albatross station 5222, San Andreas Island, between Marinduque and Luzon islands, 353-357 m, 24 Apr. 1908; USNM 98943 (1) and 98953 (2), Albatross station 5372, Tayabas Lighthouse, Marinduque Island and vicinity, 274–347 m, 24 Feb. – 2 Mar. 1909; USNM 98944 (4), 98946 (3), 98949 (3), 98951 (4), 98954 (5) and 98968 (1), Albatross station 5418, Lauis Point Lighthouse, between Cebu and Bohol islands, 265–320 m, 23-25 Mar. 1909; USNM 98945 (1), Albatross station 5110, Corregidor Lighthouse, off southern Luzon, 247 m, 15 Jan. 1908; USNM 98947 (1), 98956 (2) and 98971 (1), Albatross station 5501, Macabalan Point Lighthouse, northern Mindanao and vicinity, 391–413 m, 4 Aug. 1909; USNM 98948 (1), Albatross station 5440, San Fernando Lighthouse, west coast of Luzon, 315 m, 10 May 1909; USNM 98952 (2), Albatross station 5508, Camp Overton Lighthouse, northern Mindanao and vicinity, 494 m, 5 Aug. 1909; USNM 98953 (1), 98958 (2) and 98960 (6), Albatross station 5402, Capitancillo Island Lighthouse, between Leyte and Cebu islands, 333–344 m, 16-18 Mar. 1909; USNM 98957 (6), 98964 (1), 98970 (1), 98973 (1) and 98974 (3), Tagolo Point Lighthouse, northern Mindanao and vicinity, 187–320 m, 9-20 Aug. 1909; USNM 98962 (1), Albatross station 5260, Balanja Point, off southeastern Mindoro Island, 428 m, 3 Jun. 1908; USNM 98975 (2), Albatross station 5255, Dumalag Island, Gulf of Davao, 183 m, 18 May 1908.

**Remarks.** Objectively invalid; preoccupied by *Bembropsfilifera* Gilbert, 1905, replaced by *Bembropsphilippinus* Fowler, 1939, and replaced by *Bembropsfilamentosa* Norman, 1939. *Bembropsphilippinus* Fowler, 1939 was assigned on 17 May 1939 while *Bembropsfilamentosa*Norman 1939 was assigned on 25 Nov. 1939.

**(939) *Bembropsphilippinus* Fowler, 1939: 2**

**Holotype.**USNM 98866 (260.0), Albatross station 5216 (12°52.00'N, 123°23.50'E), Anima Sola Island, between Burias and Luzon islands, 393 m, 22 Apr. 1908.

**Paratypes.**CAS-SU 8645 (1), CAS-SU 40194 [ex USNM 98973] (1); USNM 98940 (2), Apo Island, Negros, 465 m, 18 Aug. 1909; USNM 98942 (1) and 98950 (2), San Andreas Island, between Marinduque and Luzon islands, 353–357 m, 24 Apr. 1908; USNM 98943 (1) and 98953 (2), Tayabas Lighthouse, Marinduque Island and vicinity, 274–347 m, 24 Feb. – 2 Mar. 1909; USNM 98944 (4), 98946 (3), 98949 (3), 98951 (4), 98954 (5) and 98968 (1), Lauis Point Lighthouse, between Cebu and Bohol islands, 265–320 m, 23–25 Mar. 1909; USNM 98945 (1), Corregidor Lighthouse, off southern Luzon, 247 m, 15 Jan. 1908; USNM 98947 (1), 98956 (2) and 98971 (1), Macabalan Point Lighthouse, northern Mindanao and vicinity, 391–413 m, 4 Aug. 1909; USNM 98948 (1), San Fernando Lighthouse, west coast of Luzon, 315 m, 10 May 1909; USNM 98952 (2), Camp Overton Lighthouse, northern Mindanao and vicinity, 494 m, 5 Aug. 1909; USNM 98953 (1), 98958 (2) and 98960 (6), Capitancillo Island Lighthouse, between Leyte and Cebu islands, 333–344 m, 16–18 Mar. 1909; USNM 98957 (6), 98964 (1), 98970 (1), 98973 (1) and 98974 (3), Tagolo Point Lighthouse, northern Mindanao and vicinity, 187–320 m, 9–20 Aug. 1909; USNM 98962 (1), Balanja Point, off southeastern Mindoro Island, 428 m, 3 Jun. 1908; USNM 98975 (2), Dumalag Island, Gulf of Davao, 183 m, 18 May 1908.

**Remarks.** A replacement name for *Bembropsfilifer* Fowler, 1938. *Bembropsphilippinus* Fowler, 1939 was assigned on 17 May 1939 while *Bembropsfilamentosa*Norman 1939 was assigned on 25 Nov. 1939. Priority was given to *B.philippinus*. Radiographs of the holotype are available in the USNM record.

**(940) *Bembropsnelsoni* Thompson & Suttkus, 2002: 290, fig. 9**

**Holotype.** NTM S.14950-008. North of Lynedoch Bank, Arafura Sea, Indonesia.

**Paratypes.**USNM 98943 (1), Albatross station 5372, Tayabas Lighthouse, Marinduque Island, 274 m, 25 Feb. 1909; USNM 98968 (1), Albatross station 5412, Lauis Point Lighthouse, between Cebu and Bohol islands, 296 m, 23 Mar. 1909; USNM 351307 (1) and USNM 351308 [ex USNM 09851] (1), Albatross station 5417, same locality as USNM 98943, 302 m, 25 Mar. 1909; USNM 390289 [ex USNM 98969] (1), Albatross station 5403, Capitancillo Island, between Leyte and Cebu islands.

**Remarks.** Specimens are also paratypes of *Bembropsphilippinus* (above).

##### Family Epinephelidae

**(941) C *ephalopholis aitha* Randall & Heemstra, 1991: 29, pl. 1c, fig. 5**

**Holotype.** BPBM 32655. off Nagada Harbor, Madang, Papua New Guinea.

**Paratypes.**USNM 236456 (1), SP 78-43, Pamilacan Island, Bohol, 0–24 m, 12 Jun. 1978; BMNH 1989.2.3.1 (1), same data as above.

**Other catalog number.**BMNH 1989.2.3.1 (NHMUK:ecatalogue:2559471).

**Remark.** One USNM 236456 specimen was exchanged to BPBM on 5 Feb. 1986.

**(942) *Cephalopholisalbomarginatus* Fowler & Bean, 1930: 235, fig. 11**

= *Gracilaalbomarginata* (Fowler & Bean, 1930).

**Holotype.**USNM 89985. Danawan Island, Sibuko Bay, Borneo.

**Paratypes.**USNM 149319 (1, 185.0), Albatross station 5216, Anima Sola Island, between Burias and Luzon islands, 393 m, 22 Apr. 1908; USNM 183851 (1, 298.0), Sablayan Bay, Mindoro Island, 2–4 m, 12 Dec. 1908; USNM 183852 (1, 330.0), Port Maricaban, southern Luzon, 4–6 m, 21 Jul. 1908.

**(943) *Cephalopholiskendalli* Evermann & Seale, 1907: 76, fig. 11**

= *Cephalopholiscyanostigma* (Valenciennes, 1828).

**Holotype.**USNM 55911 (190.5), Bacon, Sorsogon, Luzon.

**Remarks.** The original description or USNM record did not indicate the collection date.

**(944) *Cephalopholismaculatus* Seale & Bean, 1907: 235, fig. 5**

= *Cephalopholisminiata* (Forsskål, 1775).

**Holotype.**USNM 57843 (247.7), Zamboanga, Mindanao.

**Paratype.**USNM 61153 (1, 247.7), same locality as holotype.

**Remarks.** The original description or USNM record did not indicate the collection date.

**(945) *Cephalopholisobtusauris* Evermann & Seale, 1907: 77, fig. 12**

= *Cephalopholisaurantia* (Valenciennes, 1828).

**Holotype.**USNM 55910 (232.4), Bacon, Sorsogon, Luzon.

**Remarks.** The original description or USNM record did not indicate the collection date.

**(946) *Epinephelusalbimuculatus* Seale, 1910: 509, pl. 8**

= *Epinephelusbleekeri* (Vaillant 1878).

**Holotype.** BSMP 1908 (280.0), Butuan Bay, Mindanao, 26 Sep. 1908.

**Remarks.** The type specimen was presumed destroyed.

**(947) *Epinephelusmatterni* Fowler, 1918: 31, fig. 13**

= *Epinephelusrivulatus* (Valenciennes, 1830).

**Holotype.**ANSP 47506 (178.0), Philippines.

**Remarks.** The original description or ANSP did not provide a specific locality or collection date. A photograph and a radiograph are available in ANSP’s record.

**(948) *Plectropomustruncatus* Fowler & Bean, 1930: 196, fig. 5**

= *Plectropomusareolatus* (Rüppell, 1830).

**Holotype.**USNM 89984 (350.0), Atulayan Island, Lagonoy Gulf, east of Luzon, 2–3 m, 18 Jun. 1909.

**Paratypes.**USNM 149324 (1, 137.0 TL), Tumindao Island, Sibutu Island, Sulu Archipelago, 3–5 m, 26 Feb. 1908; USNM 149396 (1, 85.0), Alimango Bay, Burias Island, Masbate, 4–7 m, 5 Mar. 1909; Uncat. (2, 287.0–314.0 TL) Endeavor Strait, Malampaya Sound, Taytay, Palawan Island, 23 Dec. 23, 1908; (1, 323.0 TL), Gigoso Point, Samar Island, 28 Jul. 1909; Uncat. (1, 518.0 TL), Port Matalvi, Luzon, 23 Nov 1908; (1, 425.0 TL), Sulade Island, Jolo, Sulu Archipelago, 17 Sep. 1909; Uncat. (1, 370.0 TL), Tapiantana Island, south of Zamboanga, Mindanao, 13 Sep. 1909.

**Remarks.** The original description mentioned that USNM 149324 was taken in Atulayan Bay, Luzon on 18 Jun. 1909, but the USNM record showed this was taken in Tumindao Island, Sulu Archipelago on 26 Feb. 1908. One specimen (USNM 321679) in the USNM record with the same collection locality with one of the un-cataloged paratypes. Further clarification is needed (Smithsonian Museum Archive 2024).

##### Family Grammistidae

**(949) *Pseudogrammaerythreum* Randall & Baldwin, 1997: 25, pl. 1 (fig. D)**

= *Pseudogrammaerythrea* Randall & Baldwin, 1997.

**Holotype.** BPBM 22233, 13°39.00'N 120°50.00'E, north of Caban Island, Batangas, off southwest Luzon, 30 m, 2 Sep. 1977.

##### Family Liopropomatidae

**(950) *Liopropomacollettei* Randall & Taylor, 1988: 30, pl. 3A**

**Holotype.** BPBM 14652. Kaneohe Bay, Oahu Island, Hawaiian Islands.

**Paratypes.**USNM 219867 (1), SP 78-10, off Bonbonon Point at the southern tip of Negros Island, 0–18.3 m, 13 May 1978; USNM 285954 (1), SP 78-11, same locality and collection date as preceding, 0–12 m; USNM 219868 (1, 45.6), SP 78-41, off the southwest tip of Pamilacan Island, Bohol, 0–33.5 m, 12 Jun. 1978; USNM 285948 (2), SP 78-38, west of Balicasag Island, Luzon, 0–24 m, 10 Jun. 1978;

**Remarks.** The original description mentioned two specimens each in USNM 219867 and 219868 but only one was found in the USNM records.

**(951) *Ypsigrammalineata* Schultz in Schultz et al., 1953: 375, fig. 59**

= *Liopropomasusumi* (Jordan & Seale, 1906).

**Holotype.**USNM 141872. Off Namu Island, Bikini Atoll, Marshall Islands.

**Paratypes.**USNM 141874 (1, 66.7), Baganga Bay, eastern Mindanao, 3–9 m, 18 May 1908; USNM 141875 (1, 36.5, in bad condition), Paluan Bay, Mindoro Island, 3–6 m, 11 Dec. 1908.

**Remarks.** Radiographs of the paratypes are available in the USNM record.

##### Family Synanceiidae

**(952) *Aploactoidesphilippinus* Fowler, 1938: 88, fig. 40**

= *Erisphexphilippinus* (Fowler, 1938).

**Holotype.**USNM 98880 (119.0), Albatross station 5504 (08°35.50'N, 124°36.00'E), off Macabalan Point Lighthouse, northern Mindanao, 366 m, 5 Aug. 1909.

**Paratypes.**USNM 135949 (1), Albatross station 5536, Apo Island, between Negros and Siquijor islands, 510 m, 19 Aug. 1909; USNM 135950 (1), USNM 136348 (3), USNM 136349 (1), USNM 136351 (2) and USNM 136353 (1), Albatross station 5502, Macabalan Point Lighthouse, northern Mindanao, 366–413 m, 4 Aug. 1909; USNM 136350 (1), Albatross station 5505, Bagatao Island Lighthouse, between Burias and Luzon islands, 382 m, 11 Mar. 1909; USNM 136352 (1), Albatross station 5365, Cape Santiago Lighthouse, Balayan Bay, Luzon, 391 m, 22 Feb. 1909; USNM 136354 (1), Albatross station 5221, San Andreas Island, between Marinduque and Luzon Islands, 353 m, 24 Apr. 1908; USNM 136441 (1), Albatross station 5374, Tayabas Lighthouse, Marinduque Island, 347 m, 2 Mar. 1909.

**(953) *Coccotropusobbesi* Weber, 1913: 503, figs 104–105**

= *Paraploactisobbesi* (Weber, 1913).

**Holotype.** ZMA 110243, Siboga station 99 (06°07.50'N, 120°26.00'E), north Ubian Island, Sulu Archipelago.

**Remarks.** The collection date was not indicated in the original description. The original genus should have been spelled *Cocotropus*.

**(954) *Inimicusbifilis* Fowler, 1938: 85, fig. 38**

= *Inimicusdidactylus* (Pallas, 1769).

**Holotype.**USNM 98905 (57.0), Canmahala Bay, Ragay Gulf, between Burias and Luzon islands, 11 Mar. 1909.

**(955) *Minousradiatus* Matsunuma & Motomura, 2018: 237, figs 22A–C, 23, 25A–C**

**Holotype.** KAUM-I 39286. Off Da-xi, Yilan, Taiwan.

**Paratypes.**USNM 272200 (3, 67.7–79.3), vicinity of Samar and Leyte islands, Visayan Sea, Oct. 1979.

**(956) *Ocosiazaspilota* Poss & Eschmeyer, 1975: 9, figs 2E, 7**

**Holotype.**CAS 33069 (69.5.0), south of Bario Salong, Balayan Bay, Luzon, 229–247 m, 18 Jul. 1966.

**Paratypes.**CAS 33063 (1, 59.0) and CAS 33321 (1, 69.0), same data as holotype; CAS 33320 (1, 46.4), Pagapas Bay, Batangas, Luzon, 192–207 m, 21 Jul. 1966.

**(957) *Prosopodasyscypho* Fowler, 1938: 86, fig. 39**

= *Pseudovespiculacypho* (Fowler, 1938).

**Holotype.**USNM 98902 (45.0), Beach, east of Davao Gulf, Mindanao, 2 m, 16 May 1908.

**(958) *Prosopodasysgogorzae* Jordan & Seale, 1905: 792, fig. 11**

= *Trichosomustrachinoides* (Cuvier, 1829).

**Holotype.**USNM 52054 (29.2), south of Negros Island, 1901.

**Remarks.** Radiographs of the holotype are available in the USNM record.

**(959) *Prosopodasyszonatus* Weber, 1913: 502, pl. 10 (fig. 8)**

= *Paracentropogonzonatus* (Weber, 1913).

**Syntypes.** ZMA 110239 (1), Siboga station 99, north Ubian, Sulu Archipelago, 16–23 m.

**Remarks.** The collection date was not indicated in the original description.

**(960) *Xenaploactisanopta* Poss & Eschmeyer, 1980: 290, figs 3, 4 (upper)**

**Holotype.**CAS 32633 (37.0), 4 km west of Calguaguin Cove, Zambales, Luzon, 64–81 m, 9 Jun 1966.

#### ﻿﻿ORDER ACANTHURIFORMES (91)

##### Family Acanthuridae (501)

**(961) *Acanthurusalbimento* Carpenter, Williams & Santos, 2017: 37, figs 3, 4, 6A**

**Holotype.**PNM 15199 [ex USNM 438093] (252.4), Dinadiawan Market, Aurora, Luzon, 2 Aug. 2015.

**Paratypes.**USNM 438094 (1, 211.2), same data as holotype; USNM 438101 (1, 226.6) and USNM 438102 (1, 213.5), Baler City Market, Aurora, Luzon, 4 Aug. 2015; PNM 15200 (1, 205.), Santa Ana, Cagayan, northern Luzon.

**Remarks.** All type specimens were bought in the fish market, but exact collection localities were unknown.

**(962) *Acanthurusauranticavus* Randall, 1956: 210, figs 2u, 19**

**Holotype.**USNM 136194 (203.0), Atulayan Island, Lagonoy Gulf, Luzon, 2–3 m, 17 Jun 1909.

**Paratypes.**USNM 163619 [ex 136194] (2), same locality as the holotype, 2–3 m, 18 Jun. 1909; CAS-SU 47695 [ex USNM 163619] (2, 179.0–207.0), same data as holotype; USNM 163823 (1, 144.0), Mansalay, southeast of Mindoro Island, 2–5 m, 4 Jun. 1908; BMNH 1956.8.14.1 [ex USNM 136188] (1, 217), Talajit Island, Buang Bay, between Samar and Masbate islands, 15 Mar. 1909.

**Other catalog number.**BMNH 1956.8.14.1 (NHMUK:ecatalogue:2523489).

**Remarks.** The holotype was mixed with six specimens; two paratypes were re-cataloged as USNM 163619 but were exchanged to SU. Two specimens share BMNH 1956.8.14.1 as catalog number based on NMH’s record.

**(963) *Acanthurusleucocheilus* Herre, 1927d: 419, pl. 12 (fig. 3)**

**Syntypes.** BSMP (3, 175–200.0), Bantayan Island, Cebu; BSMP (1, 196.0), Cebu Island; BSMP (1, 186.0), Agutaya Island, Palawan.

**Remarks.** The original description did not indicate the collection date of the holotype. All type specimens are presumed destroyed.

**(964) *Acanthurusmindorensis* Herre, 1927d: 433, pl. 4 (fig. 2)**

**Holotype.** BSMP (170.0), Calapan, Mindoro Island.

**Paratype.** BSMP (1, 184.0), Bantayan Island, Cebu.

**Remarks.** All type specimens were presumed destroyed. A neotype was designated by De la Paz et al. (1988), but probably invalid as it was not done in a revisionary work but only as a checklist (Fricke et al. 2024). Uncertain status (Carpenter et al. 2017).

**(965) *Acanthurusphilippinus* Herre, 1927d: 434, pl. 5 (fig. 1)**

= *Acanthurusthompsoni* (Fowler, 1923).

**Syntypes.** BSMP (9, 103.0–143.0), Calapan, Mindoro Island; Verde Island Passage; Sibuyan Sea; and South China Sea.

**Remarks.** All type specimens are presumed destroyed.

**(966) *Ctenochaetusbinotatus* Randall, 1955: 155, 164, fig. 1 G**

**Holotype.**USNM 136125 (111.0), Pagapas Bay, Luzon, 20 Feb. 1909.

**Paratypes.**CAS-SU 27174 (2, 106.0–121.0), Jolo Island, Sulu Archipelago, 15 Aug. 1931; USNM 136061 (1) and USNM 136103 (1), Endeavor, Malampaya Sound, Taytay, Palawan Island, 3–6 m, 23–24 Dec. 1908; USNM 136064 (2), Little Santa Cruz Island, Zamboanga, Mindanao, 2–9 m, 28 May 1908; USNM 136075 (2), Varadero Bay, Mindoro Island, southern Luzon, 2–5 m, 23 Jul. 1908; USNM 136076 (1), Lagonoy Gulf, near Palah Bay, east of Luzon, 2–8 m, 16 Jun. 1909; USNM 136077 (1), Maricaban Island, Balayan Bay and Verde Island Passage, Luzon, 2–6 m, 20 Jan. 1908; USNM 136078 (1), Camahala Bay, between Burias and Luzon islands, 1–9 m, 11 Mar. 1909; USNM 136079 (1), Caracaran Bay, Batan Island, east of Luzon, 2–3 m, 8 Jun. 1909; USNM 136080 (1), Tutu Bay, Jolo Island, Sulu Archipelago, 1–6 m, 19 Sep. 1909; USNM 136083 (1), Port Galera, Mindoro Island, 2–6 m, 9 Jun 1908; USNM 136095 (4), Sablayan Bay, Mindoro Island, 2–4 m, 12 Dec. 1908; USNM 136099 (1), Lanago Point, extreme southern Luzon, 2–5 m, 24 Jun. 1909; USNM 136102 (1), Pandan Island, Sablayan Bay, Mindoro, 2–3 m, 13 Dec. 1908; USNM 136104 (1), Port Matalvi, off west of Luzon, 2–9 m, 23 Nov. 1908; USNM 136105 (1), Port Busin, Burias Island, Masbate, 3–6 m, 8 Mar. 1909; USNM 136109 (1), Pantocomi Point, Paluan Bay, Mindoro Island, 3–6 m, 11 Dec. 1908; USNM 136111 (1), Destacado Island, Lode Bay, between Samar and Masbate islands, 5 m, 13 Mar. 1909; USNM 136073 (1) and USNM 136114 (1), Port Maricaban, southern Luzon, 4–6 m, 21 Jul. 1908; USNM 136116 (1), Macajalar Bay, northern Mindanao, 2–4 m, 4 Aug. 1909; USNM 136119 (1), Grande Island reef, Subig Bay, off southern Luzon, 2–6 m, 8 Jan. 1908.

**Remarks.** The paratypes have sizes between 79.0–141.0. Radiographs of the holotype are available in the USNM record.

**(967) *Nasocaeruleacauda* Randall, 1994: 122, fig. 3; pls 1C, 2A**

**Holotype.**CAS-SU 13663 (254.0), Dumaguete, Negros Island, 1931.

**Paratype.** BPBM 35379 [ex CAS-SU 13663] (1, 191.0), Manila, Luzon, 26 Aug. 1931.

**Remarks.** A photograph and a radiograph of the holotype are available in the CAS record.

**(968) *Nasofageni* Morrow, 1954: 799**

**Holotype.**USNM 122107 (571.0), Bugsuk Island, Balabac, Palawan, 3–5 m, 5 Jan. 1909.

**Paratypes.**USNM 122106 (1), same data as holotype; USNM 122108 (1), Zamboanga Market, Mindanao, 25 May 1908; YPM (1) (245.0–571.0).

**Remarks.**Morrow (1954) noted that the type specimen was found to be identical to three specimens described by Fowler and Bean (1929) as *Nasotapeinosoma* (Bleeker), who also mentioned differences of some important aspects.

**(969) *Nasolopezi* Herre, 1927d: 467, pl. 7 (fig. 2)**

**Holotype.** BSMP 14084, Ambil Island, southwest of Manila Bay, Luzon.

**Paratype.** BSMP (1).

**Remarks.** The original description did not indicate the collection date of the type specimens. All type specimens are presumed destroyed.

#### ﻿﻿ORDER SPARIFORMES (82)

##### Family Nemipteridae (505)

**(970) *Dentexfiliformis* Seale, 1910: 512, pl. 9**

= *Pentapodussetosus* (Valenciennes, 1830).

**Holotype.** BSMP 1755 (130.0), Surigao, Mindanao, 17 Sep. 1907.

**Remarks.** The type specimen was presumed destroyed.

**(971) *Nemipterusworcesteri* Evermann & Seale, 1907: 81, fig. 14**

= *Nemipterusfurcosus* (Valenciennes, 1830).

**Holotype.**USNM 55917 (215.9), Bacon, Sorsogon, Luzon.

**Remarks.** The original description or USNM record did not indicate the collection date.

**(972) *Parascolopsistanyactis* Russell, 1986: 140, fig. 3**

**Holotype.** WAM P.26263-001. Larrey Point, North West Shelf, Western Australia.

**Paratype.** NTM S. 10781-001 (3, 77.0–103.3), 11°23.63'N, 123°54.00'E, Caduruan Point, Visayan Sea, between northern Negros and Masbate islands, 98.7 m, 7 Jun. 1978; USNM 231473 (1 male, 154.0), 11°37.12'N, 123°54.75'E, Caduruan Point, Visayan Sea, between northern Negros and Masbate islands, 91.4 m, 6 Jun. 1978; USNM 231480 (1 male, 144.7), 11°27.00'N–12°27.00'N, 124°25.10'E–124°47.60'E, Carigara Bay, Samar Sea, Apr.–May 1980.

**(973) *Pentapoduslineoscapularis* Fowler, 1943: 63, fig. 10**

= *Pentapodusbifasciatus* (Bleeker, 1848).

**Holotype.**USNM 108465 (135.0), Iloilo, Panay Island, 9 May 1929.

**(974) *Scolopsisbulanensis* Evermann & Seale, 1907: 85, fig. 5**

= *Scolopsisxenochrous* Günther, 1872.

**Holotype.**USNM 55909 (108.0), Bulan, Sorsogon, Luzon.

**Remarks.** The original description or USNM record did not indicate the collection date.

**(975) *Scolopsisluzonia* Jordan & Seale, 1907: 22, fig. 8**

= *Scolopsisciliata* (Lacepède 1802).

**Holotype.**CAS-SU 9243 (81.3), Cavite, Luzon, 1900.

**Remarks.** A photograph and a radiograph of the holotype are available in the CAS record.

##### Family Lethrinidae (506)

**(976) *Lethrinusatkinsoni* Seale, 1910: 515, pl. 11**

**Holotype.** BSMP 5080 (220.0), Balabac Island, Palawan, 6 Aug. 1908.

**Remarks.** The type specimen was presumed destroyed.

**(977) *Lethrinuscutambi* Seale, 1910: 514, pl. 10**

= *Lethrinusobsoletus* (Forsskål, 1775).

**Holotype.** BSMP 4678 (210.0), Sitanki Island, Sulu Archipelago, 1 Jul. 1908.

**Paratype.** BSMP 4680 (1).

**Remarks.** All type specimens were presumed destroyed.

**(978) *Lethrinusjagorii* Peters, 1868: 257**

**Holotype.** ZMB 6302 (50.0), Paracali, Luzon.

**Remarks.** Uncertain status (Carpenter and Allen 1989). The type specimen was in poor condition. The collection date was not indicated in the original description.

##### Family Sparidae (507)

**(979) *Dentexelongatus* Marion de Procé, 1822: 132**

Manila Bay, Luzon.

**Remarks.** No type known. *Incertae sedis* in Sparidae (Parenti 2019). Collection of specimens and further study is needed to confirm its status.

#### ﻿﻿ORDER LOPHIIFORMES (84)

##### Family Lophiidae (509)

**(980) *Lophiodesinfrabrunneus* Smith & Radcliffe in Radcliffe, 1912c: 202, pl. 16 (fig. 3), fig. 2**

= *Lophiodestriradiatus* (Lloyd, 1909).

**Holotype.**USNM 70265 (370.0), Albatross station 5488 (10°00.00'N, 125°06.75'E), between Leyte and Mindanao islands, 1412 m, 31 Jul. 1909.

**Paratypes.**USNM 122281 (1), Albatross station 5410, Bagacay Point Lighthouse, between Cebu and Leyte islands, 704 m, 18 Mar. 1909; USNM 122282 (1), Albatross station 5219, Mompog Island, between Marinduque and Luzon islands, 969 m, 23 Apr. 1908; USNM 122283 (2), Albatross station 5373, Tayabas Lighthouse, Marinduque Island, 618 m, 2 Mar 1909; USNM 122286 (1) and USNM 122284 (1), Albatross stations 5508 and 5511, Camp Overton Lighthouse, northern Mindanao, 494–750 m, 5–7 Aug. 1909; USNM 122285 (1) and USNM 150918 (1), Albatross stations 5406-07, Ponson Island, Dupon Bay, Leyte, 545–640 m, 17 Mar. 1909.

**Remarks.** Radiographs and photographs of the holotype are available in the USNM record.

**(981) *Lophiodesolivaceus* Smith & Radcliffe in Radcliffe, 1912c: 200, pl. 16 (fig. 2), fig. 1**

= *Lophiodesmutilus* (Alcock, 1894).

**Holotype.**USNM 70264 (290.0), Albatross station 5505 (08°37.25'N, 124°36.00'E), Macabalan Point Lighthouse, northern Mindanao, 402 m, 5 Aug. 1909.

**Remarks.** Radiographs and photographs of the holotype are available in the USNM record.

##### Family Antennariidae (510)

**(982) *Antennariusaltipinnis* Smith & Radcliffe in Radcliffe, 1912c: 204, fig. 3**

= *Abantennariusdorehensis* (Bleeker, 1859).

**Holotype.**USNM 70267 (21.0), Nogas Point, Panay Island, 4 Dec. 1908.

**(983) *Antennariusrosaceus* Smith & Radcliffe in Radcliffe, 1912c: 203, pl. 17 (fig. 2)**

= *Abantennariusrosaceus* (Smith & Radcliffe, 1912).

**Holotype.**USNM 70266 (38.0), Romblon Island, 37 m, 25 Mar. 1908.

**(984) *Antennariussubteres* Smith & Radcliffe in Radcliffe, 1912c: 205, pl. 17**

= *Nudiantennariussubteres* (Smith & Radcliffe, 1912).

**Holotype.**USNM 70268 (56.0), Albatross station 5442 (16°30.60'N, 120°11.10'E), San Fernando Point Lighthouse, Lingayen Gulf, west of Luzon, 82 m, 10–11 May 1909.

**Remarks.** The collection date was not indicated in the original description.

**(985) *Phryneloxlochites* Schultz, 1964: 179, pl. 1**

= *Antennariusstriatus* (Shaw, 1794).

**Holotype.**CAS-SU 38194 (48.5), Dapitan Bay, northern Mindanao, Aug. 1940.

**Paratypes.**CAS-SU 60459 (2, 33.5–40.0) and USNM 197325 (1, 39.0), same locality as holotype.

**Remarks.** A photograph and a radiograph of the holotype are available in the CAS record.

##### Family Chaunacidae (514)

**(986) *Chaunaxalbatrossae* Ho & Ma, 2022: 150, figs 5, 6A**

**Holotype.**MNHN 2005-0517 (77.3), ca. 13°38.00'N, 121°39.00'E, Tayabas Bay, off southern Luzon, 195–200 m, 1 Dec. 1973.

**Paratypes.**MNHN 2005-0608 (2, 59.3–93.5) and MNHN 2005-0876 (3, 44.9–119.2), Musorstom 3, Tablas Strait, between Mindoro and Tablas islands, 673–702 m, 4 Jun. 1985; USNM 168872 (1, 113.7), Albatross station 5515, Camp Overton Lighthouse, Iligan Bay, northern Mindanao, 1280 m, 8 Aug. 1909; USNM 168883 (1, 137.7), Albatross station 5488, San Ricardo Point, between Leyte and Mindanao islands, 1412 m, 31 Jul. 1909.

**Remarks.**MNHN record indicates that the holotype was collected by Musorstom 3 on 5 Jun. 1985. Photographs of the holotype are available in the MNHN record.

**(987) *Chaunaxbreviradius* Le Danois, 1978: 91, figs 3, 4**

**Holotype.**MNHN 1977-0762 (110.0), Musorstom 1 station 6 (14°01.02'N, 120°19.98'E, off Lubang Island, west of Luzon, 182–200 m, 19 Mar. 1976.

**Paratypes.**MNHN 1977-0763 (1, 90.0) Musorstom 1 station 2, 182–187 m, 19 Mar. 1976; MNHN 1977-1100 (1, 29.6), Musorstom 1 station 24, 189–209 m, 22 Mar. 1976; MNHN 1977-0764 (1, 72.3), Musorstom 1 station 9, 180–194 m, 19 Mar. 1976; MNHN 1977-1101 (1, 33.5), Musorstom 1 station 27, 188–192 m; all have the same locality as holotype; MNHN 1977-0765 (3, 29.7–40.4), Musorstom 1 station 50, off west of Lubang Island, Luzon, 415–510 m, 25 Mar. 1976.

**(988) *Chaunaxgomoni* Ho, Kawai & Satria, 2015: 302, figs 1A, B, 2A, B**

**Holotype.** HUMZ 193991. Off Java, Indonesia.

**Paratypes.**USNM 168256 (1, 138.0), Albatross station 5117, Sombrero Island, Balayan Bay and Verde Island Passage, Luzon, 216 m, 21 Jan. 1908; CAS 34598 (1, 121.0), east of Dilao Point, Batangas Bay, Luzon, 294–329 m, 1 Aug. 1966.

##### Family Ogcocephalidae (515)

**(989) *Coelophrysarca* Smith & Radcliffe in Radcliffe, 1912c: 213, pls 24 (fig. 2), 26 (fig. 4), 27 (fig. 4)**

**Holotype.**USNM 70276 (41.0), Albatross station 5295 (13°33.25'N, 121°00.00'E), Escarceo Lighthouse, Verde Island Passage, Luzon, 422 m, 24 Jul. 1908.

**(990) *Coelophrysbrevipes* Smith & Radcliffe in Radcliffe, 1912c: 213, pls 25 (fig. 2), 26 (fig. 2), 27 (fig. 2)**

**Holotype.**USNM 70277. Cape Loko Loko, Gulf of Boni, Sulawesi, Indonesia.

**Paratype.**USNM 169021 (1), Albatross station 5238, Lambajon Point, east coast of Mindanao, 695 m, 12 May 1908.

**(991) *Coelophrysmollis* Smith & Radcliffe in Radcliffe, 1912c: 212, pls 24 (fig. 1), 26 (fig. 5), 27 (fig. 5)**

**Holotype.**USNM 70275 (39.0), Albatross station 5348 (10°57.75'N, 118°38.25'E), Palawan Passage, 686 m, 27 Dec. 1908.

**Remarks.** The type specimen is in bad condition, almost totally destroyed (Smithsonian Museum Archive 2024).

**(992) *Dibranchussimulus* Smith & Radcliffe in Radcliffe, 1912c: 211, pls 22 (fig. 1), 23 (fig. 1)**

= *Halieutopsissimula* (Smith & Radcliffe, 1912).

**Holotype.**USNM 70274 (86.0), Albatross station 5283 (13°48.50'N, 120°28.67'E), near Malavatuan Island, southern Luzon, 512 m, 18 Jul. 1908.

**(993) *Halicmetusreticulatus* Smith & Radcliffe in Radcliffe, 1912c: 208, pls 20 (fig. 2), 21 (fig. 2)**

**Holotype.**USNM 70271 (78.0), Albatross station 5118 (13°48.75'N, 120°41.85'E), off Sombrero Island, Balayan Bay and Verde Island Passage, southern Luzon, 291 m, 21 Jan. 1908.

**Paratypes.** Uncat. (4, 54.0–78.0), 291–366 m.

**Remarks.** The original description did not provide catalog numbers. A radiograph of the holotype is available in the USNM record. Three specimens (USNM 150902–03) collected from Tagolo Point Lighthouse, northern Mindanao, and Capitancillo Island Lighthouse, Cebu were noted in the USNM record. They had similar collection depths to the paratypes mentioned in the original description. However, it was not indicated whether they are paratypes.

**(994) *Halieutopsisvermicularis* Smith & Radcliffe in Radcliffe, 1912c: 209, pls 20 (fig. 1), 21 (fig. 1)**

= *Halieutopsisnasuta* (Alcock, 1891).

**Holotype.**USNM 70272 (80.0), Albatross station 5660 (13°44.40'N, 120°45.50'E), Cape Santiago Lighthouse. Balayan Bay, Luzon, 391 m, 22 Feb. 1909.

**(995) *Malthopsisarrietty* Ho, 2020: 861, figs 1–3**

**Holotype.**USNM 169204 (45.4), Albatross station 5113 (13°51.50'N, 120°50.52'E), Sombrero Island, Balayan Bay, Luzon, 291 m, 17 Jan. 1908.

**Paratypes.**CAS 34261 (1, 41.0), Bantuin Point, Ragay Gulf, Luzon, 534–543.2 m, 12 Nov. 1966; CAS 227265 (1, 44.2), Tayabas Bay, Marinduque Island, 234–256 m, 2 Oct. 1966; MNHN 2005-0666 (10, 31.6–48.5), Sibuyan Sea, among Semirara, Caluya and Sibay islands, Luzon, 388–404 m, 4 Jun. 1985; NSMT-P 96969 (2, 44.7–45.8), Sulu Sea, off Panay Island, 362–372 m, 8 Dec. 2002; USNM 169215 (2, 39.8–44.2), Albatross station 5503, Macajalar Bay, Mindanao, 413 m, 4 Aug. 1909; USNM 122371 (15, 34.4–45.1), same locality as USNM 169215, 402 m, 5 Aug. 1909; USNM 122372 (12, 25.9–44.6), USNM 168864 (17, 26.1–44.9) and NMMB-P 34201 (4, 34.1–43.0), Albatross station 5501, same locality as USNM 169215, 391 m, 4 Aug. 1909; USNM 150855 (10, 27.3–38.3), Albatross station 5506, same locality as USNM 169215, 479 m, 5 Aug 1909; USNM 169203 (1, 46.2), Albatross station 5112, Balayan Bay, Luzon, 324–326 m, 17 Jan. 1908; USNM 169207 (1, 39.3), Albatross station 5371, Tayabas Bay, Luzon, 152 m, 24 Feb. 1909; USNM 169209 (1, 41.8), Albatross station 5402, Camotes Sea, between Leyte and Cebu islands, 0–344 m, 16 Mar. 1909; USNM 169210 (8, 29.1–43.5), same locality as USNM 169209, 0–346 m, 18 Mar. 1909; USNM 169212 (1, 30.5), Albatross station 5507, Iligan Bay, northern Mindanao, Aug. 1909; USNM 169213 (1, 54.0), Albatross station 5518, Bohol Sea, Mindanao, 366 m, 9 Aug. 1909; USNM 169214 (1, 48.2), same locality and depth as SNM 169213, 20 Aug. 1909.

**Remarks.** The USNM records did not indicate USNM 169204 as holotype.

**(996) *Malthopsisocellata* Smith & Radcliffe in Radcliffe, 1912c: 207, pls 18 (fig. 1), 19 (fig. 1)**

= *Malthopsisannulifera* Tanaka, 1908.

**Holotype.**USNM 70270 (98.0), Albatross station 5393 (12°03.50'N, 124°03.60'E), between Samar and Masbate islands, 249, 13 Mar. 1909.

**Paratypes.** Uncat.

**Remarks.** The original description mentioned that this is the most abundant species in their collection, comprised of adult and young specimens taken between 69 and 777 m depth. However, it was not mentioned if these specimens were considered as type specimens. The USNM record showed five lots with eight specimens collected from different locations in the Philippines between 1908 and 1909. These were collected using a beam trawl similar to the collection method of the holotype, but were not indicated if these are paratypes of *M.ocellata*.

##### Family Oneirodidae (521)

**(997) *Chirophrynexenolophus* Regan & Trewavas, 1932: 82, figs 131, 132**

**Holotype.** ZMUC P9296, Dana station 3731 (14°37.00'N, 119°52.00'E), off western Luzon, 1250 m, 17 Jun. 1929.

**(998) *Dermatiasplatynogaster* Smith & Radcliffe in Radcliffe, 1912c: 206, pl. 17 (fig. 3)**

**Holotype.**USNM 70269 (182.0), Albatross station 5463 (13°40.95'N, 123°57.75'E), near Sialat Point Lighthouse, east of Luzon, 549 m, 16 Jun. 1909.

**Remarks.** Radiographs and photographs of the holotype are available in the USNM record.

##### Family Histiophrynidae

**(999) *Antennariuslithinostomus* Jordan & Richardson, 1908: 286, fig. 12**

= *Lophiocharonlithinostomus* (Jordan & Richardson, 1908).

**Holotype.**CAS-SU 20204 (101.6), Cuyo Island, Palawan.

**Remarks.** The original description or CAS record did not indicate the collection date. A photograph and a radiograph of the holotype are available in the CAS record.

#### ﻿﻿ORDER TETRAODONTIFORMES (85)

##### Family Triacanthodidae (528)

**(1000) *Halimochirurgusmacraulos* Fowler, 1934: 367, fig. 117**

= *Halimochirurgusalcocki* Weber, 1913.

**Holotype.**USNM 93169 (193.0 TL), Albatross station 5517 (08°45.50'N, 123°33.75'E), Tagolo Point Lighthouse, northern Mindanao, 309 m, 9 Aug. 1909.

**Paratypes.**USNM 93475 (2), same data as the holotype, 333 m; USNM 93474 (1, 123.5), Albatross station 5441, San Fernando Point Lighthouse, west of Luzon, 340 m, 10 May 1909; USNM 93476 (1), Albatross station 5291, Escarceo Lighthouse, southern Luzon, 416 m, 23 Jul. 1908; USNM 93477 (2), Albatross station 5273, Corregidor Lighthouse, southern Luzon, 208 m, 14 Jul. 1908.

**Remarks.** Radiographs of the holotype are available in the USNM record.

**(1001) *Halimochirurgustriacanthus* Fowler, 1934: 365, fig. 116**

= *Halimochirurgusalcocki* Weber, 1913.

**Holotype.**USNM 93168 (115.0 TL), Albatross station 5110 (13°59.33'N, 120°75.75'E), Corregidor Lighthouse, off southern Luzon, 247 m, 15 Jan. 1908.

**Paratype.**USNM 98829 (1), Albatross station 5112, Sombrero Island, off southern Luzon, 324–326 m, 17 Jan. 1908.

**Remarks.** Radiographs of the holotype are available in the USNM record.

**(1002) *Macrorhamphosodesplatycheilus* Fowler, 1934: 365, fig. 115**

**Holotype.**USNM 93170 (113.0 TL), Albatross station 5118, Sombrero Island, Balayan Bay and Verde Island Passage, Luzon, 291 m, 21 Jan. 1908.

**Paratypes.**USNM 93478 (1), same data as holotype; USNM 93480 (6), USNM 93482 (4), Albatross stations 5516-17, Tagolo Point Lighthouse, northern Mindanao 309–320 m, 9 Aug. 1909; CAS-SU 40195 (3), off Tagolo Point Lighthouse, northern Mindanao, 320 m, 9 Aug. 1909; USNM 93479 (1), Albatross station 5403, Capitancillo Island Lighthouse, between Leyte and Cebu islands, 333 m, 16 Mar. 1909; USNM 93481 (1), Albatross station 5112, Sombrero Island, off southern Luzon, 324–326 m, 17 Jan. 1908; USNM 93483 (1), Albatross station 5525, Balicasag Island, between Siquijor and Bohol islands, 741 m, 11 Aug. 1909; USNM 93484 (1), Albatross station 5549, Jolo Lighthouse, Sulu Archipelago, 481 m, 17 Sep. 1909.

**Remarks.** A radiograph of the holotype is available in the USNM record.

**(1003) *Paratriacanthodesherrei* Myers, 1934: 9**

**Holotype.**USNM 93293 (73.0), Albatross station 5519 (08°47.00'N, 123°31.25'E), off Tagolo Point Lighthouse, northern Mindanao, 333 m, 9 Aug. 1909.

**Paratypes.**USNM 93302 (2), same data as holotype.

**Remarks.** All type specimens were original type series of *P.retrospinnis* which contained two species. Radiographs of the holotype are available in the USNM record.

##### Family Balistidae (532)

**(1004) *Balisteshumilis* Herre, 1924c: 434**

= *Sufflamenchrysopterum* (Bloch & Schneider, 1801).

**Holotype.** BSMP 10252, south of Cotabato, Mindanao, Mar. 1923.

**Paratypes.** BSMP (31, 25.0–32.0), taken with holotype.

**Remarks.** All type specimens are presumed destroyed.

**(1005) *Balistesrotundatus* Marion de Procé, 1822: 130**

= *Canthidermisrotundata* (Marion de Procé, 1822).

Manila, Luzon.

**Remarks.** No type known.

**(1006) *Canthidermisviola* Herre, 1926b: 534, pl. 1**

= *Canthidermismaculata* (Bloch, 1786).

**Holotype.** BSMP (320.0), Manukan, Cagayancillo Island, Palawan, 22 Sep. 1925.

**Remarks.** The type specimen was presumed destroyed.

##### Family Monacanthidae (533)

**(1007) *Cantherhinescerinus* Randall, 2011: 7, pl. IA, B**

**Holotype.** BPBM 36836, offshore reef, Bolinao, Luzon, 8 Oct. 1995.

**Paratype.** FMNH 119627 (1), Tara Island, Coron, Palawan, 22–25 m, 4 Mar. 2003; USNM 399289 (1).

**(1008) *Monacanthustessellatus* Günther, 1880: 54, pl. 23 (fig. B)**

= *Thamnaconustessellatus* (Günther, 1880).

**Holotype.**BMNH 1879.5.14.390 (127.0), Challenger Station 204, northwest Tablas Island, Romblon, 210 m, 2 Nov. 1874.

**Other catalog number.** NHMUK:ecatalogue:3108726.

**(1009) *Stephanolepisnigrolineatus* Herre, 1927a: 415**

= *Pervagornigrolineatus* (Herre, 1927).

**Syntypes.** BSMP (2, 48.0–49.0), the reef at Bungau, Sulu Archipelago.

**Remarks.** The original description did not indicate the collection date. All type specimens are presumed destroyed.

**(1010) *Stephanolepisretrospinis* Fowler, 1943: 90**

**Holotype.**USNM 108467 (51.0), Cebu Island, 24 Apr 1929.

**Remarks.** Questionably a synonym of *Paramonacanthuschoirocephalus* (Bleeker, 1851) (Hutchins 1997). Radiographs of the holotype are available in the USNM record.

**(1011) *Tetraodoncompressus* Marion de Procé, 1822: 130**

= *Canthigastercompressa* (Marion de Procé, 1822).

Manila, Luzon.

**Remarks.** No type known.

##### Family Tetraodontidae (535)

**(1012) *Canthigasterleoparda* Lubbock & Allen, 1979: 88, figs 1–2**

**Holotype.**BMNH 1979.1.9.41, caves on vertical drop-off, 0.5 km north of Caubyan Daku Island, Camotes Sea, 35–40 m, 15 Aug. 1976.

**Paratype.**BMNH 1979.1.9.42 (1), same data as holotype.

**Other catalog number.**BMNH 1979.1.9.41 (NHMUK:ecatalogue:2545207), BMNH 1979.1.9.42 (NHMUK:ecatalogue:2545208).

**(1013) *Tetraodonmanilensis* Marion de Procé, 1822: 130**

= *Arothronmanilensis* (Marion de Procé, 1822).

Manila, Luzon.

**Remarks.** No type known.

#### ﻿﻿ORDER ACROPOMATIFORMES

##### Family Acropomatidae (399)

**(1014) *Acropomaboholensis* Yamanoue & Matsuura, 2002: 21, figs 1, 2A, 3A, 4**

= *Acropomaboholense* Yamanoue & Matsuura, 2002.

**Holotype.**USNM 232332 (male, 132.0), Dumaguete Market, Negros Island, 27 Mar. 1976.

**Paratype.**USNM 362367 [ex USNM 232332] (1 female, 132.0), same data as holotype.

**Remarks.**USNM 362367 is partially dissected (Smithsonian Museum Archive 2024).

**(1015) *Rhomboserranusgracilispinis* Fowler, 1943: 59, fig. 8**

= *Doederleiniaberycoides* (Hilgendorf, 1879).

**Holotype.**USNM 99519 (80.0), Albatross station 5273 (13°58.75'N, 120°21.58'E), off Corregidor Lighthouse, southern Luzon, 209 m, 14 Jul. 1908.

**Paratypes.**USNM 99520 (6, 41.0–61.0), same data as holotype; USNM 99521 (9, 49.0–65.0), Albatross station 5266, Matocot Point, Verde Island Passage and Batangas Bay, Luzon, 183 m, 8 Jun. 1908.

**Remarks.** Originally, USNM 99520 contained only five specimens. Radiographs of the holotype are available in the USNM record.

##### Family Hemerocoetidae

**(1016) *Pteropsarondabfar* Iwamoto, 2014: 252, figs 1–3**

**Holotype.**CAS 236400 (33.6), 13°54.35'N, 120°21.26'E, between Luzon and Mindoro islands, 82–86 m, 2 Jun. 2011.

**Paratypes.**CAS 236560 (1, 33.0) and CAS 236667 (1, 30.0), taken with holotype.

**(1017) *Pteropsaronlevitoni* Iwamoto, 2014: 255, figs 4–6**

**Holotype.**CAS 236401 (23.), 13°54.35'N, 120°21.26'E, between Luzon and Mindoro islands, 82–86 m, 2 Jun. 2011.

**Paratypes.**CAS 236399 (6, 19.8–27.9), same data as holotype.

**(1018) *Pteropsaronspringeri* Smith & Johnson, 2007: 365, figs 1B, 2–5**

**Holotype.**USNM 367912 (male, 27.5), 09°36.97'N, 123°10.08'E, east of Bais, Negros Island, 0–37 m, 17 Jun. 1978.

**Paratypes.** AMS I.21918-036 (female, 26.5), Caban Island, Verde Island Passage, Luzon, 1980; USNM 288848 (male, 25.7), the mouth of Bais Bay, Negros Island, 30–35 m, 17 May 1987; USNM 288849 (male, 29.1), Bais Bay, Negros Island 24–37 m, 15 May 1987; USNM 288850 (8 females + 2 males), USNM 368827 (1), north Bais Bay near main channel, Cebu Strait, Negros Island, 18–35 m, 19 May 1987.

**(1019) *Roxasellafusiforme* Fowler, 1943: 87, fig. 23**

= *Matsubaraeafusiformis* (Fowler, 1943).

**Holotype.**USNM 99517 (63.0), Aparri, Cagayan, Luzon, 19 Nov. 1908.

**Paratype.**USNM 99518 (1, 44.0), same data as holotype.

**Remarks.** Radiographs of the holotype are available in the USNM record.

##### Family Malakichthyidae

**(1020) *Malakichthyssimilis* Yamanoue & Matsuura, 2004: 524, figs 1F, 9**

**Holotype.**USNM 184144 (150.0), Albatross station 5501 (08°37.60'N, 124°35.00'E), north of Macabalan Point Lighthouse, Mindanao, 391 m, 4 Aug. 1909.

**Paratypes.**USNM 367114 [ex USNM 184144] (10, 28.0–147.0), taken with the holotype.

**Remarks.** The holotype was originally mixed with paratypes and 10 specimens were re-cataloged to USNM 367114.

##### Family Synagropidae

**(1021) *Acropomaphilippinense* Günther, 1880: 51**

= *Parascombropsphilippinensis* (Günther, 1880).

**Lectotype.**BMNH 1879.5.14.167 (70.0), West of Zamboanga, Mindanao, 150–187 m.

**Paralectotypes.**BMNH 2016.9.14.1–4 (4) [ex BMNH 1879.5.14.167], west of Zamboanga, Mindanao, 187 m, 26 Oct. 1874.

**Other catalog numbers.**BMNH 1879.5.14.167 (NHMUK:ecatalogue:3108528), BMNH 2016.9.14.1–4 (NHMUK:ecatalogue:6750927).

**(1022) *Parascombropsglossodon* Schwarzhans & Prokofiev, 2017: 33, figs 7C, 8F, 9C, 12A, 12D, 13C, 14J–N, 18**

**Holotype.**USNM 436687 [ex USNM 99330] (70.0), Albatross station 5517 (08°45.00'N, 123°33.00'E), Tagolo Point Lighthouse, northern Mindanao, 309 m, 9 Aug. 1909.

**Paratypes.**USNM 438230 [ex USNM 438230] (2, 61.5–64.5), same data as holotype; USNM 99335 (1, 64.5), Albatross station 5121, Malabrigo Lighthouse, east coast of Mindoro Island, 198 m, 2 Feb. 1908; USNM 436684 [ex 99321] (1, 73.0), Albatross station 5117, Sombrero Island, between Balayan Bay and Verde Island Passage, Luzon, 216 m, 21 Jan. 1908; USNM 436685 [ex 99322] (7, 55.5–64.0), Albatross station 5273, Corregidor Lighthouse, off southern Luzon, 208 m, 14 Jul. 1908; USNM 436686 [ex 99329] (3, 69.5–77.0), Tagolo Point Lighthouse, northern Mindanao and vicinity, 320 m, 9 Aug. 1909.

**Remarks.** Three specimens originally assigned to USM 99330 are considered as type specimens. One specimen was re-cataloged to USNM 436687 and designated as the holotype, while two specimens were re-cataloged to USNM 438230 which became paratypes.

**(1023) *Parascombropsnakayamai* Schwarzhans & Prokofiev, 2017: 39, figs 7F, 8C, 9F, 12B, 13F, 14O–R, 21, 27**

**Holotype.** BSKU 102255. Tosa Bay, Kochi, Japan.

**Paratypes.**CAS 34870 (1, 81.5), off Balayan Bay, Luzon, 214–247 m, 5 Aug. 1966; CAS 236019 (1, 84.0), Verde Island Passage, Luzon, 216261 m, 4 Jun. 2011; CAS 238017 (4, 76.0–84.0), eastern Luzon, 435–451 m, 27 Sep. 1995; MNHN 2005-0934 (2, 58.0–104.0), Musorstom 3, north of Cebu Island, 7 Jun. 1985; USNM 99303 (1, 106.0), Albatross station 5503, Macabalan Point Lighthouse, northern Mindanao and vicinity, 413 m, 4 Aug. 1909; USNM 99315 (2, 88.0–110.0), Albatross station 5403, Capitancillo Island Lighthouse, between Leyte and Cebu islands, 333 m, 16 Mar. 1909.

**(1024) *Parascombropsparvidens* Schwarzhans & Prokofiev, 2017: 43, figs 7H, 8B, 9G, 11C, 13H, 14V–Y, 23**

**Holotype.** BSKU 98886. Sulawesi, Indonesia.

**Paratypes.** BSKU 15219–21 (3, 69.0–75.0), 08°14.0'N, 119°59.0'E, middle of Sulu Sea (near Tubbataha Reef Natural Park), Palawan; USNM 436686 [ex 99329] (3, 56.0–65.0), Albatross station 5516, Tagolo Point Ligthouse, northern Mindanao and vicinity, 320 m, 9 Aug. 1909.

**(1025) *Synagropsserratospinosa* Smith & Radcliffe in Radcliffe, 1912b: 444, pl. 38 (fig. 2)**

= *Parascombropsserratospinosus* (Smith & Radcliffe, 1912).

**Holotype.**USNM 70254 (female, 81.0), Albatross station 5365 (13°44.40'N, 120°45.50'E), Cape Santiago Lighthouse, Balayan Bay, Luzon, 391 m, 22 Feb. 1909.

**Paratypes.**USNM 99339 (9), USNM 99345 (16), USNM 99354 (3), USNM 99349 (3), USNM 99337 (12) and USNM 99339 (9), Macabalan Point Lighthouse, northern Mindanao, 366–413 m, 4 Aug. 1909; USNM 99338 (2), USNM 99340 (3) and USNM 99342 (3), Tagolo Point Lighthouse, northern Mindanao, 309 m, 9–10 Aug. 1909; USNM 99343 (2), Escarceo Lighthouse, southern Luzon, 296, 23 Jul. 1908; USNM 99344 (11), USNM 99356 (2), USNM 99357 (4), USNM 99352 (2) and USNM 99347 (1), Lauis Point Lighthouse, between Cebu and Bohol islands, 265–302 m, 23 Mar. 1909; USNM 99346 (2), USNM 99348 (3), USNM 99359 (2) and USNM 99336 (4), Apo Island, between Negros and Siquijor islands, 465–510 m, 19 Aug. 1909; USNM 99351 (2), Lusaran Lighthouse, between Panay and Negros islands, 176 m, 30 Mar. 1908; USNM 99350 (1) and USNM 99355 (1), Matocot Point, Verde Island Passage and Batangas Bay, Luzon, 247 m, 6 Jun. 1908; USNM 99353 (1), Cape Santiago Lighthouse, Balayan Bay, Luzon, 391 m, 22 Feb. 1909; USNM 99358 (4), USNM 99360 (2), Bagatao Island Lighthouse, between Burias and Luzon islands, 382 m, 11 Mar. 1909; USNM 99361 (1) and USNM 99341 (61), Corregidor Lighthouse, southern Luzon, 208–247 m, 14–15 Jul. 1908; USNM 367313 (1), Tayabas Lighthouse, Marinduque Island, 194 m, 24 Feb. 1909.

**Remarks.** The original description mentioned Batangas Bay as the holotype locality but USNM records it was in Balayan Bay. Radiographs of the holotype are available in the USNM record.

#### ﻿﻿ORDER CENTRARCHIFORMES

##### Family Terapontidae (413)

**(1026) *Datniaplumbea* Kner, 1864: 484 [4**]

= *Leiopotheraponplumbeus* (Kner, 1864).

**Syntypes.** NMW 38325 (3) and NWM 76470 (1), Philippines.

**Remarks.** The collection dates were not indicated in the original description. The original description erroneously indicated Cape of Good Hope from St. Paul Island as the type locality. However, Vari (1978) indicated that the type locality is uncertain, while Kottelat (2013) considered it a Philippine endemic.

**(1027) *Theraponbrevispinnis* Peters, 1868: 256**

= *Leiopotheraponplumbeus* (Kner, 1864).

**Syntypes.** ZMB 6681 (6), Quingoa River, Bulacan, Luzon.

**Remarks.** The collection date was not indicated in the original description.

##### Family Cirrhitidae (439)

**(1028) *Cirrhitichthysanalis* Fowler, 1938: 48, fig. 18**

= *Cirrhitichthysaprinus* (Cuvier, 1829).

**Holotype.**USNM 98901 (69.0), Albatross station 5145 (06°04’.50'N, 120°59.50'E), near Jolo Lighthouse, Sulu Archipelago, 42 m, 15 Feb. 1908.

**(1029) *Cirrhitichthysfalco* Randall, 1963: 435, pl. 13 (fig. 28)**

**Holotype.** AMNH 20412 (female, 41.8), Davao Gulf, Mindanao, 1937.

**Paratypes.** AMNH 20413 (1, 25.2) and USNM 195954 (1, 32.2), same data as holotype.

**(1030) *Oxycirrhitesmorrisi* Fowler, 1934: 359, fig. 109**

= *Oxycirrhitestypus* Bleeker, 1857.

**Holotype.**USNM 93172 (86.0 TL), Albatross station 5432, Corandagos Island, eastern Palawan, 93 m, 8 Apr. 1909.

**Remarks.** The original description mentioned USNM 93170 as the holotype. However, USNM record indicate USNM 93172 as the holotype (Smithsonian Museum Archive 2024).

#### ﻿﻿ORDER CHAETODONTIFORMES

##### Family Leiognathidae (431)

**(1031) *Gazzarhombea* Kimura, Yamashita & Iwatsuki, 2000: 2, fig. 1**

**Holotype.** NSMT-P 55508. Fumauki Bay, Iriomote Island, Okinawa, Japan.

**Paratypes.** FMNH 105070 (1, 76.0), Tayabas, Quezon, Southern Luzon, 16 Feb. 1931; FMNH 105071 (34, 51.0–124.0), Puerto Princesa, Palawan Island, 19 May 1962; NSMT-P 45892 (1, 125.0), Igang Bay, Philippines, 20 Mar. 1975; USNM 056237 (1, 107.0), Bulan, Sorsogon, Luzon; YCM-HLP 122 (1, 74.0), Cebu Island; YCM-HLP 443 (1, 59.0), Cebu Island, 28 Jan. – 2 Feb. 1980.

**(1032) *Leiognathusedwardsi* Evermann & Seale, 1907: 68, fig. 7**

**Holotype.**USNM 55904 (127.0), San Fabian, Pangasinan, Luzon.

**Remarks.** The collection date was not indicated in the original description.

**(1033) *Leiognathuspanayensis* Kimura & Dunlap in Kimura et al., 2003: 229, fig. 8**

= *Photopectoralispanayensis* (Kimura & Dunlap, 2003).

**Holotype.** UMMZ 240300 (female, 65.0), Parara Norte, Tigbauan, Iloilo, Panay Island, 27 Mar 2001.

**Paratypes.**USNM 371378 (2, 66.0–68.0), FRLM 29202–06 (5, 46.0–78.0), NSMT-P 64817 (1, 72.0) and UMMZ 240303 (8, 49.0–82.0), same data as the holotype; FRLM 29207 (1, 67.0) and YCM-P 37682 (1, 53.0), Iloilo, Panay Island; YCM-P 37683–92 (10, 51.0–57.0), Philippines, 11 Feb. 1981; BMNH 2002.10.1.1–2 (2, 65.0–76.0), MNHN 2002-3321 (2, 59.0–67.0), UMMZ 240304 (16, 47.0–76.0), same locality as holotype, 10 Jul. 2001; NSMT-P 64816 (1, 64.0), UMMZ 240301 (4, 64.0–83.0) and FRLM 29199 (1, 65.0), same locality as holotype, 20 Jul. 2000; AMS I. 41699-001 (2, 64.0–74.0), UMMZ 240302 (5, 65.0–75.0), MUFS 21943 (1, 65.0) and FRLM 29200-01 (2, 74.0), same locality as holotype, 18 Oct. 2000.

**Other catalog numbers.**BMNH 2002.10.1.1–2 (NHMUK:ecatalogue:2573099).

**(1034) *Leiognathusphilippinus* Fowler, 1918: 15, fig. 7**

= *Eubleekeriasplendens* (Cuvier, 1829).

**Holotype.**ANSP 47486 (65.0), Philippines.

**Paratypes.**ANSP 47487 [ex 47487–90] (4, 63.0–68.0), same data as holotype.

**Remarks.** The original description or ANSP record did not provide a specific locality or collection dates.

**(1035) *Leiognathusstercorarius* Evermann & Seale, 1907: 67, fig. 6**

= *Photolateralisstercorarius* (Evermann & Seale, 1907).

**Holotype.**USNM 55906 (120.7), Bulan, Sorsogon, Luzon.

**Paratypes.**ANSP 33289 [ex USBF 3592] (1), IU (1), BSMP (1), MCZ 32200 [ex USNM 4019] (1, 61.0), CAS-SU 20004 (1), CAS-SU 42171 [ex IU 11595] (1), USNM 55920 (1), USNM 126395 [ex USFC 1490 = USBF 4537] (1) (95.3–101.6), same data as holotype, 1903.

**Remarks.** The original description or USNM record did not indicate the collection date of the holotype. BSMP specimen was presumed destroyed.

**(1036) *Nuchequulaflavaxilla* Kimura, Kimura & Ikejima, 2008: 29, fig. 2b, 4a, 5b, 7b, 10**

**Holotype.** NSMT-P 76261 (female, 106.0), Banate, Iloilo, Panay Island, 6 Dec. 2006.

**Paratypes.** AMS I. 44080-001 (1, 107.0), CAS 224637 (1, 89.0), CSIRO H 6488-01 (1, 82.0), FRLM 32743–45 (3), FRLM 32748–49 |(2), FRLM 32778 (1), FRLM 32780 (1), FRLM32782 (1) (81.0–109.0), KAUM-I 1788 (1, 87.0), MUFS 23149 (1, 81.0), RMNH 35542 (1, 85.0), taken with holotype; BMNH 2007.2.19.2 (1, 68.0), QM I. 38087 (1, 69.0), FRLM 30978, FRLM 30980–81 (3, 79.0–87.0), Roxas, Capiz, Panay Island, 21–24 Feb. 2004; FRLM 28836–37 (2, 79.0–81.0), Iloilo fish market, Panay Island, 18 Nov. 2000.

**Other catalog number.**BMNH 2007.2.19.2 (NHMUK:ecatalogue:3135732).

**(1037) *Photoplagioslaterofenestra* Sparks & Chakrabarty, 2007: 624, figs 3, 4**

= *Equuliteslaterofenestra* (Sparks & Chakrabarty, 2007).

**Holotype.**USNM 387899 [ex USNM 228508] (male, 115.2), 11°55.70'N, 124°28.80'E to 11°53.80'N, 124°29.10'E, Carigara Bay, Samar Sea, 0–86 m, 30 Apr. 1980.

**Paratypes.** AMNH 238682 (2, 115.3–121.0) and USNM 228508 (4, 115.9–127.9), same data as holotype.

**Remarks.** The holotype was removed from USNM 228508 and found missing on Nov. 2022 (Smithsonian Museum Archive 2024).

##### Family Chaetodontidae (432)

**(1038) *Chaetodonadiergastos* Seale, 1910: 116, pl. 1 (fig. 2)**

**Holotype.** BSMP 5800 (116.0), Bantayan Island, Cebu.

**Paratype.** BSMP 5791 (1, 111.0).

**Remarks.** The original description did not indicate the collection date. All type specimens were presumed destroyed.

**(1039) *Chaetodonargentatus* Smith & Radcliffe, 1911: 319, fig. 1**

**Holotype.**USNM 67353 (96.0), Agojo Point, Catanduanes Island, Luzon, 4 m, 10 Jun. 1909.

**Paratype.**USNM 346582 (1, 85.0), Port San Pio, Camaguin Island, northern Mindanao, 4–6 m, 11 Nov. 1908.

**(1040) *Chaetodoncarens* Seale, 1910: 115, pl. 1 (fig. 1)**

= *Chaetodonnippon* Steindachner & Döderlein, 1883.

**Holotype.** BSMP 6173 (108.0), Bantayan Island, Cebu.

**Remarks.** The collection date was not indicated in the original description. The type specimen was presumed destroyed.

**(1041) *Heniochussingularius* Smith & Radcliffe, 1911: 321, fig. 2**

**Holotype.**USNM 67354 (228.0), Alibijaban Island, Ragay Gulf, Luzon, 4–9 m, 6 Mar. 1909.

**(1042) *Roaharaguchiae* Uejo, Senou & Motomura, 2020: [2], figs 1, 3, 5a, 6a, 7a**

**Holotype.** KAUM-I 138475 [ex SNFR 21416]. East China Sea.

**Paratype.** UPVMI 617 (1, 83.9), off Iloilo, Panay Island, 14 Feb. 2013.

**Remarks.** The specimen was purchased at Iloilo Central Market, Iloilo City.

**(1043) *Roarumsfeldi* Rocha, Pinheiro, Wandell, Rocha & Shepherd, 2017: 129, figs 1, 2**

**Holotype.**PNM 15198 (77.53), 13°31.30'N, 120°59.70'E, Puerto Galera, Mindoro Island, 110 m, 10 Apr. 2015.

#### ﻿﻿ORDER GERREIFORMES

##### Family Gerreidae (395)

**(1044) *Gerresphilippinus* Günther, 1862: 258**

= *Gerresfilamentosus* Cuvier, 1829.

**Holotype.**BMNH 1984.6.1.2 (88.9), Philippines.

**Other catalog number.** NHMUK:ecatalogue:2553946.

**Remarks.** The original description or BMNH record did not provide a specific locality or collection date.

**(1045) *Xystaemabaconensis* Evermann & Seale, 1907: 69, fig. 8**

= *Gerresbaconensis* (Evermann & Seale, 1907).

**Holotype.**USNM 55912 (177.8), Bacon, Sorsogon, Luzon.

**Paratype.**USNM 126221 [ex USBF 1491] (1, 139.7), Jolo Island, Sulu Archipelago, 1903.

**Remarks.** The original description or USNM record did not indicate the collection date.

#### ﻿﻿ORDER LOBOTIFORMES

##### Family Lobotidae (504)

**(1046) *Hapalogenysfilamentosus* Iwatsuki & Russell, 2006: 39, figs 1C, D, 3E, F**

**Holotype.** MUFS 7666 (147.0), off Iloilo, Sulu Sea, Panay Island, 30–80 m, 10 Mar. 1981.

**Paratypes.** MUFS 7667–68 (2, 129.0–143.0), taken with holotype; MUFS 7654 (1, 149.0), same locality as holotype., ~40 m, 11 Mar. 1981.

**Remarks.** The type locality is near Cuyo Island, Palawan.

#### ﻿﻿ORDER LUTJANIFORMES

##### Family Haemulidae (435)

**(1047) *Plectorhynchusdoanei* Seale, 1910: 511**

= *Plectorhinchuschaetodonoides* Lacepède, 1801.

**Holotype.** BSMP 4760 (40.0), Sitanki Island, Sulu Archipelago, 15 Jul. 1908.

**Paratype.** BSMP 1695 (1), Cagayan, Mindanao.

**Remarks.** The original description did not indicate the collection date. All type specimens were presumed destroyed.

**(1048) *Hapalogenysmeyenii* Peters, 1866: 96**

= *Parapristipomatrilineatum* (Thunberg, 1793).

**Holotype.** ZMB 1050, Manila, Luzon.

**Remarks.** The collection date was not indicated in the original description.

##### Family Lutjanidae (437)

**(1049) *Aphareusmanillae* Borodin, 1930: 51**

= *Aphareusfurca* (Lacepède, 1801).

**Holotype.** VMM 510, Manila, Luzon.

**Remarks.** The original description did not indicate the collection date.

**(1050) *Lutianusluzonius* Evermann & Seale, 1907: 79, fig. 13**

= *Lutjanusboutton* (Lacepède, 1802).

**Holotype.**USNM 55918 (172.7), Bacon, Sorsogon, Luzon.

**Paratypes.**USNM 126209 [ex USBF 1489] (1) and CAS-SU 20003 (1), same locality as holotype.

**Remarks.** The original description or USNM record did not indicate the collection date of the holotype. The holotype was found during the 1982–1983 inventory on the type shelf but with the wrong label number.

**(1051) *Lutianusorientalis* Seale, 1910: 513**

= *Lutjanusrussellii* (Bleeker, 1849).

**Holotype.** BSMP 2201 (95.0), Limbones Cove, Luzon, 14 Jan. 1908.

**Paratypes.** BSMP (3), Balabac Island, Palawan.

**Remarks.** All type specimens were lost (Kottelat 2013).

**(1052) *Lutjanuspalmeri* Fowler, 1931: 94**

= *Lutjanusmaxweberi* Popta, 1921.

**Holotype.**USNM 89995 (128.0), Malabang, Lanao, Mindanao, 21 May 1908.

**(1053) *Macolormacularis* Fowler, 1931: 181**

**Neotype.**USNM 145811 (218.0), near Palag Bay, Lagonoy Gulf, Luzon, 2–8 m, 16 Jun. 1909.

**Remarks.** The former holotype was lost (USNM 89996), and the former paratype (USNM 145811) was designated as the neotype by Kishimoto et al. (1987). A radiograph of the holotype is available, but not found on the shelf in 2014. in the USNM record.

**(1054) *Paracaesiogonzalesi* Fourmanoir & Rivaton, 1979: 429, figs 18, 19**

**Holotype.**MNHN 1978-0691 (226.0), 06°55.02'N, 122°04.02'E, south of Zamboanga, Mindanao, 150 m.

**(1055) *Pinjalolewisi* Randall, Allen & Anderson, 1987: 12, fig. 3, pl. 1 (fig. C)**

**Holotype.** BPBM 28543, Dumaguete Fish Market, Negros Island, 4 Jun. 1981.

**Paratypes.**USNM 146259 (1), Cebu Market, Cebu Island, 27 Mar 1909.

**Remarks.** A radiograph of the paratype is available in the USNM record.

**(1056) *Pterocaesiorandalli* Carpenter, 1987: 35, pls 3D, 7F**

**Holotype.** BPBM 30819, Moalboal Beach, southwest Cebu Island, 12 Jun. 1983.

**Paratypes.** BPBM 22110 (1), Solpa Island, near Lapu-Lapu, Mactan Island, Cebu, 25 m, 24 Aug. 1977; BPBM 28496 (4), CAS 57407 (1) and USNM 274001 (1), Lapu-Lapu Market, Mactan Island, Cebu, 27 May 1981; BPBM 30820 (1), Dumaguete Fish Market, Negros Island, 29 Jul 1984; BMNH 1985.9.17.2 (1), Mactan Island market, Cebu; and MNHN 1985-0878, Mactan Island, Cebu, May 1981.

**Other catalog number.**BMNH 1985.9.17.2 (NHMUK:ecatalogue:2555902).

**Remarks.** The holotype was purchased at the fish market, reportedly caught at Pescador Island, Tañon Strait.

**(1057) Pterocaesio (Squamosicaesio) tessellata Carpenter, 1987: 47, pls 5A, 7J**

= *Pterocaesiotessellata* Carpenter, 1987.

**Holotype.** BPBM 30821, east Sumilon Island, off southeast tip of Cebu Island, 10 m, 28 Jul. 1984.

**Paratypes.** BPBM 30822 (20), same data as holotype; BMNH 1985.9.17.3 (1), BPBM 30823 (3) and CAS 57408 (1), west of Palawan Island, 30 m, 7 Feb 1985; USNM 264349 (1), Puerto Princesa Fish Market, Palawan Island, 2 Aug. 1979; BPBM 30728 (12) and BPBM 30735 (17), Cubao Fish Market, Manila, Luzon, 27–31 May 1983; BPBM 28542 (6), 3 Jun 1981; BPBM 30668 (4), 26 Feb. 1982; BPBM 30673 (5), 1 Mar. 1982; BPBM 30741 (5), 14 Jun. 1983; all from Dumaguete Fish Market, Negros Island; MNHN 1985-0879, Puerto Princesa, Palawan Island, 30 m, Feb. 1985.

**Other catalog number.**BMNH 1985.9.17.3 (NHMUK:ecatalogue:2555903).

**(1058) Pterocaesio (Squamosicaesio) trilineata Carpenter, 1987: 43, pls 4D, 7I**

= *Pterocaesiotrilineata* Carpenter, 1987.

**Holotype.** ROM 50959. Dravuni Island, Fiji Islands.

**Paratypes.**USNM 144991 (8), Pandaon Island, between Cebu and Bohol islands, 2–4 m, 23 Mar. 1909.

**(1059) *Rhomboplitoidesmegalops* Fowler, 1918: 33, fig. 14**

= *Lutjanuslutjanus* Bloch, 1790.

**Holotype.**ANSP 47507 (162.0), Philippines.

**Remarks.** The original description, nor the ANSP record, did not provide a specific locality or collection date. A photograph and a radiograph are available in ANSP’s record.

#### ﻿﻿ORDER PEMPHERIFORMES

##### Family Champsodontidae (380)

**(1060) *Champsodoncurtipes* Fowler, 1943: 83, fig. 21**

= *Champsodonnudivittis* (Ogilby, 1895).

**Holotype.**USNM 99506 (female, 133.0), Albatross station 5396 (11°57.00'N, 124°12.40'E), Panalangan Point, Talajit Island, between Samar and Masbate islands, 251 m, 15 Mar. 1909.

**Paratypes.**USNM 99507 (15) and USNM 323057 [ex 99507] (1, 101.8), same data as holotype.

**Remarks.**USNM 323057 was re-identified as *Champsodonatridorsalis*. Radiographs of the holotype are available in the USNM record.

**(1061) *Champsodonguentheri* Regan, 1908: 244**

**Syntypes.**BMNH 1879.5.14.367–368 (2, 90.0–140.0 TL), Philippines, 210 m.

**Remarks.** The original description mentioned two specimens without voucher codes from the Philippine Islands and Nares Harbour, Admiralty Islands. One specimen in NMH’s record (1879.5.14.367) from the Philippines has similar collection depth to the one indicated in the description but is not labeled as a type specimen. This could be one of the specimens from the Philippines mentioned by Regan .

##### Family Howellidae (398)

**(1062) *Schistopercamacrobrachium* Fowler, 1943: 62, fig. 9**

= *Bathysphyraenopssimplex* Parr, 1933.

**Holotype.**USNM 99489 (100.0, Tataan Pass, Simulac Island, southern end of Basún Channel, Sulu Archipelago, 19 Feb. 1908.

**Paratypes.**USNM 99529 (1, 86.0), Albatross station 5423, Cagayancillo Island, Palawan, 929 m, 31 Mar. 1909; USNM 99532 (2, 68.0–77.0), Albatross station 5296, Matacot Point, southern Luzon, 384 m, 24 Jul. 1908; USNM 99533 (1, 95.0), Albatross station 5216, Anima Sola Island, between Burias and Luzon islands, 393 m, 22 Apr. 1908.

**Remarks.** Radiographs and photographs of the holotype are available in the USNM record.

##### Family Epigonidae (400)

**(1063) *Hynnodusmegalops* Smith & Radcliffe in Radcliffe, 1912b: 445, pl. 38 (fig. 3)**

= *Epigonusmegalops* (Smith & Radcliffe, 1912).

**Holotype.**USNM 70255 (125.3), Albatross station 5388 (12°51.50'N, 123°02.62'E), near Bagatao Island, between Burias and Luzon islands, 413 m, 11 Mar. 1909.

**Paratypes.**USNM 147374 (2, 106.6–116.0), same data as holotype; USNM 147375 (1, 84.6), Albatross station 5508, Bohol Sea, northern Mindanao, 494 m, 5 Aug. 1909.

**Remarks.** Radiographs of the holotype are available in the USNM record.

##### Family Glaucosomatidae (404)

**(1064) *Glaucosomataeniatus* Fowler, 1934: 357, fig. 108**

= *Glaucosomabuergeri* Richardson, 1845.

**Holotype.**USNM 93173 (146.0 TL), Albatross station 5213 (12°15.00'N, 123°57.50'E), Destacado Island, east of Masbate, 146, 20 Apr. 1908.

**Remarks.** Radiographs of the holotype are available in the USNM record.

##### Family Ostracoberycidae (405b)

**(1065) *Ostracoberyxdorygenys* Fowler, 1934: 353, fig. 105**

**Holotype.**USNM 93143 (175.0 TL), Albatross station 5503, Macabalan Point Lighthouse, northern Mindanao, 413 m, 4 Aug. 1909.

**Paratypes.**CAS-SU 40193 [ex USNM 93143] (3), USNM 93380 (10) and USNM 93381 (8), Albatross station 5291, Escarceo Lighthouse, southern Luzon, 296–316 m, 23 Jul. 1908.

**Remarks.** Radiographs of the holotype are available in the USNM record.

##### Family Bathyclupeidae (408)

**(1066) *Bathyclupeagracilis* Fowler, 1938: 34, fig. 8**

= *Neobathyclupeagracilis* (Fowler, 1938).

**Holotype.**USNM 93320. Between and Gillolo and Makyan Island, Indonesia.

**Paratypes.**USNM 93313 (1), Albatross station 5508, Camp Overton Lighthouse, northern Mindanao, 494 m, 5 Aug. 1909; USNM 93315 (2), Albatross station 5222, San Andreas Island, between Marinduque and Luzon islands, 357 m, 24 Apr. 1908; USNM 93316 (1), Albatross station 5374, Tayabas Lighthouse, Marinduque Island, 347 m, 2 Mar. 1909; USNM 93318 (1), Albatross station 5402, Capitancillo Island Lighthouse, between Leyte and Cebu islands, 344 m, 16 Mar. 1909; USNM 93319 (4) and USNM 93321 (6), Albatross stations 5503–04, Macabalan Point Lighthouse, northern Mindanao, 366–413 m, 4–5 Aug. 1909.

**Remarks.** The original description, nor the USNM record, did not indicate the sizes of the specimens.

**(1067) *Bathyclupeamegaceps* Fowler, 1938: 33, fig. 7**

= *Neobathyclupeamegaceps* (Fowler, 1938).

**Holotype.**USNM 93323 (238.0), Albatross station 5507 (08°21.20'N, 124°12.10'E), Camp Overton Lighthouse, northern Mindanao, 777 m, 5 Aug. 1909.

**Paratypes.**USNM 93322 (1), Albatross station 5538, Apo Island, between Negros and Siquijor islands, 468 m, 19 Aug. 1909; USNM 93324 (1), Albatross station 5405, Ponson Island, Dupon Bay, Leyte, 479 m, 17 Mar. 1909.

**Remarks.** Radiographs of the holotype are available in the USNM record.

#### ﻿﻿ORDER PRIACANTHIFORMES

##### Family Cepolidae (444)

**(1068) *Loxopseudochromisdorypterus* Fowler, 1934: 354, fig. 106**

= *Owstoniadorypterus* (Fowler, 1934).

**Lectotype.**USNM 93166 (64.9), Albatross station 5516 (08°46.0'N, 123°32.50'E), Tagolo Point Lighthouse, northern Mindanao, 320 m, 9 Aug. 1909.

**Paralectotype.**USNM 410302 (1, 56.0), same data as lectotype.

**Remarks.** The lectotype and paralecotype were designated by Smith-Vaniz and Johnson (2016). Radiographs of the holotype are available in the USNM record.

**(1069) *Owstoniaaurora* Liao, Reyes & Shao, 2022: 123, figs 1, 2**

**Holotype.** ASIZP 0068194 (male, 74.9), 16°01.20'N, 121°52.80'E, Aurora, east of Luzon, 344–347 m, 1 Jun. 2007.

**Paratypes.** ASIZP 0067846 (male, 69.8), taken with the holotype, 342–358 m, 20 May 2007; ASIZP 0068193 (female, 88.0) same data as holotype.

**(1070) *Owstoniacontodon* Smith-Vaniz & Johnson, 2016: 40, figs 29, 30**

**Holotype.**PNM 15193 [ex USNM 438011] (female, 165.0), Iloilo City “Super” Fish Market, Panay Island, 23 Jul. 2015.

**Paratypes.**USNM 438013 (1, 162.0), USNM 437757 (1, 303.0), USNM 437759 (1, 260.0), USNM 437761 (1, 240.0), USNM 437766 (1, 262.0) and USNM 437775 (1, 193.0), same data as holotype; MNHN 2002-2971 (1, 146.0), Musorstom 3, Leyte Island, 205–214 m, Jun. 1985.

**Remarks.** The vendor mentioned that the holotype came from the shipment of fresh fish from Palawan.

**(1071) *Owstoniageminata* Smith-Vaniz & Johnson, 2016: 48, fig. 37**

**Holotype.**MNHN 2016-0021. Vanuatu.

**Paratype.** ASIZP 68128 (1, 79.0), Aurora station cp2719, Lamon Bay, Luzon, 155–160 m, 29 May 2007.

**(1072) *Owstoniamelanoptera* Smith-Vaniz & Johnson, 2016: 65, fig. 54**

**Holotype.** AMS I.36454-005 (16.0), 13°08.98'N, 124°04.73'E to 13°09.84'N, 124°00.01'E, Albay Gulf, Luzon, 363–385 m, 3 Sep. 1995.

**(1073) *Owstoniasarmiento* Liao, Reyes & Shao, 2009: 522, figs 1A, 1B**

**Holotype.**PNM 17006 [ex ASIZP 0067939] (61.0), 15°55.80'N, 121°46.80'E, Aurora, east of Luzon, 292–307 m, 21 May 2007.

**Paratypes.** ASIZP 0068216 (1, 64.0), 302–309 m, 2 Jun. 2007; ASIZP 0067820 (1, 63.0) and ASIZP 0068380 (1, 63.0), 262–278 m, 20 May 2007, both taken with holotype.

**(1074) *Sphenanthiasmacrophthalmus* Fourmanoir, 1985: 38, fig. 3**

= *Owstoniamacrophthalma* (Fourmanoir, 1985).

**Holotype.**MNHN 1983-0557 (68.0), Musorstom 2 station 2 (14°00.00'N, 120°18.00'E), off Lubang Island, west of Luzon. 184–186 m.

**Paratype.**MNHN 1983-0558 (2, 52.0–70.0), taken with holotype, 185 m, 20 Nov. 1980.

**Remarks.** The original description or MNHN record did not indicate the collection date. The holotype was previously lost and a paratype was designated as a neotype (which should have been a lectotype) by Bauchot and Desoutter (1989).

**(1075) *Sphenanthiasnigromarginatus* Fourmanoir, 1985: 40, fig. 4**

= *Owstonianigromarginata* (Fourmanoir, 1985).

**Holotype.**MNHN 1982–1251 (68.0), Musorstom 2 station 2 (14°01.02'N, 120°16.98'E), off Lubang Island, west of Luzon, 184–186 m, 20 Nov. 1980.

**Paratype.**MNHN 1982-1252 (1), same data as holotype.

**(1076) *Sphenanthiaspectinifer* Myers, 1939: 19**

= *Owstoniasibogae* (Weber, 1913).

**Holotype.**USNM 93455 (141.0), Albatross station 5255 (07°03.00'N, 125°39.00'E), off Dumalag Island, Davao Gulf, Mindanao, 183 m, 18 May 1908.

**Remarks.** Radiographs of the holotype are available in the USNM record.

##### Family Priacanthidae (430)

**(1077) *Priacanthusfitchi* Starnes, 1988: 164, pl. 1h; fig. 3e**

**Holotype.**USNM 263760. Sumatra, Indonesia.

**Paratypes.**USNM 183207 (1, 117.0), Albatross station 5406, Ponson Island, near Dupon Bay, Leyte, 545 m, 17 Mar. 1909.

**(1078) *Priacanthussagittarius* Starnes, 1988: 178, pl. 3 e–g; figs 3, 5, 8, 12, 18**

**Holotype.**USNM 285042. Southern Sumatra, Indonesia.

**Paratypes.**CAS 54998 (1, 69.0), 5 miles off northwest Fortune Island, Batangas, Luzon, 2 Oct. 1947.

**(1079) *Priacanthuszaiserae* Starnes & Moyer in Starnes, 1988: 183, pl. 3h; fig. 3d**

**Holotype.**USNM 284063. Ako, Miyake-Jima, Japan.

**Paratypes.**USNM 289282 (1, 230.0), GDJ 87-6, Siayan Island, Batanes, 25 Apr. 1987.

**Remarks.** The paratype was purchased from local fishermen.

#### ﻿﻿ORDER STOMIATIFORMES

##### Family Sternoptychidae (201)

**(1080) *Neophosnexilis* Myers, 1932: 61**

= *Thorophosnexilis* (Myers, 1932).

**Holotype.**CAS-SU 24798 (60.0), Albatross station 5516, Tagolo Point Lighthouse, northern Mindanao.

**Remarks.** The collection date was not indicated in the original description.

**(1081) *Polyipnusdanae* Harold, 1990: 1112, fig. 1**

**Holotype.** ZMUC P206919 (male, 26.5), Dana station 3716 (19°18.50'N, 120°13.00'E), northwest of Luzon, 2,000 m, 22 May 1929.

**Paratypes.** ZMUC P206920 (male, 24.2), ZMUC P208577 (female, 24.8), ZMUC P208578 (female, 27.3), ZMUC P208579 (female, 25.4), ZMUC P208580 (female, ~26.0) and ZMUC P208581 (male, ~30.0), Dana station 3729 (20°03.50'N, 120°50.00'E), north of Luzon, 1,000 m, 14 Jun. 1929.

**(1082) *Polyipnusfraseri* Fowler, 1934: 257, fig. 19**

**Holotype.**USNM 92324 (51.0), Albatross station 5476, San Bernardino Lighthouse, east of Luzon, 494 m, 24 Jun. 1909.

**Remarks.** The original description mentioned Albatross station 5648, Buton Strait, Philippines as the type locality, but USNM record indicated station 5476 (San Bernardino Lighthouse, east coast of Luzon) as the type locality. Radiographs of the holotype are available in the USNM record (Smithsonian Museum Archive 2024).

**(1083) *Polyipnusovatus* Harold, 1994: 480, fig. 25**

**Holotype.**CAS 33347 (45.0), 13°00.00'N, 121°00.00'E, Pagapas Bay, Luzon, 194–209 m, 21 Jul. 1966.

**(1084) *Polyipnussoelae* Harold, 1994: 520, fig. 55**

**Holotype.** AMS I.22808-028 (54.8), 17°59.00'N, 118°17.00'E, northwest of Luzon, 404–420 m, 3 Apr. 1982.

**Paratypes.** AMS 1.2280.8[0.28] (29 of 30) from AMS 1.2280.8-028 (38.5–52.1), same data as holotype.

**(1085) *Polyipnusspinosus* Günther, 1887: 170, pl. 51 (fig. B)**

**Holotype.**BMNH 1887.12.7.159 (57.2), Challenger station 200 (06°47.00'N, 122°28.00'E), Moro Gulf, Mindanao, 23 Oct. 1874.

**Other catalog number.** NHMUK:ecatalogue:3112556.

**(1086) *Polyipnustriphanos* Schultz, 1938: 140, fig. 45**

**Holotype.**USNM 103027 (20.0), Albatross station 5368 (13°35.50'N, 121°48.00'E), Tayabas Lighthouse, Marinduque Island, 331 m, 23 Feb. 1909.

**Paratypes.**USNM 103028 (2, 17.5–21.5), Albatross station 5500, Macabalan Point Lighthouse, northern Mindanao, 488 m, 4 Aug. 1909.

**Remarks.** Radiographs of the holotype are available in the USNM record.

**(1087) *Polyipnusunispinus* Schultz, 1938: 137, fig. 43**

**Holotype.**USNM 103153 [ex USNM 103029] (20.5), Albatross station 5451 (13°22.37'N, 124°00.80'E), San Bernardino Strait, east of Luzon, 695 m, 5 Jun. 1909.

**Paratypes.**USNM 103029 (5, 16.0–19.0), same data as holotype.

**Remarks.** Radiographs of the holotype are available in the USNM record.

**(1088) *Thorophoseuryops* Bruun, 1931: 288, pl. 8 (fig. 2)**

**Holotype.** ZMUC P202774, Dana station 3736 (09°17.00'N, 123°58.00'E), Mindanao Sea, 200 m.

**Paratypes.**USNM 326695 (1) and USNM 326696 (1), Mindanao Sea, 28 Jun. 1929.

**Remarks.** The original description did not indicate the collection date of the holotype.

##### Family Stomiidae (203)

**(1089) *Eustomiascrossotus* Gibbs, Clarke & Gomon, 1983: 49, figs 11b, 12d**

**Holotype.** ZMUC P202846 (male, 128.1), 16°55.00'N, 120°03.00'E, off La Union, west of Luzon, 0–300 m, 15 Jun. 1929.

**Paratypes.** IOAN uncat. (female, 153.7), 17°53.00'N, 127°56.00'E, Philippine Sea, ~595 km east of Luzon, 0–200 m, 11 Feb 1975; USNM 223965 (female, 122.9), 15°21.00'N, 126°54.00'E, Philippine Sea, ~570 km east of Luzon Island, 0–200 m, 13 Feb. 1975.

**(1090) *Eustomiassuluensis* Gibbs, Clarke & Gomon, 1983: 77, fig. 20b**

**Holotype.**USNM 223714 (female, 138.3), 09°22.00'N, 122°06.00'E, eastern Sulu Sea, west of southern Negros Island, 0–110 m, 5 Jun. 1975.

**Paratype.** ZMUC P202847 (male, 109.1), southeast Sulu Sea, west of Zamboanga, Mindanao, 0–300 m, 30 Jun. 1929.

**(1091) *Melanostomiasglobulifer* Fowler, 1934: 263, fig. 24**

**Holotype.**USNM 92339 (180.0 TL), Albatross station 5438 (15°54.70'N, 119°44.70'E), Dasol Bay, west of Luzon, 543 m, 8 May 1909.

**(1092) *Melanostomiasstewarti* Fowler, 1934: 262, fig. 23**

**Holotype.**USNM 92338 (215.0 TL), Albatross station 5405, Ponson Island, Dupon Bay, Leyte, 479 m, 17 Mar. 1909.

**(1093) *Melanostomiasvierecki* Fowler, 1934: 265, fig. 25**

**Holotype.**USNM 92340 (118.0 TL), Albatross station 5215, Palanog Point Lighthouse, east of Masbate Island, 1105 m, 21 Apr. 1908.

**(1094) *Pseudeustomiasmyersi* Fowler, 1934: 262, fig. 22**

= *Stomiasaffinis* Günther, 1887.

**Holotype.**USNM 92342 (108.0 TL), Albatross station 5205, Nogas Island, southern Panay Island, 752 m, 3 Feb. 1908.

#### ﻿﻿ORDER URANOSCOPIFORMES

##### Family Pinguipedidae (381)

**(1095) *Parapercisdiplospilus* Gomon, 1981: 990, figs 1, 2**

**Holotype.**USNM 220470 (75.0), 11°22.00'N, 123°19.80'E, SE of Sicogon Island, between Negros and Masbate islands, 38.4 m, 9 Jun. 1978.

**Paratypes.** AMS I.21362-001 (3, 64.0–76.9), BMNH 1979.11.2.1–3 (3, 56.9–73.4), BPBM 22770 (3, 56.6–78.0) and CAS 44715 (3, 61.7–70.1), same data as holotype; USNM 220406 (1, 56.5) and USNM 220407 (1, 48.7), southeast of Gigante Island, between Negros and Masbate islands, 38.4 m, 8 Jun. 1978; USNM 220409 (6, 51.6–66.6), east of Sicogon Island, between Negros and Masbate islands, 47.6 m, 4 Jun. 1978.

**Other catalog number.**BMNH 1979.11.2.1–3 (NHMUK:ecatalogue:2545242).

**Remarks.** A radiograph of the holotype is available in the USNM record.

**(1096) *Parapercisflavipinna* Johnson & Motomura, 2017: 131, fig. 3**

**Holotype.** KAUM-I 52614 (male, 115.8), 10°41.00'N 122°35.00'E, off Iloilo, Panay Island, 13 Feb. 2013.

**Paratype.** QM I.40748 (1, 104.3), same data as holotype.

**(1097) *Parapercisfuscolineata* Fourmanoir, 1985: 36, fig. 1**

**Holotype.**MNHN 1984-0430 (78.0), Musorstom 2 station 51 (13°58.98'N, 120°16.02'E), north of Lubang Island, west of Luzon, 170–187 m, 27 Nov. 1980.

**Paratype.** BPBM 29668 (1, 65.0), taken with holotype.

**(1098) *Parapercislineopunctata* Randall, 2003: 12, figs 7, 8**

**Holotype.** BPBM 36841 (female, 57.8), Bolinao, Pangasinan, Luzon, 5–7 m, 9 Oct. 1995.

**Paratype.**CAS-SU 27131 (1, 60.5), Cebu Island, 27 Aug. 1931.

**(1099) *Parapercispacifica* Imamura & Yoshino, 2007: 88, figs 3B, 6, 7, 8A**

**Holotype.** NSMT-P 34936. Amami-oshima Island, Ryukyu Islands, Japan.

**Paratypes.**USNM 228413 (2, 147.5–185.5, male and intersexual specimen with female color), Cocoro Island, Cuyo, Palawan, 0–2.1 m, 26 May 1978; USNM 309434 (2 males, 2 females), Barrio Anqib, Station Ana, Cagayan, Luzon, 18 May 1989.

**Remarks.**USNM 309434 originally contained five specimens, but one was re-cataloged as USNM 385006 (non-type).

**(1100) *Parapercisrufa* Randall, 2001a: 3505**

**Holotype.**MNHN 1984-0431 (87.0), Musorstom 2 station 8 (13°55.00'N, 120°20.00'E), off Lubang Island, west of Luzon, 85–90 m, 21 Nov. 1980.

**Paratypes.** BPBM 29666 (1), same data as holotype; MNHN 1984-04342 (2), possible types.

**Remarks.** A replacement name for *Parapercisrosea* Fourmanoir, 1985 pre-occupied by *Parapercisrosea* (Liénard 1829) (Randall 2001). The original description mentioned MNHN 1984-31 as the holotype but in error; four specimens (46.0–68.0) were mentioned but only two are listed with catalog numbers. Ho and Causse (2012) consider MNHN 1984-04342 as paratype but MNHN record did not mention these are paratypes, although these have the same collection information as the holotype.

**(1101) *Parapercisrubricaudalis* Johnson & Motomura, 2017: 126, fig. 2**

**Holotype.** CSIRO H.6596-01. Cape Leveque, Western Australia.

**Paratype.** AMS I.46929-001 (1, 59.2), Zambales, Luzon, 1 Dec. 2015.

**(1102) *Parapercissoliorta* Johnson & Motomura, 2017: 122, fig. 1**

**Holotype.** KAUM-I 52626 (male, 112.5), 10°41.00'N 122°35.00'E, off Iloilo, Panay Island, 15 Feb. 2013.

#### ﻿﻿ORDER-LEVEL *INCERTAE SEDIS* IN CARANGARIA

##### Family Sphyraenidae (334)

**(1103) *Sphyraenaaureoflammea* Seale, 1910: 502**

= *Sphyraenaobtusata* Cuvier, 1829.

**Holotype.** BSMP 4138 (280.0), Zamboanga, Mindanao, 22 May 1908.

**Paratypes.** BSMP (4).

**Remarks.** All type specimens were lost (Kottelat, 2013).

##### Family Polynemidae (408a)

**(1104) *Polynemuslongifilis* Cuvier in Cuvier & Valenciennes, 1829: 365**

= *Polynemusparadiseus* Linnaeus, 1758.

**Syntype.**MNHN A-3045 (1, 115.0), 14°36.00'N, 120°58.02'E, Manila, Luzon.

**Remarks.** Locality is probably an error due to the distribution of other syntypes (Motomura et al. 2002).

**(1105) *Polydactyluslongipes* Motomura, Okamoto & Iwatsuki, 2001: 1087, fig. 1**

**Holotype.**USNM 363173 (female, 159.0), Davao Market, Mindanao, 10 Jan. 1977.

**Paratypes.** FSKU-P 19840 (female, 134.0) and MUFS 20290 (female, 152.0), same data as holotype.

**(1106) *Polydactylusopercularis* Seale & Bean, 1907: 234**

= *Filimanussealei* (Jordan & Richardson, 1910).

**Holotype.**USNM 57844 (171.5), Zamboanga, Mindanao.

**Remarks.** Subjectively invalid; preoccupied by *Trichiodonopercularis* Gill, 1863; replaced by *Polydactylusseali* Jordan & Richardson, 1910 ((Feltes 1991; Motomura 2004; Tashiro et al. 2013). The original description nor USNM record did not indicate the collection date but radiographs of the holotype are available.

**(1107) *Polydactylussealei* Jordan & Richardson, 1910: 16**

= *Filimanussealei* (Jordan & Richardson, 1910).

**Holotype.**USNM 57844 (171.5), Zamboanga, Mindanao.

**(1108) *Polydactyluszophomus* Jordan & McGregor in Jordan & Seale, 1907: 11, fig. 4**

= *Polydactylusmicrostoma* (Bleeker, 1851).

**Holotype.**CAS-SU 20113, Cavite, Luzon, 1 Jun. 1900.

**Paratypes.**CAS-SU 20111 (1) and USNM 55598 (2) (6.35–203.2), same data as holotype.

**Remarks.**Böhlke (1953) mentioned that USNM 55598 is the holotype. However, CAS’ records indicated CAS 20113 as the holotype. CAS 20111 has two specimens and one was re-cataloged as SU 69883 but considered as non-type by H. Motomura (Smithsonian Museum Archive 2024). A photograph and a radiograph of the holotype are available in the CAS record.

#### ﻿﻿ORDER-LEVEL *INCERTAE SEDIS* IN EUPERCARIA

##### Family Pomacanthidae (433)

**(1109) *Chaetodontopluscaeruleopunctatus* Yasuda & Tominaga, 1976: 130, fig. 1**

**Holotype.** ZUMT 52825 (77.0), Philippines, Jun. 1972.

**Remarks.** The original description did not provide a specific locality.

**(1110) *Holacanthuschapmani* Herre, 1933: 19**

= *Genicanthuslamarck* (Lacepède, 1802).

**Holotype.**CAS-SU 25504 (115.0), Dumaguete, Negros Island, 26 Jun. 1931.

**Paratypes.**BMNH 1933.3.11.394 (1) and CAS-SU 25506 (2) (96.0–126.0), reef between Burias and Ticao islands, Aug. 1929; CAS-SU 25505 (1, 101.0) and UMMZ 100225 (1), Jolo Island, Sulu Archipelago, 2Aug. 1931.

**Other catalog number.**BMNH 1933.3.11.394 (NHMUK:ecatalogue:2511633).

**Remarks.**BMNH 1933.3.11.394 is noted “syntype” in NMH’s record. A photograph and a radiograph of the holotype are available in the CAS record.

**(1111) *Holacanthusmultifasciatus* Smith & Radcliffe, 1911: 324, fig. 3**

= *Centropygemultifasciata* (Smith & Radcliffe, 1911).

**Holotype.**USNM 67355 (93.0), Port Galera, Mindoro Island, 27 Oct. 1909.

**Paratypes.**USNM 180339 (1), same data as holotype; UNSM 180341 (1), Romblon Harbor, Romblon, 2–5 m, 25 Mar. 1908.

**Remarks.** It is in the Bureau of Fisheries possession based on the USNM record. Need confirmation.

##### Family Malacanthidae (434)

**(1112) *Hoplolatiluschlupatyi* Klausewitz, McCosker, Randall & Zetzsche, 1978: 42, figs 1–14**

**Holotype.** SMF 14089, Philippines (probably Cebu).

**Paratype.** BPBM 21090 (1), obtained from San Francisco aquarium fish dealer; CAS 40825 (1, 107.5), Batangas or Cebu (?).

**Remarks.** The original description did not indicate the collection date. The BPBM specimen contained a fish imported from the Philippines. The locality of CAS 40825 in the original description is Batangas, but the accession file indicated Cebu as the locality.

**(1113) *Hoplolatilusmarcosi* Burgess, 1978: 47**

**Holotype.**USNM 217860, around Tingloy Island, off Orensi near Mabini, Batangas, Luzon 18–20 m.

**Paratype.**USNM 217859 (1), same data as holotype.

**Remarks.** The original description or USNM record did not indicate the collection date of the type specimens but radiographs of the holotype are available.

**(1114) *Hoplolatiluspurpureus* Burgess, 1978: 43**

**Holotype.**USNM 217858, Tingloy Island, near Mabini, Batangas, Luzon, 18–24 m.

**Paratype.**USNM 217857 (1), same data as holotype.

**Remarks.** The original description or USNM record did not indicate the collection date of the holotype.

##### Family Siganidae (446)

**(1115) *Amphacanthusovatus* Marion de Procé, 1822: 133**

Manila Bay, Luzon.

**Remarks.** No type known. Questionably a synonym of *Siganusfuscescens* (Houttuyn, 1782) (Kottelat 2013). A further study is needed to confirm its status.

**(1116) *Lounimaculatus* Evermann & Seale, 1907: 98, fig. 19**

= *Siganusunimaculatus* (Evermann & Seale, 1907).

**Holotype.**USNM 55915 (190.5), Bacon, Sorsogon, Luzon.

**Remarks.** The original description or USNM record did not indicate the collection date.

**(1117) *Siganuspunctatissimus* Fowler & Bean, 1929: 298, fig. 25**

**Holotype.**USNM 89979 (285.0), Masinloc Bay, Zambales, west of Luzon, 23 Nov. 1908.

**Remarks.** The original description lists information on several specimens from the Philippines but does not elaborate if these are type specimens. The USNM records only shows USM 89979 as the only type specimen.

##### Family Emmelichthyidae (497)

**(1118) *Emmelichthyspapillatus* Girard, Santos & Bemis, 2024: 99, figs 1–3B**

**Holotype.**PNM 15806 [ex KAMU-I 91845] (130.0), Oton Fish Market, Iloilo, Panay Island (11°00.00'N, 123°00.00'E) (likely captured off Iloilo), 12 Sep. 2016.

**Paratypes.**USNM 424606 (1, 122.0) and KAUM-I 193858 [ex USNM 424607] (1, 119.0), Pasil Market, Cebu Island (10°17.50'N, 123°53.52'E), 1 Jun. 2013.

**Remarks.** The holotype was previously identified as *E.struhsakeri* (Motomura et al., 2017).

##### Family Sciaenidae (498)

**(1119) *Otolithusleuciscus* Günther, 1872: 398**

= *Pennahiaaneus* (Bloch, 1793).

**Syntypes.**BMNH 1872.10.18.128–129 (2, 152.4), rivers near Laguna de Bay, Luzon.

**Other catalog number.** NHMUK:ecatalogue:3104703.

**Remarks.** The original description mentioned Manila Bay as the type locality but NMH’s record indicated these were caught in rivers near Laguna de Bay in Luzon. The collection date was also not indicated in the original description.

**(1120) Johnius (Johnieops) philippinus Sasaki, 1999: 271**

= *Johniusphilippinus* Sasaki, 1999.

**Holotype.** NSMT-P 55912 (67. l), Agdao Fish Market, Davao, Mindanao, 20 Dec. 1998.

**Paratypes.** BSKU 86010 (10, 38.2–53.6), BSKU 86011 (9, 53.2–61.4), BSKU 86012 (10, 57.6–69.7), BSKU 86013 (2, 73–73.2), BSKU 86014 (1, 71.5) and NSMT-P 55913 (9, 49.l–56.3), taken with the holotype.

##### Family Callanthiidae (502)

**(1121) *Callanthiascrosnieri* Fourmanoir, 1981: 91, fig. 15**

= *Grammatonotuscrosnieri* (Fourmanoir, 1981).

**Holotype.**MNHN 1978-79 (119.0), Musorstom 1station 6 (14°01.02'N, 120°19.98'E), off Lubang Island, west of Luzon, 182–200 m, 19 Mar. 1976.

**Paratypes.**MNHN 1978-0080 (2, 115.0–117.0), Musorstom 1 station 20, same locality as holotype, 208 m, 21 Mar. 1976.

**Remarks.** A photograph of the holotype is available in the MNHN record.

**(1122) *Grammatonotusbrianne* Anderson, Greene & Rocha, 2016: 290, fig. 1**

**Holotype.**PNM 15196 [ex CAS 237785] (84.4), 13°48.04'N, 120°54.64'E, Mabini Dive and Trek, off Batangas, Luzon, 150 m, 21–22 May 2014.

**Paratypes.**CAS 237786 (1, 77.7), CAS 237787 (1, 72.9), and USNM 432499 [ex CAS 237788] (1, 82.4), taken with holotype.

##### Family Sillaginidae (503)

**(1123) *Sillagoargentifasciata* Martin & Montalban, 1935: 226, pl. 1 (fig. 3)**

**Holotype.** BSMP 15680, Lumbucan Island, Balabac Strait, Palawan, 29 Nov. 1927.

**Paratypes.** BSMP 31146–47 (2, 81.8–116.3) same data as holotype.

**Remarks.** All type specimens were presumed destroyed.

##### Family Latilidae

**(1124) *Branchiostegusilocanus* Herre, 1928: 32, pl. 3**

**Holotype.** BSMP (270.0), Narvacan Market, Ilocos, Luzon.

**Remarks.** The original description did not indicate the collection date. The type specimen was presumed destroyed.

**(1125) *Branchiostegussaitoi* Dooley & Iwatsuki, 2012: 32, figs 1–5**

**Holotype.** MUFS 36081 (male, 329.0), 13°38.40'N, 121°25.60'E, Laiya, Batangas, Luzon, 210 m, 14 Apr. 2011.

**Paratype.** NSMT-P 106562 (1 male, 315.6), same data as holotype.

**(1126) *Branchiostegusvittatus* Herre, 1926b: 535, pl. 2**

**Syntypes.** Uncat. (3, 240.0), Manila market, Luzon.

**Remarks.** The original description did not provide catalog numbers or collection date.

#### ﻿﻿ORDER-LEVEL *INCERTAE SEDIS* IN OVALENTARIA

##### Family Ambassidae (283)

**(1127) *Priopislungi* Jordan & Seale, 1907: 18, fig. 6**

= *Ambassisurotaenia* Bleeker, 1852.

**Holotype.**USNM 53066 (72.39 mm), Cavite, Luzon.

**Paratype.**CAS-SU 9242 (1), Manila Bay, Luzon, 1907.

**Remarks.** The holotype was originally published as USNM 53060, but that voucher number was already assigned to *Gonostomaelongatum*. The original description or USNM record did not indicate the collection date of the holotype.

##### Family Plesiopidae (286)

**(1128) *Acanthoplesiopsechinatus* Smith-Vaniz & Johnson, 1990: 239, figs 1H, 2H, 11**

**Holotype.** BPBM 34177. Ambon Bay, Molucca Islands, Indonesia.

**Paratype.**USNM 146453 (1), Albatross station 5555, Cabalian Point, Jolo Island and vicinity, 62 m, 18 Sep. 1909.

**(1129) *Beliopsbatanensis* Smith-Vaniz & Johnson, 1990: 232, figs 1F, 2F, 7, 8**

**Holotype.**USNM 288976 (female, 21.0), Chawa Point (20°25.75'N, 121°56.67'E), Batan Island, Batanes, 9–12 m, 1 May 1987.

**Paratype.**USNM 309905 (1, 21.1), taken with holotype.

**Remarks.** The type specimens were originally mixed; the paratype was separated from holotype.

**(1130) *Calloplesiopsniveus* Fowler & Bean, 1930: 317**

= *Calloplesiopsaltivelis* (Steindachner, 1903).

**Holotype.**USNM 89986, Romblon reef, Romblon Island, 26 Mar. 1908.

**Paratypes.**USNM 146404 (1, 148.0), Escarpada Island, Bagacay Bay, between Samar and Masbate islands, 2–9 m, 13 Mar. 1909; USNM 146405 (1, 120.0), Bisucay Island, Cuyo, Palawan, 2–5 m, 9 Apr. 1909; USNM 146406 (2, 134.0–143.0), Little Santa Cruz Island, Zamboanga, Mindanao, 2–9 m, 28 May 1908; USNM 146407 (1, 80.0), Port Galera, Mindoro Island, 27 Oct 1909; USNM 146408 (2, 108.0–151.0), Port Palapag, Batag Island, Samar, 2–5 m, 3 Jun. 1909; USNM 146409 (1, 89.0), Pandan Island, Sablayan Bay, Mindoro, 2–3 m, 13 Dec. 1908; USNM 146410 (2, 112.0–135.0), near bay anchorage, Sabtan Island, Batanes, 3–8 m, 8 Nov. 1908; USNM 146411 (1, 160.0), San Miguel Island, Tabaco Bay, east of Luzon, 3–5 m, 4 Jun. 1909; USNM 146412 (1, 106.0), 2^nd^ Anchorage, Tutu Bay, Jolo Island, Sulu Archipelago, 1–6 m, 19 Sep. 1909.

**Remarks.** The original description mentioned East Indies, Philippines as the holotype locality, but the Catalog of Fishes stated Gomomo Island in Pitt Passage, Philippines as the locality (Fricke et al. 2024). However, USNM record indicated Romblon Reef as the type locality for USNM 89986 (Smithsonian Museum Archive 2024).

**(1131) *Plesiopscephalotaenia* Inger, 1955: 272, fig. 4**

**Holotype.** FMNH 44708, Sitankai Island, Sulu Archipelago, 11 Aug. 1931.

**Paratypes.** FMNH 47293 (3), same data as holotype; USNM 146460 (1), Rapu-rapu Island, east of Luzon, 3–5 m, 22 Jun. 1909; USNM 146462 (1), Mantacao Island, west of Bohol, 3–9 m, 8 Apr. 1908; USNM 146464 (1), Port San Miguel, Ticao Island, between Burias and Luzon islands, 2–9 m, 21 Apr. 1908; USNM 146466 (7), Cataingan Bay, east of Masbate Island, 18 Apr. 1908; USNM 146467 (1), Maculabo Island, Camarines Norte, east of Luzon, 2–5, 14 Jun. 1909; USNM 151323 (1), Tara Island, Coron, Palawan, 15 Dec. 1908.

**(1132) *Plesiopsgracilis* Mooi & Randall, 1991: 374, pl. 1A; figs 1–3**

**Holotype.** BPBM 31410. Auluptagel Island, Palau Islands.

**Paratypes.**USNM 226939 (1, 70.0), Sept. 1965 and USNM 226940 (1, 70.0), 27 Sep. 1976; both bought from Puerto Princesa City Market, Palawan Island.

##### Family Pomacentridae (288)

**(1133) *Abudefdufazurepunctatus* Fowler & Bean, 1928: 149**

= *Chrysipteraoxycephala* (Bleeker, 1876).

**Holotype.**USNM 89963, Romblon Harbor, Romblon Island, 26 Mar. 1908.

**Paratypes.**ANSP 85288 [ex USNM 95301] (4); USNM 95277 (1, 48.0), USNM 95279 (6), USNM 95287 (1), USNM 95295 (11, 45.0–69.0), USNM 95302 (1), USNM 95303 (1), USNM 95304 (1), USNM 95305 (1), USNM 95306 (1), USNM 95307 (1) and USNM 95308 (1) (45.0–69.0), Endeavor Strait, Malampaya Sound, Taytay, Palawan Island, 3–6 m, 22–23 Dec. 1908; USNM 95280 (2), USNM 95284 (2), USNM 95292 (2), USNM 95313 (5) and USNM 95318 (5) (40.0–68.0), reef south of Agbatan Point, Romblon Harbor, Romblon, 2–5 m, 25–26 Mar. 1908; USNM 95281 (2), USNM 95294 (1) and USNM 95301 (2) (39.0–64.0), Port Matalvi, off west of Luzon, 22–23 Nov 1908; USNM 95285 (2) and USNM 95288 (3) (54.0–55.0), Bolalo Bay, Malampaya Sound, Taytay, Palawan Island, 21 Dec. 1908; USNM 95286 (4, 49.0–72.0), Candaraman Island, northern Balabac Strait, Palawan, 3–5 m, 4 Jan. 1909; USNM 95282 (1, 48.0) and USNM 95289 (1, 47.0), Port Busin, Burias Island, Luzon, 3–6 m, 7–8 Mar. 1909; USNM 95296 (5, 38.0–65.0), same locality as USNM 95282, 2–9 m, 23 Apr. 1908; USNM 95291 (1, 82.0), Santa Cruz Island, between Marinduque and Luzon islands, 24 Apr 1908; USNM 95293 (1, 59.0), Mantacao Island, western Bohol, 1908; USNM 95297 (1, 55.0), unnamed river, Nakoda Bay, southeast of Maricaban Island, Quezon, Palawan, 31 Dec 1908; USNM 95298 (1), Batan Island, east of Luzon, 5 Jun. 1909; USNM 95299 (1, 50.0), Ulugan Bay, Puerto Princesa, Palawan Island, 28–30 Dec. 1908; USNM 95309 (1, 45.0), Pujada Bay, Mindanao, 5 May 1908; USNM 95278 (1) and USNM 95310 (1) (42.0–64.0), Machesi Island, east of Palawan, 2–4 m, 5 Apr 1909; USNM 95311 (1, 56.0), Malcochin Harbor, Linapacan Island, Palawan, 19 Dec. 1908 (original description stated Port Uson in Busuanga Island); USNM 95314 (2, 62.0–68.0), Sacol Island, east of Zamboanga, Mindanao, 4–5 m, 9 Sep 1909; USNM 95315 (1), Philippines, 7 Nov. 1907; USNM 95316 (1, 59.0), Balicuatro Island, Biri Channel, east of Luzon, 3–4 m, 2 Jun. 1909; USNM 95317 (1, 50.0), San Miguel Harbor, between Burias and Luzon islands, 21 Apr. 1908;

**(1134) *Abudefdufbleekeri* Fowler & Bean, 1928: 165, pl. 15**

= *Chrysipterableekeri* (Fowler & Bean, 1928).

**Holotype.** Uncat. Albatross station 5145, Jolo Lighthouse, Sulu Archipelago.

**Paratypes.**ANSP 85278 [ex USNM 59378] (1) and USNM 93531 (3), same locality as holotype, 42 m, 15 Feb. 1908.

**Remarks.** The original description mentioned several specimens but did not specify which is the holotype and it was not found in the USNM record too. The species was originally proposed as a new name for several *Glyphidodontopscyaneus* (not Quoy and Gaimard). Based on the ledger, USNM 93531 previously contained five specimens. Based on ANSP’s record, ANSP 85278 specimen has identification notes of *Chrysipteraglyphidontops* and *Chrysipterarex*.

**(1135) *Abudefdufcoracinus* Seale, 1910: 520, pl. 13**

= *Neoglyphidodonnigroris* (Cuvier, 1830).

**Holotype.** BSMP 4908 (123.0), Sitanki Island, Sulu Archipelago, 18 Jul. 1908.

**Remarks.** The type specimen was presumed destroyed.

**(1136) *Abudefdufmelanocarpus* Fowler & Bean, 1928: 182**

= *Neopomacentrusazysron* (Bleeker, 1877).

**Holotype.**USNM 95680 (75.0), Cabugao Bay, Catanduanes Island, east of Luzon, 3–5 m, 9 Jun. 1909.

**Paratypes.**USNM 95678 (1, 73.0), same data as holotype; USNM 956779 (1, 63.0), Lianga Bay, Mindanao, 17 May 1908.

**Remarks.** The original description indicated USNM 89965 as the catalog number and 9 Jan. 1909 as the collection date of the holotype, but different from USNM record (Smithsonian Museum Archive 2024).

**(1137) *Abudefdufmelanopselion* Fowler, 1918: 59, fig. 23**

= *Hemiglyphidodonplagiometopon* (Bleeker, 1852).

**Holotype.**ANSP 47538 (93.0), Philippines.

**Remarks.** The original description or ANSP record did not provide a specific locality and collection date. A photograph and radiograph are available in ANSP’s record.

**(1138) *Abudefdufparasema* Fowler, 1918: 56, fig. 22**

= *Chrysipteraparasema* (Fowler, 1918).

**Holotype.**ANSP 47533 (43.0), Philippines.

**Paratypes.**ANSP 47534 [ex 47534–37] ((4, 37.0–43.0), same data as holotype.

**Remarks.** The holotype was mixed in a jar with the paratypes. The original description or ANSP record did not provide a specific locality and collection dates. Specimens from lots ANSP 47535–37 were fused into lot ANSP 47534. Photographs and radiographs of holotype are available in ANSP record.

**(1139) *Abudefdufphilippinus* Fowler, 1918: 54, fig. 21**

= *Amblyglyphidodonternatensis* (Bleeker, 1853).

**Holotype.**ANSP 47531 (41.0), Philippines.

**Paratype.**ANSP 47532 (1, 36.0), same data as holotype.

**Remarks.** The original description or ANSP did not provide a specific locality and collection dates. Photographs and radiographs of the holotype are available in ANSP’s record.

**(1140) *Abudefdufsapphirus* Jordan & Richardson, 1908: 264, fig. 10**

= *Chrysipteracyanea* (Quoy & Gaimard, 1825).

**Holotype.**CAS-SU 20207, Ticao Island, Luzon.

**Paratypes.**CAS-SU 20212 (2) and USNM 61682 (3), same data as holotype.

**Remarks.** The original description or CAS record did not indicate the collection date. A photograph and a radiograph of the holotype are available in the CAS record.

**(1141) *Abudefdufthoracotaeniatus* Fowler & Bean, 1928: 158, pl. 14**

= *Neoglyphidodonthoracotaeniatus* (Fowler & Bean, 1928).

**Holotype.**USNM 89964, Balicuatro Islands, Biri Channel, east of Luzon, 4–7 m, 1 Jun. 1909.

**Paratypes.**ANSP 80427 [ex USNM 95683] (5), Philippine Gov’t. [ex USNM 95683] (3), USNM 95689 (9, 48.0–87.0), same data as holotype.

**Remarks.**USNM 89964 and USNM 95689 were recorded as syntypes with two and nine specimens in the USNM record, respectively. USNM 95683 previously had 10 specimens: 5 to ANSP, 3 to Philippines, and 2 missing. It is unclear whether the specimens sent to the Philippines were deposited in PNM. USNM 95696 was previously listed as type specimen but was excluded by virtue of ICZN Article. 72.4.6.

**(1142) *Abudefdufturchesius* Jordan & Seale, 1907: 28, fig. 10**

= *Chrysipteracyanea* (Quoy & Gaimard, 1825).

**Holotype.**CAS-SU 9245 (50.8), Cavite, Luzon, 1 Jun. 1900.

**Remarks.** A photograph and a radiograph of the holotype are available in the CAS record.

**(1143) *Altrichthysalelia* Bernardi, Longo & Quiros, 2017: 48, figs 1–4**

**Holotype.**PNM 15195 (54.7), 12°11.47'N, 120°06.13'E, San José, Busuanga Island, Palawan, 3 m.

**Paratypes.**CAS 241438 (3, 51.0–54.1) and CAS 241439 (8, 13.9–19.7), taken with holotype.

**Remarks.** The collection date was not indicated in the original description.

**(1144) *Altrichthyscuratus* Allen, 1999: 24, figs 1–3A**

**Holotype.** UPMSI 1998-000002-FI, 10°51.20'N, 121°00.23'E, Cuyo Island, Palawan.

**Paratypes.** UPMSI 1998-000003-FI (10); WAM P.31400-001 (6), WAM P.31406-001 (3), WAM P.31407-001 (8); USNM 348456 (94, 16.1–40.5), same locality as holotype, 1 m, 21 May 1978.

**Remarks.** Fifteen of the original 109 specimens were removed for deposit at UP-MSI in Manila. However, only 11 such specimens are mentioned in the description. The collection date was not indicated in the original description.

**(1145) *Amphiprionboholensis* Cartier, 1874: 96**

= *Amphiprionclarkii* (Bennett, 1830).

**Holotype.** Uncat. (37.0), Bohol.

**Remarks.** The original description did not provide a catalog number and collection date. Whereabout is unknown (Parenti 2021).

**(1146) *Amphiprionsandaracinos* Allen, 1972: 81, figs 29–30**

**Holotype.**USNM 147130, Capulaan Bay, Pagbilao Chica Island, Marinduque, Luzon, 24 Feb. 1909.

**Paratypes.** BPBM 6804 (1), 16 March 1969; BPBM 10617 (3), 1 Jan. 1970; USNM 160663 (2), same data as holotype; USNM 160664 (2), Bolinao Bay, west of Luzon, 3–4 m, 10 May 1909.

**(1147) *Amphiprionthiellei* Burgess, 1981: 69**

**Holotype.**USNM 228445, Cebu Island.

**Paratype.** WAM P.30191-001 (1, 39.0), same locality as holotype.

**Remarks.**Hutchins and Smith (1991) reported that the paratype was uncataloged. It was reported in the original description as being deposited in WAM but could not be found and they presumed lost (Moore et al. 2008). The original description nor USNM record did not indicate the collection date of the holotype.

**(1148) *Chromisalpha* Randall, 1988: 74, fig. 1**

**Holotype.** BPBM 14993. Tetiaroa Atoll, Society Islands.

**Paratypes.** BPBM 18467 (3, 70.5–75.9), off marine laboratory of University of San Carlos, Mactan Island, Cebu, 30 m, 25 Jun. 1975; USNM 273546 (1, 76.0), 09°44.00'N, 118°45.00'E, Puerto Princesa, Palawan Island, 6–12 m, 2 Jul. 1979.

**(1149) *Chromisatripes* Fowler & Bean, 1928: 43, pl. 2**

= *Pycnochromisatripes* (Fowler & Bean, 1928).

**Holotype.**USNM 89952, Sablayan, Mindoro Island, 12 Dec. 1908.

**Paratypes.**USNM 97045 (6) and USNM 98525 (10), (33.0–65.0), taken with holotype; USNM 98470 (27), USNM 98472 (1), USNM 98479 (2) and USNM 98480 (1) (38.0–67.0), Tapiantana Island, south of Zamboanga, Mindanao, 2–3 m, 13 Sep. 1909; USNM 98473 (2, 70.0), Romblon reef, Romblon, 2–6 m, 26 Mar. 1909; USNM 98474 (2, 58.0–63.0), Limbones Cove, Manila Bay, Luzon, 8 Feb. 1909; USNM 98475 (1, 64.0), Port Maricaban, southern Luzon, 4–6 m, 21 Jul. 1908; USNM 98482 (1, 65.0), Singaan Island, between Jolo and Tawi-Tawi islands, Sulu Archipelago, 3–4 m, 21 Sep. 1909; USNM 98486 (1) and USNM 98521 (2) (53.0–68.0), Little Santa Cruz Island, Zamboanga, Mindanao, 2–9 m, 28 May 1908; USNM 98490 (1) and USNM 98507 (1, 64.0), Tataan Island, Tawi-tawi Group, Sulu Archipelago, 2 and 20 Feb. 1908; USNM 98493 (1, 66.0), Tutu Bay, First Anchorage, Jolo Island, Sulu Archipelago, 3–6 m, 19 Sep. 1909; USNM 98491 (3) and USNM 98511 (1) (49.0–75.0), Philippines, 7 Nov. 1907; USNM 98477 (1, 63.0) and USNM 98495 (2, 40.0–70.0), Port Galera, Galera Bay, Mindoro Island, 9 Jun. 1908; USNM 98492 (1, 77.0), same locality as USNM 98477, 27 Oct. 1909; USNM 98498 (1, 67.0), Tinakta Island, Tawi-Tawi Group, Sulu Archipelago, 22 m, 22 Feb. 1908; USNM 98502 (2, 52.0–65.0), Paluan Bay, Mindoro Island, 11 Dec. 1908; USNM 98503 (1) and USNM 98515 (1) (57.0–64.0), Polloc (Marigabato Point), Illana Bay, Mindanao, 1–8 m, 22 May 1908; USNM 98518 (1), Bulan Island, south of Zamboanga, Mindanao, 13 Sep. 1909; USNM 98519 (2, 69.0–73.0), Port San Pio Quinto, Camiguin Island, Mindanao, 10 Nov. 1908; USNM 98508 (3, 65.0–66.0), Wreck Bay, Dalaganem Island, Araceli, Palawan, 4–5 m, 8 Apr. 1909; USNM 98481 (1, 60.0), Simaluc Island, north of Tawi-Tawi, 2–5 m, 22 Sep. 1909.

**Remarks.** The holotype was previously mixed with other specimens; some specimens were removed to USNM 98525 (10) and 97045 (6). Some specimens are mixed and cannot be separated according to USNM records: [1] 98470 (27), 98472 (1), and 98480 (1); [2] 98489 (2) and 98511 (1); and [3] 98503 (1) and 98515 (1).

**(1150) *Chromisazurelineatus* Fowler & Bean, 1928: 57, pl. 5**

= *Altrichthysazurelineatus* (Fowler & Bean, 1928).

**Holotype.**USNM 89957, Port Uson, west of Pinas Island, Busuanga, Palawan, 17 Dec. 1908.

**Paratypes.**ANSP 80317 [ex USNM 96398] (3); USNM 96397 (12) and USNM 96424 (6), same data as holotype; USNM 96398 (4.),USNM 96425 (1), (38.0–90.0), Tara Island, Coron, Palawan, 15 Dec. 1908; USNM 96399 (5, 50.0–62.0), Candaraman Island, north of Balabac Strait, Palawan, 3–5 m, 4 Jan. 1909.

**Remarks.** Specimens in USNM 96397 and 96424 are mixed and cannot be separated based on USNM records. USNM 96398 previously had 10 specimens: 3 to ANSP, 3 to P.I. (possibly Philippine Island).

(**1151) *Chromisbitaeniatus* Fowler & Bean, 1928: 56, pl. 6**

= *Neoglyphidodonnigroris* (Cuvier, 1830).

**Holotype.**USNM 89956 (45.0), Maricaban Island, Balayan Bay and Verde Island Passage, Luzon, 2–6 m, 20 Jan. 1908.

**(1152) *Chromisbowesi* Arango, Pinheiro, Rocha, Greene, Pyle, Copus, Shepherd & Rocha, 2019: 9, figs 3a–c**

**Holotype.**PNM 15359 (82.1), 13°480.60'N, 120°54.65'E, Dive and Trek, Batangas, Luzon, 120 m, 12 Dec. 2013.

**Paratypes.** BPBM 41350 (77.5), same data as holotype; USNM 440406 (1, 78.3), same locality as holotype, 14 Dec. 2013; CAS 242324 (1, 66.0), Puerto Galera Bay, Mindoro Island, 105 m, 9 Apr 2015; CAS 242278 (77.5), Verde Island, Batangas, Luzon, 110 m, 12 Apr. 2015.

**(1153) *Chromisdelta* Randall, 1988: 78, fig. 3**

= *Pycnochromisdelta* (Randall, 1988).

**Holotype.** BPBM 15584. Guadalcanal, Solomon Island.

**Paratype.** BPBM 18469 (2, 34.7–41.0), off marine laboratory of University of San Carlos, Mactan Island, Cebu, 28–35 m, 26–27 Jun. 1975; BPBM 6512 (2, 19.1–26.3), off south end of Dumaguete City, Negros Island, 22 m, 9 Aug. 1978; BPBM 26527 (1, 40.4), Caban Island, Batangas, Luzon 45 m, 13 Aug. 1978.

**(1154) *Chromisdesmostigma* Fowler & Bean, 1928: 51**

= *Acanthochromispolyacanthus* (Bleeker, 1855).

**Holotype.**USNM 89954, Tataan Island, Tawi Tawi Group, Sulu Archipelago, 21 Feb. 1908.

**Paratypes.**USNM 93520 (3) and USNM 98241 (2), same data as holotype.

**(1155) *Chromiselerae* Fowler & Bean, 1928: 52, pl. 4**

= *Azurinaelerae* (Fowler & Bean, 1928).

**Holotype.**USNM 89955 (45.0), Pasacao, Ragay Gulf, Luzon, 9 Mar. 1909.

**Paratypes.**ANSP 85281 [ex USNM 98717] (1); USNM 98720 (1); USNM 232920 [ex 89955] (1), same data as holotype; USNM 98716 (1, 48.0), Balicuatro Island, Biri Channel, east of Luzon, 4–7 m, 1–2 Jun. 1909; USNM 98718 (1, 17.0), Ligpo Point Reef, between Balayan Bay and Verde Island Passage, 2–6 m, 18 Jan. 1908; USNM 98719 (1, 60.0), Refugio Island, Pasacao Anchorage, Ragay Gulf, Luzon, 9 Mar. 1909.

**Remarks.**USNM 89955 was previously comprised of two specimens but one was re-cataloged as USNM 242920.

**(1156) *Chromisgunting* Arango, Pinheiro, Rocha, Greene, Pyle, Copus, Shepherd & Rocha, 2019: 3, figs 1a–c**

**Holotype.**PNM 15357 (67.7), 13°41.38'N, 120°50.21'E, Layag-Layag Point, Batangas, Luzon, 6 Dec. 2013.

**Paratype.**CAS 242328 (1, 77.4), Puerto Galera, Mindoro Island, 100 m, 9 Apr. 2015.

**(1157) *Chromishangganan* Arango, Pinheiro, Rocha, Greene, Pyle, Copus, Shepherd & Rocha, 2019: 6, figs 2a–b**

**Holotype.**PNM 15358 (57.8), 13°45.75'N, 120°07.64'E, Lubang Island, Batangas, off west of Luzon, 130 m, 16 May 2014.

**Paratype.**CAS 243205 (1, 47.9), same data as holotype.

**(1158) *Chromislineatus* Fowler & Bean, 1928: 50, pl. 3**

= *Pycnochromislineatus* (Fowler & Bean, 1928).

**Holotype.**USNM 89953 (53.0), Pangasinan Island, Jolo, Sulu Archipelago, 2–4 m, 13 Feb. 1908.

**Paratype.**USNM 93530 (1), Alimango Bay, Burias Island, Masbate, 4–7 m, 5 Mar. 1909.

**(1159) *Chromisphilippinus* Fowler, 1918: 63, fig. 25**

= *Chromisternatensis* (Bleeker, 1856).

**Holotype.**ANSP 47541 (70.0), Philippines.

**Paratypes.**ANSP 47542 [ex 47542–47] (6, 58.0–75.0), same data as holotype.

**Remarks.** The original description or ANSP record did not provide a specific locality and collection date.

**(1160) *Chromisscotochiloptera* Fowler, 1918: 61, fig. 24**

**Holotype.**ANSP 47539 (115.0), Philippines.

**Paratype.**ANSP 47540 (1, 75.0), same data as holotype.

**Remarks.** The original description or ANSP record did not provide a specific locality and collection date. A photograph and a radiograph of the holotype are available in ANSP’s record.

**(1161) *Chromisweberi* Fowler & Bean, 1928: 41, pl. 1**

**Holotype.**USNM 72713. Java, Indonesia.

**Paratypes.**USNM 96955 (4) and USNM 98242 (3), (90.0–110.0), 2–5 m, Panpan Point, Tara Island, between Jolo and Tawi-Tawi islands, Sulu Archipelago, 20 Sep. 1909 (specimens cannot be separated).

**Remarks.** The holotype was re-identified as *Chromiscinerascens*, while USNM 96955 and 98242 (mixed and cannot be separated) was re-identified as *Chromisopercularis* by G.R. Allen. He also noted that the holotype does not agree with other paratypes (Smithsonian Museum Archive 2024). The materials in the type catalogs of Böhlke (1984) and Ibarra and Stewart (1987) were regarded as non-types.

**(1162) *Dascylluscaudofasciatus* Montalban, 1928: 26, pl. 6 (fig. 1)**

= *Azurinalepidolepis* (Bleeker, 1876).

**Syntypes.** BSMP (13, 45.0–54.0); Tambagaan, Bungau, and Sibutu Islands, Sulu Archipelago, Mindanao.

**Remarks.** All type specimens were presumed destroyed.

**(1163) *Glyphidodonmutabilis* Cartier, 1874: 100**

**Syntypes.** Uncat. (4, 114.3–190.5), Cebu Island.

**Remarks.** The original description did not provide catalog numbers and collection date. The type specimens cannot be located. Unknown status in Fricke et al. (2024). Further study is needed to verify its status.

**(1164) *Glyphidodontaenioruptus* Cartier, 1874: 101**

= *Chrysipteraleucopoma* (Cuvier, 1830).

**Holotype.** Uncat. (177.8), Bohol Island.

**Remarks.** The original description did not provide catalog numbers and collection date. The type specimens cannot be located.

**(1165) *Pomacentrusalbolineatus* Montalban, 1928: 70, pl. 14 (fig. 1)**

**Lectotype.**CAS-SU 25571 (37.0), Bungau Island, Tawi-Tawi, Sulu Archipelago, 18 Jun. 1921.

**Paralectotypes.** BSMP (1, 37.0), same data as lectotype.

**Remarks.** Two specimens (CAS-SU and BSMP) were considered syntypes. BSMP specimen was apparently destroyed and a lectotype was designated by Böhlke (1953). A photograph and a radiograph of the lectotype are available in the CAS record. Uncertain status (Allen 1991; Parenti 2021) and further study is needed to verify its status.

**(1166) *Pomacentrusalexanderae* Evermann & Seale, 1907: 90, fig. 17**

**Holotype.**USNM 55919 (88.9), Bacon, Sorsogon, Luzon.

**Paratypes.**ANSP 33296 [ex USBF 3996] (1), BSMP (1), CAS 79635 [ex Bur. Fish. 3996, IU 11594] (1), CAS-SU 20005(1), MCZ 37285 [ex USNM 126403] (1, 65,0), USNM 55922 (1) and USBF 4540 (1) (82.6–88.9), same data as holotype.

**Remarks.** The original description or USNM record did not indicate the collection date of the holotype. BSMP type specimen presumed destroyed.

**(1167) *Pomacentrusbeauforti* Fowler & Bean, 1928: 115, pl. 13**

= *Amblypomacentrusbreviceps* (Schlegel & Müller, 1840).

**Holotype.**USNM 89962, Catbalogan, Samar Island, 15 Apr. 1908.

**Paratypes.**ANSP 80291 [ex USNM 97209] (1) and USNM 97209 (4, 40.0–66.0), Cebu Market, 28 Mar. 1909; ANSP 80318 [ex USNM 97211] (4), BPBM 10558 [ex ANSP 80318] (1), USNM 97911 (1) and USNM 97211 (97, 23.0–87.0) and USNM 437432 (1) [ex USNM 97211], same data as holotype; USNM 97208 (1, 58.0) and USNM 97210 (4, 48.0–53.0), Cataingan Bay, east of Masbate Island, 2–3 m, 18–19 Apr. 1908; USNM 97212 (3, 27.0–37.0), Ragay Gulf, Luzon, 1–6 m, 10 Mar. 1909.

**Remarks.** Some specimens in USNM 97211 were sent to other museums: 5 to ANSP, 5 to P.I., 1 specimen removed for tissue sampling and re-cataloged as 437432.

**(1168) *Pomacentrusburroughi* Fowler, 1918: 48, fig. 19**

**Holotype.**ANSP 47526 (70.0), Philippines.

**Paratyes.**ANSP 47527 (1, 57.0), same data as holotype.

**Remarks.** The original description or ANSP record did not provide a specific locality and collection date. A photograph and a radiograph of the holotype are available in ANSP’s record.

**(1169) *Pomacentruscheraphilus* Allen, Erdmann & Hilomen, 2011: 36, figs 1–3**

**Holotype.** WAM P.33030-005. East Brunei.

**Paratypes.** UPLB Z-NS 0491–0500 (9), Philippines.

**Remarks.** The original description did not provide a specific locality nor collection dates for paratypes from the Philippines.

**(1170) *Pomacentrusdelurus* Jordan & Seale, 1905: 783, fig. 6**

= *Pomacentrusbankanensis* Bleeker, 1854.

**Holotype.**USNM 52052 (38.1), south of Negros Island, 1901.

**Paratype.**CAS-SU 9130 (1), same data as holotype.

**Remarks.** The original description mentioned USNM 51 as the type specimen.

**(1171) *Pomacentruselongatus* Seale, 1910: 518, pl. 12 (fig. 2)**

= *Pomacentrustripunctatus* Cuvier, 1830.

**Holotype.** BSMP 2214 (765.0), Limbones Cove at the entrance to Manila Bay, Luzon, 14 Jan. 1908.

**Paratypes.** BSMP 2212 (1) and BSMP 2213 (1), same data as holotype.

**Remarks.** All type specimens were presumed destroyed.

**(1172) *Pomacentrusgrammorhynchus* Fowler, 1918: 44, fig. 17**

**Holotype.**ANSP 47518 (115.0), Philippines.

**Remarks.** The original description did not provide a specific locality and collection date. A photograph and a radiograph are available in ANSP’s record.

**(1173) *Pomacentrushebardi* Fowler, 1918: 46, fig. 18**

= *Pomacentrussimsiang* Bleeker, 1856.

**Holotype.**ANSP 47519 (56.0), Philippines.

**Paratypes.**ANSP 47520 [ex ANSP 47520–25] (6, 43.0–54.0), same data as holotype.

**Remarks.** The original description or ANSP record did not provide a specific locality and collection date. Type specimens were mixed in the same jar. One of the paratypes was identified as *Pomacentrusmollucensis*. A photograph of the holotype is available in ANSP record.

**(1174) *Pomacentruslepidogenys* Fowler & Bean, 1928: 98, pl. 8**

**Holotype.**USNM 89959 (68.0), Ligpo Point, Balayan Bay, Batangas, Luzon, 2–6 m, 18 Jan 1908.

**Paratypes.** Uncat. (2, 59.0–61.0), Batan Island, Batanes, 5 Jun 1909.

**Remarks.** The original description or USNM record did not provide catalog numbers for paratypes.

**(1175) *Pomacentrusopisthostigma* Fowler, 1918: 51, fig. 20**

**Holotype.**ANSP 47528 (65.0), Philippines.

**Paratypes.**ANSP 47529 [ex ANSP 47529–30] (2, 41.0–61.0), same data as holotype.

**Remarks.** The original description or ANSP did not provide a specific locality. A photograph and a radiograph of the holotype are available in ANSP’s record.

**(1176) *Pomacentrusovoides* Cartier, 1874: 98**

**Syntypes.** Uncat. (2, 228.6–254.0), Bohol Island and Cavite, Luzon.

**Remarks.** The original description did not provide catalog numbers and collection date. Unknown status in Fricke et al. (2024). Further study is needed to verify its status.

**(1177) *Pomacentrusphilippinus* Evermann & Seale, 1907: 91, fig. 18**

**Holotype.**USNM 55901 (63.5), Bacon, Sorsogon, Luzon.

**Paratype.**CAS-SU 20009 (1, 69.85), same data as holotype.

**Remarks.** The original description or USNM record did not indicate the collection date.

**(1178) *Pomacentruspopei* Evermann & Seale, 1907: 90, fig. 16**

= *Pomacentrusmoluccensis* Bleeker, 1853.

**Holotype.**USNM 55903 (62.2), Bacon, Sorsogon, Luzon.

**Remarks.** The original description or USNM record did not indicate the collection date.

**(1179) *Pomacentruspunctatolineatus* Cartier, 1874: 98**

= *Pomacentrustripunctatus* Cuvier, 1830.

No type specimen is known. Bohol Island.

**Remarks.** The original description mentioned three specimens measuring 46.0–57.0.

**(1180) *Pomacentrusreidi* Fowler & Bean, 1928: 99, pl. 9**

**Holotype.**USNM 89960 (115.0), Tataan Island, Tawi Tawi Group, Sulu Archipelago, 2 Feb. 1908.

**Paratypes.**ANSP 85294 [ex USNM 98752] (2), Biri Channel, 1 Jun. 1909; FMNH 62626 (2, 73.0–80.0), Dalanganem Island, Araceli, Palawan, 8 Apr. 1909; USNM 98744 (1) and USNM 98745 (1) (47.0–68.0), Port Palapag, Batag Island, Samar, 2–5 m, 3 Jun. 1909; USNM 98746 (1), 4–7 m, Alimango Bay, Burias Island, 5 Mar 1909; USNM 98747 (1, 70.0), Nasugbu Bay, off south of Luzon, 2–5 m, 16 Jan. 1908; USNM 98758 (5) and USNM 98760 (1) (60.0–75.0), Albay Gulf, between Paron and Jesus Point, east of Luzon, 3 m, 21 Jun. 1909; USNM 98761 (1, 73.0), Philippines, 7 Nov 1907; USNM 98762 (1) and USNM 98768 (1) (55.0–64.0), Balayan Bay and Verde Island Passage, Batangas, Luzon, 2–6 m, 18 Jan. 1908; USNM 98765 (1, 61.0), Bugsuk Island, Balabac, Palawan, 3–5 m, 5 Jan. 1909; USNM 98766 (1, 70.0), Tara Island, Coron, Palawan, 15 Dec. 1908; USNM 98770 (1, 67.0), Casiguran Island, between Samar and Leyte islands, 3–5 m, 27 Jul. 1909; USNM 98764 (0), Batan Island, Caracaran Bay, east of Luzon, 2–3 m, 8 Jun. 1909 (specimens not found during 1985 inventory); USNM 98751 (1) and USNM 98752 (7) (58.0–75.0), Balicuatro Island, Biri Channel, west of Luzon, 4–7 m, 1–2 Jun. 1909; USNM 98753 (1) and USNM 98754 (1) (68.0), Matnog Bay, east of Luzon, 3–4 m, 31 May 1909; USNM 98756 (1), USNM 98763 (1) and USNM 98767 (2), Pujada Bay, Davao, Mindanao, 15 May 1908; USNM 98771 (3, 65.0–70.0), Porongpong Island, Palumbanes, east of Luzon, 2–3 m, 11 Jun. 1909.

**Remarks.** Some paratypes (USNM 98751–54, 98756, 98763, 98767, and 98771) were re-identified as *Pomacentruslepidogenys* by G.R. Allen in 1976 (Smithsonian Museum Archive 2024).

**(1181) *Pomacentrussmithi* Fowler & Bean, 1928: 75, pl. 7**

**Holotype.**USNM 89958, Biri Channel, Balicuatro Island, northern Samar, 4–7 m, 1–2 Jun. 1909.

**Paratypes.**USNM 97979 (12), USNM 97980 (1) and USNM 97984 (1) (44.0–67.0), same data as holotype; ANSP 80431 [ex USNM 97972] (2), Mantacao Island, west coast of Bohol; ANSP 80432 [ex USNM 97979] (3), Biri Channel, 1 Jun. 1909; USNM 97968 (1, 60.0), Cataingan Bay, Masbate Island, 2–3 m, 17 Apr. 1908; USNM 97969 (1, 47.0), Rasa Island, Narra, Palawan Island, 2–4 m, 1 Apr 1909; USNM 97971 (3, 67.0–72.0), Balabac Island, Palawan, 5 Jan. 1909; USNM 97972 (6, 38.0–60.0), Mantacao Island, Bohol, 8 Apr. 1908; USNM 97973 (1, 63.0), Tataan Island, Sulu Archipelago, 20 Feb. 1908; USNM 97974 (3, 43.0–58.0), South Point reef, Pangasinan Island, Jolo, Sulu Archipelago, 2–4 m, 13 Feb. 1908; USNM 97976 (1, 40.0), Isabel Channel, Basilan Island, 3–9 m, 11 Sep. 1909; USNM 97977 (1, 58.0), Sablayan, Mindoro Island, 12 Dec. 1908; USNM 97978 (13, 40.0–64.0), Cataingan Bay, Masbate Island, 18 Apr. 1908 ; USNM 97981 (1, 48.0), Romblon Reef, Romblon, 26 Mar. 1908; USNM 97986 (1, 65.0), Tara Island, Coron, Palawan, 3–6 m, 15 Dec. 1908.

**(1182) *Pomacentrusstigma* Fowler & Bean, 1928: 100, pl. 10**

**Holotype.**USNM 89961, Romblon Harbor, Romblon, 2–5 m, 25 Mar. 1908.

**Paratypes.**USNM 97965 (19, 75.0–110.0), same locality as holotype, 2–5 m, 25 Mar. 1908 ; ANSP 80161 [ex USNM 97956] (1), Port Jamelo, Luzon, 13 Jul. 1908; FMNH 62627 (1, 105.0), Paluan Bay, Mindoro Island, 11 Dec 1908; USNM 89961 (1 of 2) and USNM 97939 (2, 98.0–110.0), Canimo Island, near Daet, east of Luzon, 15 Jun. 1909; USNM 97941 (1) and USNM 97959 (1) (85.0–93.0), Port Matalvi, Luzon, 23 Nov. 1908; USNM 97946 (1, 112.0), Inamucan Bay, northern Mindanao, 2–5 m, 8 Aug. 1909; USNM 97947 (1, 110.0), Ulugan Bay, Puerto Princesa, Palawan Island, 28 Dec. 1908; USNM 97948 (4), USNM 97955 (1) and USNM 97956 (6) (54.0–114.0), Port Jamelo, south Luzon, 13 Jul. 1908; USNM 97944 (1, 53.0) and USNM 97949 (2, 95.0–114.0), Port Busin, Burias Island, 3–5 m, 7 Mar. 1909; USNM 97940 (1), USNM 97950 (4), USNM 97963 (1) and USNM 97966 (1) (90.0–98.0), Masamat Bay, Quinalasag Island, east of Luzon, 3 m, 12 Jun. 1909; USNM 97951 (2, 74.0–84.0), Limbones Cove, Manila Bay, Luzon, 8 Feb. 1909; USNM 97952 (1, 97.0), Tutu Bay, 1^st^ Anchorage, Jolo Island, Sulu Archipelago, 1–6 m, 19 Sep. 1909; USNM 97958 (1, 108.0), Anchorage, Dupon Bay, Leyte Island, 17 Mar. 1909; USNM 97960 (1) and USNM 97964 (3) (108.0–112.0), Endeavor Strait, Malampaya Sound, Taytay, Palawan Island, 22 Dec. 1908; USNM 97962 (1, 110.0), Port Caltom, Busuanga Island, Palawan, 15 Dec. 1908; USNM 98826 (1, 102.0) and USNM 97954 (1) San Miguel Harbor, between Burias and Luzon islands, 21 Apr. 1908.

**Remarks.** The collection date of holotype was not indicated in the original description. The USNM record indicates 2 specimens for USNM 89971. USNM 98826 and USNM 97954 may be the same specimen (Smithsonian Museum Archive 2024). Mixed specimens and cannot be separated based on USNM records: 97941 and 97959; 97940, 97950, 97963 and 97966; 97948, 97955 and 97956; 97960 and 97964.

**(1183) *Pomacentrussuluensis* Seale, 1910: 519**

= *Dischistodusmelanotus* (Bleeker, 1858).

**Holotype.** BSMP 4689 (355.0) Sitanki Island, Sulu Archipelago, 12 Jul. 1908.

**Remarks.** The type specimen was presumed destroyed.

**(1184) *Pomacentrustablasensis* Montalban, 1928: 72, pl. 14 (fig. 2)**

= *Pomacentrusstigma* Fowler & Bean, 1928.

**Holotype.** BSMP (95.0), Tablas Island, Romblon.

**Remarks.** The collection date was not indicated in the original description. The type specimen was presumed destroyed.

**(1185) *Pomacentrustropicus* Seale, 1910: 517, pl. 12 (fig. 1)**

= *Pomacentrussimsiang* Bleeker, 1856.

**Holotype.** BSMP 4737 (75.0), Sitanki Island, Sulu Archipelago, 15 Jul. 1908.

**Paratypes.** BSMP 4736 (5), same data as holotype.

**Remarks.** All type specimens were presumed destroyed.

##### Family Pseudochromidae (289)

**(1186) *Congrogadushierichthys* Jordan & Richardson, 1908: 285, fig. 11**

**Holotype.**CAS-SU 20208, Cuyo Island, Palawan.

**Paratype.**USNM 61684 (1), same data as holotype.

**Remarks.** The original description mentioned two sizes measuring 76.2 and 101.6 but did not specify the size of each type. Böhlke (1953) also did not mention the size of the holotype in his catalog. The original description or CAS record did not indicate the collection date of the holotype. A photograph and a radiograph of the holotype are available in the CAS record.

**(1187) *Cyphozaps* Gill, 2004: 20, figs 8–10; pl. 1G**

**Holotype.**USNM 291625 (35.4) White Beach, Batan Island, Batanes, 15–21 m, 2 May 1987.

**Paratypes.**USNM 304577 [ex USNM 291625] (7, 33.6–37.5), same data as holotype; USNM 291602 (3, 39.3–43.3), Y’ami Island, Batanes, 15–19.5 m, 26 Apr. 1987; AMS I.39543-001 (4, 33.7–38.3), NMST-P 46416 (1, 34.6), USNM 291601 (9, 30.4–40.6) and USNM 291627 (3, 42–43.1), White Beach, Batan Island, Batanes, 9–12 m, 22 Apr. 1987 – 1 May 1987; ANSP 163457* (6, 39.7–43.8), Chawa Point, Batan Island, Batanes, 1 May 1987; USNM 291626 (2, 31.7–38) and USNM 293355 (1, 36.8), Sabtang Island, Duvuck Bay, Batanes, 18–24 m, 3–4 May 1987; USNM 339204 (1, 31.2), Balingasay Reef, off Bolinao, Pangasinan, Luzon, 21–24 m, 9 Oct. 1995.

**Remarks.**USNM 291625 originally contained 14 specimens: seven were removed as paratypes to USNM 304577, and six were removed as non-types to USNM 304578. (*Based on ANSP’s record, 163457 contains two specimens of *Hypostomus* sp. from Venezuela). There may be an error in the original description.

**(1188) *Dampieriaatrofasciatus* Herre, 1933: 17**

= *Labracinusatrofasciatus* (Herre, 1933).

**Holotype.**CAS-SU 25518 (105.0), Culion Island, Palawan, Apr. 1931.

**Remarks.** The original description or CAS record did not indicate the collection date.

**(1189) *Dampieriabitaeniata* Fowler, 1931: 18, fig. 4**

= *Pseudochromisbitaeniatus* (Fowler, 1931).

**Holotype.**USNM 89990, Port Galera, Galera Bay, Mindoro Island, 9 Jun. 1908.

**Paratype.**USNM 144578 (1, 67.0), Port Caltom, Busuanga Island, Palawan, 1 m, 15 Dec. 1908.

**Remarks.** The original description mentioned three paratypes: one specimen (67.0) from Santa Cruz, Marinduque has a tag number 220703, but USNM record indicated this tag belongs to USNM 144578. Radiographs of the holotype are available in the USNM record (Smithsonian Museum Archive 2024).

**(1190) *Dampieriamelanostigma* Fowler, 1931: 16, figs 2, 3**

= *Labracinuscyclophthalmus* (Müller & Troschel, 1849).

**Holotype.**USNM 89989, Langao Point, east of Luzon, 2–5 m, 24 Jun. 1909.

**Paratypes.**USNM 146416 (4, 150.0–160.0), Burias Island, Alimango Bay, Masbate, 4–7 m, 5 March 1909; USNM 146418 (1, 173.0), Camahala Bay, between Burias and Luzon islands, 1–9 m, 11 Mar. 1909; USNM 146419 (2, 150.0–158.0), Saboon Island, Ragay Gulf, Luzon, 1–6 m, 10 Mar. 1909; USNM 146421 (1, 181.0), Mactan Island, 25 Mar. 1909; USNM 146422 (2, 112.0–150.0), Alinijaban Island, Ragay Gulf, Luzon, 4–9, 6 Mar. 1909; USNM 146423 (1, 133.0) and USNM 146417 (1, 149.0), Port Busin Burias Island, 3–6 m, 7–8 Mar. 1909; ANSP 101790 (1), Cebu Market, Cebu Island, 4 Apr. 1908.

**Remarks.** The original description did not mention the size of the holotype and was also not found in USNM record. Radiographs of the holotype are available in the USNM record.

**(1191) *Labracinusflavipinnis* Seale, 1910: 530**

= *Pseudochromistapeinosoma* Bleeker, 1853.

**Holotype.** BSMP 4410 (46.0), Zamboanga, Mindanao, 1 Jun. 1908.

**Remarks.** The type specimen was presumed destroyed.

**(1192) *Manonichthysscintilla* Gill & Williams, 2011:54, figs 5, 6**

**Holotype.**PNM 15176 [ex USNM 382743] (27.4), Apo Reef, Mindoro Island, 5–33 m, 3 Mar. 2003.

**Paratypes.**USNM 382744 (1, 31.5), same locality as holotype, 2–17 m, 2 Mar. 2003; ASU 19100 (1, 28.8) and FMNH 120315 (2, 23.6–24.6), Coron Island, Palawan, 15–31 m, 7 Mar. 2003.

**(1193) *Manonichthyswinterbottomi* Gill, 2004: 42, figs 14, 15, 17**

**Holotype.** ROM 52857 (70.0), north of Bais Bay near the main channel, Cebu Strait, 18–35 m, 19 May 1987.

**Paratypes.**BMNH 1979.1.9.32–34 (3, 30.1–58.5) Moalboal, southwest of Cebu, 8–10 m, 7–8 Aug. 1976.

**Other catalog number.**BMNH 1979.1.9.32–34 (NHMUK:ecatalogue:2545202).

**Remarks.** The original description mentioned the paratype having catalog number BMNH 1979.1.9.32–35 having 4 specimens but NMH’s record indicated BMNH 1979.1.9.32–3 with 3 specimens.

**(1194) *Pseudochromisaurea* Seale, 1910: 528**

= *Pseudochromisfuscus* Müller & Troschel, 1849.

**Neotype.** AMS I. 31423-001 [ex CAS-SU 28507] (32.9), Sitankai Island, Sulu Archipelago, 7 Aug. 1931.

**Remarks.** The original type specimens were destroyed during World War II (Herre, 1953a) and a neotype was designated by Gill (2004).

**(1195) *Pseudochromiscolei* Herre, 1933: 18**

**Holotype.**CAS-SU 30974 (59.0), Culion Island, Palawan.

**Remarks.** The original description or CAS record did not indicate the collection date.

**(1196) *Pseudochromiseichleri* Gill, Allen & Erdmann, 2012: 33, fig. 5**

**Holotype.** WAM P.31398-014 (68.3), 11°57'N, 119°50'E, off the southeast tip of Galoc Island, Culion, Palawan, 10–20 m, 12 Feb. 1998.

**Paratypes.** AMS I.45651-001 (1, 56.4), south of Dilumacad Island, El Nido, 20 m, 6 Jun. 2007; WAM P.32997-001 (1, 58.2), Daracotan Bay, El Nido, 30 m, 7 Jun. 2008; WAM P.33003-006 (2, 50.8–57.2), Tigpuro Ang Bobog, El Nido, 25 m, 9 Jun. 2008; all from Palawan Island.

**(1197) *Pseudochromisfowleri* Herre, 1934: 45**

**Holotype.**CAS-SU 28510 (44.0 TL), tide pool near Dumaguete, Negros Island, 22 Jun. 1931.

**Remarks.** A photograph and a radiograph of the holotype are available in the CAS record.

**(1198) *Pseudochromisfuligifinis* Gill & Williams, 2011: 50, figs 1–4**

**Holotype.**PNM 15177 [ex USNM 383120)] (32.0), 13°31.97'N, 121°06.18'E, Verde Island, Mindoro, 27–30 m, 28 May 2000.

**Paratypes.** AMS I.40141-002 (1, 30.9), FMNH 120316 (1, 32.1) and USNM 383121 (1, 31.9), taken with holotype; ASU 19098 (1, 23.8), ASU 19099 (1, 26.0), FMNH 120317 (3, 13.5–20.4) and USNM 382745 (2, 23.1–32.3), Apo Reef, Mindoro Island, 5–33 m, 3 Mar. 2003; USNM 383122 (1, 24.4), Mamburao Reef, Mindoro Island, 30 m, 3 Jun. 2000.

**(1199) *Pseudochromismoorei* Fowler, 1931: 39, fig. 6**

**Holotype.**USNM 89993 (100.0), Albatross station 5146, Sulade Island, Siasi, Sulu Archipelago, 16 Feb. 1908.

**Paratypes.**USNM 144576 (1, 62.0), Albatross station 5254, Linao Point, Davao Gulf, Mindanao, 38 m, 18 May 1908; USNM 144577 (1, 68.0), Albatross station 5163, Observation Island, Tawi Tawi Islands, Sulu Archipelago, 51 m, 24 Feb. 1908.

**Remarks.** Radiographs of the type specimens are available in the USNM record.

**(1200) *Pseudochromisrex* Seale, 1910: 529**

= *Pseudochromisperspicillatus* Günther, 1862.

**Holotype.** BSMP 4631 (120.0), Sitanki Island, Sulu Archipelago, 4 Jul. 1908.

**Paratypes.** BSMP (numerous).

**Remarks.** All type specimens were presumed destroyed.

**(1201) *Pseudochromissimilimus* Herre, 1933: 19**

= *Pseudochromisperspicillatus* Günther, 1862.

**Holotype.**CAS-SU 25517 (44.0), Culion Island, Palawan, Apr. 1931.

**Remarks.** A photograph and a radiograph of the holotype are available in the CAS record.

**(1202) *Pseudochromisstriatus* Gill, Shao & Chen, 1995: 79, fig. 1**

**Holotype.**USNM 291616 (female, 31.9), White Beach (20°24.75'N, 121°55.00'E), Batan Island, Batanes, 15–21 m, 2 May 1987.

**Paratypes.** ASIZT P.057128 (1) and USNM 304579 (1), same data as holotype.

**(1203) *Pseudochromisswaini* Herre, 1934: 46**

= *Pseudochromisfowleri* Herre, 1934.

**Holotype.**CAS-SU 26772, a reef in Culion Harbor, Culion Island, Palawan, 3 May 1931.

**Remarks.** A photograph and a radiograph of the holotype are available in the CAS record.

**(1204) *Pseudoplesiopsimmaculatus* Gill & Edwards, 2002: 20, figs 1, 2**

**Holotype.** AMS I.20757-069. Raine Island, Great Barrier Reef, Australia.

**Paratype.**USNM 291609 (1, 25.7), GDJ 87-12, White Beach, past Mahatao, Batan Island, Batanes, 9–12 m, 1 May 1987.

**(1205) *Pseudoplesiopssargenti* Schultz in Schultz et al., 1953: 405, fig. 67**

= *Pseudoplesiopstypus* Bleeker, 1858.

**Paratypes.** SAIAB 805 [ex RUSI] [USNM 146520] (1), Biri Channel, Samar Island, 2–3 m, 1 Jun. 1909; USNM 146515 (2), Batan Is, Caracaran Bay, Batanes, 2–3 m, 8 Jun. 1909; USNM 146516 (1), Endeavor Straits, Malampaya, Taytay, Palawan Island, 23 Dec. 1908; USNM 146517 (2), Port Matalvi, Zambales, Luzon, 23 Nov. 1908; USNM 146518 (2), Rasa Island, Narra, Palawan Island, 2–4 m, 1 Apr. 1909; USNM 146519 (1), Port Palapag, Batag Island, Samar, 2–5 m, 3 Jun. 1909; USNM 146521 (2), Romblon Harbor, Romblon, 2–5 m, 25 Mar. 1908.

##### Family Opistognathidae (290)

**(1206) *Gnathypopsdendritica* Jordan & Richardson, 1908: 261, fig. 9**

= *Opistognathusdendriticus* (Jordan & Richardson, 1908).

**Holotype.**CAS-SU 20313 (133.4), Cuyo Island, Palawan.

**Remarks.** The original description or CAS record did not indicate the collection date. A photograph and a radiograph are available in the CAS record.

**(1207) *Opistognathusalbomaculatus* Smith-Vaniz, 2023: 36, figs 16C, 43–45**

**Holotype.**USNM 357198. Duff Island, Santa Cruz Islands, Solomon Islands.

**Paratypes.**USNM 320260 (1), SP 78-21, Bararin Island, Cuyo, Palawan, 0–17 m, 24 May 1978; USNM 396246 (1), BUS 03-39, ~20 miles west of Busuanga Island, Palawan, 25–37 m, 13 Mar. 2003; USNM 396236 (6), off southeast side of West Nalaut Island off western Busuanga, Palawan, 10–15 m, 11 Mar. 2003; USNM 436245 (1, 44.0), VERDE-2015-22, off western entrance to Manila Channel, Puerto Galera, Mindoro Island, 11–12 m, 12 Apr. 2015.

**(1208) *Opistognathushyalinus* Smith-Vaniz, 2023: 63, figs 10A, 16S, 20A, B, 90–92**

**Holotype.**PNM 15647 [ex USNM 315660], 1 km west of Larena, Siquijor Island.

**Paratypes.**USNM 315661 [ex USNM 315660] (1), LK 79-16, 1 km west of Larena, Siquijor Island, 26–30 m, 15 May 1979.

**(1209) *Opistognathusrandalli* Smith-Vaniz, 2009: 87, figs 1c, 2d, 3c, 4c–d, 19–14**

**Holotype.**ANSP 142963, Cocoro Island (10°53.15'N, 121°11.60'E), Cuyo, Palawan, 21 m, 1978.

**Paratypes.** BPBM 20796 (2) and BPBM 20797 (1), Sumilon Island, Cebu, 30 m, 25–26 Aug. 1977; BPBM 26838 (2), same locality as BPBM 20796, 3 Jun. 1981; FMNH 118179 (5), Tara Island, Coron, Palawan, 4 Mar. 2003; ROM 52745 (1), Siquijor Island, 10 May 1987; USNM 113147 (1), Murcielagos Bay, northern Mindanao, 9 Aug. 1909; USNM 122378 (1), Lagonoy Gulf, near Palag Bay, Luzon, 8 m, 16 Jun. 1909; USNM 122379 (1), Port Galera, Galera Bay, Mindoro Island, 27 Oct. 1909; USNM 150734 (2), same locality as USNM 122379, 9 Jun. 1908; USNM 150736 (1), same locality as USNM 122379, 27 Oct 1909; USNM 220354 (4), southwest of Apo Island, Negros, 11 May 1979; USNM 365704 (2), Maestro de Campo Island, Concepcion, Romblon, 28 m, 29 May 2000; USNM 373245 (1), Apo Reef, Mindoro Island, 33 m, 3 Mar. 2003.

**Remarks.** Photographs and radiographs of the holotype are available in ANSP’s record.

**(1210) *Opisthognathussuluensis* Herre, 1933: 25**

= *Opistognathuscastelnaui* Bleeker, 1859.

**Holotype.**CAS-SU 25508 (60.0), Sitankai Island, Sulu Archipelago, Aug. 1931.

**Paratype.**CAS-SU 17467 (1, 140.0), same data as holotype.

**Remarks.** A photograph and a radiograph of the holotype are available in the CAS record.

**(1211) *Opistognathusvariabilis* Smith-Vaniz, 2009: 92, figs 1e–f, 2e, 3d, 4e–f, 25–43**

**Holotype.** NCIP 6348. Tanjung Bobo, Halmahera, Indonesia.

**Paratypes.** FMNH 118180 (2) and USNM 373246 (2), Elet Island, Busuanga, Palawan, 10–30 m, 14 Mar. 2003; USNM 373246 (2) and USNM 393590 (1, 56.3), Dolarog, El Nido, Palawan Island, 17 m, 12 Jun. 2007; USNM 122420 (1), South Point Reef, Pangasinan Island, Jolo, Sulu, 2–4 m, 13 Feb. 1908.

**(1212) *Opistognathuswassi* Smith-Vaniz, 2023: 100, figs 13C, 18L, 19C–D, 167–170**

**Holotype.**ANSP 133406. Tafagamanu Point, Tutuila Island, American Samoa.

**Paratypes.**USNM 346209 (1), JTW 95-10, off rocky shore at Tando, off Lusaron Point, Guimaras Island, 14–20 m, 28 Sep. 1995; USNM 377438 (1), Langawn Reef, ~ 5 km east of Mansalay Bay, Mindoro Island, 10–26 m, 30 May 2000; USNM 379155 (1), MIN 00-60, Mindoro Island, 3–31.5 m, 2 Jun. 2000.

## ﻿﻿Additional information

### Author ORCIDs

Rodulf Anthony T. Balisco https://orcid.org/0000-0001-9503-0912

Te-Yu Liao https://orcid.org/0000-0001-7588-200X

### Data availability

All data that support the findings of this study are available in the main text or Supplementary Information.

## Supplementary materials

﻿Supplementary material 1 Species list Data type xlsx Explanation note **table S1.** List of all nominal species described from the Philippines, current status, & current valid name. Note: V–valid, VE–endemic to the Philippine waters, X–invalid. **table S2.** List of fish species described from the Philippines with uncertain/ unknown status. More specimens and studies are needed to ascertain the status of these nominal species. https://binary.pensoft.net/file/1378669This dataset is made available under the Open Database License (http://opendatacommons.org/licenses/odbl/1.0/). The Open Database License (ODbL) is a license agreement intended to allow users to freely share, modify, and use this Dataset while maintaining this same freedom for others, provided that the original source and author(s) are credited.Authors. Rodulf Anthony T. Balisco, Te-Yu Liao
